# Synthesis and In Vitro Evaluation of C-7 and C-8 Luteolin Derivatives as Influenza Endonuclease Inhibitors

**DOI:** 10.3390/ijms22147735

**Published:** 2021-07-20

**Authors:** Robert Reiberger, Kateřina Radilová, Michal Kráľ, Václav Zima, Pavel Majer, Jiří Brynda, Martin Dračínský, Jan Konvalinka, Milan Kožíšek, Aleš Machara

**Affiliations:** 1 Institute of Organic Chemistry and Biochemistry of the Czech Academy of Sciences, Gilead Sciences and IOCB Research Center, Flemingovo n. 2, 166 10 Prague, Czech Republic; robert.reiberger@uochb.cas.cz (R.R.); katerina.radilova@uochb.cas.cz (K.R.); michal.kral@uochb.cas.cz (M.K.); vaclav.zima@uochb.cas.cz (V.Z.); pavel.majer@uochb.cas.cz (P.M.); jiri.brynda@uochb.cas.cz (J.B.); martin.dracinsky@uochb.cas.cz (M.D.); jan.konvalinka@uochb.cas.cz (J.K.); 2 Department of Organic Chemistry, Faculty of Science, Charles University, Hlavova 8, 128 00 Prague, Czech Republic; 3 First Faculty of Medicine, Charles University, Kateřinská 1660, 121 08 Prague, Czech Republic; 4 Institute of Molecular Genetics of the Czech Academy of Sciences, Vídeňská 1083, 140 00 Prague, Czech Republic; 5 Department of Biochemistry, Faculty of Science, Charles University, Hlavova 8, 128 00 Prague, Czech Republic

**Keywords:** bio-isosterism, cross-coupling, endonuclease inhibitor, flavonoids, influenza, Mannich reaction, RNA polymerase

## Abstract

The part of the influenza polymerase PA subunit featuring endonuclease activity is a target for anti-influenza therapies, including the FDA-approved drug Xofluza. A general feature of endonuclease inhibitors is their ability to chelate Mg^2+^ or Mn^2+^ ions located in the enzyme’s catalytic site. Previously, we screened a panel of flavonoids for PA inhibition and found luteolin and its C-glucoside orientin to be potent inhibitors. Through structural analysis, we identified the presence of a 3′,4′-dihydroxyphenyl moiety as a crucial feature for sub-micromolar inhibitory activity. Here, we report results from a subsequent investigation exploring structural changes at the C-7 and C-8 positions of luteolin. Experimental IC_50_ values were determined by AlphaScreen technology. The most potent inhibitors were C-8 derivatives with inhibitory potencies comparable to that of luteolin. Bio-isosteric replacement of the C-7 hydroxyl moiety of luteolin led to a series of compounds with one-order-of-magnitude-lower inhibitory potencies. Using X-ray crystallography, we solved structures of the wild-type PA-N-terminal domain and its I38T mutant in complex with orientin at 1.9 Å and 2.2 Å resolution, respectively.

## 1. Introduction

Influenza viruses cause illness in a variety of species, including humans. Despite the availability of vaccines and antiviral drugs, influenza remains a serious threat to human health, causing 290,000–650,000 deaths worldwide annually [1]. Influenza virus RNA-dependent RNA polymerase (RdRP) lacks proof-reading activity, which leads to an accumulation of point mutations known as antigenic drift. This is responsible for the emergence of new viral variants causing seasonal flu, which requires the flu vaccine to be reformulated every year. Antigenic drift also contributes to increasing viral resistance against antiviral drugs. Additionally, antigenic shift―the reassortment of viral RNA segments from two or more different influenza strains in animals or humans―could lead to a new pandemic strain. In fact, various zoonotic strains are considered “ticking time bombs”, as these pathogens have the potential to mutate to facilitate human transmission given enough time and infected organisms. Generally, vaccination is the best intervention against viral pathogens including influenza. However, the last influenza pandemic in 2009 reminded us that effective vaccines are often not available at the onset of a pandemic. Reformulation of vaccines is time-consuming, and many human lives can be taken by the disease during this process. The intricacies of vaccine development, in combination with influenza’s genomic variability, makes the development of novel anti-influenza therapeutics imperative.

Influenza viruses contain a single-stranded, negative-sense RNA genome in complex with RdRP [2,3,4]. RdRP comprises the subunits PA (polymerase acidic protein), PB1 and PB2 (polymerase basic protein 1 and 2). The virus itself is unable to synthesize the 5′-mRNA cap required for eukaryotic translation; this represents the “Achillies heel” of influenza virus. The virus obtains host primers―short oligomers of host pre-mRNA that initiate transcription―by a unique “cap-snatching” mechanism [5,6,7,8,9], which serves as a target for pharmaceutical intervention. The process begins with binding of the PB2 subunit to the 5′-cap (m^7^GTP) of the host pre-mRNA. Subsequently, the PA subunit cleaves the RNA strain approximately 10–13 nucleotides downstream from the 5′-cap to acquire the cap/primer [10]. The PB1 subunit uses this detached RNA segment as a template for viral mRNA synthesis. RdRP is highly conserved across influenza strains, and the three subunits involved in the cap-snatching mechanism have been recognized as attractive targets for drug development in the past decade [11,12,13,14,15,16,17,18].

The PA subunit is a bridged binuclear metalloenzyme with an N-terminal domain (PA-Nter) harboring the endonuclease active site that carries out cleavage of the RNA segment. The active site is a negatively charged pocket that accommodates either Mg^2+^ or Mn^2+^ ions, with stronger affinity for the latter [19]. These ions are critical for endonuclease activity. Evidence suggests that PA-Nter endonuclease inhibitors must possess a metal-binding pharmacophore with the ability to bind either Mg^2+^ or Mn^2+^ ions efficiently [9].

Even though metalloenzymes comprise more than one-third of all known enzymes, clinical development of metalloenzyme inhibitors is rather limited and few such inhibitors have been approved by the Food and Drug Administration (FDA) [9,20,21,22]. Less than 5% of all FDA-approved drugs target metalloenzymes [23]. Unsurprisingly, metal-binding pharmacophores exhibit a lack of structural diversity.

**Figure 1:**
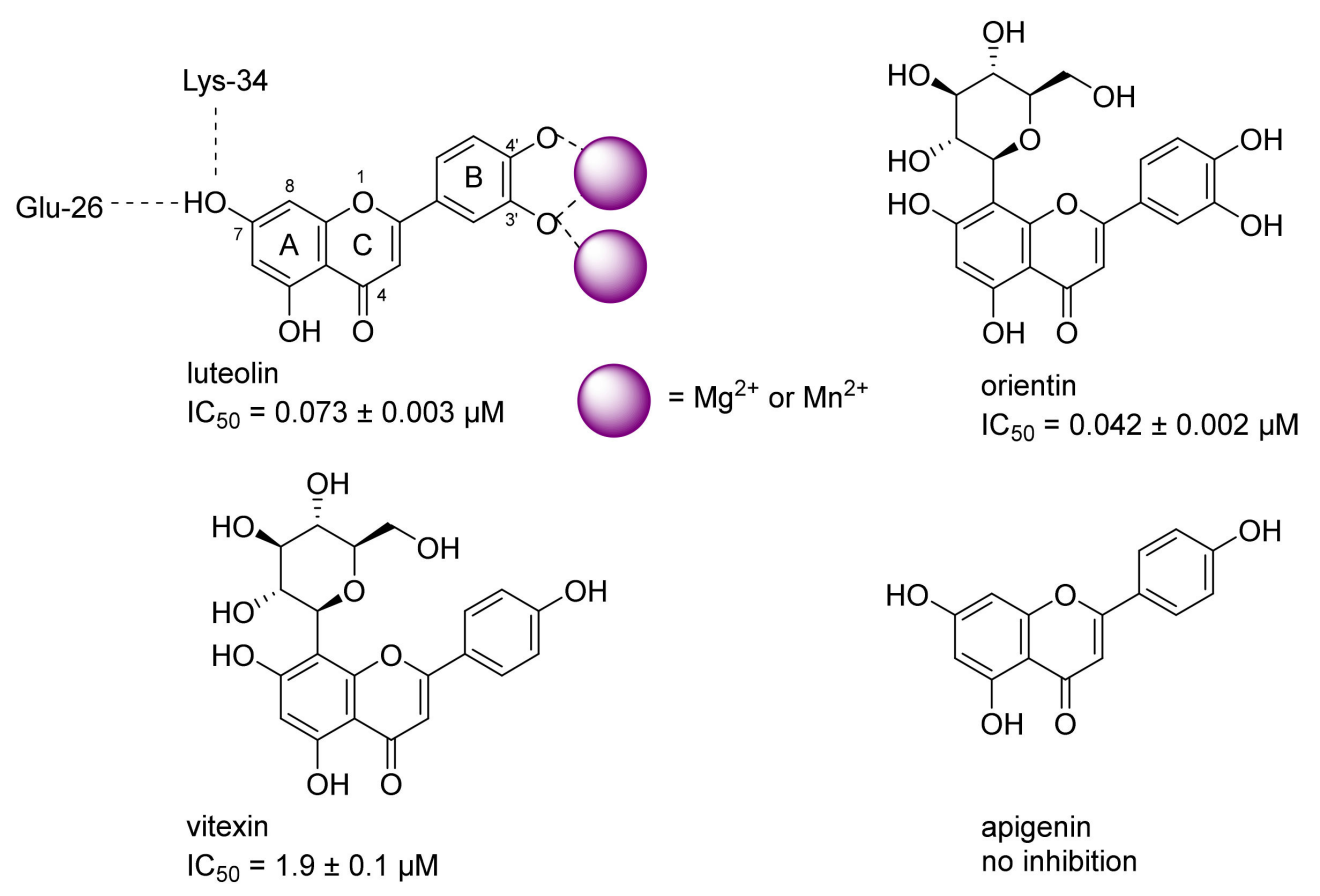
Selected hits from previous screening campaigns are shown. The tight coordination of metal ions and interactions with PA-Nter residues are indicated on the structure of luteolin.

There is currently only one FDA-approved influenza endonuclease inhibitor: baloxavir marboxil (trade name Xofluza), which is administrated as a prodrug [24,25]. Successful antiviral drugs must be able to block at least the existing variant of the target enzyme and should have a high resistance barrier. One key mutation in the influenza A/H1N1 2009 pandemic (A/H1N1pdm) and A/H3N2 viruses is Ile-38 to Thr-38 in PA-Nter. This mutation reduced patient susceptibility to baloxavir marboxil and impaired the virus’ replicative fitness in cells [26]. Resistance development could eventually lead to the loss of the clinical relevance of Xofluza. Furthermore, fewer than a dozen PA-Nter endonuclease inhibitor classes have been reported in the literature to date. These classes include diketo acids [27], dopamine derivatives [28], hydroxylated heterocycles [29,30,31], flutimide congeners [32], green tea catechins [33,34], catechol derivatives [35], hydroxylated *N*-acyl-hydrazones [36] and others [18]. Recently, we identified the molecular mode of action of flavonoids in influenza-infected cells [37]. We developed a screening assay based on AlphaScreen technology and determined the inhibitory potency of 38 flavonoids, of which luteolin (IC_50_ of 73 ± 3 nM) and its 8-*C*-glucoside orientin (IC_50_ of 42 ± 2 nM) were the most potent inhibitors (Figure 1). A gel-based endonuclease inhibitory assay confirmed our findings from the competition assay. Finally, we performed structural analysis of complexes of PA-Nter with luteolin and myricetin and described various binding poses of flavonoids in the PA-Nter active site.

In the current study, we aimed to pursue structure-assisted drug design guided by our previously reported crystal structure of PA-Nter in complex with luteolin (PDB entry 6YA5, 2.0 Å resolution) [37]. The structure revealed that the hydroxyl group at position C-7 forms a hydrogen bond with the Glu-26 residue of PA-Nter. The surface complementarity and the strong interaction of hydroxyls at the B-ring with metal ions contribute to the high affinity and inhibitory potency of luteolin. Thus, luteolin may serve as a useful scaffold to introduce modifications that improve the inhibitory potency and pharmacokinetic properties. Luteolin itself has low bioavailability [38,39] and is prone to oxidation. Therefore, we replaced the C-7 hydroxyl with other moieties capable of creating hydrogen bonds with Glu-26. Bio-isosteric replacement is a proven tool for modulating the drug-like properties of promising therapeutics [40]. The C-7 hydroxyl group has slightly higher acidity [41] compared to the other hydroxyls of luteolin. This allowed us to selectively modify the C-7 position using Pd-catalyzed cross couplings via the corresponding C-7 triflate.

We then continued to leverage the known structure–activity relationships (SAR) in 3′,4′-dihydroxyphenyl flavones. Moreover, we expanded our research effort to explore the chemical space around the C-8 position of the luteolin scaffold. Specifically, we set out to investigate whether moieties introduced at the C-8 position by Mannich reaction [42] would be tolerated or even enhance inhibitory potency. Our goal was to find additional points of interaction between luteolin derivatives and PA-Nter and, more importantly, to explain why vitexin exhibits moderate inhibitory potency even though it does not have the 3′,4′-dihydroxyphenyl motif. This ortho-hydroquinone motif was originally identified as the metal-binding pharmacophore and as such was considered indispensable for inhibition. That paradigm explains the lack of inhibitory potency of apigenin but fails to rationalize vitexin’s moderate potency (see Figure 1).

## 2. Results and Discussion

### 2.1. Compound Synthesis

**Scheme 1:**
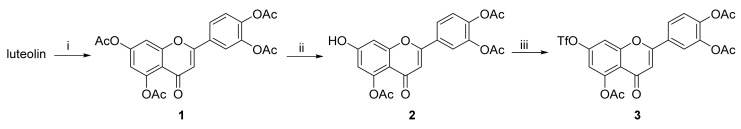
Reagents and conditions: (i) Ac_2_O, pyridine, 145 °C, 3 h, 77%; (ii) thiophenol, imidazole, NMP/THF (1:3), 0 °C to r.t., 5 h, 68%; (iii) Tf_2_O, pyridine, DCM, 0 °C, 3.5 h, 41%.

Based on the SAR outlined in Figure 1, our initial efforts focused on exploring bio-isosterism at the C-7 moiety of luteolin. Guided by general knowledge of hydrogen bond formation and prior crystallographic study, we proposed that the hydroxyl group could be replaced with a small group featuring either N-H or O-H bonds. We also decided to introduce heterocyclic moieties that are not necessarily associated with textbook OH bio-isosteric replacement. Preparation of this C-7 series started from luteolin, which was per-acetylated. Compound **1** was then selectively mono-deacetylated at C-7 using previously reported conditions [43] (Scheme 1). This regioselective deprotection takes advantage of the higher acidity of the C-7 hydroxyl group in the luteolin scaffold caused by the electron-withdrawing pyrone carbonyl moiety in the para position. Therefore, thiophenolate-mediated *O*-deacetylation selectively generates the corresponding phenolate (**2**), which was next treated with triflic anhydride to provide intermediate **3** in a decent yield.

**Scheme 2:**
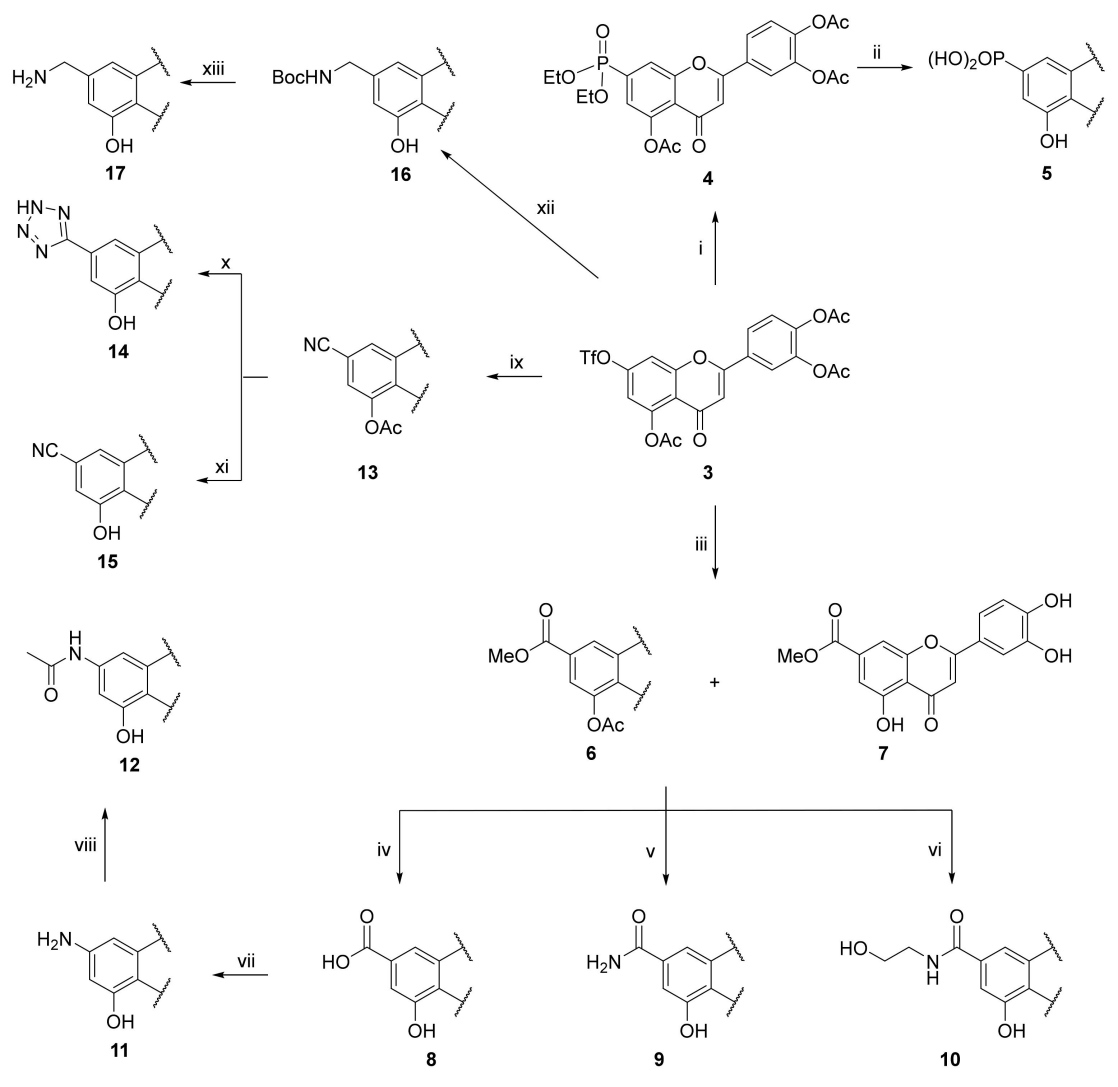
Reagents and conditions: (i) Pd(PPh_3_)_4_, (EtO)_2_P(O)H, DIPEA, MeCN, 70 °C, 3 h, 78%; (ii) (a) TMS-Br, DCM, r.t., 32 h, (b) MeOH, r.t., 1 h, (c) KOH, THF/MeOH, r.t., 1 h, 25%; (iii) Mo(CO)_6_, Pd(OAc)_2_, dppp, Et_3_N, DMSO/MeOH (3:2), 70 °C, 20 h, 40% (**6**), 48% (**7**); (iv) 2M LiOH (aq.), THF, 45 °C, 1 h, 71%; (v) 7M NH_3_ (MeOH), 50 °C, 4 d, 21%; (vi) 2-aminoethanol, K_2_CO_3_, EtOH, 50 °C, 24 h, 23%; (vii) a) DPPA, Et_3_N, DMF, 100 °C, 16 h, b) H_2_O, DMF, 100 °C, 3 h, 30%; (viii) AcOSu, Et_3_N, DMF, r.t., 36 h, 23%; (ix) Zn(CN)_2_, XantPhos Pd G2, DIPEA, DMF, 85 °C, 24 h, 61%; (x) NaN_3_, AcOH, DMF, 130 °C, 3h, 38%; (xi) 2M LiOH (aq.), THF, r.t., 24 h, 46%; (xii) potassium [[(*t*-butoxy-carbonyl)amino]methyl]trifluoroborate, SPhos Pd G2, K_2_CO_3_, THF/H_2_O (25:1), 100 °C, 2 h, 43%; (xiii) TFA, THF/H_2_O (4:1), 50 °C, 48 h, 73%.

Triflate **3** was subjected to a wide range of palladium-catalyzed cross-couplings. In addition to the desired products, certain reactions also yielded C-5 *O*-deacetylated analogues (Scheme 2). However, formation of such byproducts was not an obstacle, because we subsequently performed global deprotections of the flavone scaffolds. Hirao coupling [44] of **3** resulted in a high yield of diethyl phosphonate **4**. Subsequent standard dealkylation using trimethylsilyl bromide [45] resulted in phosphonic acid **5**. Molybdenum hexacarbonyl was used as a source of carbon monoxide for Pd-catalyzed methoxy-carbonylation. This approach was superior to methoxycarbonylation with dicobaltoctacarbonyl [46] in terms of yield and subsequent cleaning of the reaction apparatus. The pre-purified mixture of methyl esters **6** and **7** was subjected to hydrolysis and to different aminolysis reactions, which produced **8**, **9** and **10**.

One-pot Curtius rearrangement converted 3,4,5-trihydroxyflavone-7-carboxylic acid (**8**) to amino-derivative **11** in an acceptable yield of 30%. In general, electron rich anilines are prone to quick degradation, and thus the corresponding acetamide **12** was prepared from **11** using AcOSu. Palladium-catalyzed cyanation of triflate **3** by a modified Buchwald’s procedure [47] gave rise to tetrazole derivative **14** and nitrile **15**. Amino-methyl transfer to triflate **3** was accomplished by protected potassium aminomethyltrifluoroborate under slightly modified conditions, reported by Molander [48]. The crude product of cross-coupling was then subjected to trifluoroacetic-acid-mediated de-tert-butylation, affording **17** as a homolog of amine **11**.

**Scheme 3:**
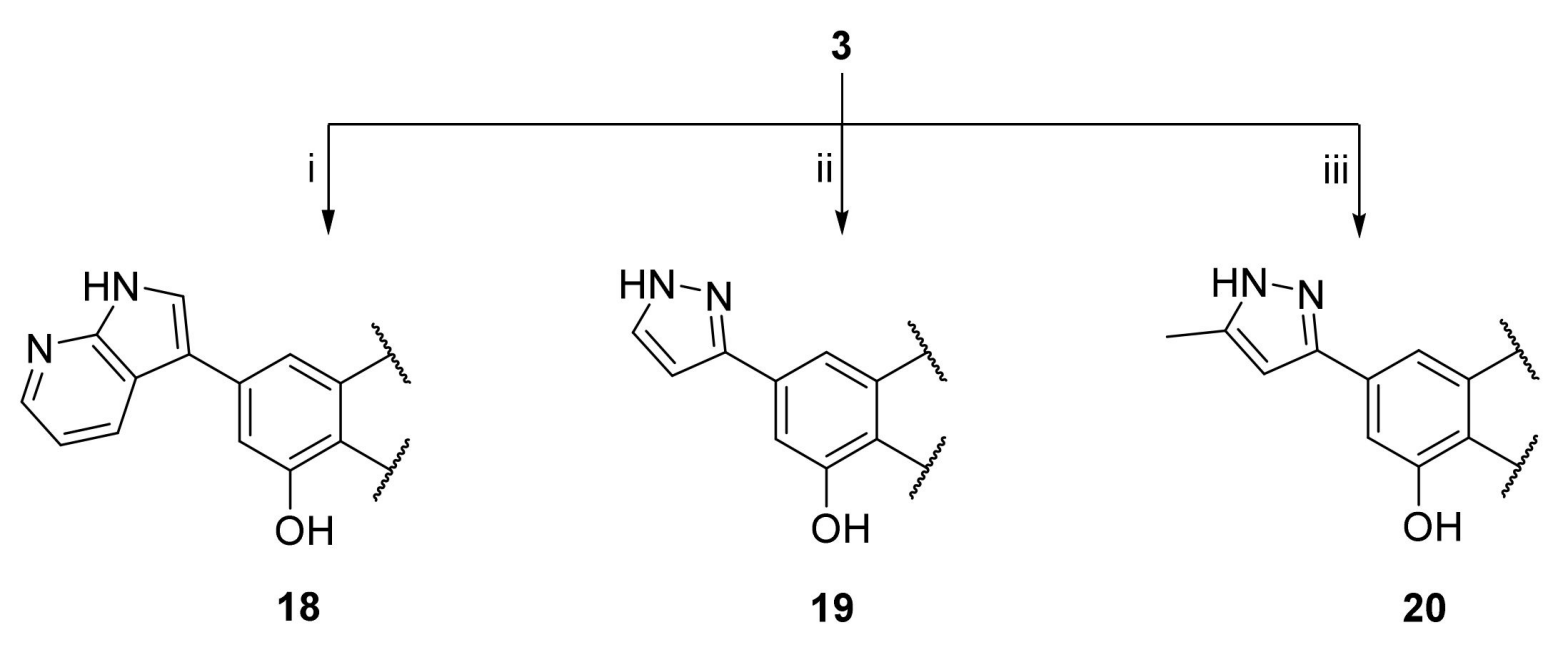
Reagents and conditions: (i) 3-(4,4,5,5-tetramethyl-1,3,2-dioxaborolan-2-yl)-1*H*-pyrrolo[2,3-*b*]pyridine, Pd(PPh_3_)_4_, Cs_2_CO_3_, DMF, 90 °C, 16 h, 10%; (ii) (1*H*-pyrazol-3-yl)boronic acid, SPhos Pd G2, K_2_CO_3_, THF, 100 °C, 16 h, 25%; (iii) (5-methyl-1*H*-pyrazol-3-yl)boronic acid, SPhos Pd G2, K_2_CO_3_, THF/H_2_O (25:1), 100 °C, 16 h, 19%.

Our well-established method for preparation of the key intermediate **3** enabled us to execute heteroarylations of the flavone scaffold. We hypothesized that replacement of the hydroxyl group with pyrazole and azaindole could, in principle, ensure engagement of Glu-26 while improving the metabolic stability of the flavone. Synthesis of **18**–**20** was mediated by Pd(PPh_3_)_4_ or by Buchwald 2nd generation pre-catalysts. Regardless of which catalyst was used, **18**–**20** were obtained in low yields (Scheme 3).

Although efficient Mannich reactions with luteolin [49] and quercetin [50,51] have been previously described, we found this type of C–C formation reaction very challenging. In our hands, formation of the desired products was in all cases accompanied by formation of C-6 regioisomers, contrary to reports in the literature. We also observed formation of other byproducts with a methylendioxy group on the B-ring serving as a 2:1 adduct of formalin to luteolin. The very limited solubility of luteolin further complicated our effort to optimize the Mannich reaction. Methanol worked least poorly among a variety of solvents screened (methanol, ethanol, propan-2-ol, trifluoroethanol, 1,4-dioxane and DMF). Room temperature and equimolar loading of formalin and the secondary amine with respect to luteolin efficiently suppressed the double Mannich reaction at both the C-6 and C-8 positions. To avoid issues with low regioselectivity and to exclude methyl-endioxy formation, we attempted Mannich reactions with tetraacetate **1** and triacetate **2**. These reactions led to complex reaction mixtures due to low conversions and the lability of acetates in the presence of secondary amines. According to HPLC analysis, subsequent methanolysis resulted in a simplified mixture of compounds, but yields of products were low (~15%). Considering the need to prepare triacetate **2**, this approach is clearly not advantageous to the Mannich reaction of parental luteolin in methanol. Data resulting from screening of the Mannich reaction with **1** and **2** are provided in the Supplementary Materials.

**Scheme 4:**
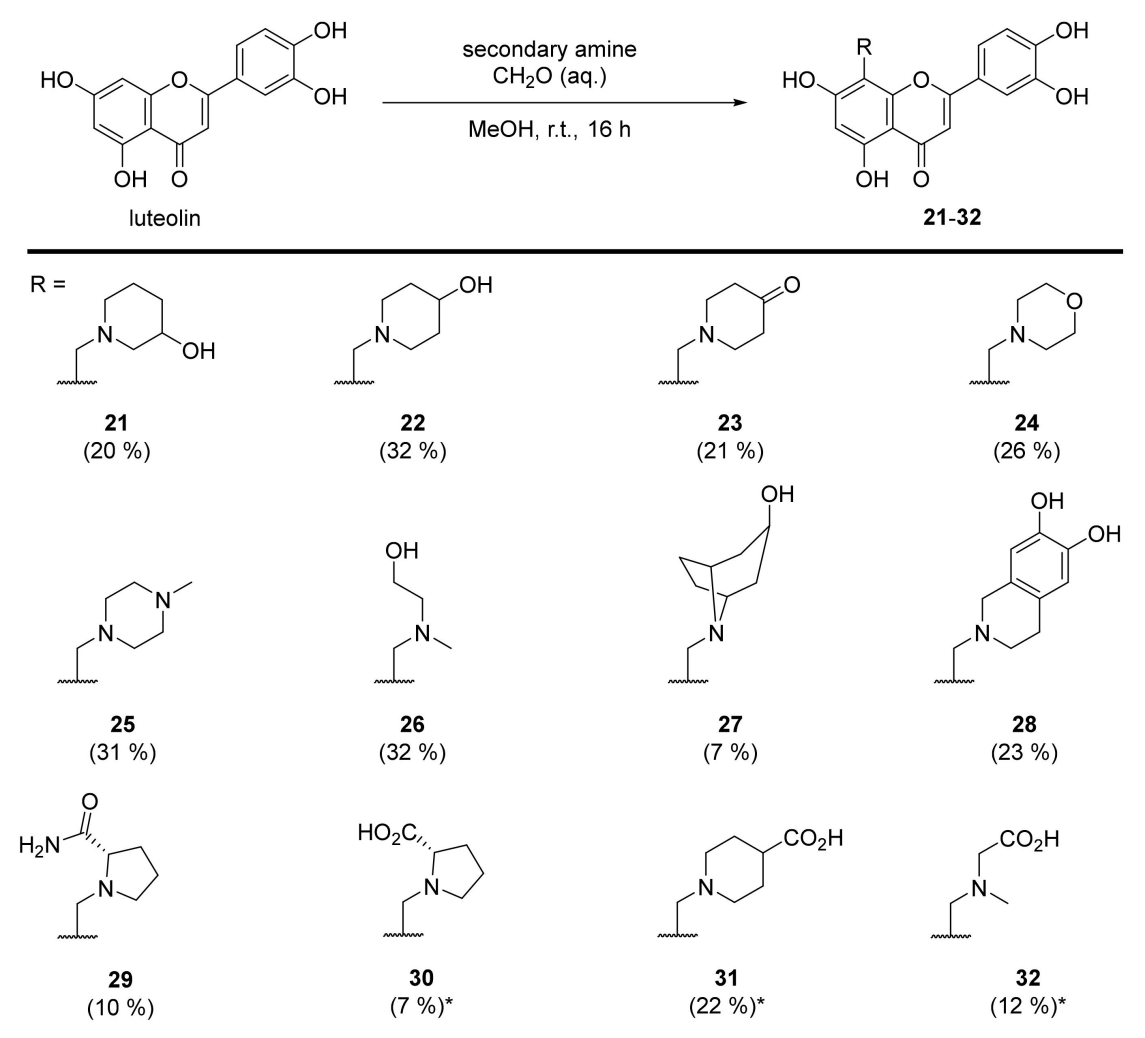
Mannich reaction of luteolin with secondary amines in methanol. The asterisk indicates the overall yield of the Mannich reaction with subsequent acidic hydrolysis of corresponding alkyl esters.

To explore the chemical space around C-8 as much as possible, we used structurally different secondary amines possessing additional functional groups (Scheme 4). Mannich reactions were stirred at room temperature overnight until almost all luteolin was consumed. The ratio between C-8 Mannich adduct and unwanted C-6 regioisomer ranged from 1.7:1 to 3.0:1. Since both regioisomers have similar retention factors, the purification of **21**–**32** was very laborious. Products were purified by multiple HPLC preparations that further negatively influenced the yields. Alkyl esters of secondary amines were used for preparation of **30**–**32**, and after the Mannich reaction, these ester moieties were trans-formed into carboxylic acids.

### 2.2. Relationship between Chemical Structure and Inhibitory Potency

**Figure 2:**
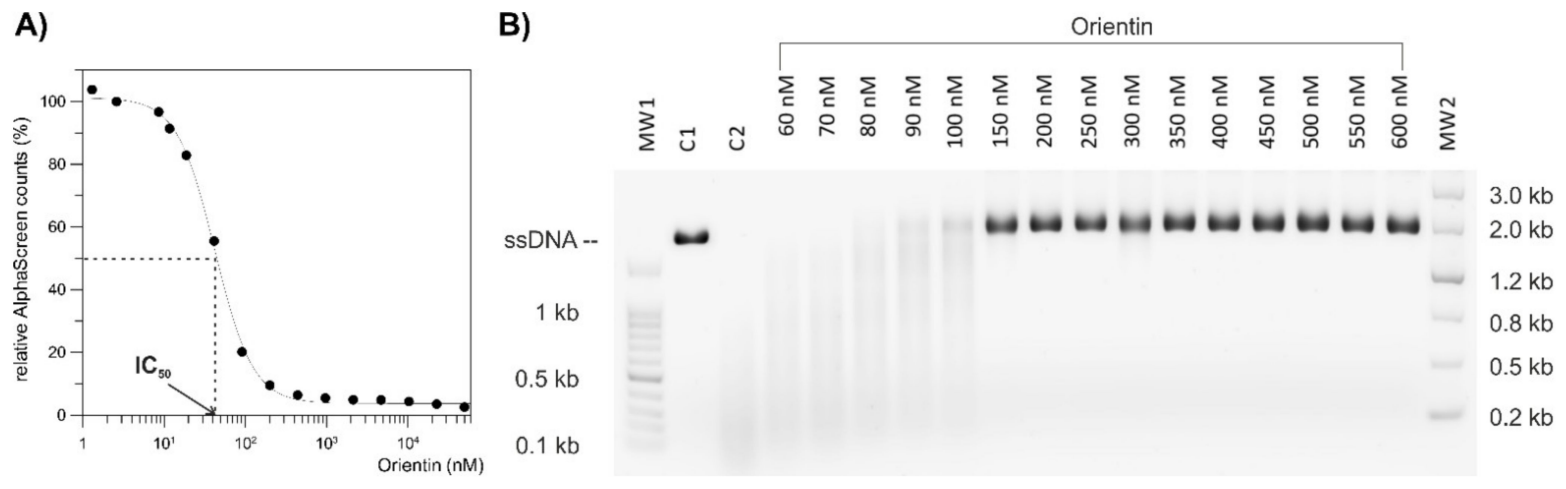
(**A**) Titration curve of orientin using the AlphaScreen assay, revealing an IC_50_ value of 42 nM. (**B**) Inhibition of endonuclease activity by orientin, documented by a gel-based endonuclease inhibitory assay. The single-stranded circular DNA M13mp18 was used as substrate (lane C1). The substrate fully cleaved by endonuclease in the absence of inhibitor (lane C2) was used as a control. The GelPilot 100 bp Plus Ladder molecular weight marker (lane MW1) and the GelPilot Wide Range Ladder molecular weight marker (lane MW2) are shown for reference.

The inhibition potencies of the prepared compounds were assessed by an assay that we recently developed for screening PA-Nter inhibitors based on the amplified luminescent proximity assay system (AlphaScreen) [37]. Examples of titration curves are shown in Figure 2A and Figure S1. The series of C-7 luteolin congeners displayed moderate inhibitory potencies with one- to two-order-of-magnitude higher IC_50_ values compared to luteolin (Table 1).

Phosphonic acid **5** inhibited PA-Nter with an IC_50_ of 9.2 μM, which was one of the weakest inhibitory potencies of the whole series. On the other hand, carboxylic acid **8** was a sub-micromolar inhibitor (IC_50_ = 0.98 μM). Related amides **9** and **10** were less potent than **8** (IC_50_ = 2.0 and 7.6 μM, respectively), which might indicate relatively confined C-7 proximal space. The amino derivative **11** had approximately 20-times weaker binding potency than luteolin. Clearly, the anticipated interaction between the C-7 amino group and side chain of Glu-26 did not result in superior binding. We speculate that solvation of the protonated amino group disrupted the effective formation of a salt bridge with Glu-26. Acetamide **12**, tetrazole derivative **14**, and nitrile **15** exhibited relatively flat SAR with IC_50_ values of 3.0, 1.4 and 1.3 μM, respectively. Amino-methyl derivative **17** exhibited a significantly decreased inhibitory activity compared to luteolin (IC_50_ = 6.7 μM). Apparently, neither strongly acidic nor basic moieties at C-7 are tolerated (see the inhibitory potencies of phosphonic acid **5** and aminomethyl derivative **17**). The relatively bulky azaindole congener **18** exhibited appreciable inhibitory potency with an IC_50_ value of 3.4 μM. Pyrazoles **19** and **20** differed slightly in inhibitory potency; the former was more potent than its methylated analogue **20,** but both exhibited IC_50_ values comparable with that of tetrazole **14** (0.81 and 2.6 versus 1.4 μM). Considering the almost identical half-maximal inhibitory concentrations of weakly basic pyrazoles **19** and **20** and acidic tetrazole **14**, we surmise that interaction with PA-Nter is not susceptible to the “proton affinity” of the moiety introduced at C-7. The inhibitory potencies of **5** and **17** further strengthen our hypothesis. On the other hand, it seems that steric effects play a key role in this specific point of interaction with the endonuclease. To confirm this, we screened cynaroside, a commercially available luteolin 7-*O*-β-D-glucoside. The introduction of a bulky glucose residue at C-7 led to an almost complete loss of inhibition (IC_50_ = 32 ± 3 μM; three orders of magnitude worse than luteolin). We conclude that relocation of amino acids surrounding Glu-26 within PA-Nter and/or a clash with the Glu-26 side chain leads to a significant drop of inhibitory potency.

Next, we assessed the effect of C-8 substituents. As shown in Table 2, almost all aminomethylene moieties were well-tolerated. Compounds **21**, **23**–**25** and **29**–**32** exhibited inhibitory potencies roughly comparable to that of orientin (IC_50_ = 0.042 μM). Moieties with additional basic (**25**) or acidic residues (**30**–**32**) had IC_50_ values within the same range. This suggests the lack of a PA-Nter amino acid featuring proton affinity in the chemical space around C-8. The bulky nor-tropine (**27**, IC_50_ = 0.075 μM) and dihydroxytetrahydro-isoquinoline (**28**, IC_50_ = 0.12 μM) scaffolds were not found to be significantly unfavorable. It is likely that both bulky residues are oriented away from the PA-Nter active site and into the solvent. This hypothesis fits well with the findings presented in Section 2.3. 4-Hydroxypiperidine derivative **22** showed a slightly decreased potency in comparison with 3-hydroxy analogue **21** (0.14 *versus* 0.083 μM). However, the micromolar inhibitory potency of alcohol **26** (IC_50_ = 1.2 μM) has no rational explanation based on the acquired SAR. Rather, we anticipated that **26**, as a cyclic analogue of **21**, would be a sub-micromolar inhibitor.

To assess the inhibition of endonuclease activity by selected compounds by a direct mechanism-based method, we applied a gel-based endonuclease inhibitory assay (Figure 2B). This assay was performed for five selected ligands (orientin, baloxavir acid, luteolin, **21** and **30**) with wild-type PA-Nter (see Supplementary Materials, Figure S2). The analysis showed that orientin had a higher inhibitory potency than luteolin, in agreement with the AlphaScreen assay. The gel-based assay also confirmed similar inhibition activities for baloxavir acid, orientin, **21** and **30**. We also attempted this assay with the PA-Nter I38T variant, but the mutation of Ile-38 to Thr-38 led to complete loss of ssDNA cleavage ability (see Figure S3). Thus, we used a fluorescent-labelled ssRNA substrate and performed a FRET-based endonuclease assay with this variant. Compared to wild-type PA-Nter, the I38T variant had only 1.9% activity (see Figure S4) toward the ssRNA substrate and none for ssDNA. This result is in line with a recently reported observation of significantly reduced fitness and ssRNA nuclease activity of a virus harboring the I38T variant [26].

### 2.3. Crystal Structures of Wild-Type and I38T PA-Nter Domains in Complex with Orientin

**Figure 3:**
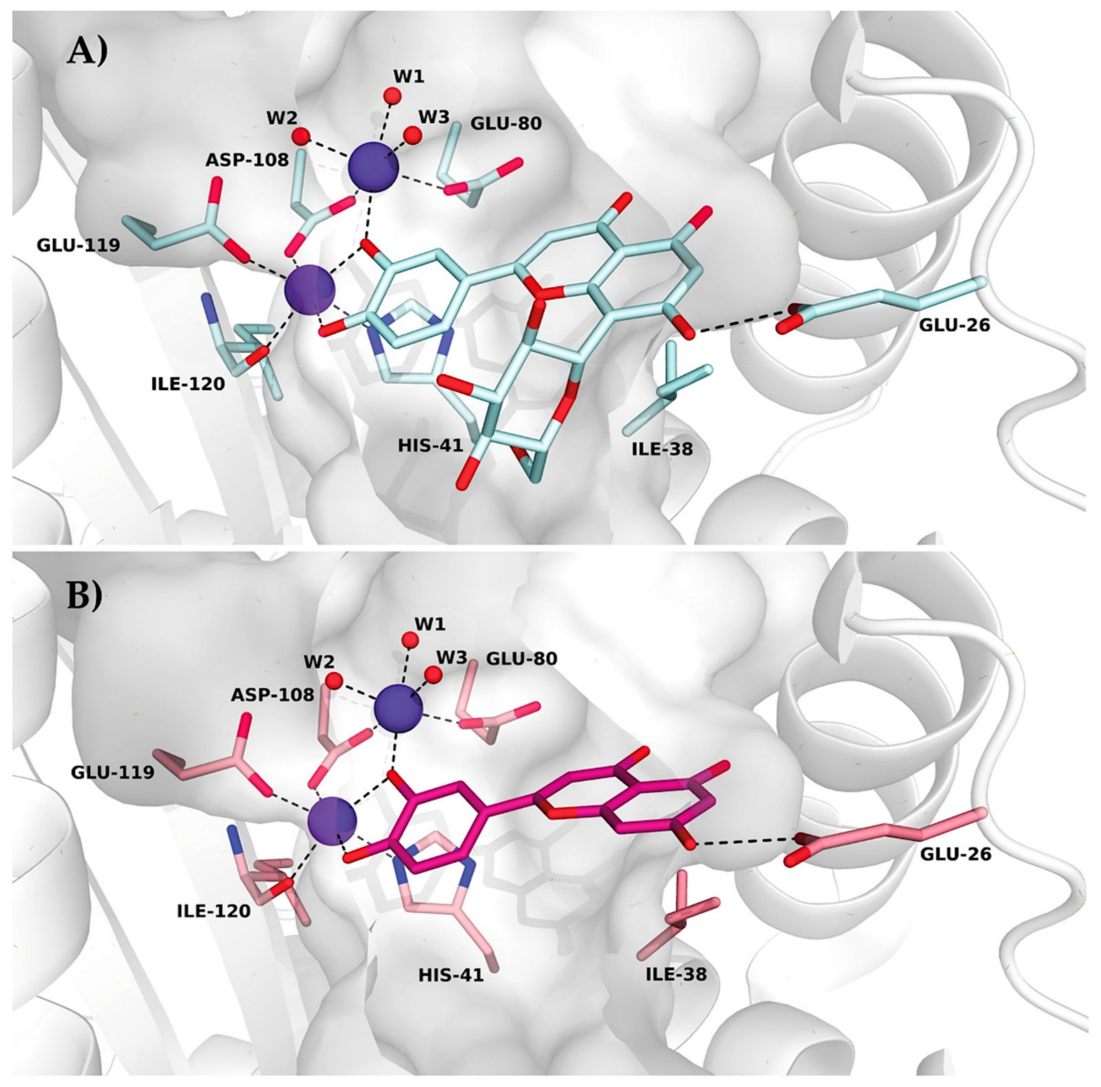
Comparison of crystal structures of PA-Nter in complex with orientin (**A**; PDB ID 7NUG) and luteolin (**B**; PDB ID 6YA5). The active site pocket containing two metal ions (purple-blue spheres) is presented as a grey surface. Interacting residues and ligands are in stick representation. Hydrogen bonds are shown as black dashes. Coordinating water molecules (W1, W2 and W3) are presented as red spheres.

To reveal the structure of orientin bound to wild-type PA-Nter and the I38T mutant, we prepared both proteins for X-ray crystallographic studies. Thanks to its high inhibitory potency and aqueous solubility, orientin (PDBe ligand USE) was soaked into the unoccupied protein crystals. The structure of wild-type PA-Nter in complex with orientin was refined to 1.9 Å resolution (PDB ID 7NUG), and the I38T mutant complex (PDB ID 7NUH) was refined to 2.2 Å resolution. Both crystallographic models consisted of one protein molecule per asymmetric unit. Two metal ions were embedded in the endonuclease active site. Based on the strong anomalous signal observed, we speculate a mixed occupancy of Mn^2+^ and Mg^2+^ cations. The majority (0.8 occupancy) proximal ion was Mn^2+^, coordinated by four protein atoms (N^ε2^His-41, O^δ2^Asp-108, O^ε2^Glu-119, O Ile-120) and two hydroxyl groups from the 3′,4′-dihydroxyphenyl moiety of orientin. The distal octahedrally coordinated sphere was partially assigned as the central Mg^2+^ cation (0.4 occupancy), which corresponds to lower anomalous scattering and is in agreement with previously reported PA-Nter complexes (Figure 3) [19,37].

**Figure 4:**
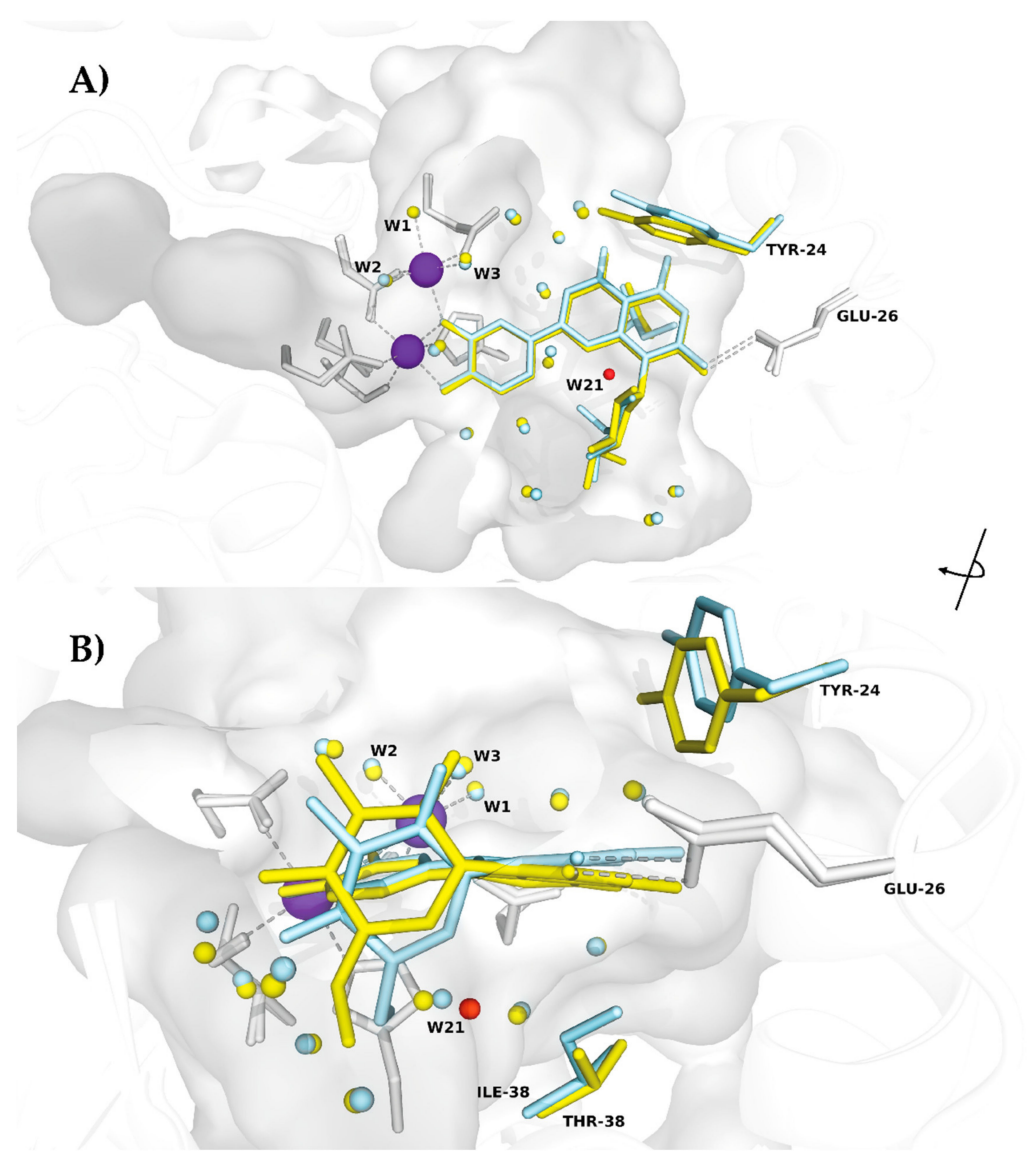
Structural alignment of wild-type PA-Nter (pale cyan) and its I38T mutant (yellow), both in complex with orientin (PDB ID 7NUG, 7NUH). The active site (**A**) containing two metal ions (purple-blue spheres) is presented as a surface cavity in grey. Closeup view, 90° clockwise rotated (**B**), onto interacting residues, Ile-38/Thr-38, and ligands (all in stick representation) reveals shift of the glucoside moiety. The hydrogen bond between ligand and the Glu-26 side chain is shown as a grey dash. Common waters contributing to protein-ligand binding, with a maximum distance of 3 Å from the ligand, are presented as spheres with color coding corresponding to each structure. Metal-coordinating water molecules (W1, W2 and W3) and W21 (red) are shown as spheres.

The distal ion was coordinated by O^ε2^Glu-80, O^δ2^Asp-108, the 4′-hydroxyl group of orientin, and three water molecules (W1, W2, W3). Unoccupied PA-Nter (PDB entry 5DES, not shown) harbors metal ions coordinated by two additional water molecules, which are replaced by the two hydroxyl groups from the flavonoid’s B ring in our wild-type and I38T variant structures. Orientin adopts a similar position as luteolin in a previously described PA-Nter structure (PDB ID 6YA5) [37]. Ligands in both structures form a hydrogen bond with O^ε2^Glu-26 at the C-7 position. The previously observed high affinity of luteolin for PA-Nter is likely to be enhanced by the additional hydrogen bonding network surrounding orientin’s glucosyl moiety. This was observed in both protein variants, as most of the water molecules in the first solvation shell are located at similar positions (Figure 4). However, there is a little ligand shift visible in the mutant variant. Wild-type Ile-38 moves orientin towards the solvent and differs from the mutant variant with an RMSD of 0.466 Å. Moreover, there is a visible movement of the Tyr-24 side chain in the mutant variant, likely to be due to the ligand shift.

**Figure 5:**
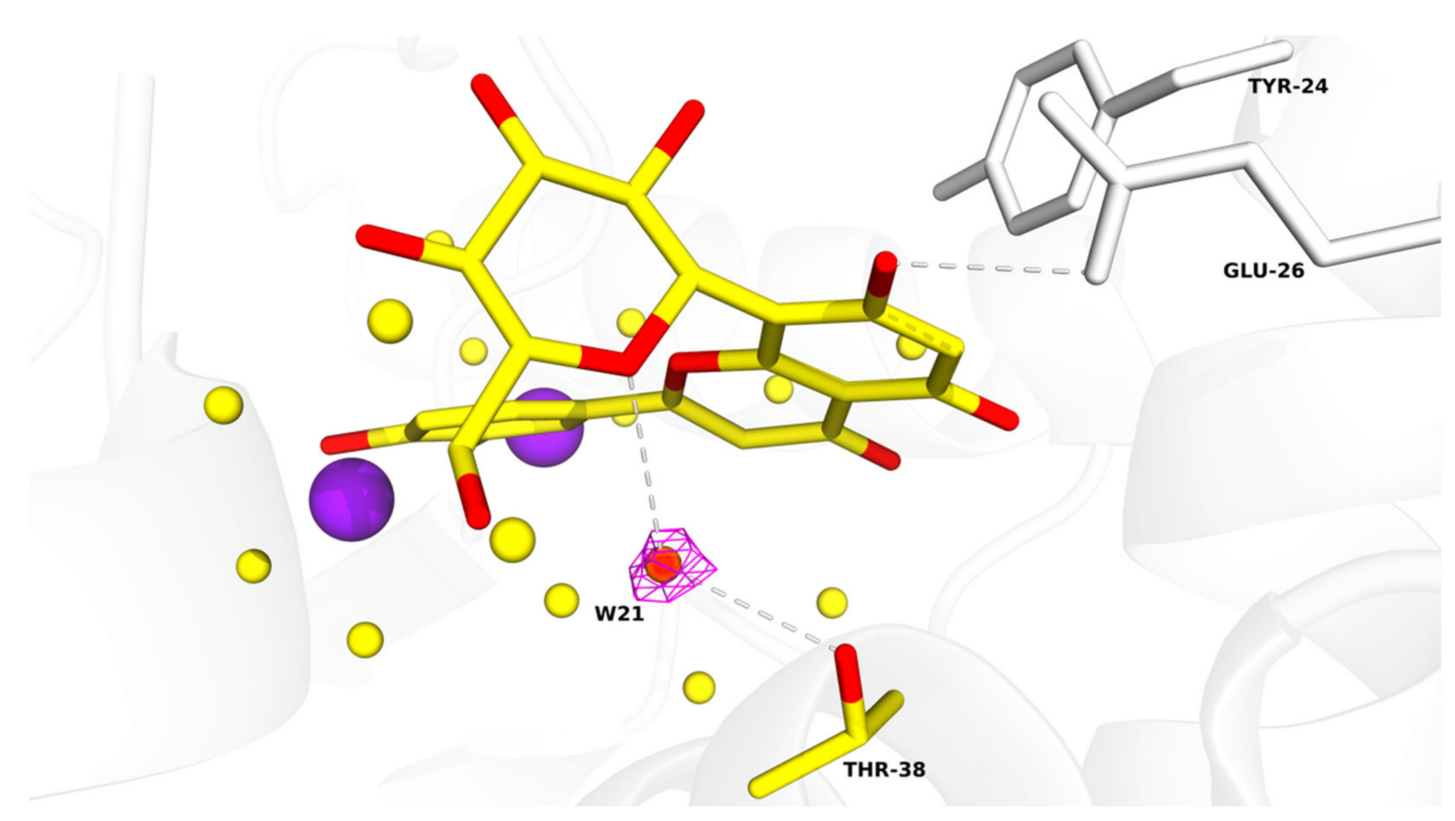
Closeup view of the active site pocket of I38T PA-Nter. Metal ions are shown as purple-blue spheres. Ligand and amino acid residues are shown in stick representation, where red represents oxygen atoms. The hydrogen bonds between the ligand and the Glu-26 side chain and between the ligand, W21, and the Thr-38 side chain are depicted as grey dashes. Water molecules are shown as yellow spheres, except for W21 which is shown as a red sphere with its 2Fo–Fc electron density map in magenta mesh contoured at 1.5 σ.

The side chain of Tyr-24 in the mutant variant approaches the active site pocket, whereas in the wild-type, Tyr-24 is pushed away from the cavity (RMSD of 0.029 Å for side chain atoms). Thr-38, which is one atom shorter than Ile-38, helps accommodate orientin. O^γ1^Thr-38 forms a hydrogen bond through W21 to the glucosyl moiety of the ligand (O6) (Figure 5). This is not observed in the wild-type, as Ile-38 does not contain a hydroxyl group capable of such interaction.

## 3. Materials and Methods

### 3.1. AlphaScreen Assay

AlphaScreen experiments were performed using a Perkin Elmer Enspire plate reader in 96-well ProxiPlates. Biotinylated L-742.001 derivative [37] was captured on Streptavidin-coated donor beads (Perkin Elmer). Separately, GST-PA-Nter fusion protein was bound to GSH-coated acceptor beads (Perkin Elmer). These solutions were incubated for 60 min at room temperature in the dark and subsequently mixed and incubated for an additional 120 min. In experiments screening for endonuclease inhibitors, compounds were mixed with both types of beads prior to the 120-min incubation. The optimal concentrations of biotinylated L-742.001 derivative and GST-PA-Nter were 15 nM and 50 nM, respectively. The concentrations of donor and acceptor beads were 5 µg/mL each in a 50 µL reaction volume. All experiments were performed in 25 mM Tris-HCl, pH 7.4, 150 mM NaCl, 0.05% Tween20, 1 mM MnCl_2_, 10 mM MgCl_2_, and 1 mM 2-mercaptoethanol.

### 3.2. Cloning, Expression and Purification of Recombinant Proteins

DNA encoding the first 196 amino acids of the N-terminal domain of the influenza polymerase acidic subunit (PA-Nter) from the viral strain A/California/07/2009 (H1N1) (GenBank accession No. CY121685.1) was prepared using GenScript USA Inx. The flexible loop (residues 51–72) was replaced with a GGS linker [52]. Constructs with affinity tags (GST-PA-Nter and His_6_-SUMO-Nter) were prepared for both wild-type and I 38T PA-Nter. First, DNA encoding PA-Nter was inserted into the plasmid pGEX-1λT. Next, PA-Nter with a (GS)_4_ linker and N-terminal extension was cloned into the plasmid pETM11-SUMO3 (EMBL, Heidelberg, Germany) using BamHI and XhoI sites. The I38T mutation was introduced into both GST- and His_6_-SUMO-tagged constructs using the following primers for site-directed mutagenesis: 5′–CAAGTTTGCTGCAACATGCACACAT TTG–3′ and 5′–CAAATGTGTGCATGTTGCACAAACTTG–3′. All tagged PA-Nter constructs were expressed in *E. coli* BL21 (DE3) RIL. Cells were harvested and resuspended in lysis buffer (25 mM Tris/HCl, pH 7.5, 150 mM NaCl, 1 mM EDTA (GST) or 50 mM Tris/HCl, pH 8.0, 200 mM NaCl, 10 mM imidazole (His_6_-SUMO)) and lysed with an EmulsiFlex device (Avestin, Ottawa, ON, Canada) at a pressure of 1200 bar. GST-tagged PA-Nter soluble proteins were loaded onto a glutathione-agarose column (ThermoFisher Scientific) and eluted with elution buffer (50 mM Tris/HCl, pH 7.5, 150 mM NaCl, 10 mM reduced L glutathione, 1 mM EDTA). Analogously, His_6_-SUMO PA-Nter was purified using Ni-NTA Agarose (Roche Diagnostics GmbH, Mannheim, Germany) and eluted with Ni-NTA elution buffer (50 mM Tris/HCl, pH 8.0, 200 mM NaCl, 250 mM imidazole). The His_6_-SUMO tag was removed by the ULP1 protease. All proteins were purified on a Superdex 75 (GE Healthcare/Amersham Pharmacia, Uppsala, Sweden) gel filtration chromatography column, yielding >95% purity as estimated by SDS-PAGE.

### 3.3. Gel-Based Endonuclease Inhibitory Assay

To compare the endonuclease activities of wild-type and I38T PA-Nter in the presence of selected compounds (baloxavir acid, luteolin, orientin), we used a gel-based endonuclease inhibitory assay. The single-stranded DNA substrate M13mp18 (New England Biolabs) was cleaved in vitro by either protein. Each reaction (10 µL) contained 1 μM protein (GST-PA-Nter wild type/GST-PA-Nter I38T mutant) in digestion buffer (25 mM Tris-HCl, pH 7.4, 150 mM NaCl, 0.05% Tween20, 1 mM MnCl_2_, 10 mM MgCl_2_, 1 mM 2-mercaptoethanol) and was incubated with various concentrations of inhibitors (BXA/LU2/OTN/**21**/**30**). Reactions were initiated by the addition of 0.2 μg of M13mp18 plasmid. Reactions were incubated at 37 °C for 5 h and stopped by adding 1 μL of 0.2 M EDTA. Finally, cleavage of DNA substrate was visualized by agarose electrophoresis using 1% agarose gel stained with GelRed.

### 3.4. Crystallization and Diffraction Data Collection

Hexagonal bifrustum crystals of empty wild-type and I38T PA-Nter subunits were obtained by the hanging-drop vapor diffusion method. Protein solution (12 mg/mL) was mixed with crystallization reservoir solution (12.5% *w*/*v* PEG 1000, 12.5% *w*/*v* PEG 3350, 0.1% M MOPS/HEPES-Na pH 7.5, 0.06 M magnesium chloride, 0.06 M calcium chloride) and PA-Nter seed in a 1:1:0.2 ratio. Crystals grew at 18 °C until they reached approximately 0.2 mm in diameter. Ligands (100 mM solution in DMSO) were soaked in for 15 min (the final DMSO concentration did not exceed 5%). Crystals were harvested, flash-cooled by plunging into liquid nitrogen and stored at −196 °C.

Diffraction qualities were tested at BESSY II and data were collected at −173 °C on a home diffractometer (MicroMax-007 HF microfocus equipped with a PILATUS 300 K detector, Rigaku). The crystal of wild-type PA-Nter soaked with orientin diffracted to a resolution of up to 1.87 Å, and the I38T PA-Nter/orientin crystal diffracted up to 2.15 Å. Diffraction data were processed, integrated, and reduced using XDS [53] and scaled using XSCALE from the XDS suite [54]. Both crystals belonged to the ***P***6_4_22 space group and contained one molecule per asymmetric unit, with a solvent content of 47.5% (wild-type PA-Nter), and 48.2% (I38T PA-Nter). We observed anomalous signals up to a resolution of 3.0 Å for wild-type and 3.5 Å for the I38T variant. Therefore, the data were processed with unmerged Friedel pairs. Detailed crystal parameters and data collection statistics are given in Table S1.

### 3.5. Structure Determination and Analyses

Structures of wild-type and I38T PA-Nter were determined by molecular replacement with MOLREP [55] from the CCP4 package [56] using a previously reported structure of PA-Nter as a template (PDB entry 6YA5 [37]). The final step of complex structure polishing was carried out by cycles of manual adjustments using Coot software [57] followed by refinement in REFMAC 5.8.0103 [58]. MolProbity [59] was used to validate the quality of the final models. Refinement statistics are given in Table S1. All figures illustrating structural representations were prepared with PyMOL (The PyMOL Molecular Graphics System, Version 2.4.2 accessed on 10 March 2020; Schrödinger, LLC., New York, NY, USA). Atomic coordinates and experimental structure factors are deposited in the Protein Data Bank under codes 7NUG for wild-type PA-Nter in complex with orientin and 7NUH for I38T PA-Nter in complex with orientin.

### 3.6. Chemistry

Unless otherwise noted, all reactions were carried out under argon in oven-dried glassware. Solvents were distilled from drying agents as indicated and transferred under nitrogen: THF (Na/benzophenone), toluene (Na/benzophenone), MeCN (CaH_2_), and DCM (CaH_2_). Chromatography was performed using a Teledyne ISCO Combi Flash Rf+ flash chromatography system with RediSep Rf Gold Silica or RediSep Rf Gold Reversed-phase C18 columns. All starting materials were used as purchased (Sigma Aldrich, Alfa Aesar, TCI, Fluorochem, Combi-Blocks), unless otherwise indicated. Compounds luteolin and orientin were purchased from Sigma-Aldrich (product numbers L9283 and O9765). All inhibitors were purified using an ECOM TOY18DAD800 compact preparative system [flow rate 15 mL/min; gradient 0–60% MeCN/H_2_O (0.1% trifluoroacetic acid) over 60 min], with a ProntoSIL 120-10-C18 ace-EPS column, 10 μm, 20 × 250 mm. The purity of compounds and composition of the reaction mixtures were tested on a Waters UPLC-MS Acquity with QDa Mass Detector (flow rate 0.5 mL/min, gradient 0–100 % MeCN/H_2_O (0.1% formic acid) over 7 min) with an ACQUITY UPLC BEH C18 Column, 130 Å, 1.7 µm, 2.1 mm × 100 mm with a 2.1 mm × 5 mm pre-column. The final inhibitors were of at least 90% purity. ^1^H-NMR spectra were recorded on Bruker instruments at 400, 500 or 600 MHz; ^13^C-NMR spectra were recorded at 100, 126 or 150 MHz. Chemical shifts are provided in *δ*-scale; coupling constants *J* are given in Hz. ESI high resolution mass spectra were recorded using a Thermo Scientific LTQ Orbitrap XL (Termo Fisher Scientific, Waltham, Massachusetts, USA) controlled by MassLynx software.

Compounds **1** and **2** were prepared according to literature procedures [43]. Analytical data for these compounds were in agreement with published data.

### 3.7. 4-(5,7-Diacetoxy-4-oxo-4H-chromen-2-yl)-1,2-phenylene Diacetate (***1***)

Neat acetic anhydride (10 mL) was added to luteolin (1.43 g, 5.0 mmol), followed by the addition of pyridine (1.0 mL). The reaction mixture was heated to 145 °C for 3 h. The hot mixture was poured into ice and stirred for 30 min. The suspension was filtered off, and the solids were mixed with a boiling mixture of MeOH/CHCl_3_ (9:1, 15 mL). The product was isolated by filtration from the cool suspension to furnish the desired compound **1** (1.75 g, 77%). ^1^H NMR (400 MHz, CDCl_3_) *δ =* 7.74 (dd, *J* = 8.4, 2.2 Hz, 1H), 7.70 (d, *J* = 2.1 Hz, 1H), 7.38–7.32 (m, 2H), 6.85 (d, *J* = 2.2 Hz, 1H), 6.61 (s, 1H), 2.44 (s, 3H), 2.35 (s, 6H), 2.33 (s, 3H) ppm. ^13^C NMR (101 MHz, CDCl_3_) *δ =* 176.3, 169.5, 168.1, 167.9, 160.9, 157.7, 154.2, 150.4, 145.0, 142.8, 129.8, 124.6, 124.5, 121.8, 115.0, 114.0, 109.2, 109.1, 21.3, 21.2, 20.8, 20.8 ppm.

### 3.8. 4-(5-Acetoxy-7-hydroxy-4-oxo-4H-chromen-2-yl)-1,2-phenylene Diacetate (***2***)

4-(5,7-Diacetoxy-4-oxo-4*H*-chromen-2-yl)-1,2-phenylene diacetate (**1**) (1.70 g, 3.7 mmol, 1.00 eq.) was dissolved in THF (56 mL) and NMP (19 mL). Then, imidazole (88.5 mg, 1.3 mmol, 0.35 eq.) was added at 0 °C followed by addition of thiophenol (0.45 mL, 4.4 mmol, 1.18 eq.) via septum under a nitrogen atmosphere at 0 °C. The reaction mixture was stirred and slowly warmed to room temperature over 2 h until the starting material was fully consumed (TLC, UPLC-MS). Volatiles were evaporated and the oily residue was dissolved in EtOAc (150 mL). The organic phase was washed with 5% HCl (aq., 5 × 45 mL), and organic solvents were evaporated. The solids were mixed with EtOH, and the product was isolated by filtration to furnish the desired compound **2** (1.05 g, 68%). ^1^H NMR (400 MHz, DMSO*-d*_6_) *δ =* 11.17 (s, 1H), 8.08–7.95 (m, 2H), 7.49 (d, *J* = 9.0 Hz, 1H), 6.97 (d, *J* = 2.3 Hz, 1H), 6.80 (s, 1H), 6.60 (d, *J* = 2.3 Hz, 1H), 2.35 (s, 3H), 2.34 (s, 3H), 2.32 (s, 3H) ppm. ^13^C NMR (101 MHz, DMSO*-d*_6_) *δ =* 175.2, 168.9, 168.2, 168.0, 162.3, 159.4, 158.2, 150.1, 144.6, 142.5, 129.5, 124.8, 124.5, 121.7, 109.5, 108.8, 108.0, 101.0, 21.0, 20.4 (2C) ppm.

### 3.9. 4-(5-Acetoxy-4-oxo-7-(((trifluoromethyl)sulfonyl)oxy)-4H-chromen-2-yl)-1,2-phenylene Diacetate (***3***)

A flask with 4-(5-acetoxy-7-hydroxy-4-oxo-4*H*-chromen-2-yl)-1,2-phenylene diacetate (**2**) (1.00 g, 2.4 mmol, 1.0 eq.) was purged with nitrogen. The solids were mixed with dry CH_2_Cl_2_ (12 mL) and the suspension was cooled to 0 °C. Pyridine (0.39 mL, 4.9 mmol, 2.0 eq.) was added dropwise. After 10 min, trifluoromethanesulfonic anhydride (0.53 mL, 3.2 mmol, 1.3 eq.) was added dropwise at 0 °C under a nitrogen atmosphere. The resulting red-colored reaction mixture was stirred for 3.5 h until the starting material was fully consumed (TLC, UPLC-MS). Then, the reaction mixture was diluted with additional CH_2_Cl_2_ (15 mL) and washed with sat. NH_4_Cl (aq., 1 × 10 mL), sat. CuSO_4_ (aq., 2 × 10 mL) and water (1 × 10 mL). The organic phase was dried over anhydrous MgSO_4_ and then evaporated under reduced pressure. The residue was purified by flash chromatography (SiO_2_, cyclohexane/EtOAc = 100:0 → 50:50) to afford the desired triflate **3** (533 mg, 41%). ^1^H NMR (400 MHz, CDCl_3_) *δ =* 7.74 (dd, *J* = 8.5, 2.3 Hz, 1H), 7.71 (d, *J* = 2.1 Hz, 1H), 7.44 (d, *J* = 2.4 Hz, 1H), 7.38 (d, *J* = 8.4 Hz, 1H), 6.99 (d, *J* = 2.4 Hz, 1H), 6.65 (s, 1H), 2.45 (s, 3H), 2.35 (s, 3H), 2.33 (s, 3H) ppm. ^13^C NMR (101 MHz, CDCl_3_) *δ =* 175.6, 169.1, 168.1, 167.8, 161.3, 157.6, 151.6, 151.3, 145.3, 142.9, 129.2, 124.7, 124.6, 121.9, 120.4, 117.1, 113.6, 109.5, 109.4, 21.1, 20.8, 20.7 ppm. ^19^F NMR (376 MHz, CDCl_3_) *δ =* −72.4 ppm. HRMS (ESI) *m/z* calcd for C_22_H_16_F_3_O_11_S [M+H^+^]^+^ 545.0359, found 545.0356.

### 3.10. 4-(5-Acetoxy-7-(diethoxyphosphoryl)-4-oxo-4H-chromen-2-yl)-1,2-phenylene Diacetate (***4***)

A tube with 4-(5-acetoxy-4-oxo-7-(((trifluoromethyl)sulfonyl)oxy)-4*H*-chromen-2-yl)-1,2-phenylene diacetate (**3**) (100 mg, 0.184 mmol, 1.0 eq.) and Pd(PPh_3_)_4_ (64 mg, 0.055 mmol, 0.3 eq.) was sealed, and the mixture was dissolved in anhydrous MeCN (1.8 mL) followed by an addition of diethylphosphite (30 μL, 0.221 mmol, 1.2 eq.) and DIPEA (42 μL, 0.240 mmol, 1.3 eq.) via septum. The mixture was degassed with a stream of argon for 15 min followed by heating to 70 °C for 3 h until the starting material was fully consumed (TLC, UPLC-MS). The reaction mixture was cooled to room temperature, filtered through Celite, and washed with EtOAc. Solvents were evaporated and the resulting mixture was purified by flash chromatography (SiO_2_, cyclohexane/EtOAc = 100:0 → 0:100) to afford the desired phosphonate **4** (76 mg, 78%). ^1^H NMR (400 MHz, CDCl_3_) *δ =* 7.93 (dd, *J* = 14.9, 1.3 Hz, 1H), 7.76–7.70 (m, 2H), 7.39–7.31 (m, 2H), 6.65 (s, 1H), 4.27–4.04 (m, 4H), 2.42 (s, 3H), 2.32 (s, 3H), 2.30 (s, 3H), 1.34 (td, *J* = 7.0, 0.6 Hz, 6H) ppm. ^13^C NMR (101 MHz, CDCl_3_) *δ =* 176.3, 169.5, 168.0, 167.8, 161.2, 156.8 (d, *J* = 23.5 Hz), 149.6, 149.4, 145.1, 142.8, 135.9, 134.0, 129.4, 124.6, 124.5, 121.8, 121.5 (d, *J* = 9.5 Hz), 120.3 (d, *J* = 10.3 Hz), 119.4 (d, *J* = 2.6 Hz), 109.2, 63.1, 63.0, 21.1, 20.7, 20.6, 16.4, 16.4 ppm. ^31^P NMR (162 MHz, CDCl_3_) *δ =* 16.58 ppm. HRMS (ESI) *m/z* calcd for C_25_H_25_O_11_PNa [M+Na^+^]^+^ 555.1026, found 555.1023.

### 3.11. (2-(3,4-Dihydroxyphenyl)-5-hydroxy-4-oxo-4H-chromen-7-yl)phosphonic Acid (***5***)

4-(5-Acetoxy-7-(diethoxyphosphoryl)-4-oxo-4*H*-chromen-2-yl)-1,2-phenylene diacetate (**4**) (312 mg, 0.58 mmol, 1.0 eq.) was dissolved in dry CH_2_Cl_2_ (18 mL). Then, bromo-trimethyl-silane (0.77 mL, 5.86 mmol, 10 eq.) was added dropwise under a nitrogen atmosphere. The reaction mixture was stirred for 31 h until the starting material was fully consumed (TLC, UPLC-MS). The solvent was evaporated and MeOH (3.1 mL) was added. The white suspension was allowed to stir for 1 h followed by evaporation of the solvent. THF (5.8 mL) was added, followed by an addition of 1 M KOH in MeOH (2.9 mL, 2.9 mmol, 5.0 eq). The reaction mixture was allowed to stir for 1 h. Solvent was evaporated, and the resulting mixture was purified by flash chromatography (SiO_2_-C_18_, H_2_O (0.1% TFA)/MeCN= 100:0 → 30:70) to afford the desired final phosphonic acid **5** (51 mg, 25%). ^1^H NMR (400 MHz, DMSO*-d*_6_) *δ =* 12.83 (s, 1H), 7.51 (dd, *J* = 8.3, 2.3 Hz, 1H), 7.48 (d, *J* = 2.3 Hz, 1H), 7.34 (dd, *J* = 14.0, 1.2 Hz, 1H), 6.97 (dd, *J* = 13.7, 1.2 Hz, 1H), 6.91 (d, *J* = 8.3 Hz, 1H), 6.87 (s, 1H) ppm. ^13^C NMR (101 MHz, DMSO*-d*_6_) *δ =* 183.1, 165.7, 159.9 (d, *J* = 19.7 Hz), 155.8, 155.6, 150.7, 146.3, 143.6, 141.9, 121.6, 120.0, 116.5, 114.2, 112.5 (d, *J* = 9.9 Hz), 111.5 (d, *J* = 2.6 Hz), 109.6 (d, *J* = 9.9 Hz), 104.2 ppm. ^31^P NMR (162 MHz, DMSO) *δ =* 11.98 ppm. HRMS (ESI) *m/z* calcd for C_15_H_10_O_8_P [M−H^+^]^–^ 349.0118, found 349.0118.

### 3.12. Palladium-Catalyzed Methoxycarbonylation of Triflate ***3***

A tube with 4-(5-acetoxy-4-oxo-7-(((trifluoromethyl)sulfonyl)oxy)-4*H*-chromen-2-yl)-1,2-phenylene diacetate (**3**) (100 mg, 0.184 mmol, 1.0 eq.), 1,3-bis(diphenylphosphino)propane (7.50 mg, 0.018 mmol, 0.1 eq.), Mo(CO)_6_ (24 mg, 0.092 mmol, 0.5 eq.) and palladium acetate (4.00 mg, 0.018 mmol, 0.1 eq.) was sealed, and dry DMSO (1.1 mL) and dry MeOH (0.7 mL) were added via septum. The mixture was degassed with a stream of argon for 15 min followed by addition of triethylamine (57 μL, 0.405 mmol, 2.2 eq.). The reaction mixture was heated to 70 °C for 20 h until the starting material was fully consumed (UPLC-MS). The reaction mixture was cooled to room temperature, diluted with MeOH (5 mL), and filtered through a syringe filter. The solvents were evaporated, and the residue was purified by flash chromatography (SiO_2_-C_18_, H_2_O (0.1% TFA)/MeCN = 100:0 → 20:80) to afford the desired monoacetylated ester **6** (28 mg, 40%) and deacetylated ester **7** (29 mg, 48%).

### 3.13. Methyl 5-acetoxy-2-(3,4-dihydroxyphenyl)-4-oxo-4H-chromene-7-carboxylate (***6***)

^1^H NMR (401 MHz, DMSO-*d*_6_) δ 9.98 (s, 1H), 9.37 (s, 1H), 8.12 (t, *J* = 1.5 Hz, 1H), 7.56 – 7.52 (m, 1H), 7.50 – 7.43 (m, 2H), 6.90 (d, *J* = 8.2 Hz, 1H), 6.69 (s, 1H), 3.92 (s, 3H), 2.33 (s, 3H) ppm. ^13^C NMR (101 MHz, DMSO-*d*_6_) δ 175.2, 169.0, 164.2, 162.9, 156.4, 149.9, 148.8, 145.8, 133.9, 121.1, 119.3, 119.2, 118.8, 117.4, 116.0, 113.6, 106.2, 53.0, 20.9 ppm. HRMS (ESI) *m/z* calcd for C_19_H_14_O_8_Na [M+Na^+^]^+^ 393.0581, found 393.0580.

### 3.14. Methyl 2-(3,4-dihydroxyphenyl)-5-hydroxy-4-oxo-4H-chromene-7-carboxylate (***7***)

^1^H NMR (401 MHz, DMSO-*d*_6_) δ 12.91 (s, 1H), 10.11 (s, 1H), 9.42 (s, 1H), 7.64 (d, *J* = 1.6 Hz, 1H), 7.55 (dd, *J* = 8.4, 2.3 Hz, 1H), 7.51 (d, *J* = 2.3 Hz, 1H), 7.22 (d, *J* = 1.6 Hz, 1H), 6.93–6.91 (m, 2H), 3.91 (s, 3H) ppm. ^13^C NMR (101 MHz, DMSO-*d*_6_) δ 182.9, 166.5, 165.3, 160.4, 156.0, 150.9, 146.3, 135.8, 121.4, 120.2, 116.5, 114.3, 113.0, 111.1, 108.7, 104.3, 53.3 ppm. HRMS (ESI) *m/z* calcd for C_17_H_12_O_7_ [M+H^+^]^+^ 329.0656, found 329.0655.

### 3.15. 2-(3,4-Dihydroxyphenyl)-5-hydroxy-4-oxo-4H-chromene-7-carboxylic acid (***8***)

A solution of methyl 2-(3,4-dihydroxyphenyl)-5-hydroxy-4-oxo-4*H*-chromene-7-carboxylate (**7**) (136 mg, 0.41 mmol, 1.0 eq.) in THF (8.4 mL) was heated to 45 °C under a nitrogen atmosphere, followed by addition of 2 M aq. LiOH (4.2 mL, 8.3 mmol, 20 eq.) via septum. The reaction mixture was stirred for 1 h until the starting material was fully consumed (UPLC-MS). The solvents were evaporated and the residue was purified by flash chromatography (SiO_2_-C_18_, H_2_O (0.1% TFA)/MeCN= 100:0 → 20:80) to afford the desired final carboxylic acid **8** (93 mg, 71 %). ^1^H NMR (400 MHz, DMSO*-d*_6_) *δ =* 12.87 (s, 1H), 10.09 (s, 1H), 9.43 (s, 1H), 7.61 (d, *J* = 1.3 Hz, 1H), 7.53 (dd, *J* = 8.4, 2.3 Hz, 1H), 7.49 (d, *J* = 2.3 Hz, 1H), 7.21 (d, *J* = 1.3 Hz, 1H), 6.91 (d, *J* = 9.0 Hz, 2H) ppm. ^13^C NMR (101 MHz, DMSO*-d*_6_) *δ =* 182.6, 165.8, 165.6, 159.9, 155.5, 150.4, 145.9, 136.9, 121.1, 119.7, 116.1, 113.8, 112.3, 110.8, 108.2, 103.8 ppm. HRMS (ESI) *m/z* calcd for C_16_H_10_O_7_ [M−H^+^]^–^ 313.0354, found 313.0351.

### 3.16. 2-(3,4-Dihydroxyphenyl)-5-hydroxy-4-oxo-4H-chromene-7-carboxamide (***9***)

A tube with a mixture of methyl 5-acetoxy-2-(3,4-dihydroxyphenyl)-4-oxo-4*H*-chromene-7-carboxylate (**6**) (21.5 mg, 0.058 mmol, 1.0 eq.) and methyl 2-(3,4-dihydroxyphenyl)-5-hydroxy-4-oxo-4*H*-chromene-7-carboxylate (**7**) (22 mg, 0.067 mmol, 1.15 eq.) was sealed, and 7 M ammonia in MeOH (10 mL) was added via septum. The reaction mixture was heated to 50 °C and stirred for 4 days until the starting materials were fully consumed (UPLC-MS). The solvents were evaporated and the residue was purified by preparative HPLC to obtain the final amide **9** (8.0 mg, 21%). ^1^H NMR (600 MHz, DMSO*-d*_6_) *δ =* 12.82 (s, 1H), 10.07 (s, 1H), 9.45 (d, *J* = 2.5 Hz, 1H), 8.20 (s, 1H), 7.68 (s, 1H), 7.60 (d, *J* = 1.5 Hz, 1H), 7.51 (dd, *J* = 8.4, 2.3 Hz, 1H), 7.47 (d*, J* = 2.3 Hz, 1H), 7.25 (d, *J* = 1.3 Hz, 1H), 6.92 (d, *J* = 8.5 Hz, 1H), 6.89 (s, 1H) ppm. ^13^C NMR (151 MHz, DMSO*-d*_6_) *δ =* 182.6, 166.1, 165.5, 159.8, 155.5, 150.3, 145.9, 140.7, 121.2, 119.5, 116.1, 113.7, 111.3, 109.6, 106.6, 103.8 ppm. HRMS (ESI) *m/z* calcd for C_16_H_11_NO_6_Na [M+Na^+^]^+^ 336.0479, found 336.0479.

### 3.17. 2-(3,4-Dihydroxyphenyl)-5-hydroxy-N-(2-hydroxyethyl)-4-oxo-4H-chromene-7-carboxamide (***10***)

Methyl 2-(3,4-dihydroxyphenyl)-5-hydroxy-4-oxo-4*H*-chromene-7-carboxylate (**7**) (70 mg, 0.213 mmol, 1.0 eq.) was mixed with dry EtOH (2 mL). Potassium carbonate (100 mg, 0.723 mmol, 3.4 eq.) was added, followed by 2-aminoethanol (0.6 mL). The reaction mixture was heated to 50 °C and stirred for 48 h until the starting materials was fully consumed (UPLC-MS). The solvents were evaporated and the residue was purified by preparative HPLC to obtain the final amide **10** (17 mg, 23%). ^1^H NMR (400 MHz, DMSO*-d*_6_) *δ =* 12.81 (s, 1H), 10.07 (s, 1H), 9.45 (s, 1H), 8.68 (t, *J* = 5.6 Hz, 1H), 7.59 (d, *J* = 1.5 Hz, 1H), 7.51 (dd, *J* = 8.3, 2.3 Hz, 1H), 7.48 (d, *J* = 2.3 Hz, 1H), 7.23 (d, *J* = 1.5 Hz, 1H), 6.92 (d, *J* = 8.4 Hz, 1H), 6.89 (s, 1H), 3.53 (t, *J* = 6.1 Hz, 2H), 3.35 (q, *J* = 5.2, 4.4 Hz, 2H) ppm. ^13^C NMR (101 MHz, DMSO*-d*_6_) *δ =* 182.6, 165.4, 164.6, 159.8, 155.5, 150.3, 145.9, 140.9, 121.2, 119.5, 116.1, 113.7, 111.2, 109.3, 106.4, 103.7, 59.5, 42.4 ppm. HRMS (ESI) *m/z* calcd for C_18_H_15_NO_7_Na [M+Na^+^]^+^ 380.0741, found 380.0742.

### 3.18. 7-Amino-2-(3,4-dihydroxyphenyl)-5-hydroxy-4H-chromen-4-one (***11***)

2-(3,4-Dihydroxyphenyl)-5-hydroxy-4-oxo-4*H*-chromene-7-carboxylic acid (**8**) (32 mg, 0.102 mmol, 1.0 eq.) was dissolved in dry DMF (1 mL) followed by dropwise addition of triethylamine (21 µL, 0.143 mmol, 1.4 eq.) and diphenylphosphoryl azide (31 µL, 0.143 mmol, 1.4 eq.) under a nitrogen atmosphere. The reaction mixture was heated to 100 °C and stirred for 16 h. Water (1 mL) was added and the reaction mixture was stirred for 3 h at 100 °C. The solvents were evaporated and the residue was purified by preparative HPLC to obtain the final amine **11** (8.7 mg, 30%). ^1^H NMR (500 MHz, DMSO*-d*_6_) *δ =* 12.93 (s, 1H), 9.83 (s, 1H), 9.39 (s, 1H), *δ =* 7.36–7.33 (m, 2H), 6.87 (d, *J* = 8.1 Hz, 1H), 6.51 (s, 1H), 6.12 (d, *J* = 1.8 Hz, 1H), 5.92 (d, *J* = 2.0 Hz, 1H) ppm. ^13^C NMR (126 MHz, DMSO*-d*_6_) *δ =* 181.1, 163.2, 161.6, 158.1, 156.3, 149.7, 146.1, 122.3, 119.0, 116.5, 113.6, 103.0, 101.5, 96.3, 90.7 ppm. HRMS (ESI) *m/z* calcd for C_15_H_12_NO_5_ [M+H^+^]^+^ 286.0710, found 286.0710.

### 3.19. N-(2-(3,4-Dihydroxyphenyl)-5-hydroxy-4-oxo-4H-chromen-7-yl)acetamide (***12***)

7-Amino-2-(3,4-dihydroxyphenyl)-5-hydroxy-4*H*-chromen-4-one **(11)** (30 mg, 0.10 mmol, 1.0 eq.) was dissolved in dry DMF (0.5 mL), followed by addition of DIPEA (37 µL, 0.21 mmol, 2.0 eq.) and *N*-succinimidyl acetate (33 mg, 0.15 mmol, 1.5 eq.). The reaction mixture was allowed to stir at room temperature for 36 h until the starting material was fully consumed (UPLC-MS). The reaction mixture was directly purified by preparative HPLC to obtain the final product **12** (7.9 mg, 23%). ^1^H NMR (400 MHz, DMSO*-d*_6_) *δ =* 10.57 (s, 1H), 7.78 (dd, *J* = 8.6, 2.3 Hz, 1H), 7.73 (d, *J* = 2.2 Hz, 1H), 7.06 (d, *J* = 8.6 Hz, 1H), 6.67 (s, 1H), 6.17 (d, *J* = 1.8 Hz, 1H), 5.94 (d, *J* = 2.0 Hz, 1H), 2.30 (s, 3H) ppm. ^13^C NMR (101 MHz, DMSO*-d*_6_) *δ =* 180.6, 168.7, 161.7, 161.1, 157.6, 155.9, 152.5, 138.7, 125.2, 122.0, 121.5, 117.3, 103.2, 101.1, 95.9, 90.4, 20.6 ppm. HRMS (ESI) *m/z* calcd for C_17_H_12_NO_6_ [M−H^+^]^–^ 326.0670, found 326.0671.

### 3.20. 4-(5-Acetoxy-7-cyano-4-oxo-4H-chromen-2-yl)-1,2-phenylene Diacetate (***13***)

A tube with 4-(5-acetoxy-4-oxo-7-(((trifluoromethyl)sulfonyl)oxy)-4*H*-chromen-2-yl)-1,2-phenylene diacetate (**3**) (100 mg, 0.184 mmol, 1.00 eq.), zinc cyanide (12.0 mg, 0.101 mmol, 0.55 eq.), and XantPhos Pd G2 (2.00 mg, 0.002 mmol, 0.01 eq.) was sealed and purged with nitrogen. Dry DMF (0.9 mL) and DIPEA (15 μL, 0.084 mmol, 0.15 eq.) were added. The reaction mixture was heated to 85 °C and stirred for 16 h until the starting material was fully consumed (TLC, UPLC-MS). Then the reaction mixture was cooled to room temperature, and EtOAc (5 mL) was added. The reaction mixture was washed with water (2 × 10 mL), and the organic phase was dried over anhydrous MgSO_4_ and then evaporated under reduced pressure. The resulting mixture was purified by flash chromatography (SiO_2_, cyclohexane/EtOAc = 100:0 → 60:40) to afford the desired nitrile **13** (47 mg, 61 %). ^1^H NMR (400 MHz, CDCl_3_) *δ =* 7.79 (d, *J* = 1.5 Hz, 1H), 7.75 (d, *J* = 2.2 Hz, 1H), 7.73–7.71 (m, 1H), 7.38 (d, *J* = 8.4 Hz, 1H), 7.27 (d, *J* = 1.5 Hz, 1H), 6.67 (s, 1H), 2.46 (s, 3H), 2.35 (s, 3H), 2.34 (s, 3H) ppm. ^13^C NMR (101 MHz, CDCl_3_) *δ =* 175.5, 169.2, 168.0, 167.7, 161.4, 156.8, 150.2, 145.3, 142.8, 128.9, 124.6, 124.5, 122.1, 121.8, 120.6, 120.2, 116.9, 116.2, 109.5, 21.0, 20.7, 20.6 ppm. HRMS (ESI) *m/z* calcd for C_22_H_15_NO_8_Na [M+Na^+^]^+^ 444.0690, found 444.0691.

### 3.21. 2-(3,4-Dihydroxyphenyl)-5-hydroxy-7-(2H-tetrazol-5-yl)-4H-chromen-4-one (***14***)

A tube with 4-(5-acetoxy-7-cyano-4-oxo-4*H*-chromen-2-yl)-1,2-phenylene diacetate (**13**) (58 mg, 0.134 mmol, 1.0 eq) and NaN_3_ (36 mg, 0.562 mmol, 4.0 eq.) was sealed and the mixture was dissolved in dry DMF (0.5 mL). The mixture was degassed with a stream of argon for 10 min, followed by addition of glacial acetic acid (2 drops) via septum and the reaction mixture was heated to 130 °C for 16 h until the starting material was fully consumed (TLC, UPLC-MS). The reaction mixture was cooled to room temperature, and 4 M HCl was added until the pH reached 2. The solvents were evaporated, and the residue was purified by preparative HPLC to obtain the final tetrazole derivative **14** (18 mg, 38%). ^1^H NMR (400 MHz, DMSO*-d*_6_) *δ =* 13.02 (s, 1H), 10.09 (s, 1H), 9.45 (s, 1H), 7.77 (d, *J* = 1.5 Hz, 1H), 7.52 (dd, *J* = 8.4, 2.4 Hz, 1H), 7.49 (d, *J* = 2.3 Hz, 1H), 7.39 (d, *J* = 1.6 Hz, 1H), 6.98 –6.86 (m, 2H) ppm. ^13^C NMR (101 MHz, DMSO*-d*_6_) *δ =* 182.3, 165.4, 160.5, 156.1, 150.4, 145.9, 130.5, 121.0, 119.6, 116.1, 113.7, 111.2, 108.7, 105.8, 103.9 ppm. HRMS (ESI) *m/z* calcd for C_16_H_11_N_4_O_5_ [M+H^+^]^+^ 339.0724, found 339.0724.

### 3.22. 2-(3,4-Dihydroxyphenyl)-5-hydroxy-4-oxo-4H-chromene-7-carbonitrile (***15***)

4-(5-Acetoxy-7-cyano-4-oxo-4*H*-chromen-2-yl)-1,2-phenylene diacetate (**13**) (47 mg, 0.11 mmol, 1.0 eq.) was dissolved in THF (1.1 mL), followed by addition of 2 M aq. LiOH (0.33 mL, 0.66 mmol, 6.0 eq.). The reaction mixture was allowed to stir for 24 h at room temperature under a nitrogen atmosphere until the starting material was fully consumed (UPLC-MS). Then, solvents were evaporated and the residue was purified by preparative HPLC to obtain the final nitrile **15** (25 mg, 77%). ^1^H NMR (400 MHz, DMSO*-d*_6_) *δ =* 13.12 (s, 1H), 10.15 (s, 1H), 9.46 (s, 1H), 7.72 (d, *J* = 1.3 Hz, 1H), 7.50 (dd, *J* = 8.3, 2.3 Hz, 1H), 7.46 (d, *J* = 2.3 Hz, 1H), 7.25 (d, *J* = 1.5 Hz, 1H), 6.94 (s, 1H), 6.91 (d, *J* = 8.3 Hz, 1H) ppm. ^13^C NMR (101 MHz, DMSO*-d*_6_) *δ =* 182.3, 165.7, 160.3, 155.5, 150.6, 145.9, 120.8, 119.8, 117.4, 116.6, 116.0, 113.9, 112.8, 111.8, 104.2 ppm. HRMS (ESI) *m/z* calcd for C_16_H_10_NO_5_ [M+H^+^]^+^ 296.0554, found 296.0551.

### 3.23. Tert-Butyl ((2-(3,4-dihydroxyphenyl)-5-hydroxy-4-oxo-4H-chromen-7-yl)methyl)carbamate (***16***)

A tube with 4-(5-acetoxy-4-oxo-7-(((trifluoromethyl)sulfonyl)oxy)-4*H*-chromen-2-yl)-1,2-phenylene diacetate (**3**) (100 mg, 0.184 mmol, 1.0 eq.), potassium (((*tert*-butoxy-carbonyl)amino)methyl)trifluoroborate (66 mg, 0.276 mmol, 1.5 eq.), K_2_CO_3_ (127 mg, 0.552 mmol, 3.0 eq.) and SPhos Pd G2 (15 mg, 0.018 mmol, 0.1 eq.) was sealed, and THF/water (25:1, 3.5 mL) was added via septum. The mixture was degassed with a stream of argon for 15 min, followed by heating to 100 °C for 2 h until the starting material was fully consumed (TLC, UPLC-MS). The reaction mixture was cooled to room temperature, diluted with EtOAc (2 mL), and filtered through a syringe filter. The solvents were evaporated and the residue was purified by flash chromatography (SiO_2_-C_18_, H_2_O/MeCN= 100:0 → 0:100) to afford the desired carbamate **16** (32 mg, 43%). ^1^H NMR (400 MHz, DMSO*-d*_6_) *δ =* 12.78 (s, 1H), 7.54 (t, *J* = 6.2 Hz, 1H), 7.47 (dd, *J* = 8.3, 2.3 Hz, 1H), 7.44 (d, *J* = 2.3 Hz, 1H), 6.98 (s, 1H), 6.91 (d, *J* = 8.3 Hz, 1H), 6.81 (s, 1H), 6.67 (d, *J* = 1.3 Hz, 1H), 4.21 (d, *J* = 6.1 Hz, 2H), 1.42 (s, 9H) ppm. ^13^C NMR (101 MHz, DMSO*-d*_6_) *δ =* 183.0, 165.2, 160.2, 156.2, 150.7, 149.8, 146.3, 121.6, 119.8, 116.5, 113.9, 109.5, 109.1, 105.5, 103.8, 78.6, 43.9, 28.7 ppm. HRMS (ESI) *m/z* calcd for C_21_H_22_NO_7_ [M+H^+^]^+^ 400.1391, found 400.1386.

### 3.24. 7-(Aminomethyl)-2-(3,4-dihydroxyphenyl)-5-hydroxy-4H-chromen-4-one (***17***)

Tert-butyl ((2-(3,4-dihydroxyphenyl)-5-hydroxy-4-oxo-4*H*-chromen-7-yl)methyl) carbamate (**16**) (25 mg, 0.063 mmol) was dissolved in THF/water (4:1, 1.5 mL), followed by addition of trifluoroacetic acid (0.6 mL). The reaction mixture was heated to 50 °C and allowed to stir for 48 h. The solvents were evaporated and the residue was purified by preparative HPLC to obtain the final amine **17** (19 mg, 73%). ^1^H NMR (400 MHz, DMSO*-d*_6_) *δ =* 12.85 (s, 1H), 10.12 (s, 1H), 9.52 (s, 1H), 8.40 (s, 2H), 7.51–7.42 (m, 2H), 7.24 (d, *J* = 1.6 Hz, 1H), 6.97–6.90 (m, 2H), 6.87 (d, *J* = 1.8 Hz, 1H) ppm. ^13^C NMR (101 MHz, DMSO*-d*_6_) *δ =* 182.5, 165.1, 159.9, 155.6, 150.3, 145.9, 142.1, 121.1, 119.3, 116.1, 113.6, 110.9, 109.5, 107.3, 103.7, 42.0 ppm. HRMS (ESI) *m/z* calcd for C_16_H_14_NO_5_ [M+H^+^]^+^ 300.0867, found 300.0870.

### 3.25. 2-(3,4-Dihydroxyphenyl)-5-hydroxy-7-(1H-pyrrolo[2 ,3-B]pyridin-3-yl)-4H-chromen-4-one (***18***)

A tube with 4-(5-acetoxy-4-oxo-7-(((trifluoromethyl)sulfonyl)oxy)-4*H*-chromen-2-yl)-1,2-phenylene diacetate (**3**) (100 mg, 0.184 mmol, 1.0 eq.), 3-(4,4,5,5-tetramethyl-1,3,2-dioxaborolan-2-yl)-1*H*-pyrrolo[2,3-*b*]pyridine (54 mg, 0.221 mmol, 1.2 eq.), Cs_2_CO_3_ (240 mg, 0.736 mmol, 4.0 eq.) and Pd(PPh_3_)_4_ (21 mg, 0.018 mmol, 0.1 eq.) was sealed, and dry THF (3.5 mL) was added via septum. The mixture was degassed with a stream of argon for 15 min. then heated to 100 °C for 16 h until the starting material was fully consumed (TLC, UPLC-MS). The reaction mixture was cooled to room temperature, diluted with MeOH (5 mL), and filtered through a syringe filter. The solvents were evaporated and the residue was purified by preparative HPLC to obtain the final product **18** (6.6 mg, 10%). ^1^H NMR (400 MHz, DMSO*-d*_6_) *δ =* 12.87 (s, 1H), 12.27 (s, 1H), 10.02 (s, 1H), 9.42 (s, 1H), 8.49 (dd, *J* = 8.1, 1.6 Hz, 1H), 8.34 (dd, *J* = 4.6, 1.6 Hz, 1H), 8.25 (d, *J* = 2.8 Hz, 1H), 7.60 – 7.43 (m, 3H), 7.28–7.23 (m, 1H), 7.20 (d, *J* = 1.5 Hz, 1H), 6.94 (d, *J* = 8.1 Hz, 1H), 6.81 (s, 1H) ppm. ^13^C NMR (101 MHz, DMSO*-d*_6_) *δ =* 182.6, 165.1, 160.6, 156.9, 150.4, 149.5, 146.3, 143.7, 143.2, 128.5, 127.3, 122.0, 119.8, 117.6, 117.2, 116.4, 114.2, 113.3, 108.3, 108.2, 104.4, 103.8 ppm. HRMS (ESI) *m/z* calcd for C_22_H_13_N_2_O_5_ [M−H^+^]^–^ 385.0830, found 385.0827.

### 3.26. 2-(3,4-Dihydroxyphenyl)-5-hydroxy-7-(1H-pyrazol-3-yl)-4H-chromen-4-one (***19***)

A tube with 4-(5-acetoxy-4-oxo-7-(((trifluoromethyl)sulfonyl)oxy)-4*H*-chromen-2-yl)-1,2-phenylene diacetate (**3**) (100 mg, 0.184 mmol, 1.0 eq.), (1*H*-pyrazol-3-yl)boronic acid (31 mg, 0.276 mmol, 1.5 eq.), K_2_CO_3_ (127 mg, 0.920 mmol, 5.0 eq.), and SPhos Pd G2 (15 mg, 0.018 mmol, 0.1 eq.) was sealed, and dry THF (3.5 mL) was added via septum. The mixture was degassed with a stream of argon for 15 min, then heated to 100 °C for 16 h until the starting material was fully consumed (TLC, UPLC-MS). The reaction mixture was cooled to room temperature, diluted with MeOH (5 mL), and filtered through a syringe filter. The solvents were evaporated and the residue was purified by preparative HPLC to obtain the final product **19** (15 mg, 25%). ^1^H NMR (400 MHz, DMSO*-d*_6_) *δ =* 12.84 (s, 1H), 7.83 (d, *J* = 2.3 Hz, 1H), 7.59 (d, *J* = 1.5 Hz, 1H), 7.52–7.46 (m, 2H), 7.27 (d, *J* = 1.5 Hz, 1H), 6.96 (d, *J* = 2.3 Hz, 1H), 6.92 (d, *J* = 8.2 Hz, 1H), 6.81 (s, 1H) ppm. ^13^C NMR (101 MHz, DMSO*-d*_6_) *δ =* 182.4, 164.9, 160.1, 157.3, 156.3, 150.0, 147.3, 145.8, 140.1, 132.2, 121.4, 119.3, 116.0, 113.7, 109.0, 107.2, 103.6, 103.5 ppm. HRMS (ESI) *m/z* calcd for C_18_H_13_N_2_O5 [M+H^+^]^+^ 337.0819, found 337.0819.

### 3.27. 2-(3,4-Dihydroxyphenyl)-5-hydroxy-7-(5-methyl-1H-pyrazol-3-yl)-4H-chromen-4-one (***20***)

A tube with 4-(5-acetoxy-4-oxo-7-(((trifluoromethyl)sulfonyl)oxy)-4*H*-chromen-2-yl)-1,2-phenylene diacetate (**3**) (100 mg, 0.184 mmol, 1.0 eq.), (5-methyl-1*H*-pyrazol-3-yl)boronic acid (35 mg, 0.276 mmol, 1.5 eq.), K_2_CO_3_ (127 mg, 0.920 mmol, 5 eq.), and SPhos Pd G2 (15 mg, 0.018 mmol, 0.1 eq.) was sealed, and THF/water (25:1, 3.5 mL) was added via septum. The mixture was degassed with a stream of argon for 15 min, then heated to 100 °C for 16 h until the starting material was fully consumed (TLC, UPLC-MS). The reaction mixture was cooled to room temperature, diluted with MeOH (5 mL), and filtered through a syringe filter. Then solvents were evaporated and the residue was purified by preparative HPLC to obtain the final product **20** (12 mg, 19%). ^1^H NMR (400 MHz, DMSO*-d*_6_) *δ =* 7.51 (d, *J* = 1.5 Hz, 1H), 7.51–7.46 (m, 2H), 7.19 (d, *J* = 1.3 Hz, 1H), 6.92 (d, *J* = 8.2 Hz, 1H), 6.80 (s, 1H), 6.66 (d, *J* = 1.0 Hz, 1H), 2.28 (s, 3H) ppm. ^13^C NMR (101 MHz, DMSO*-d*_6_) *δ =* 182.3, 164.8, 160.1, 158.4, 158.1, 156.2, 150.0, 145.8, 140.3, 121.4, 119.3, 116.0, 113.7, 108.9, 107.0, 103.5, 103.3, 102.7, 10.9 ppm. HRMS (ESI) *m/z* calcd for C_19_H_15_N_2_O_5_ [M+H^+^]^+^ 351.0976, found 351.0975.

### 3.28. 2-(3,4-Dihydroxyphenyl)-5,7-dihydroxy-8-((3-hydroxypiperidin-1-yl)methyl)-4H-chromen-4-one (***21***)

Luteolin (100 mg, 0.35 mmol, 1.0 eq.) was dissolved in MeOH (5 mL), followed by addition of 3-hydroxypiperidine (35 mg, 0.35 mmol, 1.0 eq.) and a 35% formalin solution (27 µL, 0.35 mmol, 1.0 eq.). The reaction mixture was allowed to stir at room temperature for 16 h. The solvents were evaporated and the residue was purified by preparative HPLC to obtain the final product **21** (27 mg, 20%). ^1^H NMR (500 MHz, DMSO*-d*_6_, 330 K) *δ =* 13.27 (s, 1H), 9.89 (s, 1H), 9.35 (s, 1H), 7.47 (dd, *J* = 8.2, 2.3 Hz, 1H), 7.45 (d, *J* = 2.3 Hz, 1H), 6.94 (d, *J* = 8.2 Hz, 1H), 6.74 (d, *J* = 0.6 Hz, 1H), 6.39 (s, 1H), 5.33 (s, 1H), 4.63–4.29 (m, 2H), 4.01–2.80 (m, 5H), 2.11–1.31 (m, 4H) ppm. ^13^C NMR (126 MHz, DMSO*-d*_6_, 330 K) *δ =* 181.7, 164.1, 163.7, 162.5, 156.5, 150.0, 145.9, 121.5, 119.3, 116.2, 113.7, 103.9, 103.2, 98.4, 95.5, 57.3, 51.9, 49.1 ppm. HRMS (ESI) *m/z* calcd for C_21_H_20_NO_7_ [M−H^+^]^–^ 398.1245, found 398.1244.

### 3.29. 2-(3,4-Dihydroxyphenyl)-5,7-dihydroxy-8-((4-hydroxypiperidin-1-yl)methyl)-4H-chromen-4-one (***22***)

Luteolin (100 mg, 0.35 mmol, 1.0 eq.) was dissolved in MeOH (5 mL), followed by addition of 4-hydroxypiperidine (35 mg, 0.35 mmol, 1.0 eq.) and a 35% formalin solution (27 µL, 0.35 mmol, 1.0 eq.). The reaction mixture was allowed to stir at room temperature for 16 h. The solvents were evaporated and the residue was purified by preparative HPLC to obtain the final product **22** (58 mg, 32%). ^1^H NMR (400 MHz, DMSO*-d*_6_) *δ =* 13.32 (s, 1H), 12.17 (br s, 1H), 10.12 (br s, 1H), 9.38 (br s, 1H), 7.56–7.44 (m, 2H), 6.95 (d, *J* = 9.0 Hz, 1H), 6.79 (s, 1H), 6.43 (s, 2H), 4.43 (br s, 2H), 3.91 (br s, 1H), 3.39 – 2.97 (m, 4H), 1.89–1.48 (m, 4H) ppm. ^13^C NMR (101 MHz, DMSO*-d*_6_) *δ =* 182.2, 164.5, 164.2, 162.8, 156.8, 150.4, 146.3, 121.8, 119.7, 116.6, 113.8, 104.2, 103.5, 98.8, 96.1, 59.9, 51.1, 47.9, 31.7 ppm. HRMS (ESI) *m/z* calcd for C_21_H_22_NO_7_ [M+H^+^]^+^ 400.1391, found 400.1387.

### 3.30. 1-((2-(3,4-Dihydroxyphenyl)-5,7-dihydroxy-4-oxo-4H-chromen-8-yl)methyl)piperidin-4-one (***23***)

Luteolin (100 mg, 0.35 mmol, 1.0 eq.) was dissolved in MeOH (5 mL), followed by addition of 4-piperidone monohydrate hydrochloride (54 mg, 0.35 mmol, 1.0 eq.), 35% formalin solution (27 µL, 0.35 mmol, 1.0 eq.), and triethylamine (49 µL, 0.35 mmol, 1.0 eq.). The reaction mixture was allowed to stir at room temperature for 16 h. Then, solvents were evaporated and the residue was purified by preparative HPLC to obtain the final product **23** as a 3:1 mixture of C-8 and C-6 regioisomers (38 mg, 21%). Spectral data includes the C-8 regioisomer only. ^1^H NMR (400 MHz, DMSO*-d*_6_) *δ =* 13.09 (s, 1H), 7.49–7.45 m, 2H), 6.93–6.91 (m, 1H), 6.73 (s, 1H), 6.29 (s, 1H), 3.97 (s, 2H), 2.93 (t, *J* = 5.8 Hz, 4H), 2.41 (t, *J* = 6.1 Hz, 4H) ppm. ^13^C NMR (101 MHz, DMSO*-d*_6_) *δ =* 208.1, 183.3, 164.2, 164.0, 161.0, 155.8, 150.2, 146.2, 122.2, 119.5, 116.5, 113.9, 104.1, 103.1, 98.9, 94.1, 52.5, 49.4, 40.8 ppm. HRMS (ESI) *m/z* calcd for C_21_H_18_NO_7_ [M−H^+^]^–^ 396.1089, found 396.1085.

### 3.31. 2-(3,4-Dihydroxyphenyl)-5,7-dihydroxy-8-(morpholinomethyl)-4H-chromen-4-one (***24***)

Luteolin (100 mg, 0.35 mmol, 1.0 eq.) was dissolved in MeOH (5 mL), followed by addition of morpholine (30 µL, 0.35 mmol, 1.0 eq.) and a 35% formalin solution (27 µL, 0.35 mmol, 1.0 eq.). The reaction mixture was allowed to stir at room temperature for 16 h. Then, solvents were evaporated and the residue was purified by preparative HPLC to obtain the final product **24** (46 mg, 26%). ^1^H NMR (400 MHz, DMSO*-d*_6_, 330 K) *δ =* 13.33 (s, 1H), 12.13 (br s, 1H), 10.07 (br s, 1H), 9.59 (br s, 1H), 7.51 (dd, *J* = 8.3, 2.3 Hz, 1H), 7.48 (d, *J* = 2.3 Hz, 1H), 6.93 (d, *J* = 8.3 Hz, 1H), 6.79 (s, 1H), 6.42 (s, 1H), 4.47 (s, 2H), 4.01–3.20 (m, 8H) ppm. ^13^C NMR (101 MHz, DMSO*-d*_6_, 330 K) *δ =* 181.8, 164.1, 163.9, 162.5, 156.4, 150.0, 145.9, 121.3, 119.4, 116.2, 113.6, 103.7, 103.0, 98.4, 95.2, 63.2, 51.5, 48.9 ppm. HRMS (ESI) *m/z* calcd for C_20_H_18_NO_7_ [M−H^+^]^—^ 384.1089, found 384.1087.

### 3.32. 2-(3,4-Dihydroxyphenyl)-5,7-dihydroxy-8-((4-methylpiperazin-1-yl)methyl)-4H-chromen-4-one (***25***)

Luteolin (100 mg, 0.35 mmol, 1.0 eq.) was dissolved in MeOH (5 mL), followed by addition of *N*-methyl-piperazine (40 µL, 0.35 mmol, 1.0 eq.) and a 35% formalin solution (27 µL, 0.35 mmol, 1.0 eq.). The reaction mixture was allowed to stir at room temperature for 16 h. Then, solvents were evaporated and the residue was purified by preparative HPLC to obtain the final product **25** (44 mg, 31%). ^1^H NMR (500 MHz, DMSO*-d*_6_, 330 K) *δ =* 13.10 (br s, 1H), 7.46–7.41 (m, 2H), 6.93 (d, *J* = 8.7 Hz, 1H), 6.68 (s, 1H), 6.34 (s, 1H), 4.01 (br s, 2H), 3.34–2.87 (m, 8H), 2.75 (s, 3H) ppm. ^13^C NMR (126 MHz, DMSO*-d*_6_) *δ =* 181.9, 164.0, 163.3, 161.2, 155.9, 149.9, 145.9, 121.7, 119.1, 116.2, 113.6, 103.8, 102.9, 98.4, 51.9, 49.1, 48.4, 42.3 ppm. HRMS (ESI) *m/z* calcd for C_21_H_21_N_2_O_6_ [M−H^+^]^–^ 397.1405, found 397.1403.

### 3.33. 2-(3,4-Dihydroxyphenyl)-5,7-dihydroxy-8-(((2-hydroxyethyl)(methyl)amino)methyl)-4H-chromen-4-one (***26***)

Luteolin (100 mg, 0.35 mmol, 1.0 eq.) was dissolved in MeOH (5 mL), followed by addition of *N*-methylethanolamine (28 µL, 0.35 mmol, 1.0 eq.) and a 35% formalin solution (27 µL, 0.35 mmol, 1.0 eq.). The reaction mixture was allowed to stir at room temperature for 16 h. Then, solvents were evaporated and the residue was purified by preparative HPLC to obtain the final product **26** (42 mg, 32%). ^1^H NMR (400 MHz, DMSO*-d*_6_) *δ =* 13.96 (s, 1H), 12.21 (br s, 1H), 10.08 (br s, 1H), 9.54 (br s, 1H), 7.47 – 7.41 (m, 2H), 6.91 (d, *J* = 9.0 Hz, 1H), 6.76 (s, 1H), 6.65 (s, 1H), 5.34 (br s, 1H), 4.27 (br s, 2H), 3.80 (t, *J* = 5.4 Hz, 2H), 3.25 (br s, 2H), 2.75 (s, 3H) ppm. ^13^C NMR (101 MHz, DMSO*-d*_6_) *δ =* 181.8, 164.4, 163.5, 161.3, 157.6, 150.1, 145.9, 121.2, 119.2, 116.1, 113.5, 103.3, 102.9, 100.3, 93.5, 57.6, 55.4, 47.7, 40.5 ppm. HRMS (ESI) *m/z* calcd for C_19_H_20_NO_7_ [M+H^+^]^+^ 374.1234, found 374.1235.

### 3.34. 2-(3,4-Dihydroxyphenyl)-5,7-dihydroxy-8-((3-hydroxy-8-azabicyclo[3.2.1]octan-8-yl)methyl)-4H-chromen-4-one (***27***)

Luteolin (100 mg, 0.35 mmol, 1.0 eq.) was dissolved in MeOH (5 mL), followed by an addition of nortropine hydrochloride (58 mg, 0.35 mmol, 1.0 eq.), a 35% formalin solution (27 µL, 0.35 mmol, 1.0 eq.), and triethylamine (49 µL, 0.35 mmol, 1.0 eq.). The reaction mixture was allowed to stir at room temperature for 16 h. Then, solvents were evaporated and the residue was purified by preparative HPLC to obtain the final product **27** (10 mg, 7%). ^1^H NMR (500 MHz, DMSO*-d*_6_) *δ =* 13.27 (s, 1H), 11.94 (br s, 1H), 10.09 (br s, 1H), 9.51 (br s, 1H), 8.83 (br s, 1H), 7.48 (dd, *J* = 8.3, 2.4 Hz, 1H), 7.43 (d, *J* = 2.4 Hz, 1H), 6.93 (d, *J* = 8.4 Hz, 1H), 6.81 (s, 1H), 6.38 (s, 1H), 4.95 (br s, 1H), 4.27 (s, 2H), 4.17–3.66 (m, 4H), 2.47–2.35 (m, 4H), 2.18 (br d, *J* = 15.4 Hz, 2H), 1.88 (br d, *J* = 14.3 Hz, 2H) ppm. ^13^C NMR (126 MHz, DMSO*-d*_6_) *δ =* 181.9, 164.3, 163.7, 162.5, 156.6, 150.2, 146.1, 121.7, 119.5, 116.3, 113.8, 104.0, 103.4, 98.5, 96.7, 62.1, 60.6, 44.9, 37.2 (2C), 24.4 (2C) ppm. HRMS (ESI) *m/z* calcd for C_23_H_22_NO_7_ [M−H^+^]^–^ 424.1402, found 424.1400.

### 3.35. 8-((6,7-Dihydroxy-3,4-dihydroisoquinolin-2(1H)-yl)methyl)-2-(3,4-dihydroxyphenyl)-5,7-dihydroxy-4H-chromen-4-one (***28***)

Luteolin (100 mg, 0.35 mmol, 1.0 eq.) was dissolved in MeOH (5 mL), followed by addition of 6,7-dihydroxy-1,2,3,4-tetrahydroisoquinolin-2-ium chloride (71 mg, 0.35 mmol, 1.0 eq.), a 35% formalin solution (27 µL, 0.35 mmol, 1.0 eq.), and triethylamine (49 µL, 0.35 mmol, 1.0 eq.). The reaction mixture was allowed to stir at room temperature for 16 h. Then, solvents were evaporated and the residue was purified by preparative HPLC to obtain the final product **28** (46 mg, 23 %). ^1^H NMR (400 MHz, DMSO*-d*_6_) *δ =* 13.29 (s, 1H), 12.07 (br s, 1H), 10.07 (br s, 1H), 9.62 (br s, 1H), 9.12 (br s, 1H), 9.06 (s, 1H), 7.52–7.46 (m, 1H), 7.45 (d, *J* = 2.3 Hz, 1H), 6.92 (d, *J* = 8.3 Hz, 1H), 6.79 (s, 1H), 6.57 (s, 1H), 6.53 (s, 1H), 6.41 (s, 1H), 4.54 (s, 2H), 4.36 (s, 2H), 3.58 (br s, 2H), 2.91 (t, *J* = 6.9 Hz, 2H) ppm. ^13^C NMR (101 MHz, DMSO*-d*_6_) *δ =* 182.2, 164.4, 164.2, 162.8, 156.8, 150.4, 146.3, 145.9, 144.9, 121.8 (2C), 119.7, 118.9, 116.6, 115.3, 114.0, 113.5, 104.2, 103.4, 98.7, 96.1, 53.0, 49.8, 48.2, 24.8 ppm. HRMS (ESI) *m/z* calcd for C_25_H_20_NO_8_ [M−H^+^]^–^ 462.1194, found 462.1193.

### 3.36. (S)-1-((2-(3,4-Dihydroxyphenyl)-5,7-dihydroxy-4-oxo-4H-chromen-8-yl)methyl)pyrro-lidine-2-carboxamide (***29***)

Luteolin (100 mg, 0.35 mmol, 1.0 eq.) was dissolved in EtOH (5 mL), followed by an addition of *L*-prolinamide hydrochloride (71 mg, 0.35 mmol, 1.0 eq.), a 35% formalin solution (27 µL, 0.35 mmol, 1.0 eq.), and triethylamine (49 µL, 0.35 mmol, 1.0 eq.). The reaction mixture was allowed to stir at room temperature for 16 h. Then, solvents were evaporated and the residue was purified by preparative HPLC to obtain the final product **29** (19 mg, 10%). ^1^H NMR (400 MHz, DMSO*-d*_6_) *δ =* 13.31 (s, 1H), 11.85 (br s, 1H), 10.08 (br s, 1H), 9.52 (br s, 1H), 9.38 (br s, 1H), 7.96 (s, 1H), 7.69 (s, 1H), 7.55 (dd, *J* = 8.4, 2.3 Hz, 1H), 7.47 (d, *J* = 2.3 Hz, 1H), 6.93 (d, *J* = 8.4 Hz, 1H), 6.77 (s, 1H), 6.34 (s, 1H), 4.54 (d, *J_gem_* = 13.7 Hz, 1H), 4.48 (d, *J_gem_* = 13.7 Hz, 1H), 4.16 (br d, *J* = 9.3 Hz, 1H), 3.56 (br s, 2H), 2.47–2.35 (m, 1H), 2.04–1.93 (m, 1H), 1.89–1.77 (m, 2H) ppm. ^13^C NMR (101 MHz, DMSO*-d*_6_) *δ =* 181.8, 169.3, 164.1, 163.6, 162.4, 156.4, 150.0, 145.8, 121.3, 119.6, 116.0, 113.9, 103.7, 103.0, 98.3, 96.0, 65.9, 53.9, 45.5, 29.2, 22.3 ppm. HRMS (ESI) *m/z* calcd for C_21_H_21_N_2_O_7_ [M+H^+^]^+^ 413.1343, found 413.1344.

### 3.37. ((2-(3,4-Dihydroxyphenyl)-5,7-dihydroxy-4-oxo-4H-chromen-8-yl)methyl)-L-proline (***30***)

Luteolin (100 mg, 0.35 mmol, 1.0 eq.) was dissolved in EtOH (5 mL), followed by addition of *L*-proline tert-butyl ester hydrochloride (72 mg, 0.35 mmol, 1.0 eq.) and a 35% formalin solution (27 µL, 0.35 mmol, 1.0 eq.). The reaction mixture was stirred at room temperature for 16 h. The solvent was evaporated under reduced pressure and the residue was purified by preparative HPLC to obtain the monosubstituted luteolin C-8 derivative as tert-butyl ester (UPLC-MS: *m/z* = 470). The monosubstituted luteolin C-8 derivative was used in the next step without further characterization. The tert-butyl ester was dissolved in a mixture of TFA/DCM (1:1, 1 mL) with 1,3-dimethoxybenzene (25 µL, 0.19 mmol, 0.54 eq.) as a scavenger. The reaction mixture was allowed to stir at room temperature for 16 h. The reaction mixture was concentrated using a stream of nitrogen, and the product was precipitated with tert-butyl methyl ether. The precipitate was collected and purified by preparative HPLC to obtain the product **30** (9.5 mg, 7% – over two steps). ^1^H NMR (500 MHz, DMSO*-d_6_*) *δ =* 13.25 (s, 1H), 9.91 (br s, 1H), 7.68 (d, *J* = 2.3 Hz, 1H), 7.48 (dd, *J* = 8.4, 2.3 Hz, 1H), 6.88 (d, *J* = 8.2 Hz, 1H), 6.77 (s, 1H), 6.31 (s, 1H), 4.50 (d, *J_gem_* = 13.7 Hz, 1H), 4.39 (d, *J_gem_* = 13.7 Hz, 1H), 4.03 (m, 1H), 3.39 (m, 1H), 3.13–2.98 (m, 1H), 2.39–2.31 (m, 1H), 1.96 (m, 2H), 1.79 (m, 1H) ppm. ^13^C NMR (126 MHz, DMSO*-d_6_*) *δ =* 181.9, 170.8, 164.0, 163.7, 161.9, 156.0, 149.8, 145.9, 121.2, 119.0, 116.1, 114.3, 103.7, 102.7, 98.3, 97.4, 67.0, 53.6, 46.1, 28.4, 22.5. HRMS (ESI) *m/z* calcd for C_21_H_18_NO_8_ [M−H^+^]^–^ 412.1035, found 412.1037.

### 3.38. 1-((2-(3,4-Dihydroxyphenyl)-5,7-dihydroxy-4-oxo-4H-chromen-8-yl)methyl)piperidine-4-carboxylic acid (***31***)

Luteolin (143 mg, 0.50 mmol, 1.0 eq.) was dissolved in MeOH (7 mL), followed by addition of ethyl 4-piperidinecarboxylate (77 µL, 0.50 mmol, 1.0 eq.) and a 35% formalin solution (38 µL, 0.35 mmol, 1.0 eq.). The reaction mixture was allowed to stir at room temperature for 16 h. The solvent was evaporated under reduced pressure and the residue was purified by flash chromatography (SiO_2_-C_18_, H_2_O (0.1% TFA)/MeCN= 100:0 → 20:80) to obtain the monosubstituted luteolin C-8 derivative as an ethyl ester (UPLC-MS: *m/z* = 456). The monosubstituted luteolin C-8 derivative was used in the next step without further characterization. The ethyl ester was dissolved in THF (2 mL) and 5% aq. HCl (2 mL) was added. The reaction mixture was allowed to stir at 70 °C for 7 h until the starting material was fully consumed (UPLC-MS). Then, solvents were evaporated and the residue was purified by preparative HPLC to obtain the final product **31** (60 mg, 22% – over 2 steps). ^1^H NMR (400 MHz, DMSO*-d*_6_) *δ =* 13.31 (s, 1H), 12.07 (br s, 1H), 10.09 (br s, 1H), 9.56 (br s, 1H), 7.49 (dd, *J* = 8.3, 2.3 Hz, 1H), 7.46 (d, *J* = 2.2 Hz, 1H), 6.93 (d, *J* = 8.3 Hz, 1H), 6.79 (s, 1H), 6.40 (s, 1H), 4.41 (s, 2H), 3.73–3.37 (m, 4H), 3.28–3.08 (m, 1H), 2.13–1.69 (m, 4H) ppm. ^13^C NMR (101 MHz, DMSO*-d*_6_) *δ =* 181.8, 174.7, 164.1, 163.8, 162.5, 156.4, 150.0, 145.9, 121.3, 119.3, 116.2, 113.6, 103.8, 103.1, 98.4, 95.6, 51.5 (2C), 48.7, 37.6, 25.4 (2C) ppm. HRMS (ESI) *m/z* calcd for C_22_H_20_NO_8_ [M−H^+^]^–^ 426.1194, found 426.1190.

### 3.39. N-((2-(3,4-Dihydroxyphenyl)-5,7-dihydroxy-4-oxo-4H-chromen-8-yl)methyl)-N-methylglycine (***32***)

Luteolin (143 mg, 0.50 mmol, 1.0 eq.) was dissolved in MeOH (7 mL), followed by addition of sarcosine methyl ester hydrochloride (70 mg, 0.50 mmol, 1.0 eq.), a 35% formalin solution (38 µL, 0.35 mmol, 1.0 eq.), and triethylamine (70 µL, 0.50 mmol, 1.0 eq.). The reaction mixture was allowed to stir at room temperature for 16 h. The solvent was evaporated under reduced pressure and the residue was purified by flash chromatography (SiO_2_-C_18_, H_2_O (0.1% TFA)/MeCN= 100:0 → 40:60) to obtain the monosubstituted luteolin C-8 derivative as a methyl ester (UPLC-MS: *m/z* = 402). The monosubstituted luteolin C-8 derivative was used in the next step without further characterization. The methyl ester was dissolved in THF (2 mL) and 5% aq. HCl (2 mL) was added. The reaction mixture was allowed to stir at 70 °C for 3 days until the starting material was fully consumed (UPLC-MS). The solvents were evaporated and the residue was purified by preparative HPLC to obtain the final product **32** (29 mg, 12% – over 2 steps). ^1^H NMR (400 MHz, DMSO*-d*_6_) *δ =* 13.30 (s, 1H), 10.09 (br s, 1H), 9.54 (br s, 1H), 7.52 (dd, *J* = 8.3, 2.3 Hz, 1H), 7.49 (d, *J* = 2.3 Hz, 1H), 6.92 (d, *J* = 8.3 Hz, 1H), 6.76 (s, 1H), 6.35 (s, 1H), 4.49 (s, 2H), 4.00 (s, 2H), 2.75 (s, 3H) ppm. ^13^C NMR (101 MHz, DMSO*-d*_6_) *δ =* 181.8, 168.1, 164.0 (2C), 162.3, 156.4, 149.9, 145.9, 121.3, 119.3, 116.0, 113.8, 103.8, 103.0, 98.4, 96.1, 55.5, 48.2, 40.9 ppm. HRMS (ESI) *m/z* calcd for C_19_H_18_NO_8_ [M+H^+^]^+^ 388.1027, found 388.1026.

## 4. Conclusions

Bio-isosteric replacement of the hydroxyl group at C-7 failed to provide luteolin congeners with comparable inhibitory potency against the influenza PA-Nter domain. However, the introduction of diverse substituents at C-8 increased the binding affinity of the resulting compounds. Crystallographic study of the orientin complex with I38T PA-Nter revealed an additional point of interaction of the flavonoid with Thr-38 mediated by a water molecule.

## Figures and Tables

**Table 1:** Inhibition assay of influenza endonuclease. Structure-activity relationship of C-7 luteolin derivatives.

Compound	Structure	IC_50_ (μM) (AlphaScreen)
luteolin	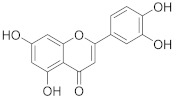	0.073 ± 0.003
**5**	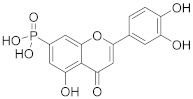	9.2 ± 2.3
**8**	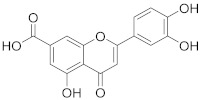	0.98 ± 0.13
**9**	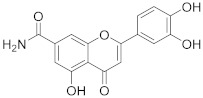	2.0 ± 0.6
**10**	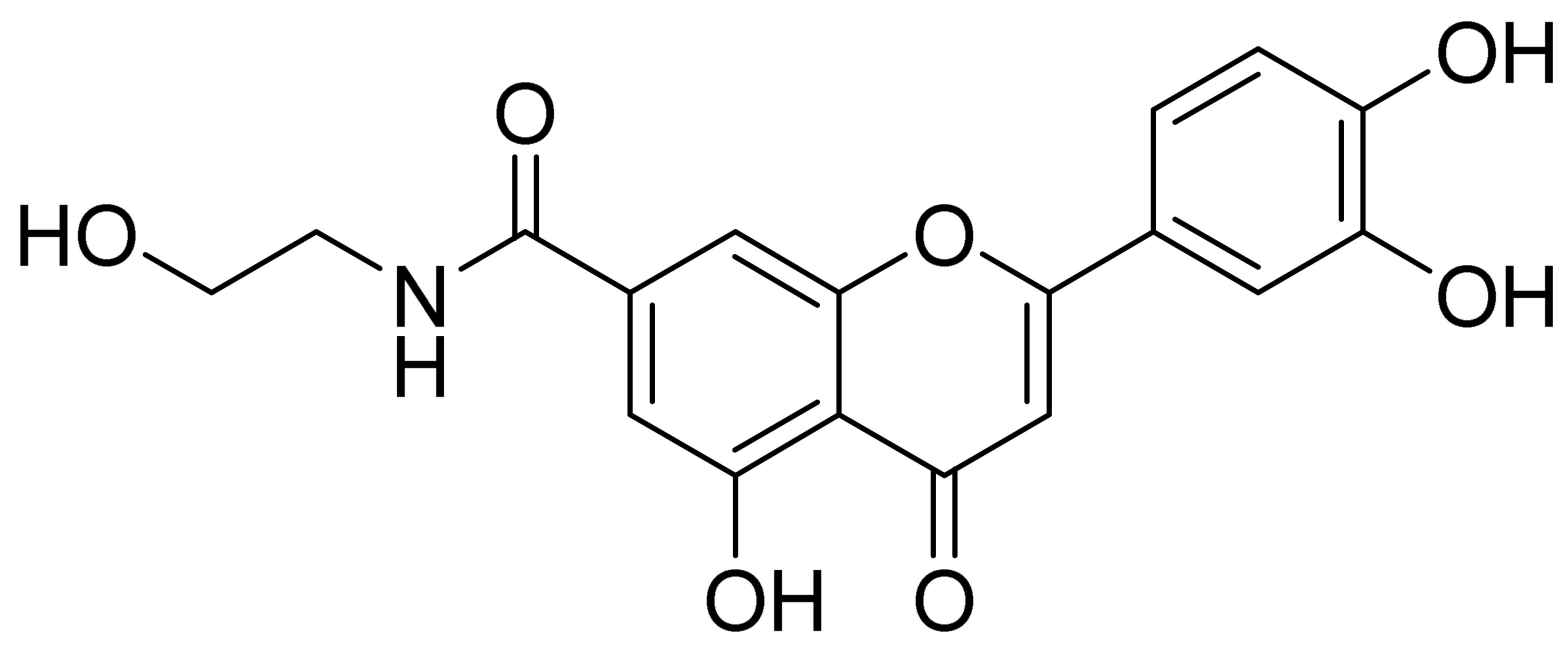	7.6 ± 1.2
**11**	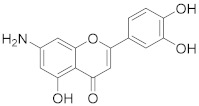	1.3 ± 0.2
**12**	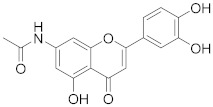	3.0 ± 0.4
**14**	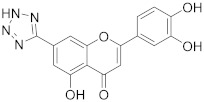	1.4 ± 0.3
**15**	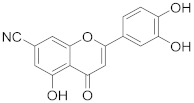	1.3 ± 0.3
**17**	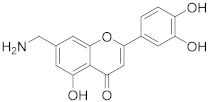	6.7 ± 0.8
**18**	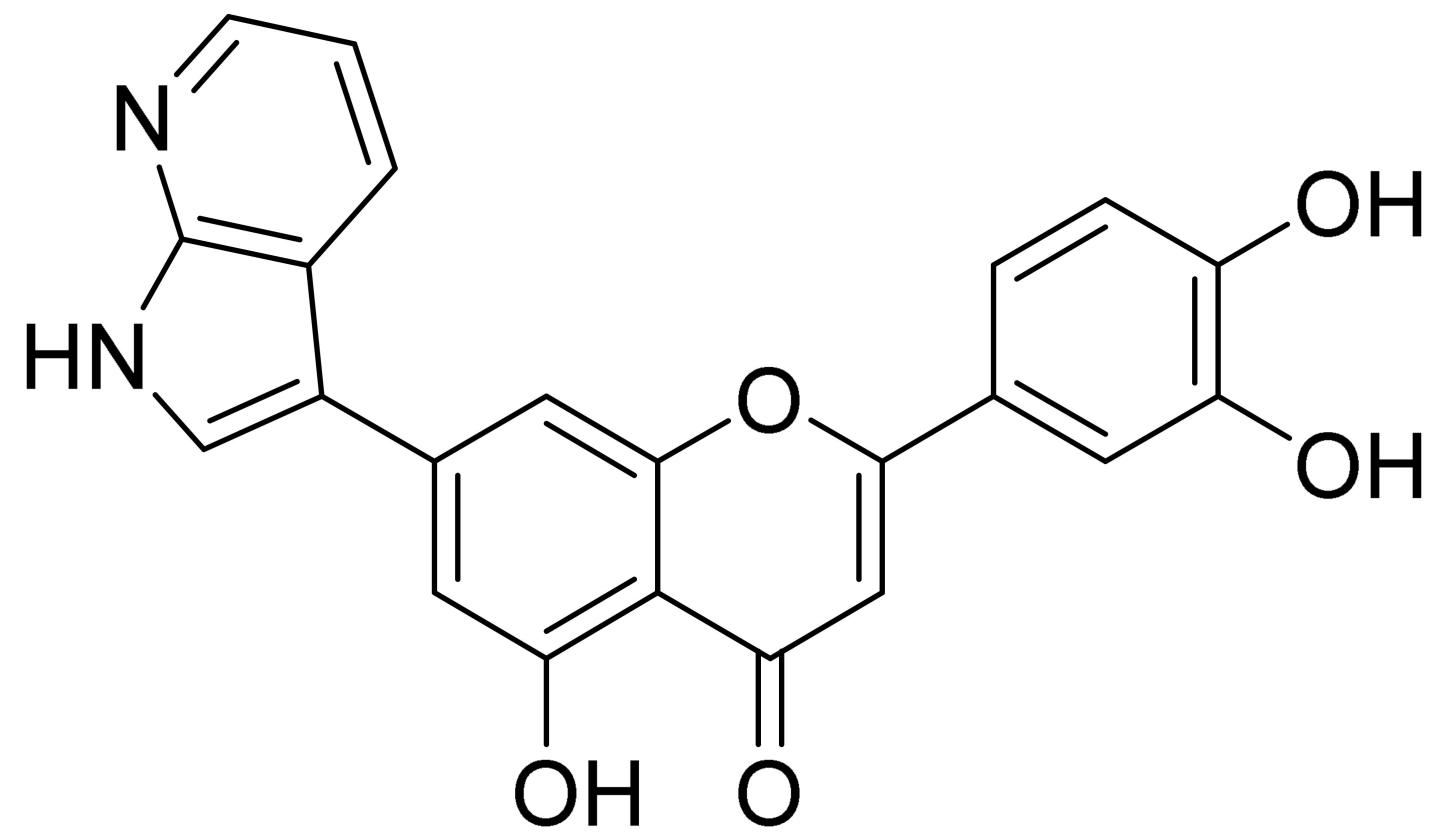	3.4 ± 0.6
**19**	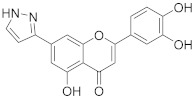	0.81 ± 0.12
**20**	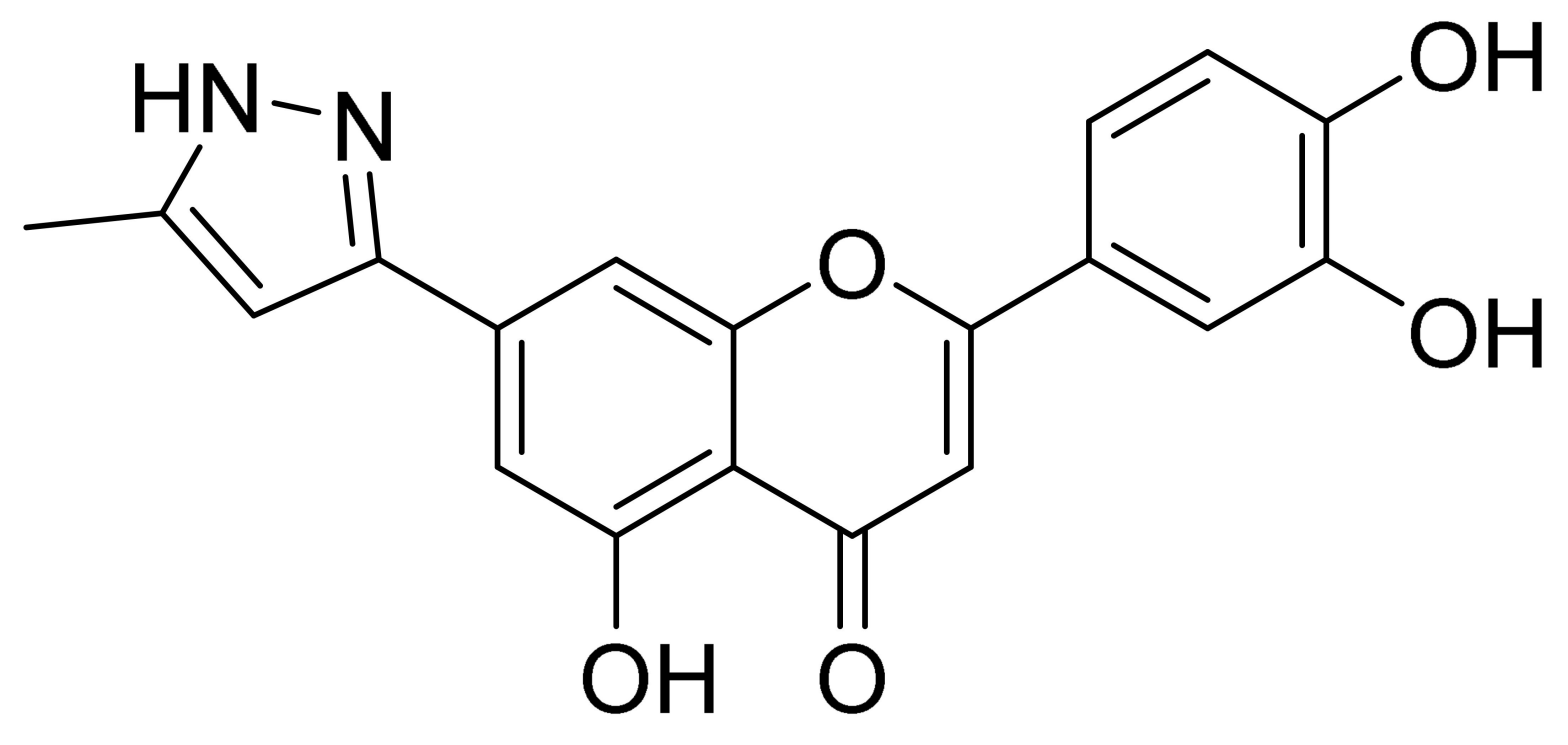	2.6 ± 0.3
cynaroside	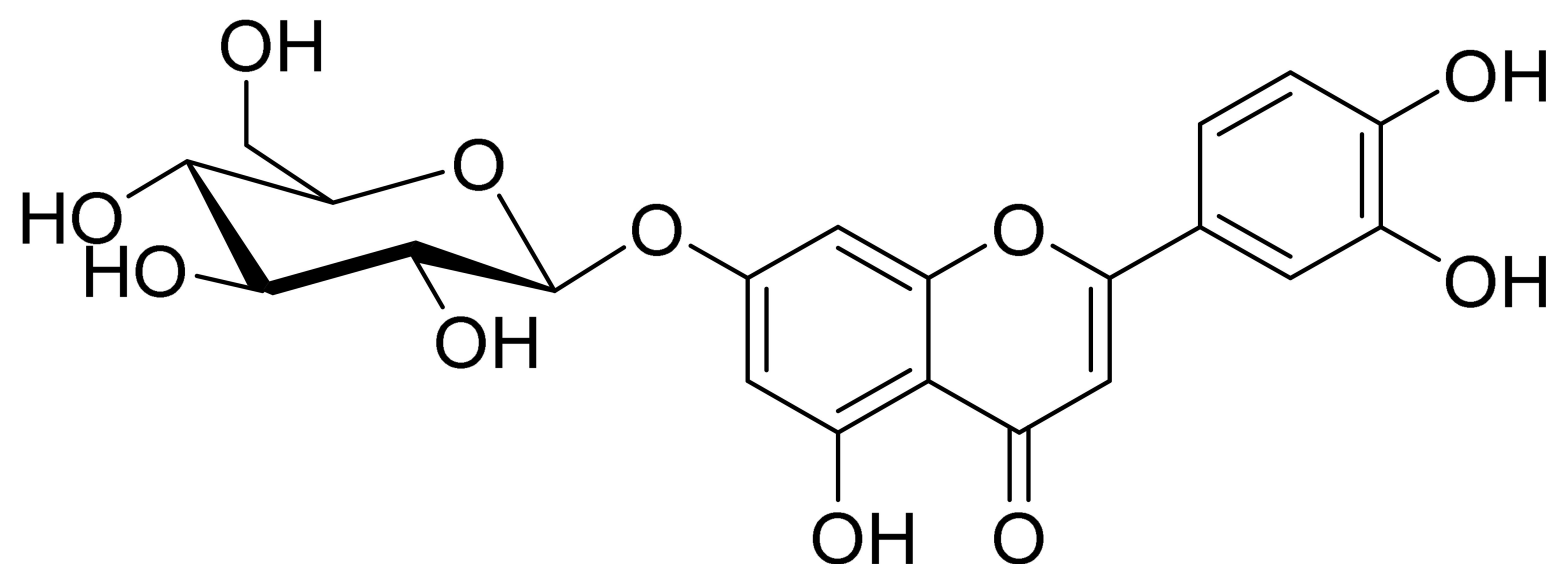	32 ± 3

**Table 2:** Inhibition assay of influenza endonuclease. Structure–activity relationship of C-8 luteolin derivatives.

Compound	Structure	IC_50_ (μM) (AlphaScreen)
orientin	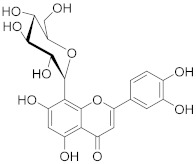	0.042 ± 0.002
**21**	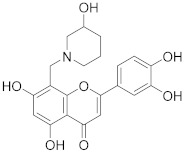	0.083 ± 0.009
**22**	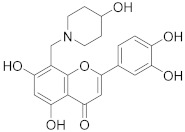	0.14 ± 0.01
**23**	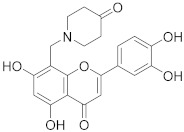	0.077 ± 0.013
**24**	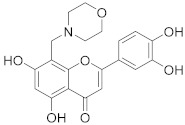	0.054 ± 0.009
**25**	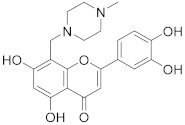	0.066 ± 0.009
**26**	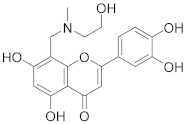	1.2 ± 0.2
**27**	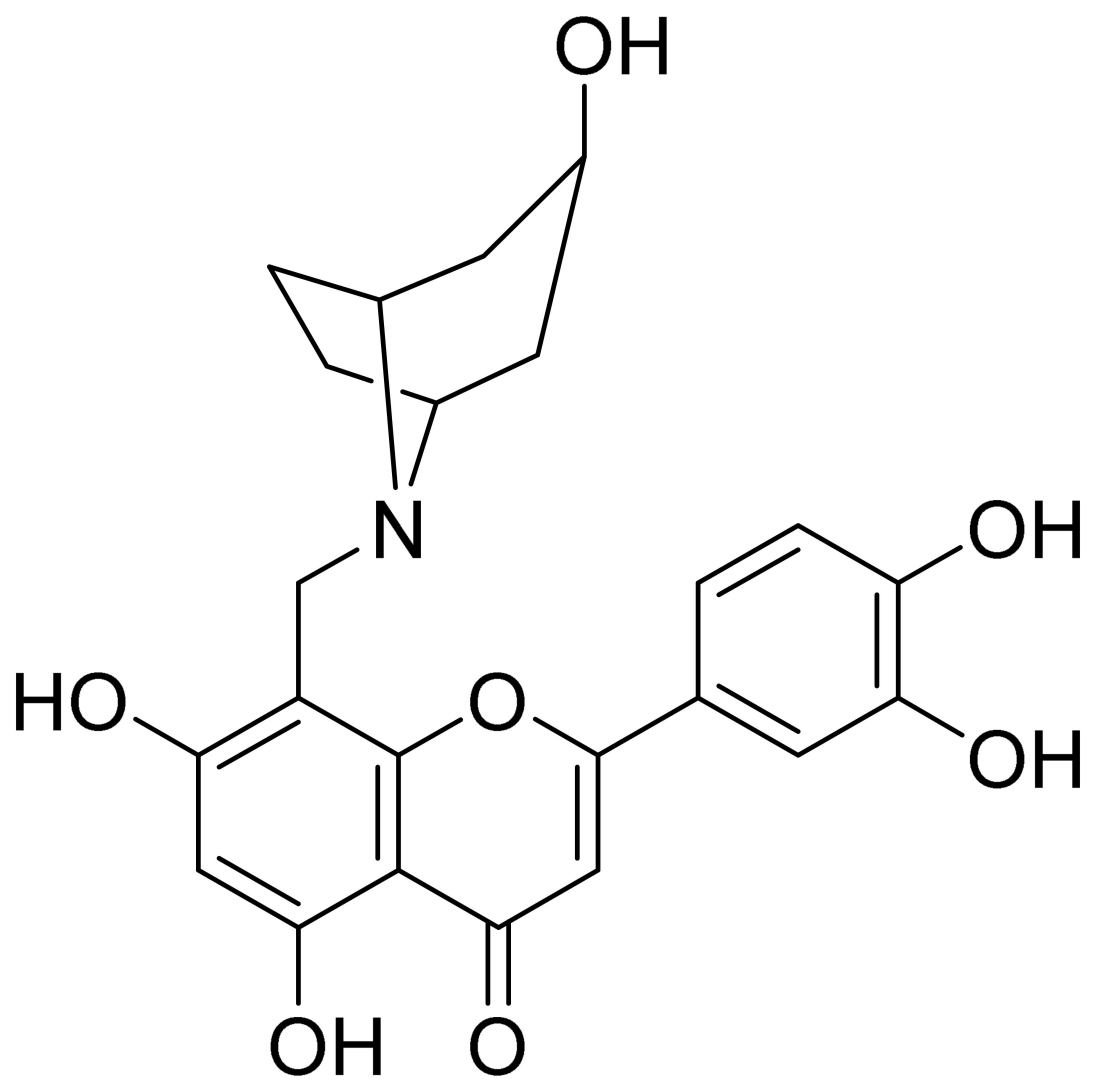	0.075 ± 0.015
**28**	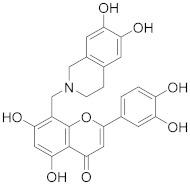	0.12 ± 0.02
**29**	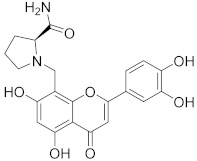	0.073 ± 0.012
**30**	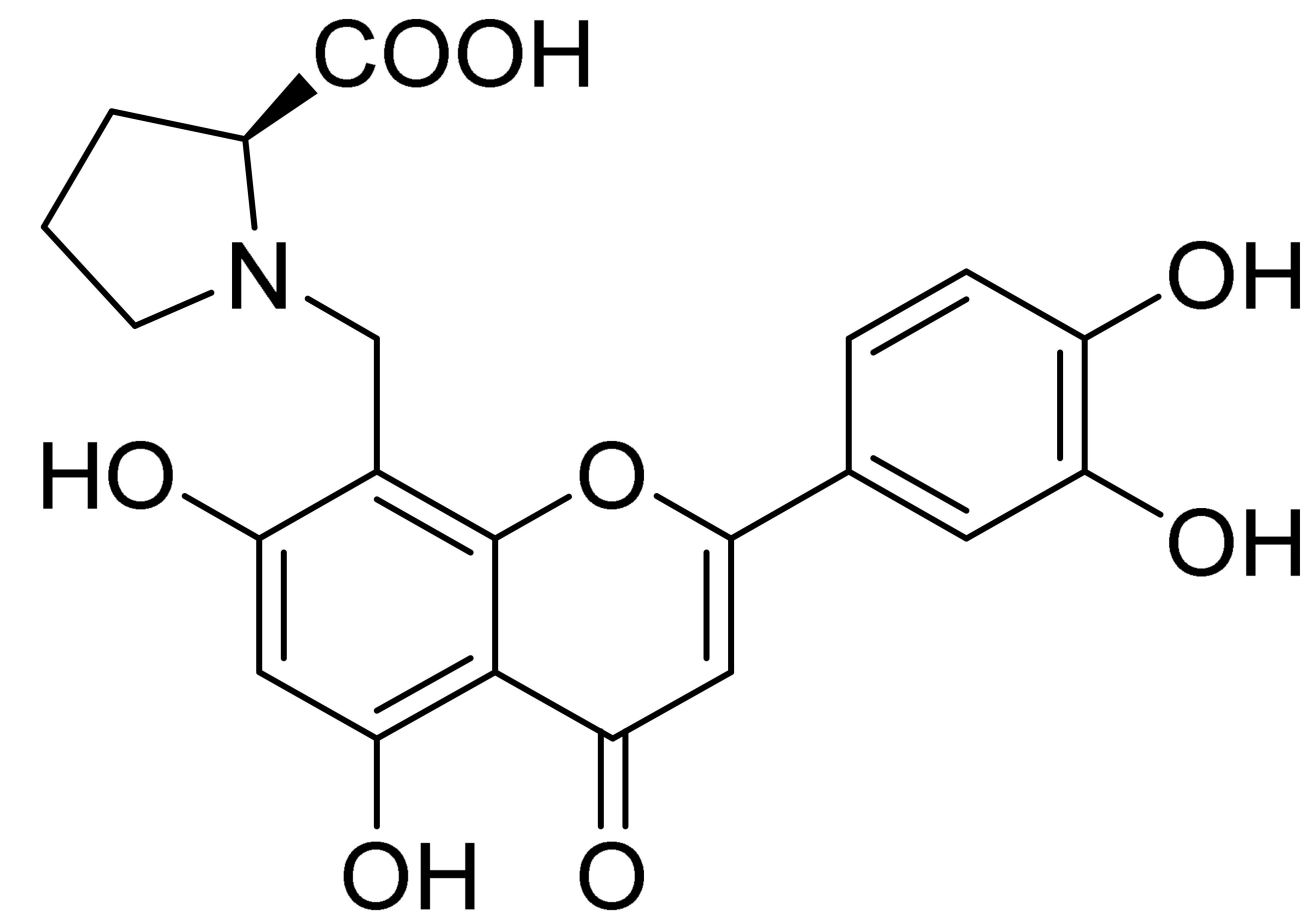	0.078 ± 0.013
**31**	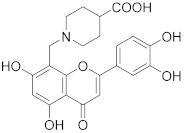	0.080 ± 0.022
**32**	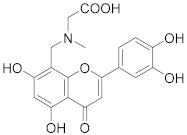	0.074 ± 0.008

## Data Availability

The data presented in this study are available in the article.

## Supplementary Materials

Supplementary materials can be found at https://www.mdpi.com/article/10.3390/ijms22147735/s1.
