# Supplementary material for: Synthesis and In Vitro Evaluation of C-7 and C-8 Luteolin Derivatives as Influenza Endonuclease Inhibitors
Source: Int J Mol Sci. 2021 Jul 20;22(14):7735. doi: 10.3390/ijms22147735 (PMC8305651; doi:10.3390/ijms22147735)
Supplement: Supplementary file 1 [file ijms-22-07735-s001.zip › ijms-1255495-supplementary.pdf]

International Journal of *Molecular Sciences*

MDPI

Supplemental Material to:

Synthesis and *in vitro* Evaluation of C7 and C8 Luteolin Derivatives as Influenza Endonuclease Inhibitors

Róbert Reiberger[1,2†], Kateřina Radilová[1,3†], Michal Kráľ[1,3], Václav Zima[1,2], Pavel Majer[1], Jiří Brynda[1,4], Martin Dračínský[1], Jan Konvalinka[1,5], Milan Kožíšek[1,*] and Aleš Machara[1,*]

[1] Institute of Organic Chemistry and Biochemistry of the Academy of Sciences of the Czech Republic, Gilead Sciences and IOCB Research Center, Flemingovo n. 2, 166 10 Prague 6, Czech Republic; robert.reiberger@uochb.cas.cz (R.R.); katerina.radilova@uochb.cas.cz (K.R.); michal.kral@uochb.cas.cz (M.K.); vaclav.zima@uochb.cas.cz (V.Z.); pavel.majer@uochb.cas.cz (P.M.); jiri.brynda@uochb.cas.cz (J.B.); martin.dracinsky@uochb.cas.cz (M.D.); jan.konvalinka@uochb.cas.cz (J.K.)

[2] Department of Organic Chemistry, Faculty of Science, Charles University, Hlavova 8, 128 00 Prague 2, Czech Republic

[3] First Faculty of Medicine, Charles University, Kateřinská 1660, 121 08 Prague 2, Czech Republic

[4] Institute of Molecular Genetics of the Academy of Sciences of the Czech Republic, Vídeňská 1083, 140 00 Prague 4, Czech Republic

[5] Department of Biochemistry, Faculty of Science, Charles University, Hlavova 8, 128 00 Prague 2, Czech Republic

[†] These authors contributed equally to this work

[*] Correspondence: milan.kozisek@uochb.cas.cz (M.K.); ales.machara@uochb.cas.cz (A.M.); Tel.: +420-220-183-479 (A.M.)

Table of Contents

AlphaScreen titration curves 3

Gel-based cleavage assay – wild type 4

Gel-based cleavage assay – comparison of wild type with I38T 6

ssDNA and ssRNA cleavage efficiency of PA-Nter WT and I38T mutant 7

Crystallographic data – Table S1 9

Conditions screening of Mannich aminomethylation 11

^{1}H, ^{13}C, ^{19}F and ^{31}P NMR spectra 12

HPLC analysis of final compounds 79

AlphaScreen titration curves

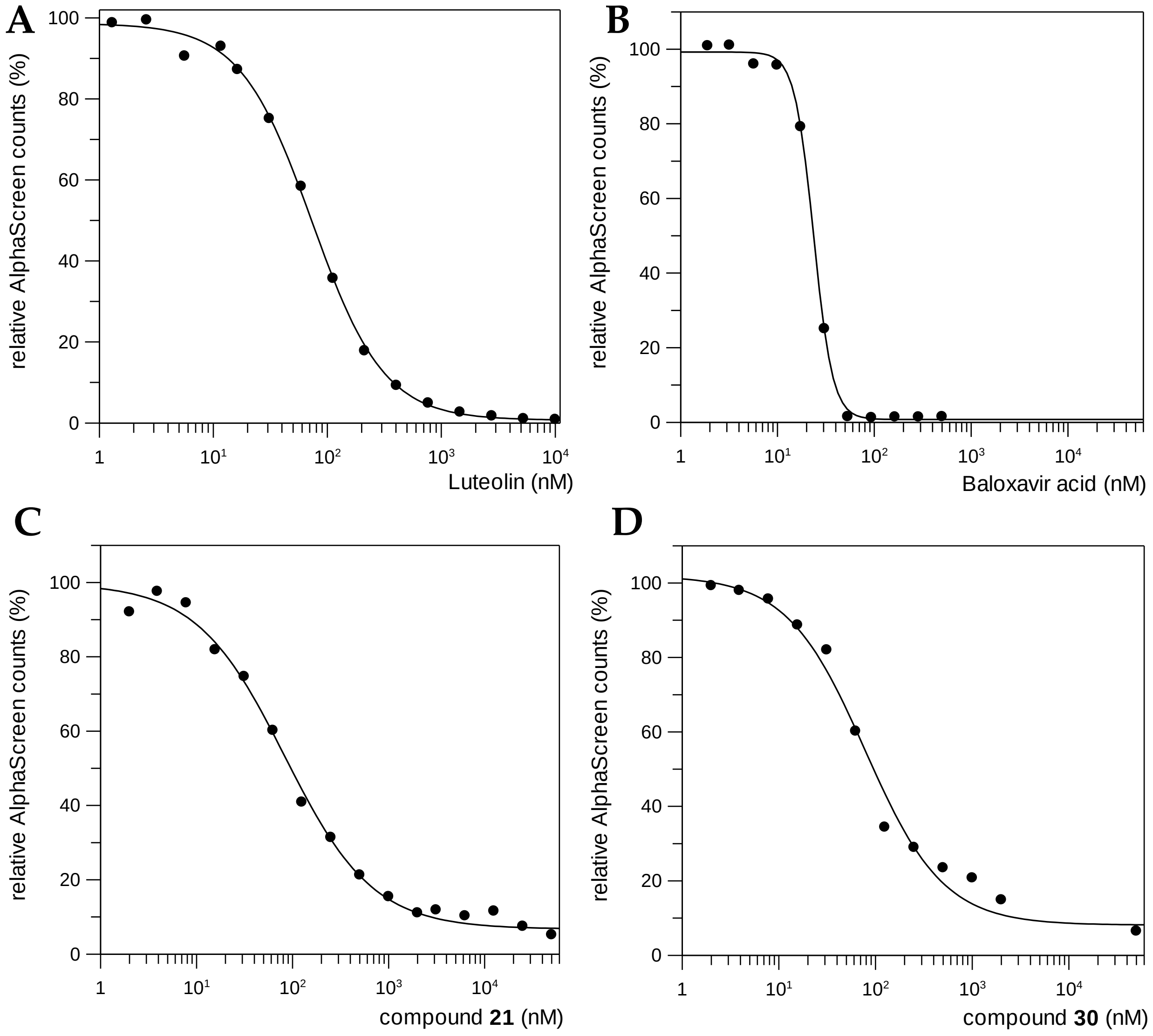

Figure S1. AlphaScreen titration curves of luteolin (A), baloxavir acid (B), compound **21** (C), and compound **30** (D).

Gel-based cleavage assay – wild type

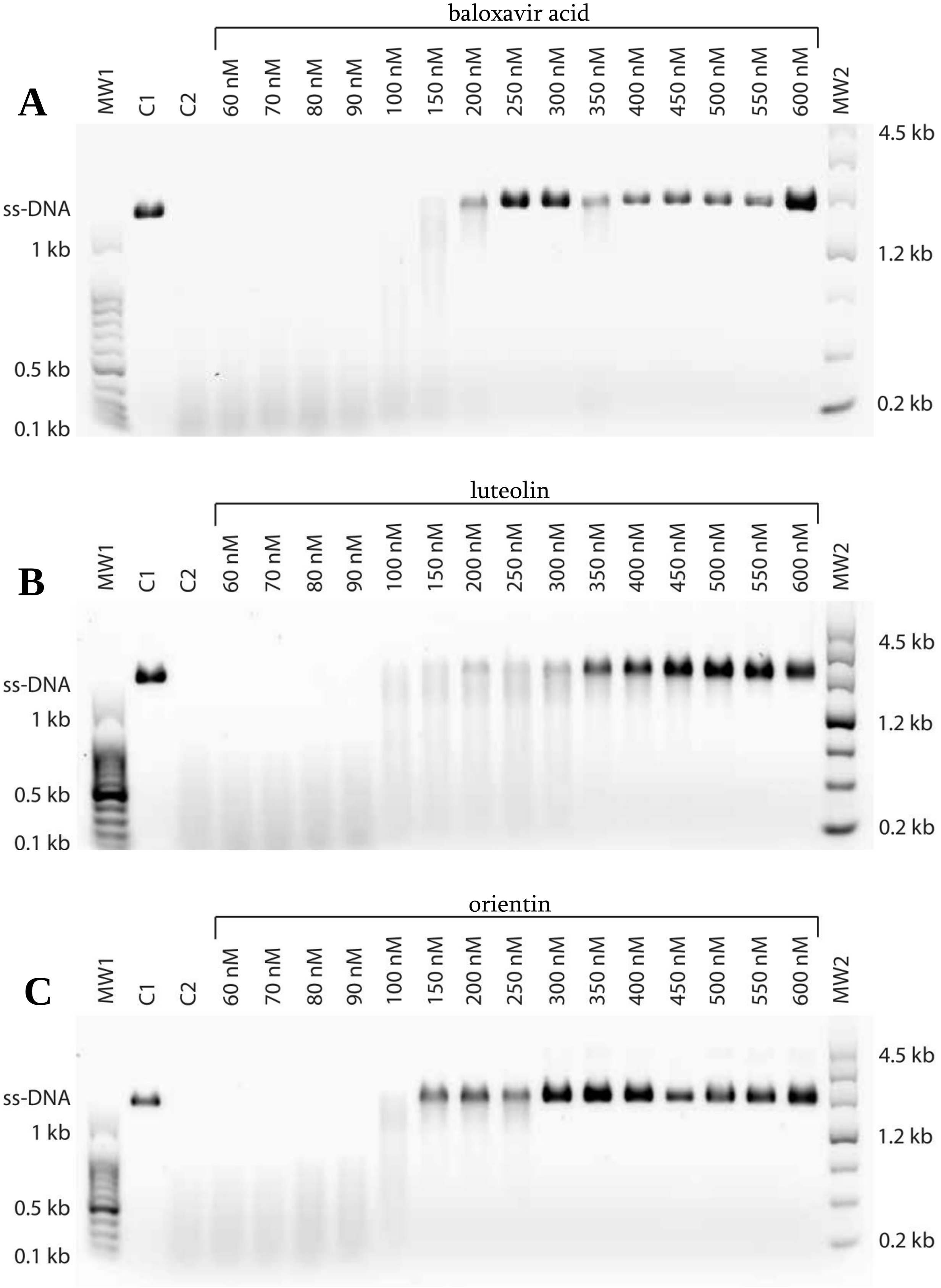

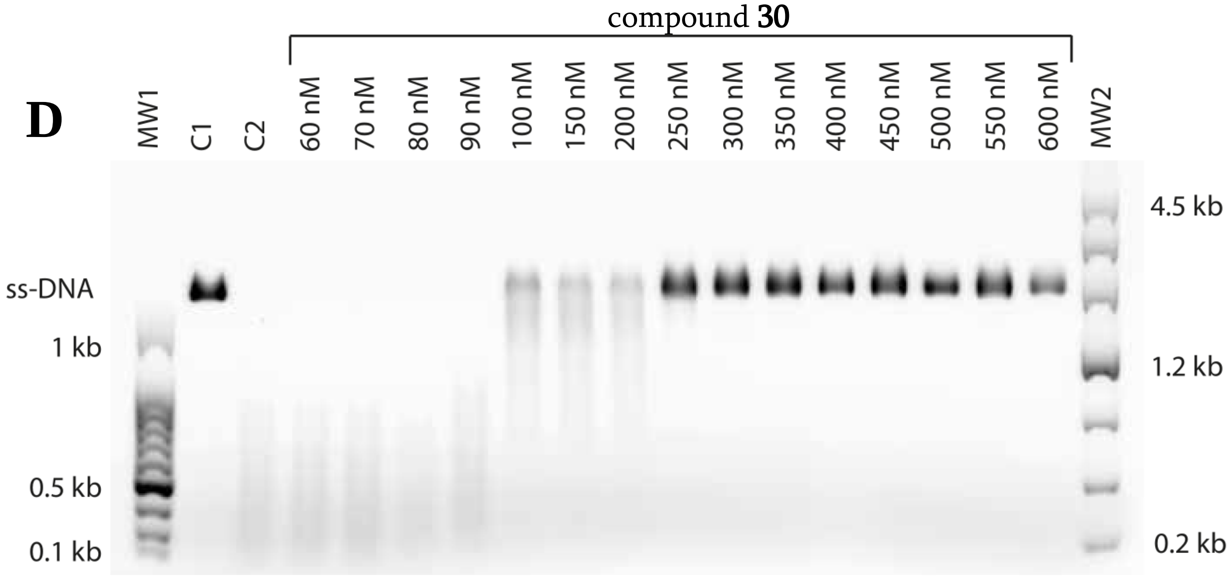

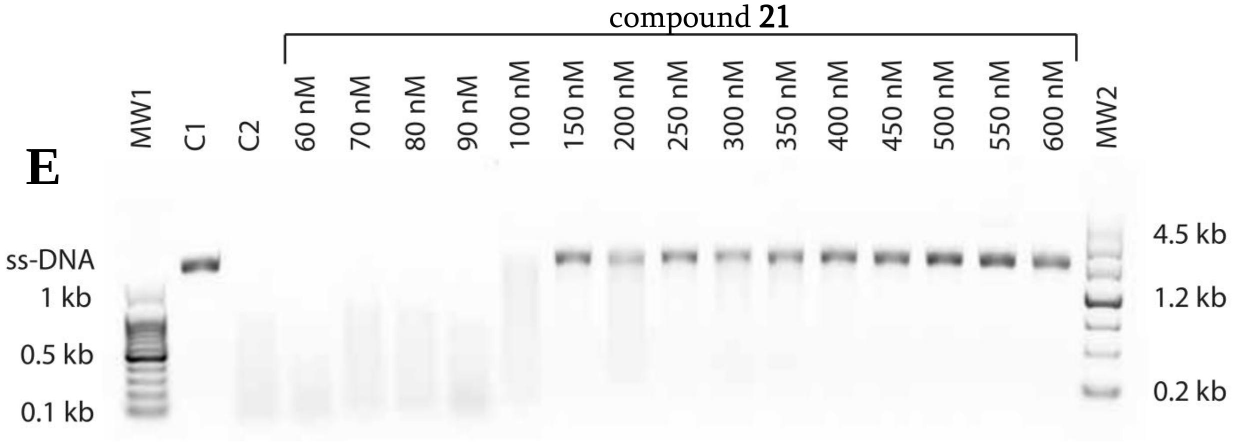

Figure S2. Inhibition of endonuclease activity by of baloxavir acid (A), luteolin (B), orientin (C), compound **30** (D), and compound **21** (E). The single-stranded DNA M13mp18 was used as substrate (lane C1). Fully cleaved substrate without any inhibitor was used as control (lane C2). The GelPilot 100bp Plus Ladder molecular weight marker (lane MW1) and the GelPilot Wide Range Ladder molecular weight marker (lane MW2) are as reference.

Gel-based cleavage assay – comparison of wild type with I38T

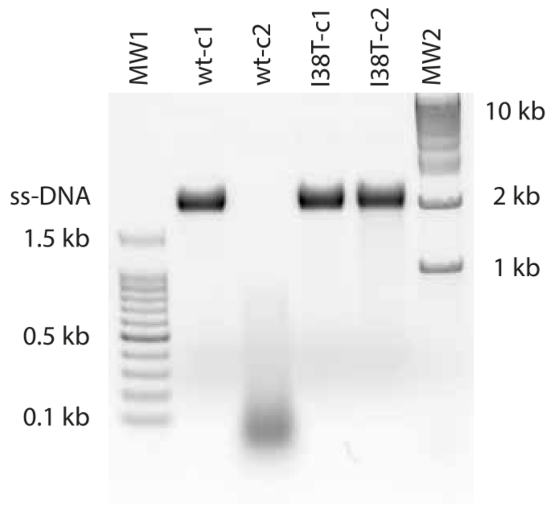

Figure S3. Endonuclease cleavage comparison of wild type and I38T mutant variant. The single-stranded DNA M13mp18 was used as substrate. The uncleaved DNA without endonuclease was used as control (lines wt-c1 and I38T-c1). The overnight cleavage of substrate by 1 μM endonucleases (PA-Nter-wt/PA-Nter-I38T) in the presence of metal ions is shown in lines wt-c2 (for PA-Nter-wt) and I38T-c2 (for PA-Nter-I38T). The GelPilot 100bp Plus Ladder molecular weight marker (lane MW1) and the GelPilot 1 kb Ladder molecular weight marker (lane MW2) are as reference.

ssDNA and ssRNA cleavage efficiency of PA-Nter WT and I38T mutant

To detect the endonuclease activity of the PA-Nter WT and I38T, FRET-based endonuclease assay was performed in 96-well black microplate and measured with Tecan Infinite M1000 reader (Tecan, Switzerland). Two dual-labelled oligonucleotides with 5′-6-FAM (5′6-carboxyfluorescein) fluorophore and a 3′-BHQ1 quencher (3′-Black Hole Quencher 1) were used as substrates for testing the endonuclease activities and specificities. The substrate S1 of a sequence 5′(6-FAM)-dT-dC-dT-dC-dT-dA-dG-dC-dA-dG-dT-dG-dG-dC-dG-dC-dC-3′(BHQ1) (Generi Biotech, Czech Republic) was used as ssDNA substrate, the substrate S2 of a sequence 5′(6-FAM)-rA-rA-rA-rA-rA-rA-rA-rA-rA-rA-rA-rA-3′(BHQ-1) (Integrated DNA Technologies, Inc., USA) was used as ssRNA substrate. Cleavage of the oligonucleotide substrate liberates the 6-FAM fluorophore from the BHQ1 quencher resulting in an increase of the fluorescent signal. FRET-based endonuclease assay was performed by incubating 1 μM GST-PA-Nter (WT or I38T mutant) with 1 μM substrate at 37°C in a final volume of 40 μl in reaction buffer 25 mM Tris-HCl, pH 7.4, 150 mM NaCl, 0.05% Tween20, 1 mM $MnCl_2$, 10 mM $MgCl_2$, 1 mM 2-mercaptoethanol. Consequent to the endonuclease cleavage the fluorescent signal was measured at the wavelength of 485 nm excitation and 535 nm emission.

The PA-Nter WT enzyme cleaved both ssRNA and ssDNA substrates, with higher efficiency to the ssRNA sequence. Implementation of the I38T mutation caused change in substrate selectivity, where only the ssRNA substrate was cleaved. This result is in agreement with the gel-based cleavage assay, where ssDNA M13mp18 was cleaved only with PA-Nter WT endonuclease (Figure S3). The catalytic efficiency was significantly affected by the mutation, the activity of the mutant endonuclease was only 1.9 % of the wild-type activity (determined as a ratio of slopes of the yellow and red linear curves in Figure S4).

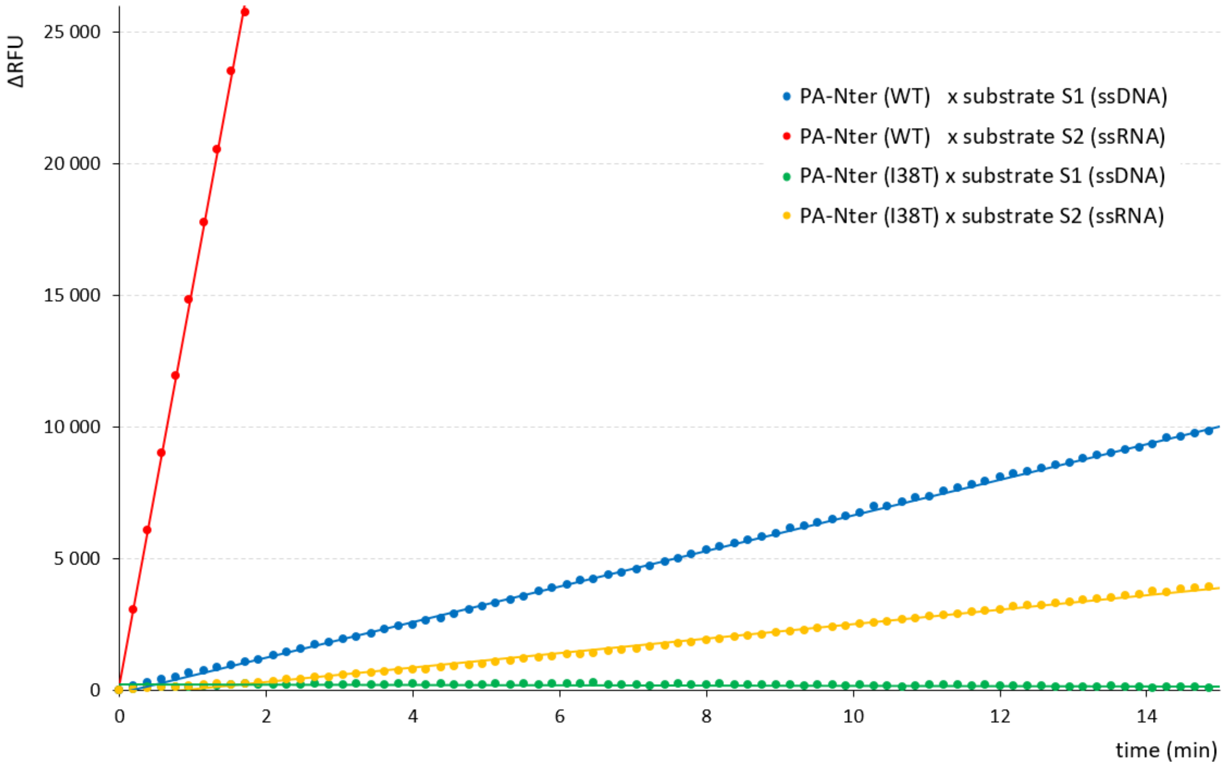

Figure S4. Detection of PA-Nter activity measured by FRET-based endonuclease assay. The enzymes were monitored for their cleavage of ssDNA substrate S1 (WT in blue, I38T in green) or ssRNA substrate S2 (WT in red, I38T in yellow).

Crystallographic data – Table S1

Table S1 – Diffraction data collection and refinement statistics:

	**PA-Nter-wt**	**PA-Nter-I38T**
**Data collection statistics**		
Wavelength (Å)	1.54187	1.54187
Space group	$P6_422$	$P6_422$
Cell parameters (Å, °)	74.36, 74.36, 126.46, 90.0, 90.0, 120.0	74.97, 74.97, 127.57, 90.0, 90.0, 120.0
Resolution range (Å)	50.00-1.90 (2.02-1.90)	50.00-2.20 (2.26-2.20)
Number of unique reflections	16,879 (1,211)	10,985 (770)
Multiplicity	9.3 (6.2)	6.8 (4.2)
Completeness (%)	99.5 (99.3)	90.4 (62.5)
R_{merge}[a]	9.4 (130.9)	5.9 (147.3)
$CC_{(1/2)}$ (%)	99.8 (58.2)	99.2 (98.1)
Average I/σ(I)	15.29 (1.6)	15.96 (1.21)
Wilson B (Å^2)	29.27	41.7
**Refinement statistics**		
Resolution range (Å)	42.15-1.90 (1.95-1.90)	45.24-2.20 (2.26-2.20)
No. of reflection in working set	16,040 (1,150)	10,437 (731)
No. of reflection in the test set	845 (61)	549 (36)
R_{work} value (%)[b]	19.50 (39.2)	19.27 (31.4)
R_{free} value (%)[c]	25.05 (38.9)	26.41 (32.8)
RMSD bond length (Å)	0.01	0.01
RMSD angle (°)	1.5	1.4
Mean ADP value (Å^2)	26.5	42.1
**Ramachandran plot statistics**[d]		
Residues in favored regions (%)	97.8	96.1
Residues in allowed regions (%)	2.2	3.9
PDB code	**7NUG**	**7NUH**

The data in parentheses refer to the highest-resolution shell for data collection statistic.

[a] $R_{merge} = (|I_{hkl} - \langle I \rangle|)/I_{hkl}$, where the average intensity ⟨I⟩ is taken over all symmetry equivalent measurements and I_{hkl} is the measured intensity for any given reflection

[b] R-value = $||F_o| - |F_c||/|F_o|$, where F_o and F_c are the observed and calculated structure factors, respectively.
[c] R_{free} is equivalent to R-value but is calculated for 5% of the reflections chosen at random and omitted from the refinement process.[1]
[d] As determined by Molprobity.[2]

---

[1] Murshudov, G. N.; Skubak, P.; Lebedev, A. A.; Pannu, N. S.; Steiner, R. A.; Nicholls, R. A.; Winn, M. D.; Long, F.; Vagin, A. A., REFMAC5 for the refinement of macromolecular crystal structures. *Acta Crystallographica Section D-Structural Biology* **2011,** *67*, 355-367.
[2] Chen, V. B.; Arendall, W. B.; Headd, J. J.; Keedy, D. A.; Immormino, R. M.; Kapral, G. J.; Murray, L. W.; Richardson, J. S.; Richardson, D. C., MolProbity: all-atom structure validation for macromolecular crystallography. *Acta Crystallographica Section D-Structural Biology* **2010,** *66*, 12-21.

Conditions screening of Mannich aminomethylation

Table S2 – Conditions screening of Mannich aminomethylathion of luteolin with 3-hydroxypiperidine:

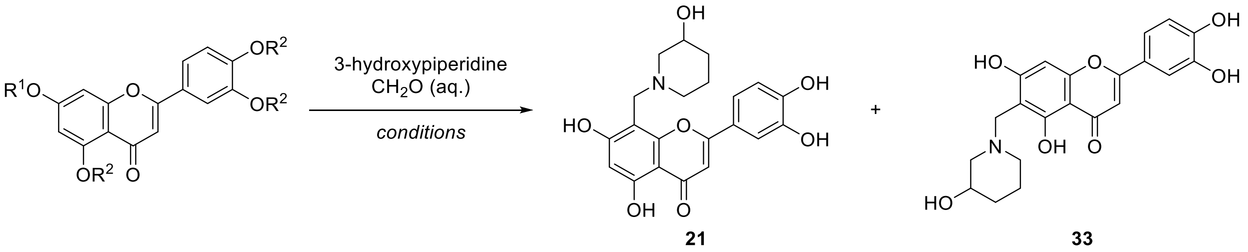

entry	R¹	R²	conditions	amine (eq.)	formalin (eq.)	*T* (°C)	conversion[a] (%)	yield **21** (%)	ratio[a] (**21**:**33**)
**1**	H	H	EtOH, 16 h	1.0	1.0	r.t	58	4[a]	82:18
**2**	H	H	MeOH, 16 h	1.0	1.0	r.t	86	20[b]	44:26
**3**	H	H	dmap, dioxane, 4 h	1.2	1.2	100	-	0[a,d]	N/A
**4**	H	H	dmap, *i*-PrOH	1.2	1.2	70	-	0[a,d]	N/A
**5**	H	H	*i*-PrOH, 5 d	1.5	0.8	35	0	0[a,c]	N/A
**6**	H	Ac	DMF/MeOH (4:1), 14 h	2.0	2.0	r.t	-	0[a,d]	N/A
**7**	Ac	Ac	1. $CHCl_3$, 36 h<br>2. 2M LiOH (aq.), MeOH	2.0	2.0	r.t<br>r.t	39<br>100	14[b]	95:5
**8**	Ac	Ac	1. $CHCl_3$, *p*-TSA, 4 d<br>2. 2M LiOH (aq.), MeOH	1.5	1.5	r.t<br>-	0<br>-	0[a,c]	N/A
**9**	Bn	Bn	$CHCl_3$, 2 d	2.0	2.0	r.t	0	0[a,c]	N/A
**10**	MOM	MOM	EtOH, 16 h	1.0	1.0	r.t	0	0[a,c]	N/A
**11**	MOM	MOM	$CHCl_3$, 36 h	2.0	2.0	r.t	0	0[a,c]	N/A

a) Determined by UHPLC-MS. **b)** Yield obtained by multiple preparative HPLC isolation. **c)** No reaction. **d)** Full decomposition of starting material was observed. N/A = not applicable

¹H, ¹³C, ¹⁹F and ³¹P NMR spectra

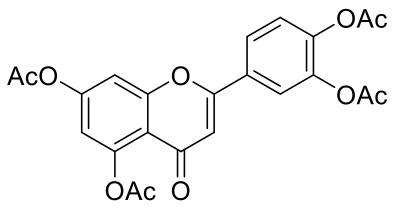

1

^{1}H NMR, 400 MHz, 298 K, $CDCl_3$

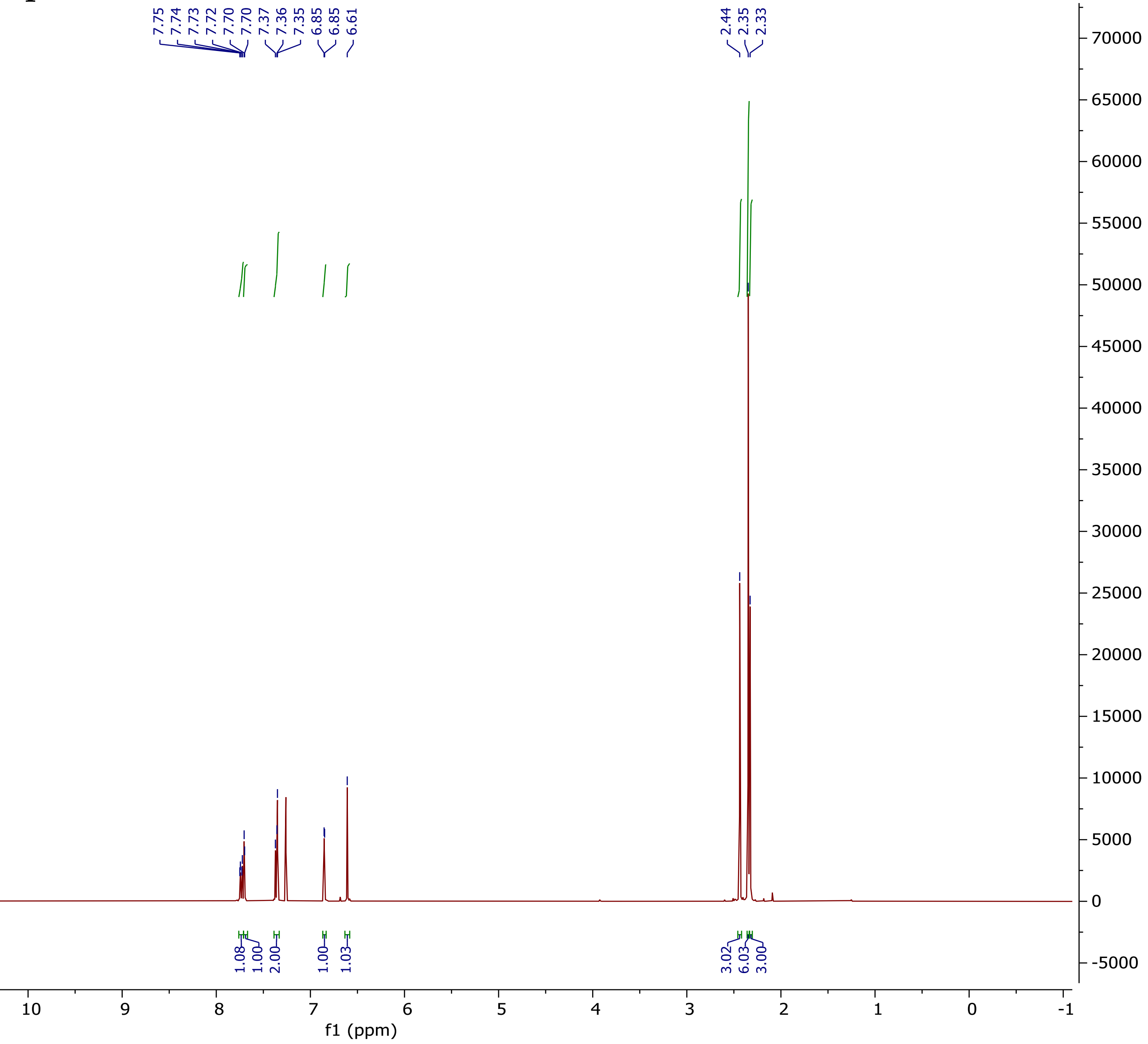

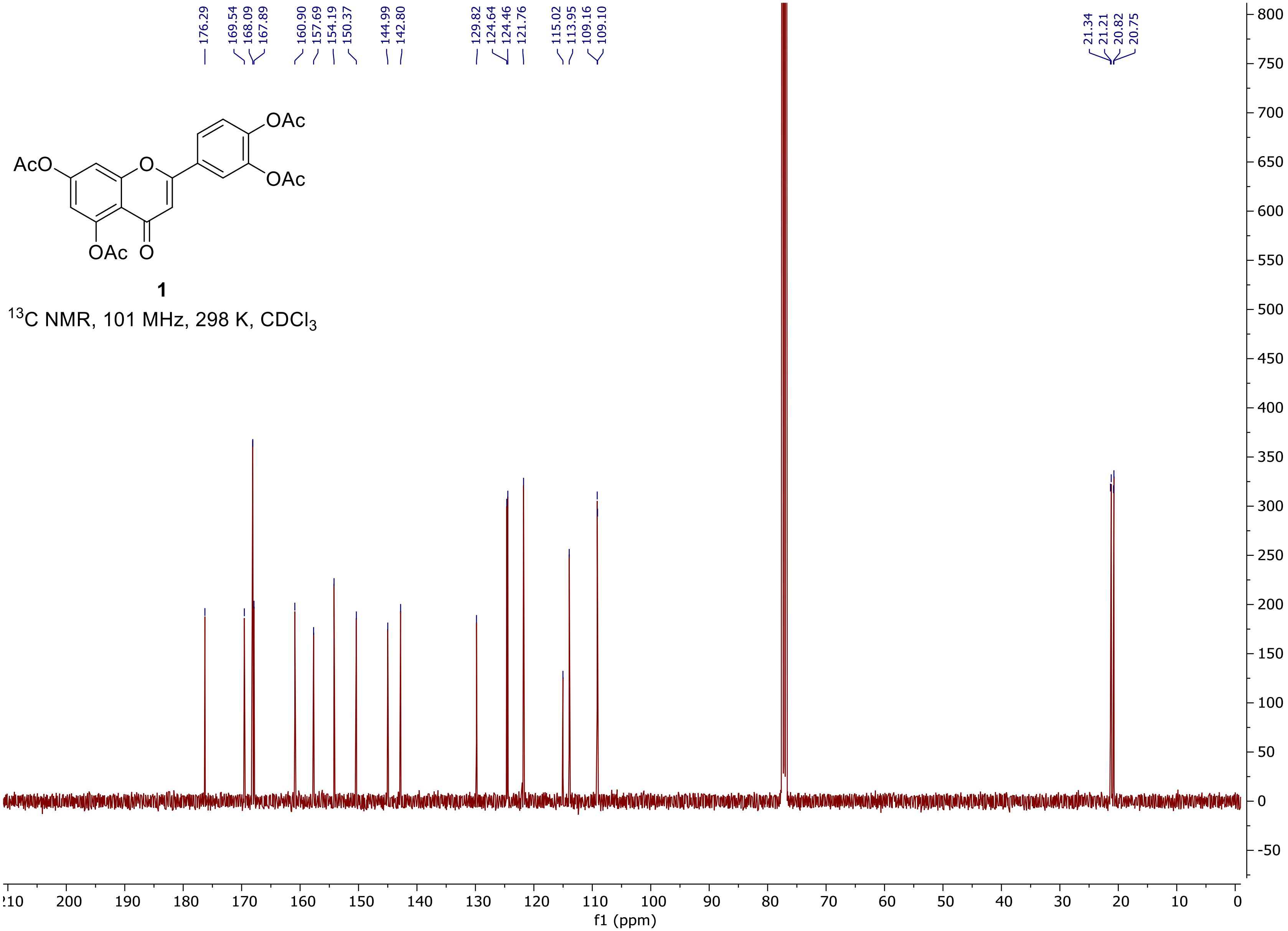

1

^{13}C NMR, 101 MHz, 298 K, $CDCl_3$

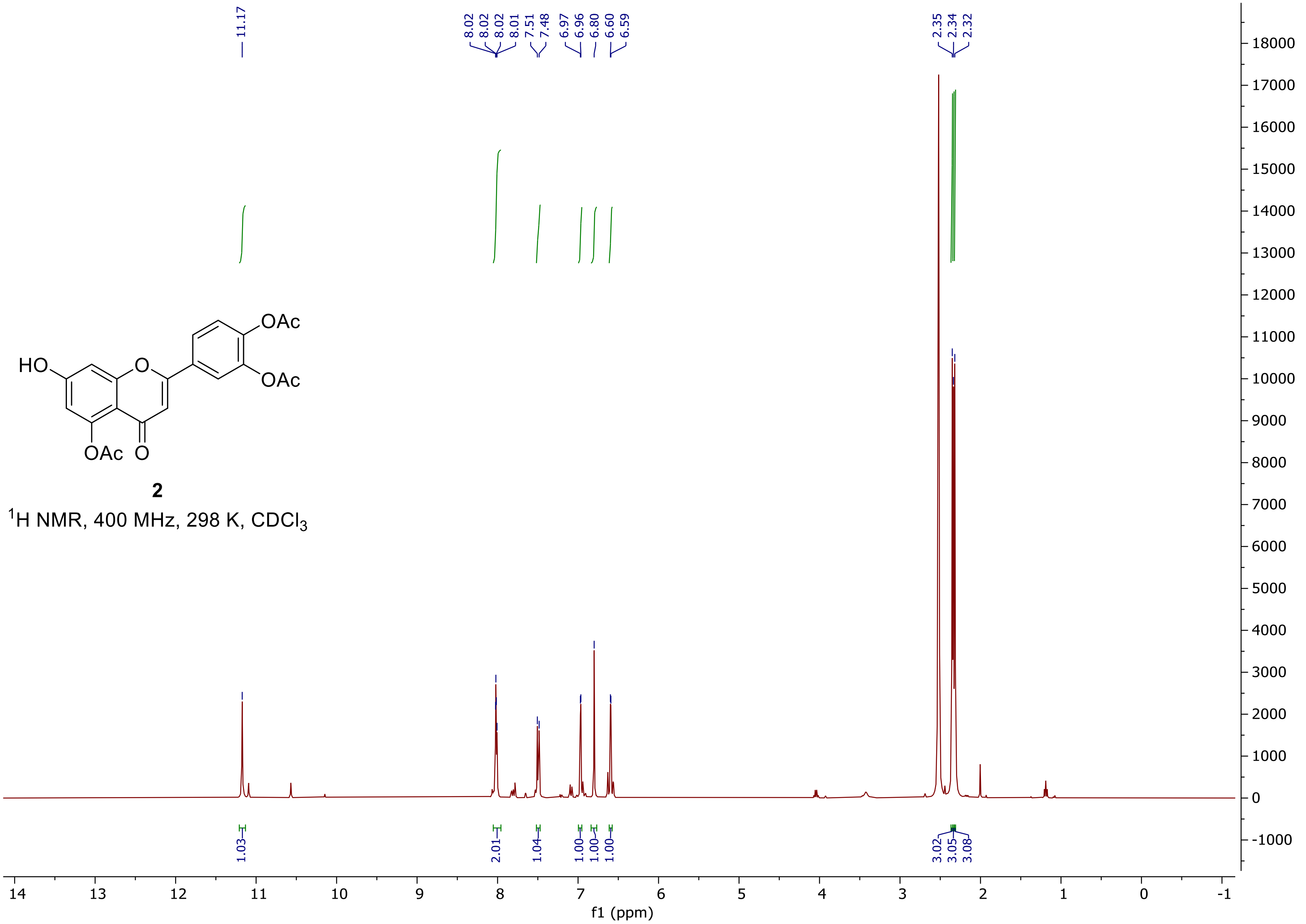

2

^{1}H NMR, 400 MHz, 298 K, $CDCl_3$

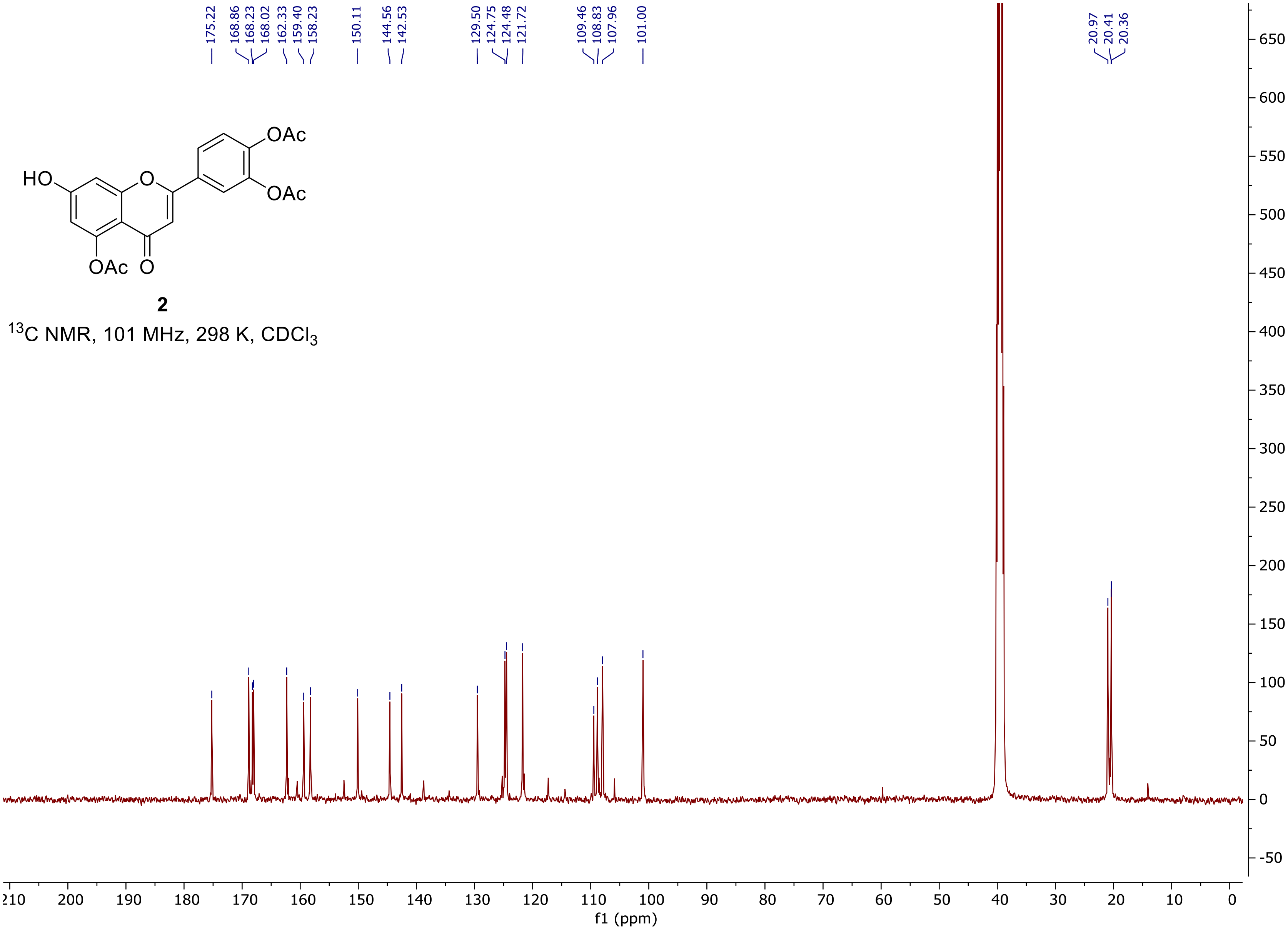

2

^{13}C NMR, 101 MHz, 298 K, $CDCl_3$

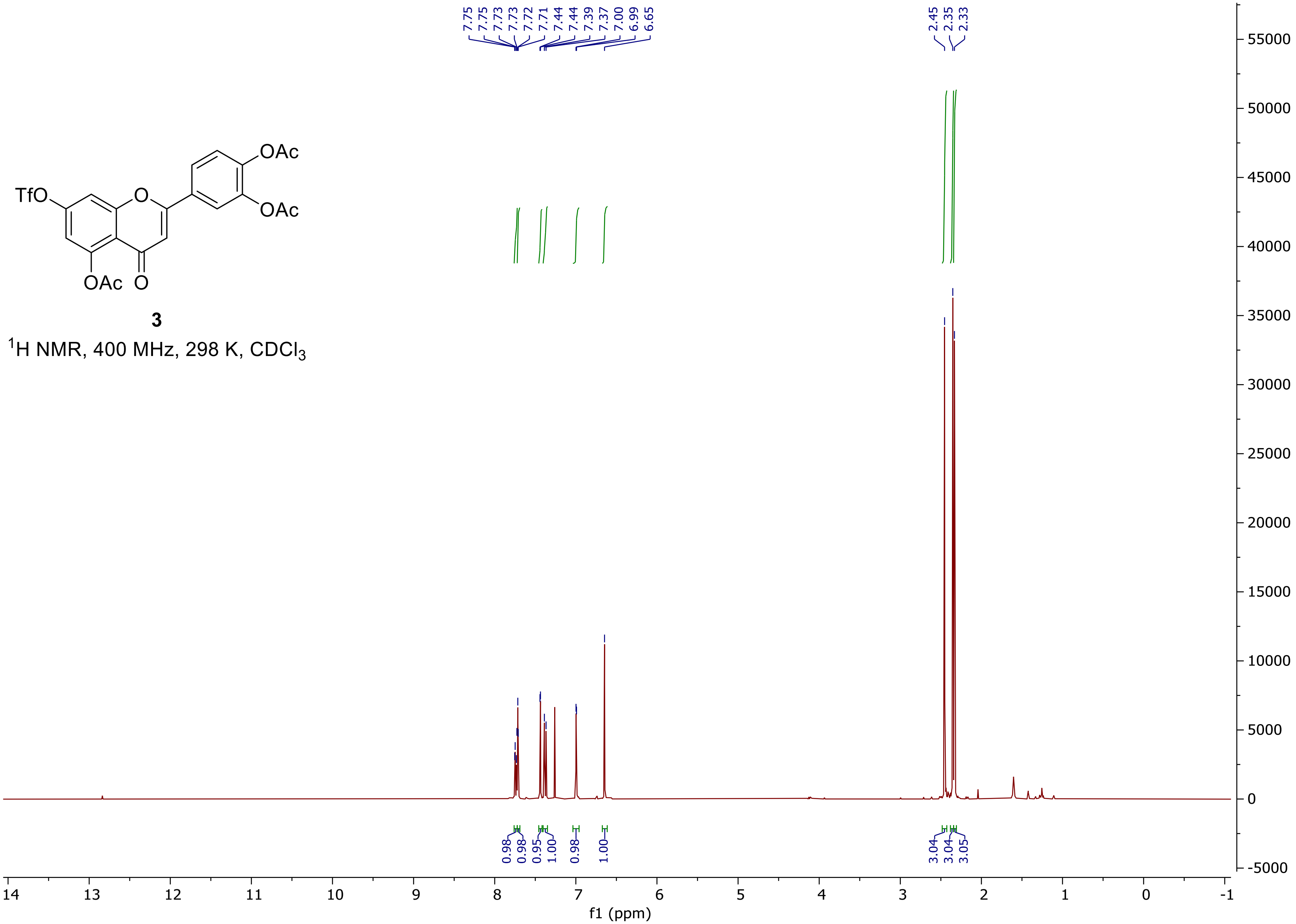
7.75
7.75
7.73
7.73
7.72
7.71
7.44
7.44
7.39
7.37
7.00
6.99
6.65
2.45
2.35
2.33
TfO
O
OAc
OAc
OAc
O
3
1H NMR, 400 MHz, 298 K, CDCl3
0.98
0.98
0.95
1.00
0.98
1.00
3.04
3.04
3.05
f1 (ppm)

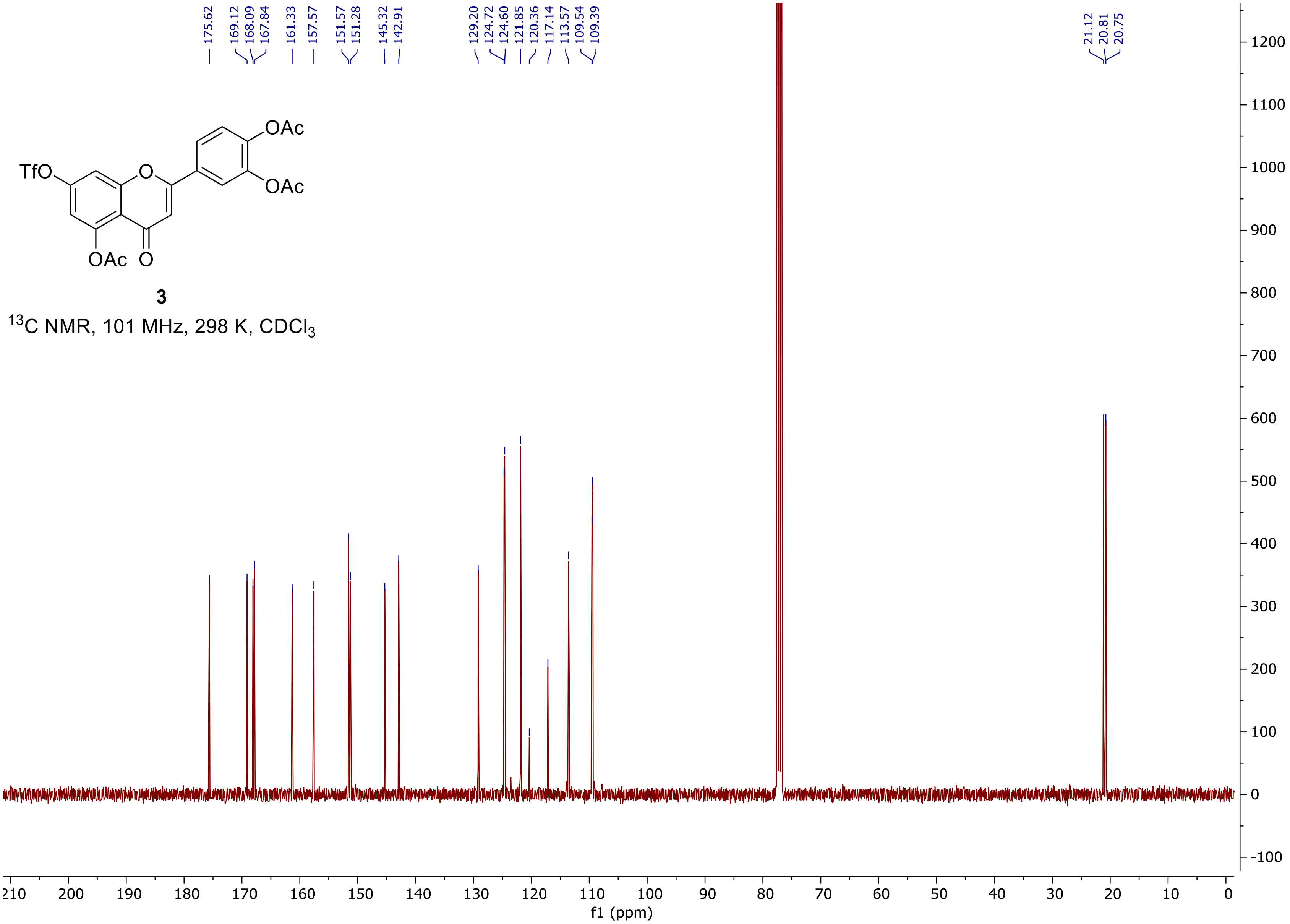

175.62
169.12
168.09
167.84
161.33
157.57
151.57
151.28
145.32
142.91
129.20
124.72
124.60
121.85
120.36
117.14
113.57
109.54
109.39
21.12
20.81
20.75
TfO
O
OAc
OAc
OAc
O
3
^{13}C NMR, 101 MHz, 298 K, CDCl$_3$
f1 (ppm)

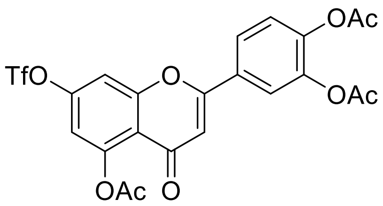

3

^{19}F NMR, 376 MHz, 298 K, $CDCl_3$

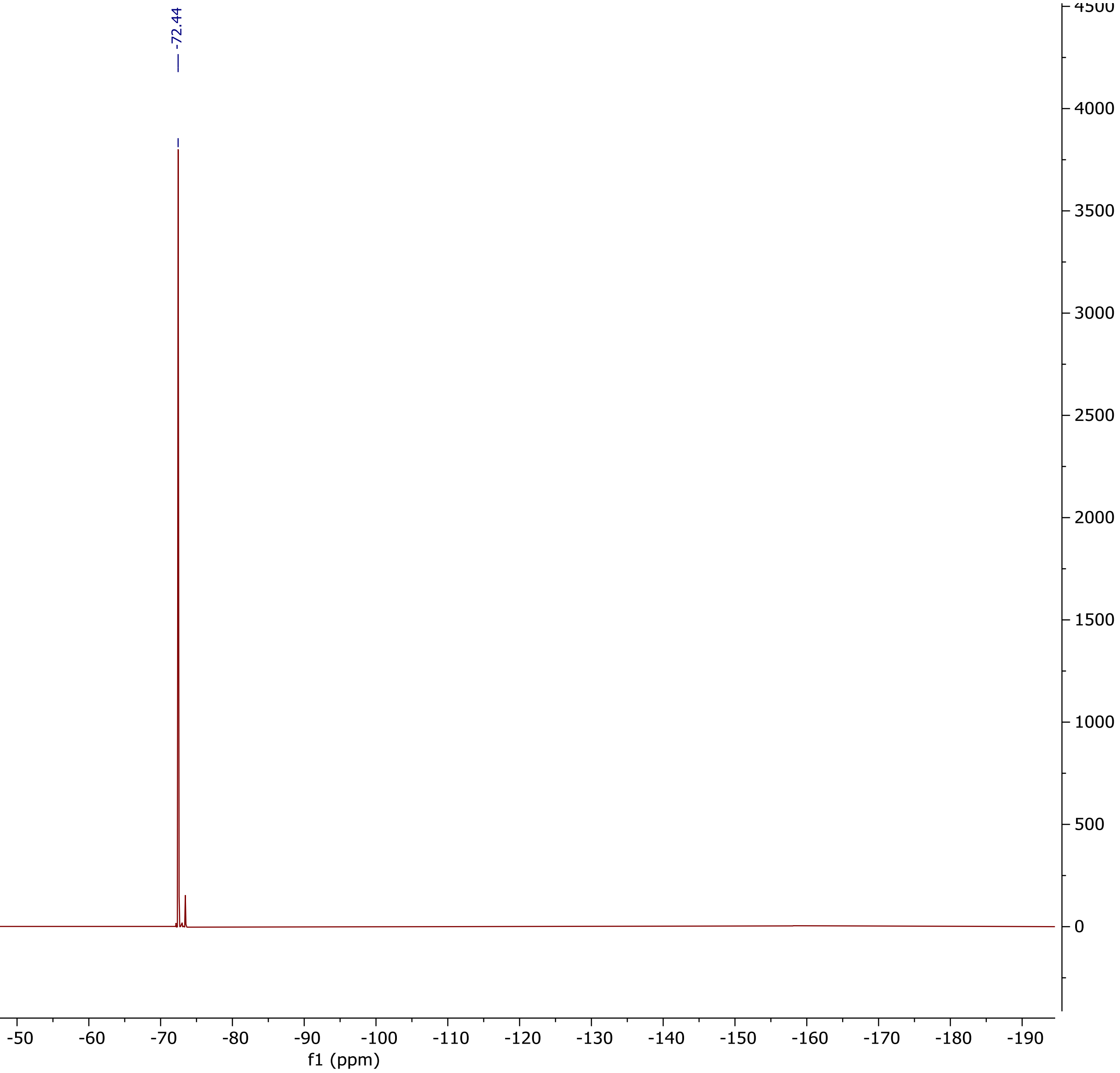

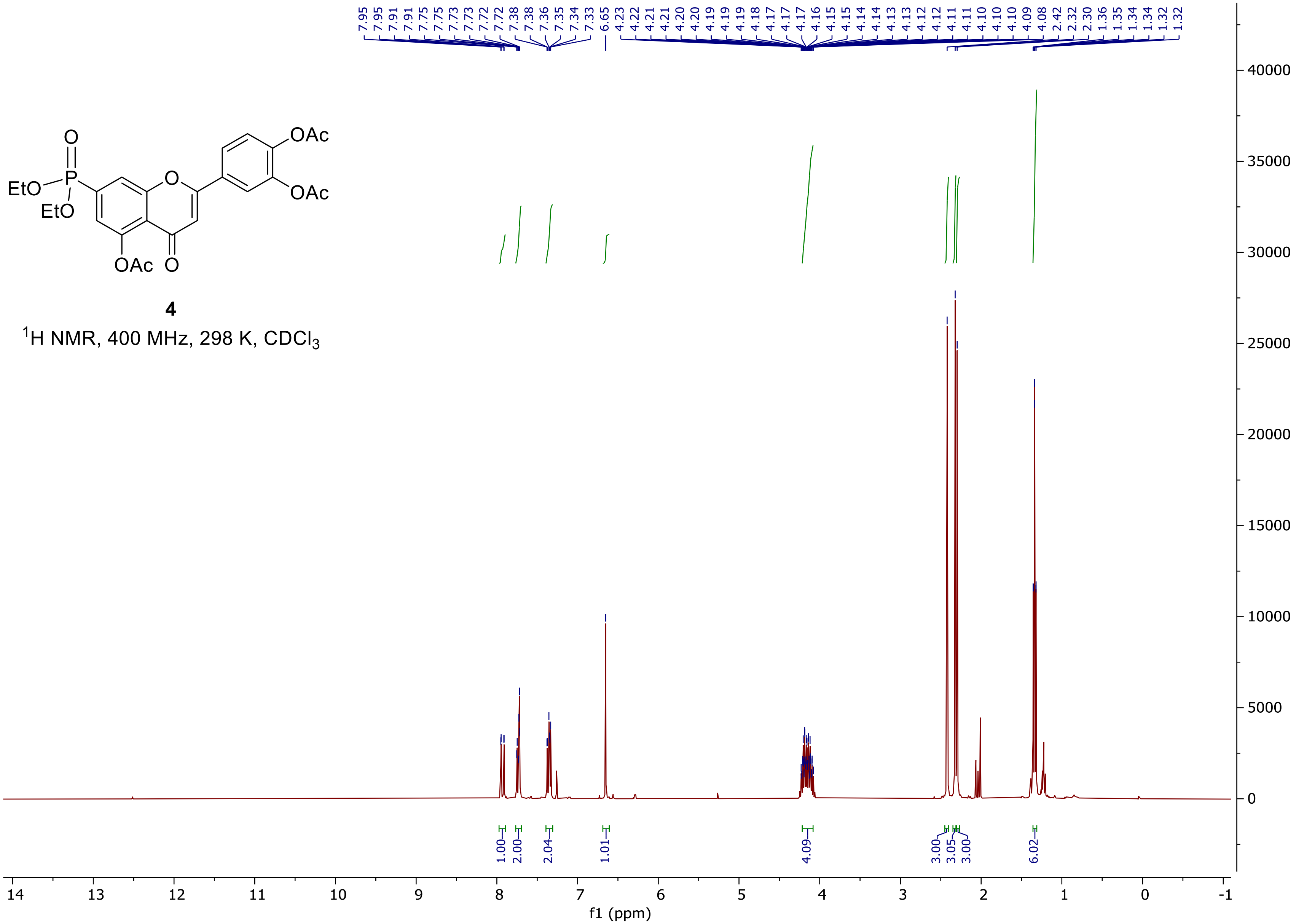

4

^{1}H NMR, 400 MHz, 298 K, $CDCl_3$

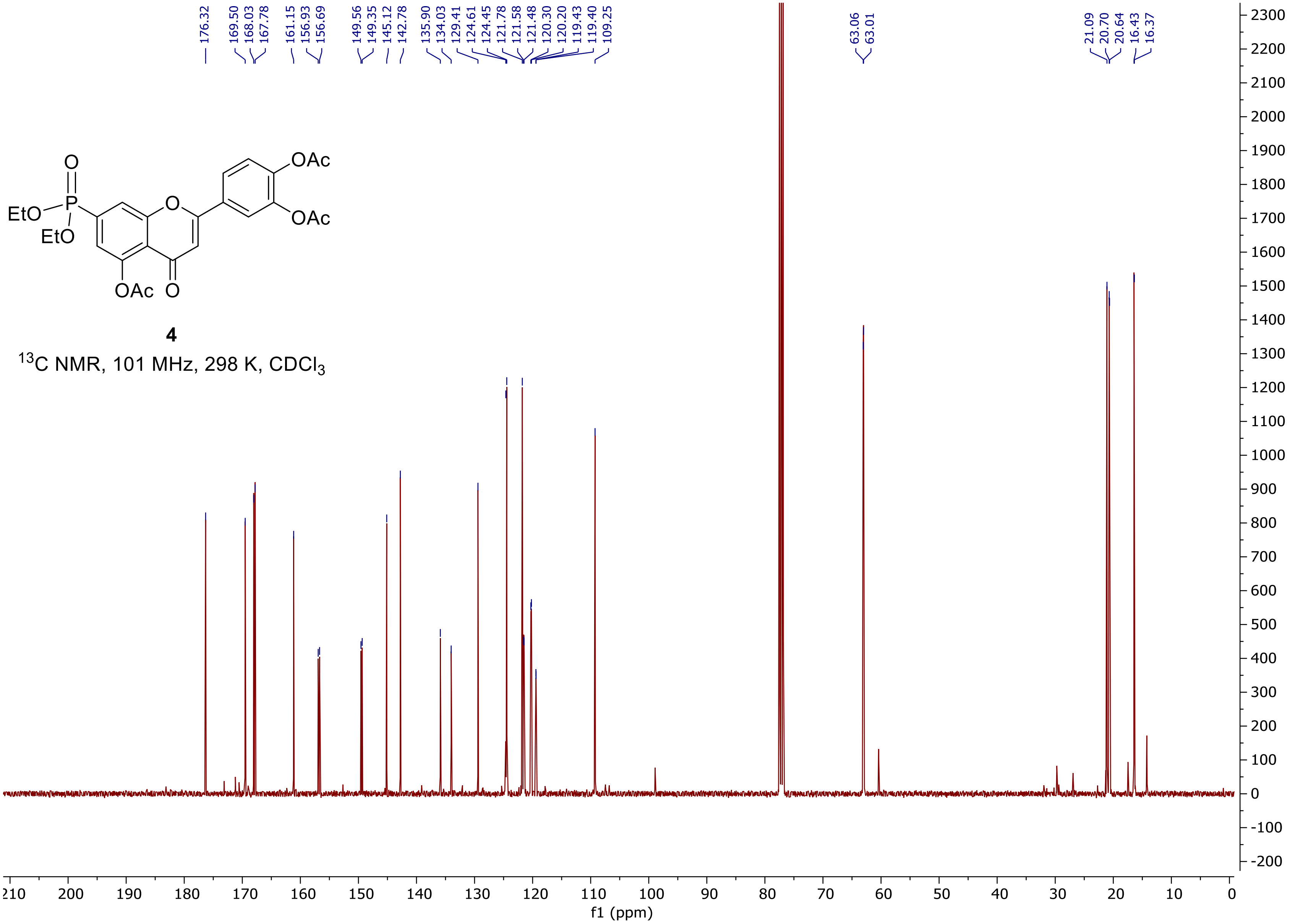

4

^{13}C NMR, 101 MHz, 298 K, $CDCl_3$

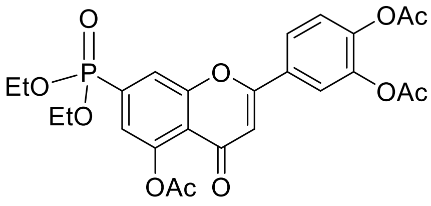

4

^{31}P NMR, 162 MHz, 298 K, $CDCl_3$

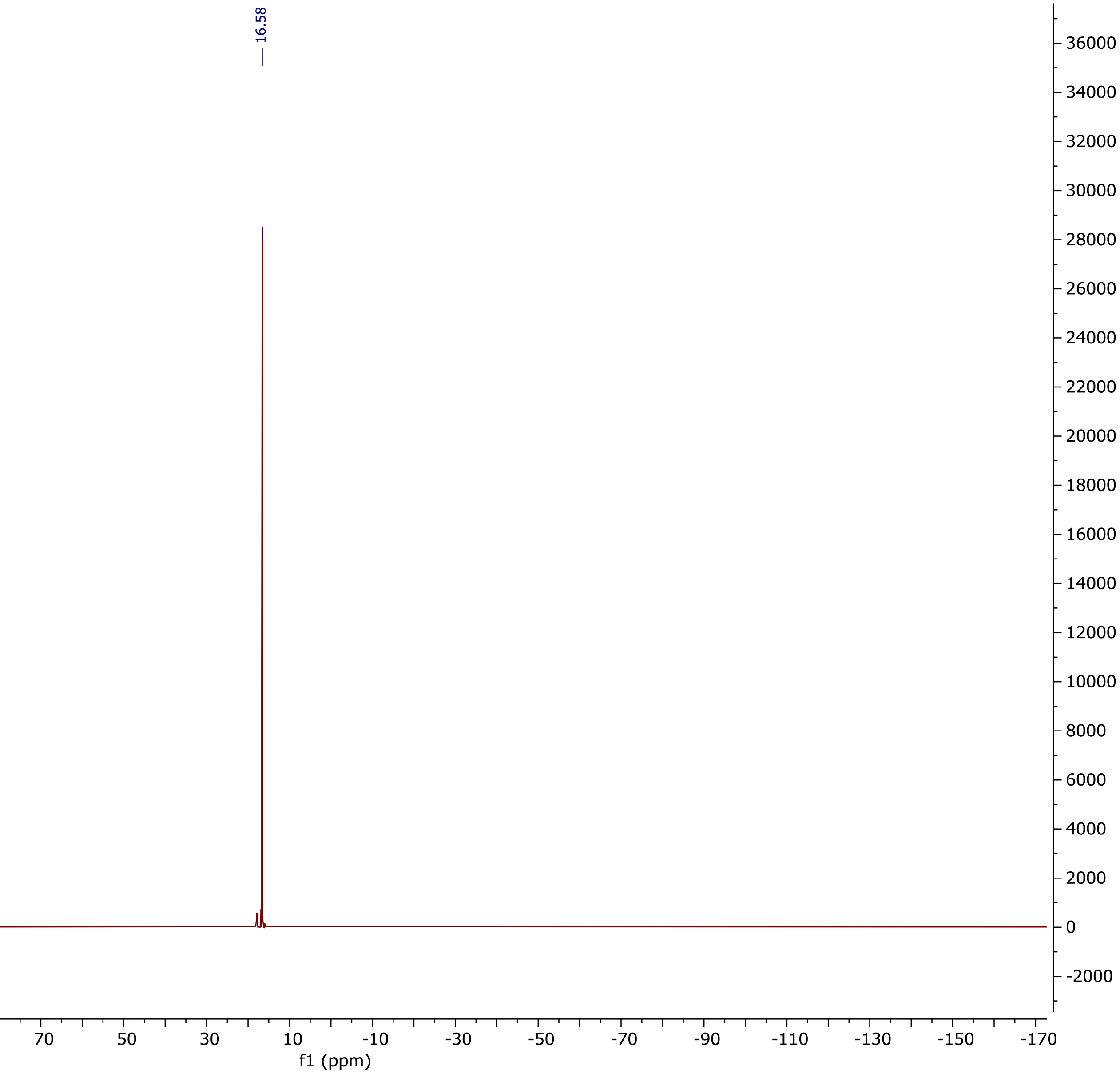

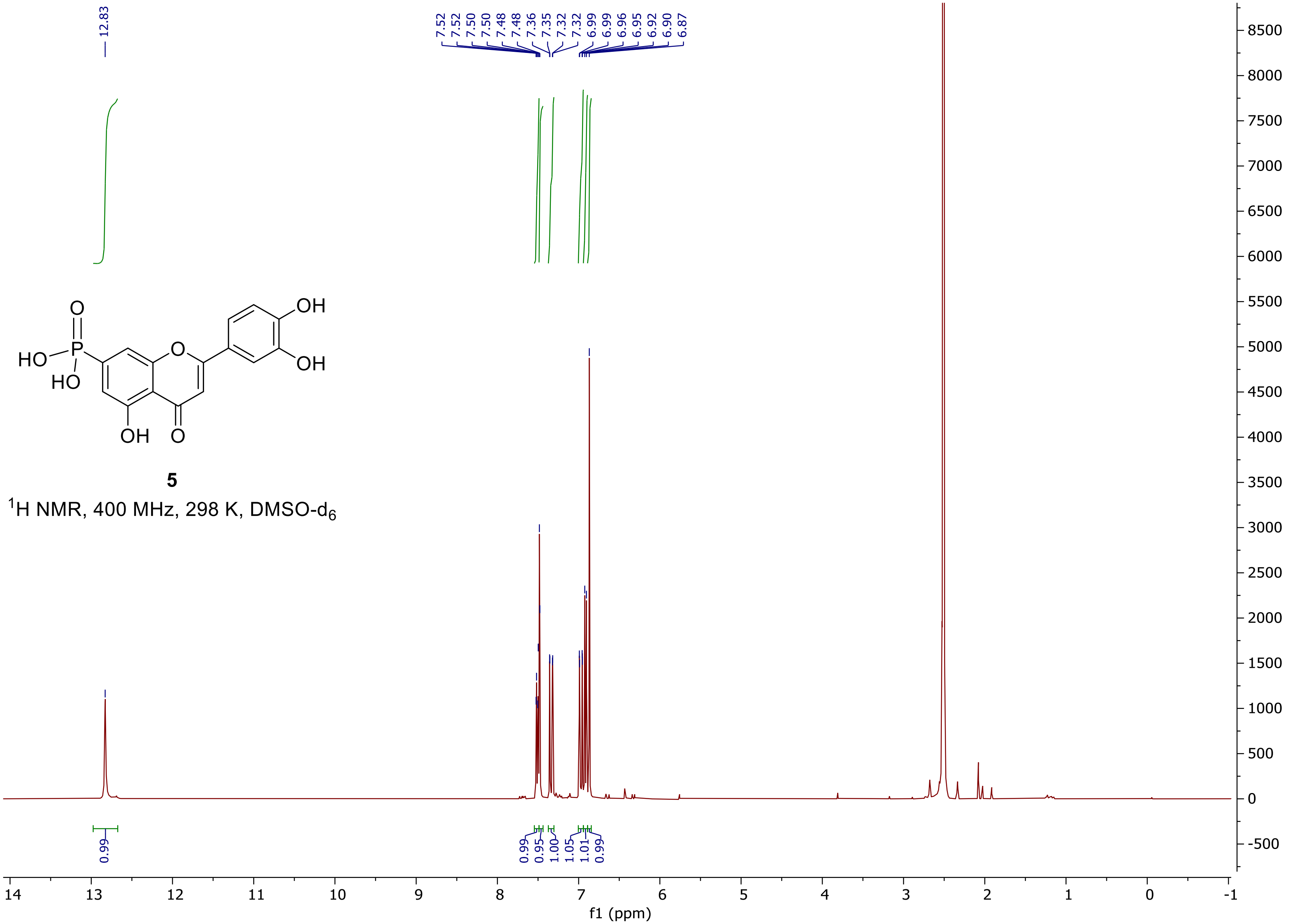
12.83
7.52
7.52
7.50
7.50
7.48
7.48
7.36
7.35
7.32
7.32
6.99
6.99
6.96
6.95
6.92
6.90
6.87
OH
O
HO
P
O
HO
OH
OH
O
5
^{1}H NMR, 400 MHz, 298 K, DMSO-d$_6$
0.99
0.99
0.95
1.00
1.05
1.01
0.99
f1 (ppm)
14 13 12 11 10 9 8 7 6 5 4 3 2 1 0 -1
8500 8000 7500 7000 6500 6000 5500 5000 4500 4000 3500 3000 2500 2000 1500 1000 500 0 -500

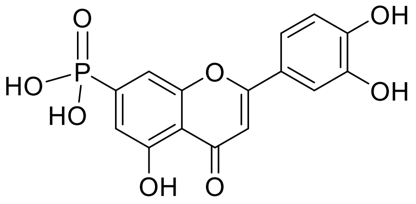

5

^{13}C NMR, 101 MHz, 298 K, DMSO-d_6

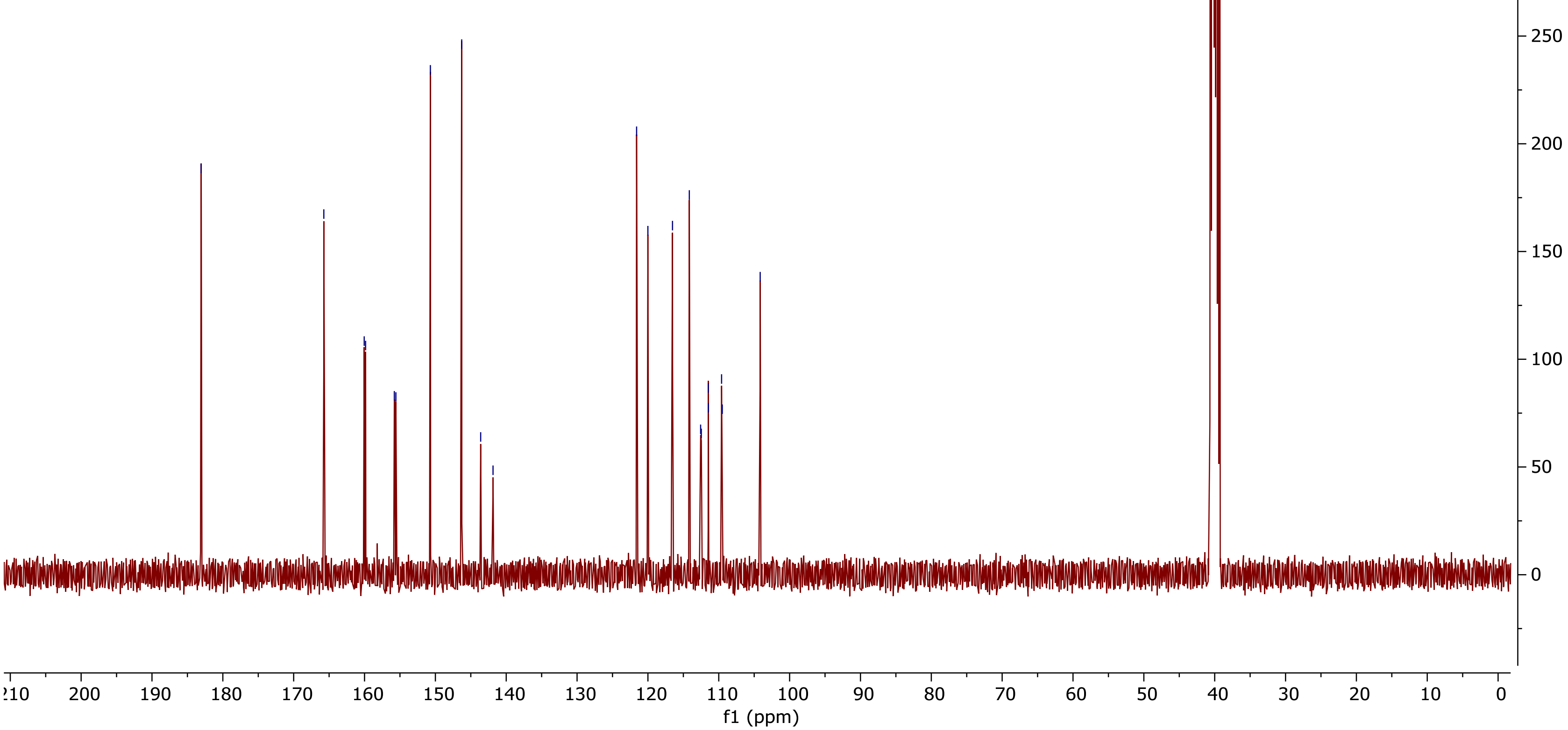

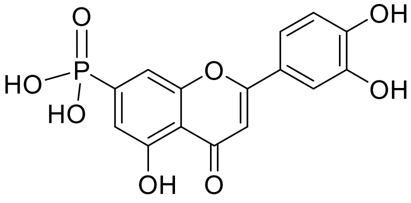

5

^{31}P NMR, 162 MHz, 298 K, DMSO-d_6

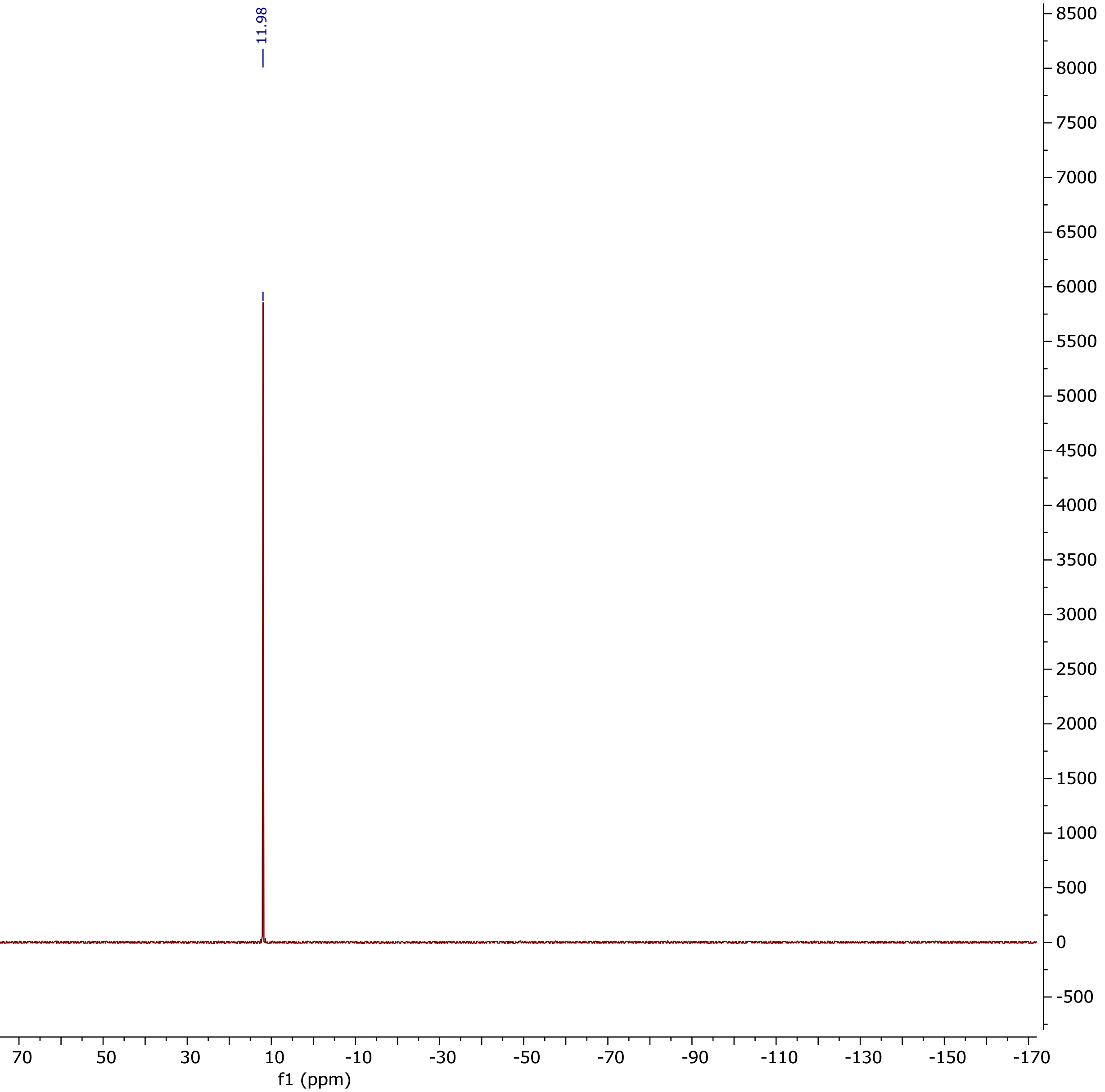

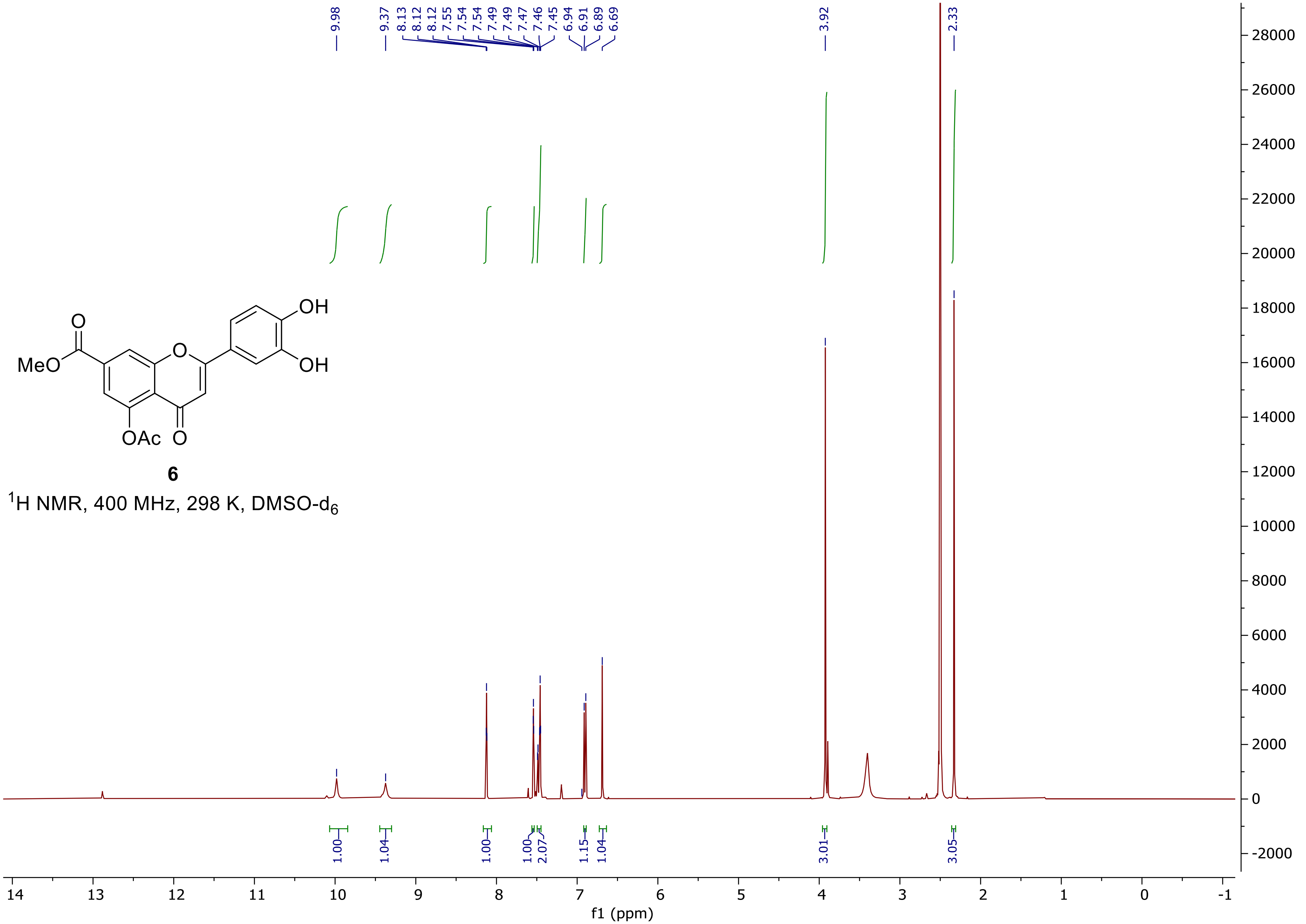

6
1H NMR, 400 MHz, 298 K, DMSO-d6
9.98
9.37
8.13
8.12
8.12
7.55
7.54
7.54
7.49
7.49
7.47
7.46
7.45
6.94
6.91
6.89
6.69
3.92
2.33
1.00
1.04
1.00
1.00
2.07
1.15
1.04
3.01
3.05
f1 (ppm)

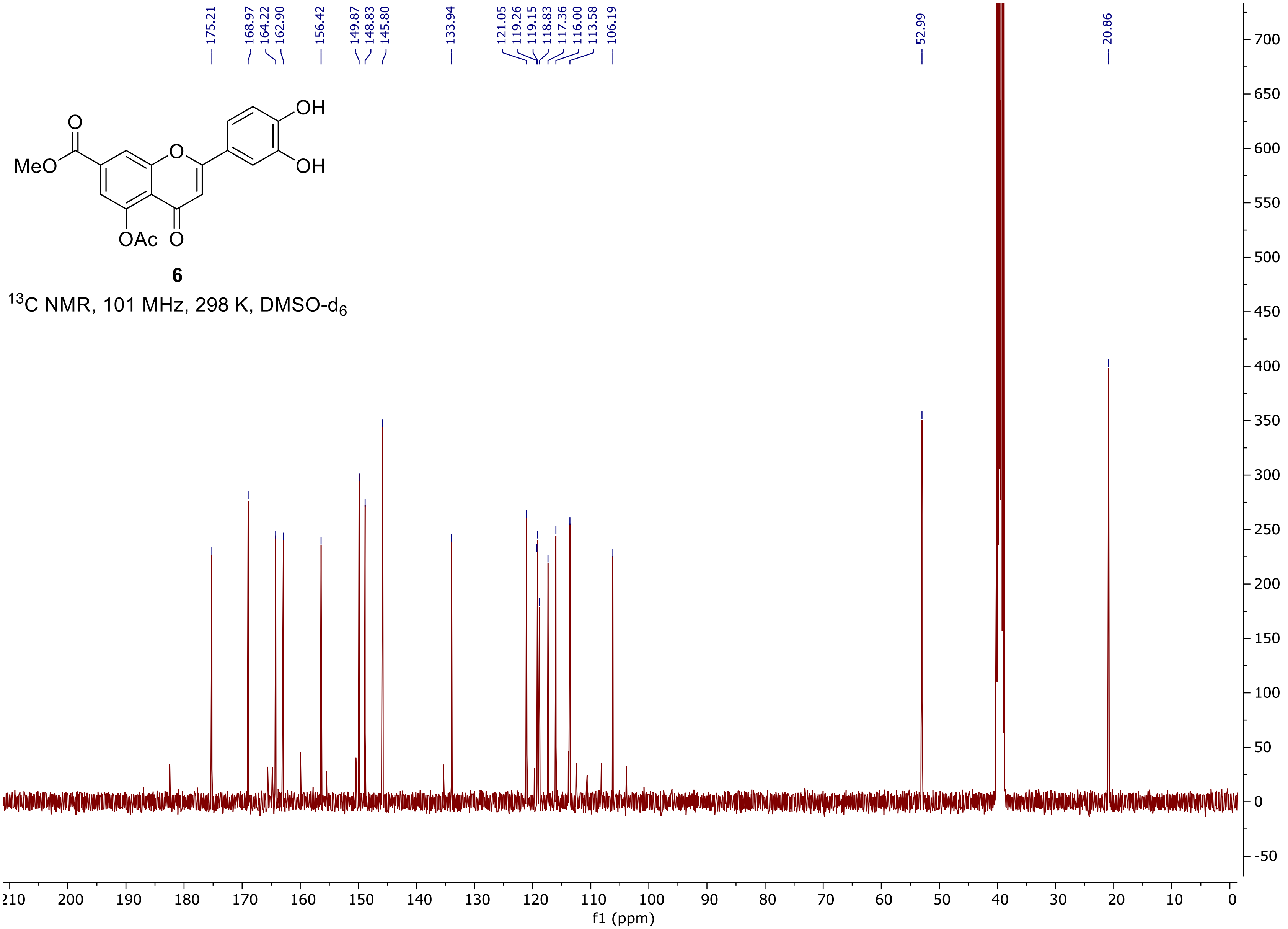
175.21
168.97
164.22
162.90
156.42
149.87
148.83
145.80
133.94
121.05
119.26
119.15
118.83
117.36
116.00
113.58
106.19
52.99
20.86
O
MeO
O
OH
OH
OAc
O
6
^{13}C NMR, 101 MHz, 298 K, DMSO-d$_6$
f1 (ppm)

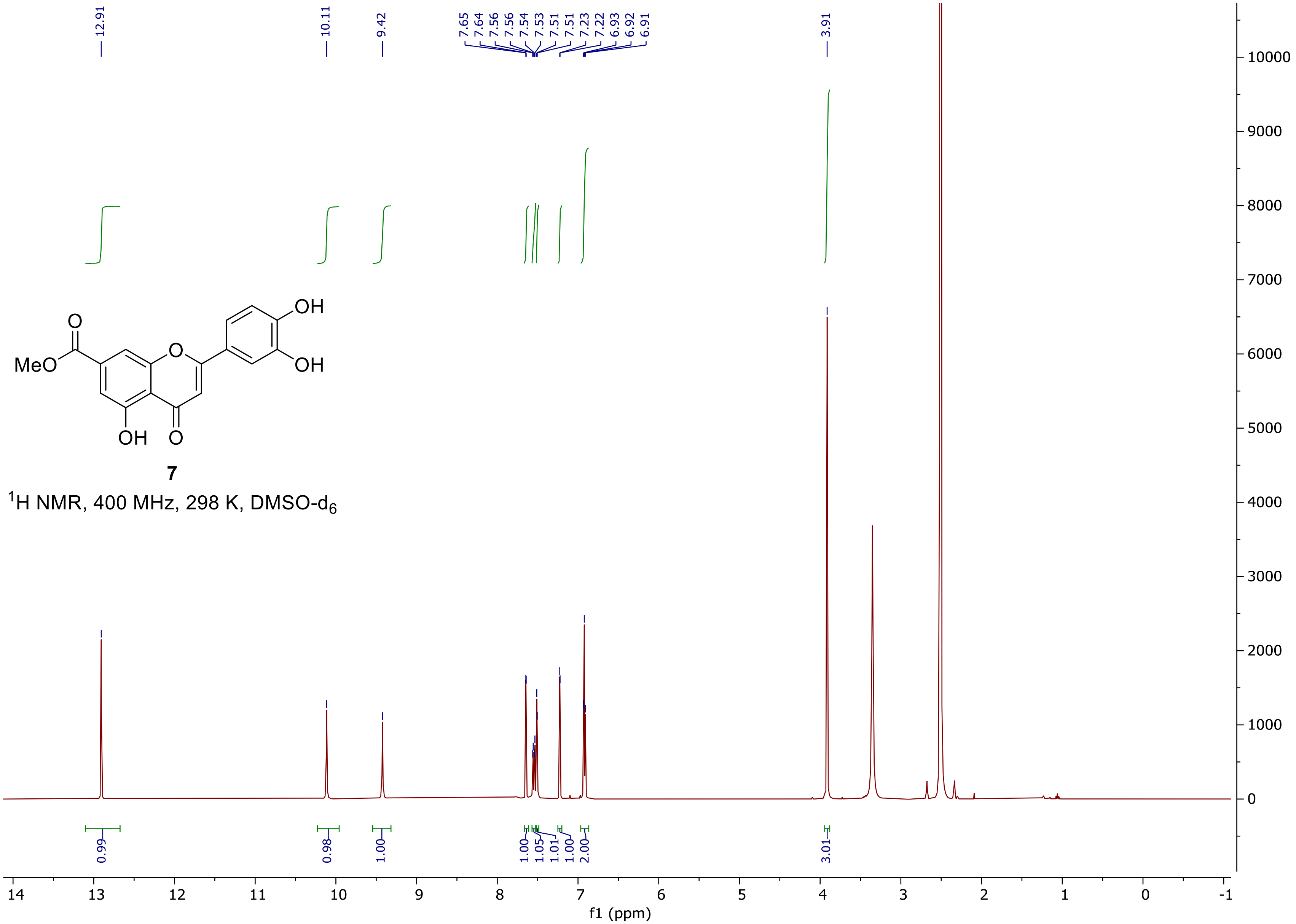
12.91
10.11
9.42
7.65
7.64
7.56
7.56
7.54
7.53
7.51
7.51
7.23
7.22
6.93
6.92
6.91
3.91
0.99
0.98
1.00
1.00
1.05
1.01
1.00
2.00
3.01
f1 (ppm)
MeO
O
OH
OH
OH
O
7
^{1}H NMR, 400 MHz, 298 K, DMSO-d6

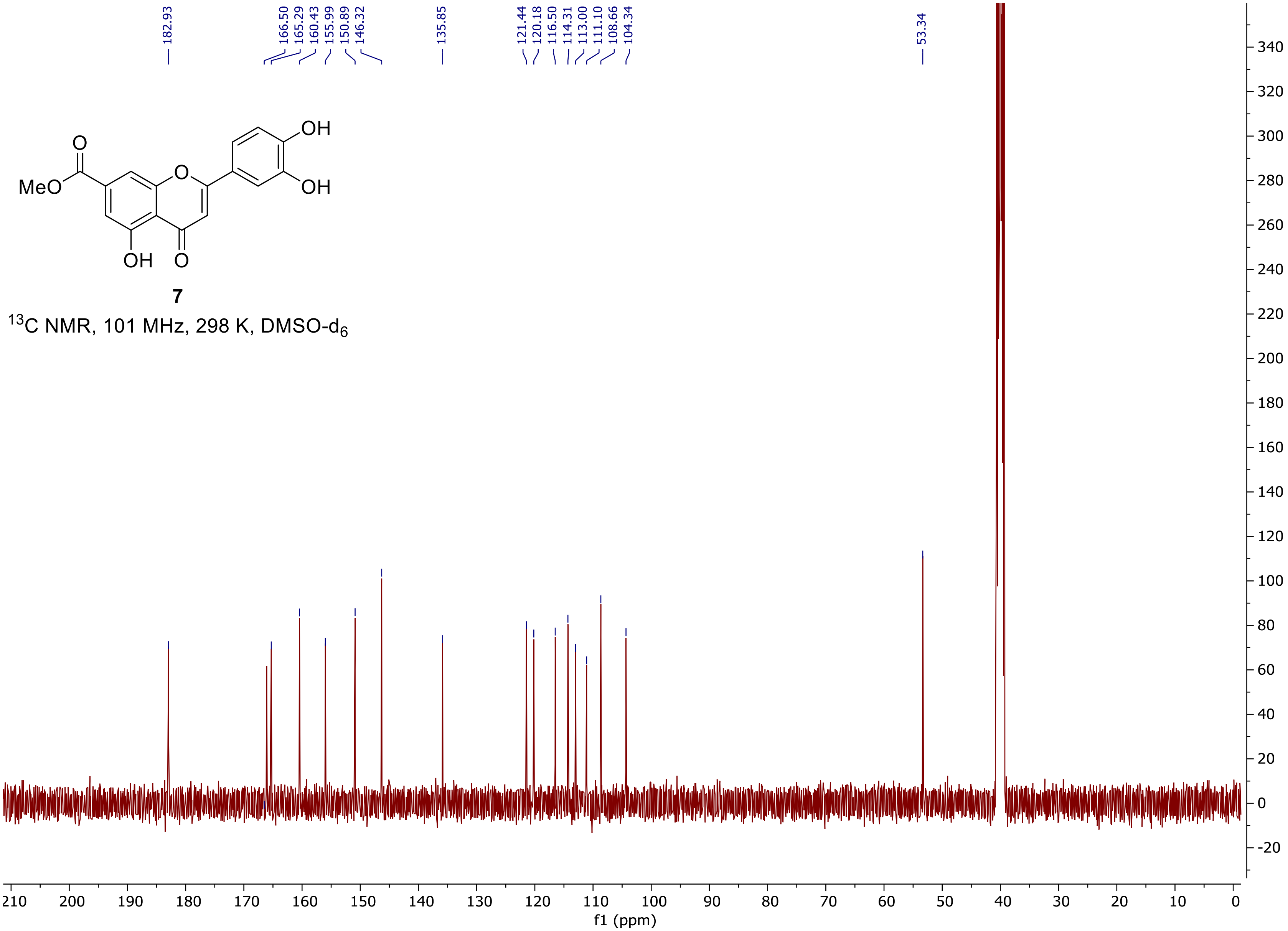

182.93
166.50
165.29
160.43
155.99
150.89
146.32
135.85
121.44
120.18
116.50
114.31
113.00
111.10
108.66
104.34
53.34
MeO
O
OH
7
^{13}C NMR, 101 MHz, 298 K, DMSO-d$_6$
f1 (ppm)

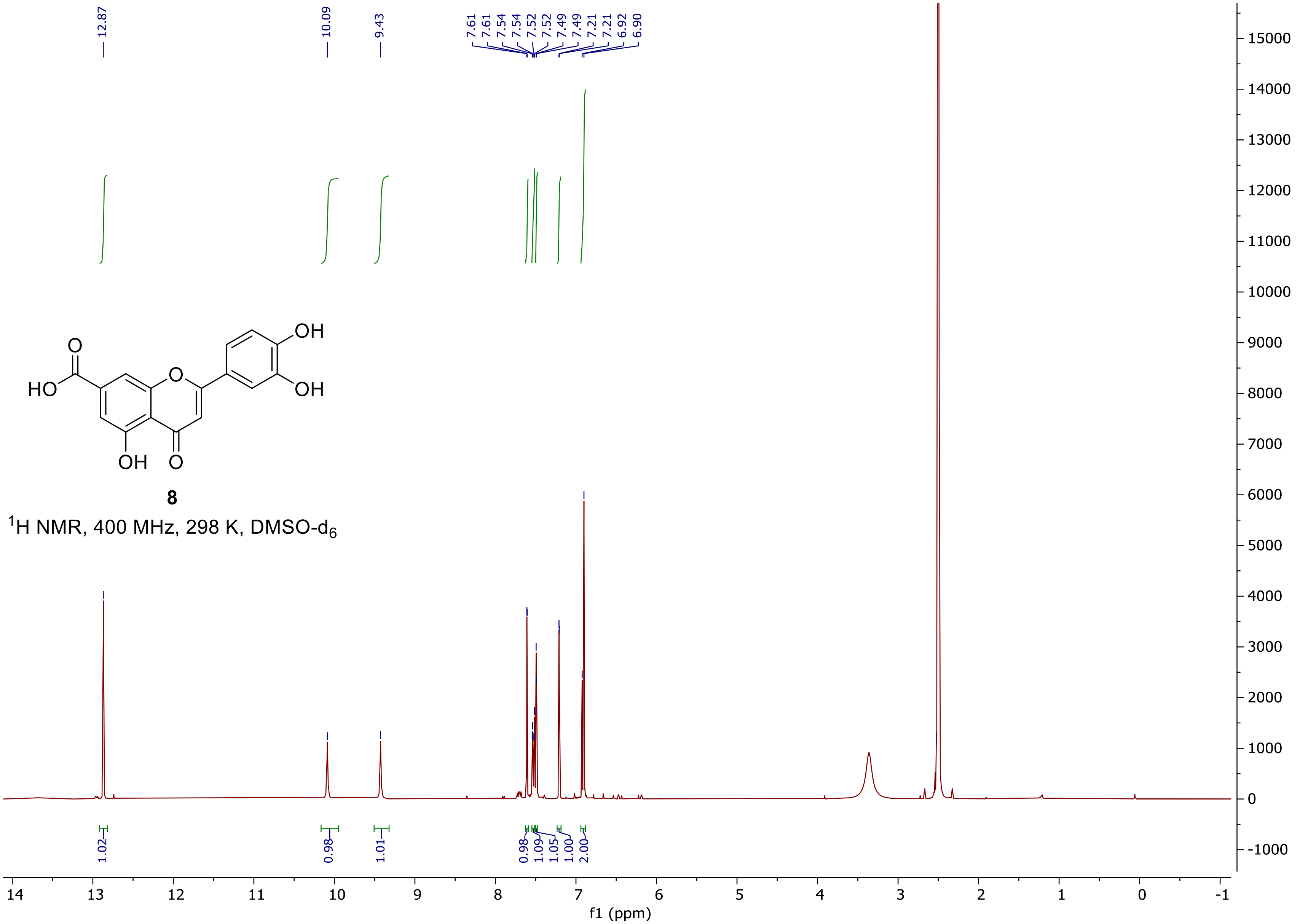
12.87
10.09
9.43
7.61
7.61
7.54
7.54
7.52
7.52
7.49
7.49
7.21
7.21
6.92
6.90
OH
O
HO
O
OH
OH
O
8
1H NMR, 400 MHz, 298 K, DMSO-d6
1.02
0.98
1.01
0.98
1.09
1.05
1.00
2.00
f1 (ppm)

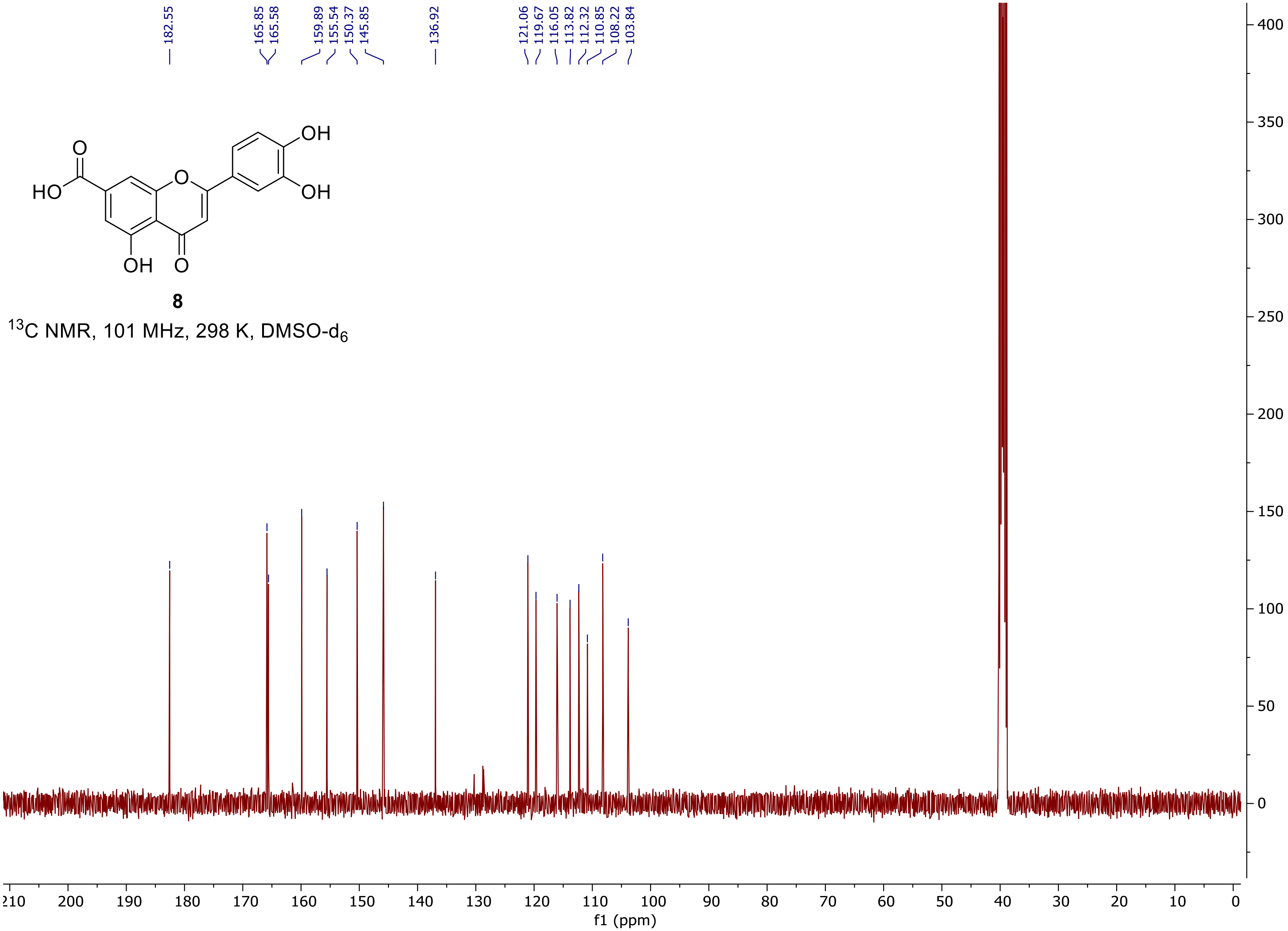

8

^{13}C NMR, 101 MHz, 298 K, DMSO-d$_6$

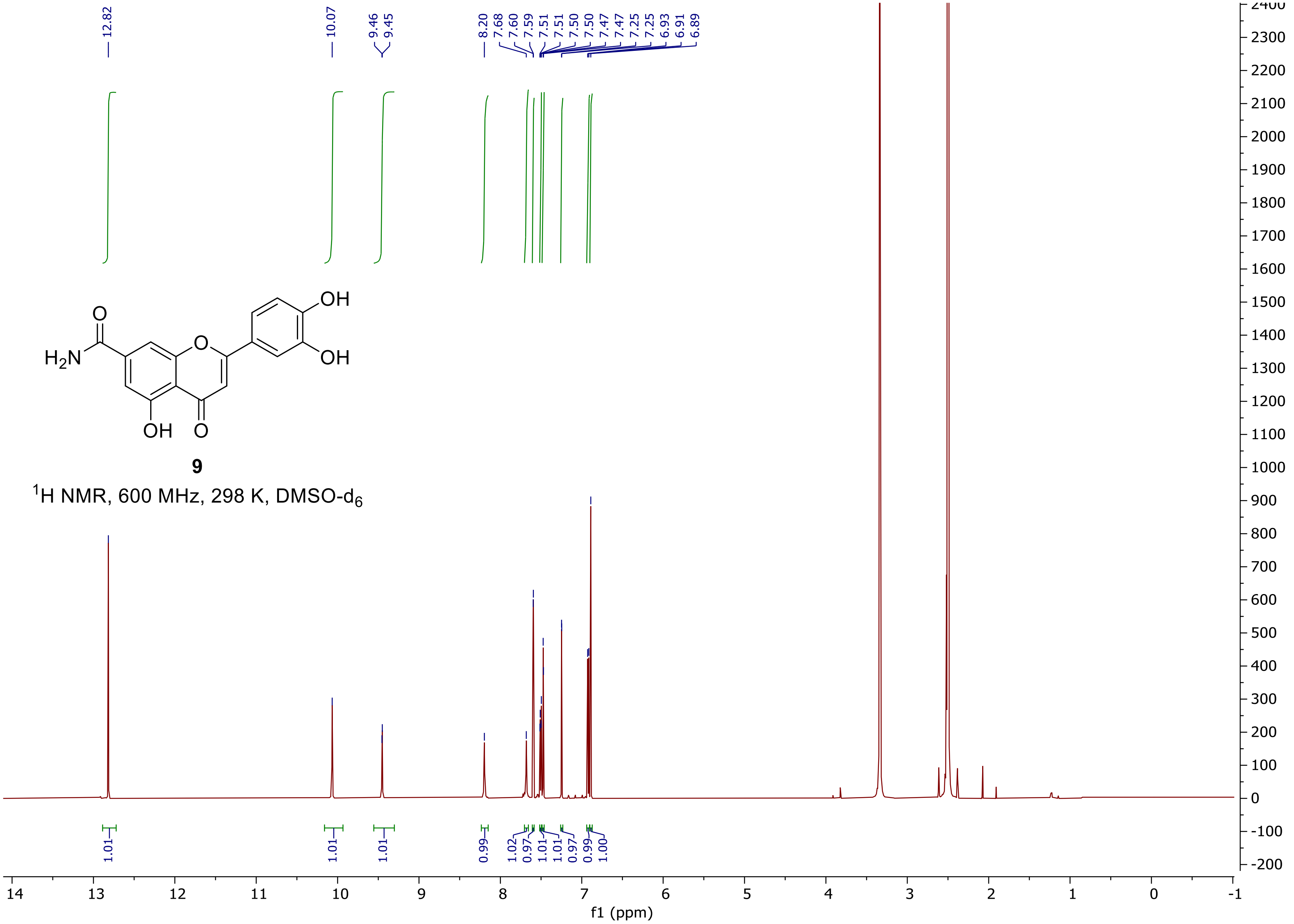
12.82
10.07
9.46
9.45
8.20
7.68
7.60
7.59
7.51
7.51
7.50
7.50
7.47
7.47
7.25
7.25
6.93
6.91
6.89
O
H_2N
O
OH
OH
OH
O
9
^{1}H NMR, 600 MHz, 298 K, DMSO-d$_6$
1.01
1.01
1.01
0.99
1.02
0.97
1.01
1.01
0.97
0.99
1.00
f1 (ppm)

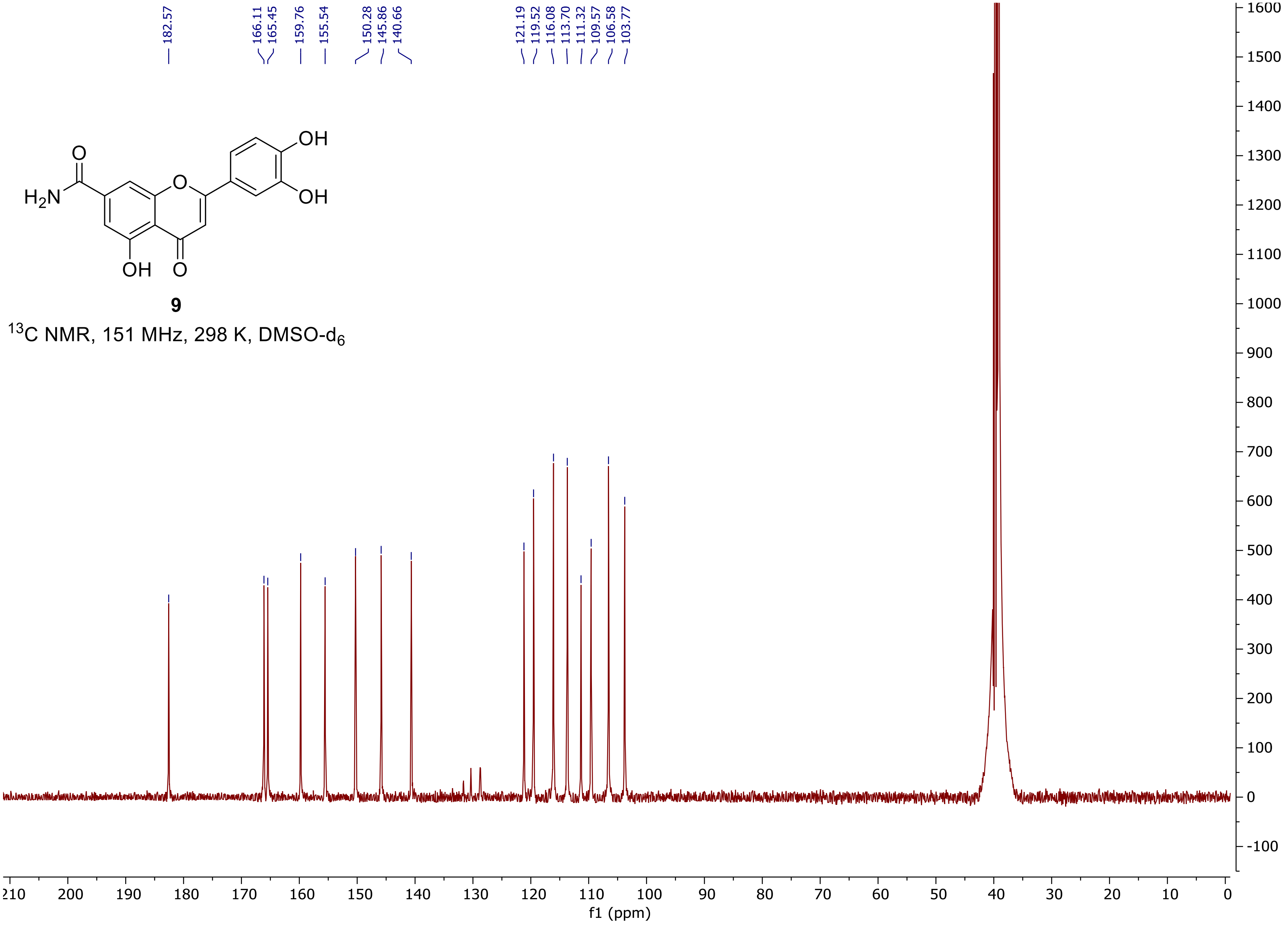
182.57
166.11
165.45
159.76
155.54
150.28
145.86
140.66
121.19
119.52
116.08
113.70
111.32
109.57
106.58
103.77
O
H2N
O
OH
OH
OH
O
9
13C NMR, 151 MHz, 298 K, DMSO-d6
f1 (ppm)

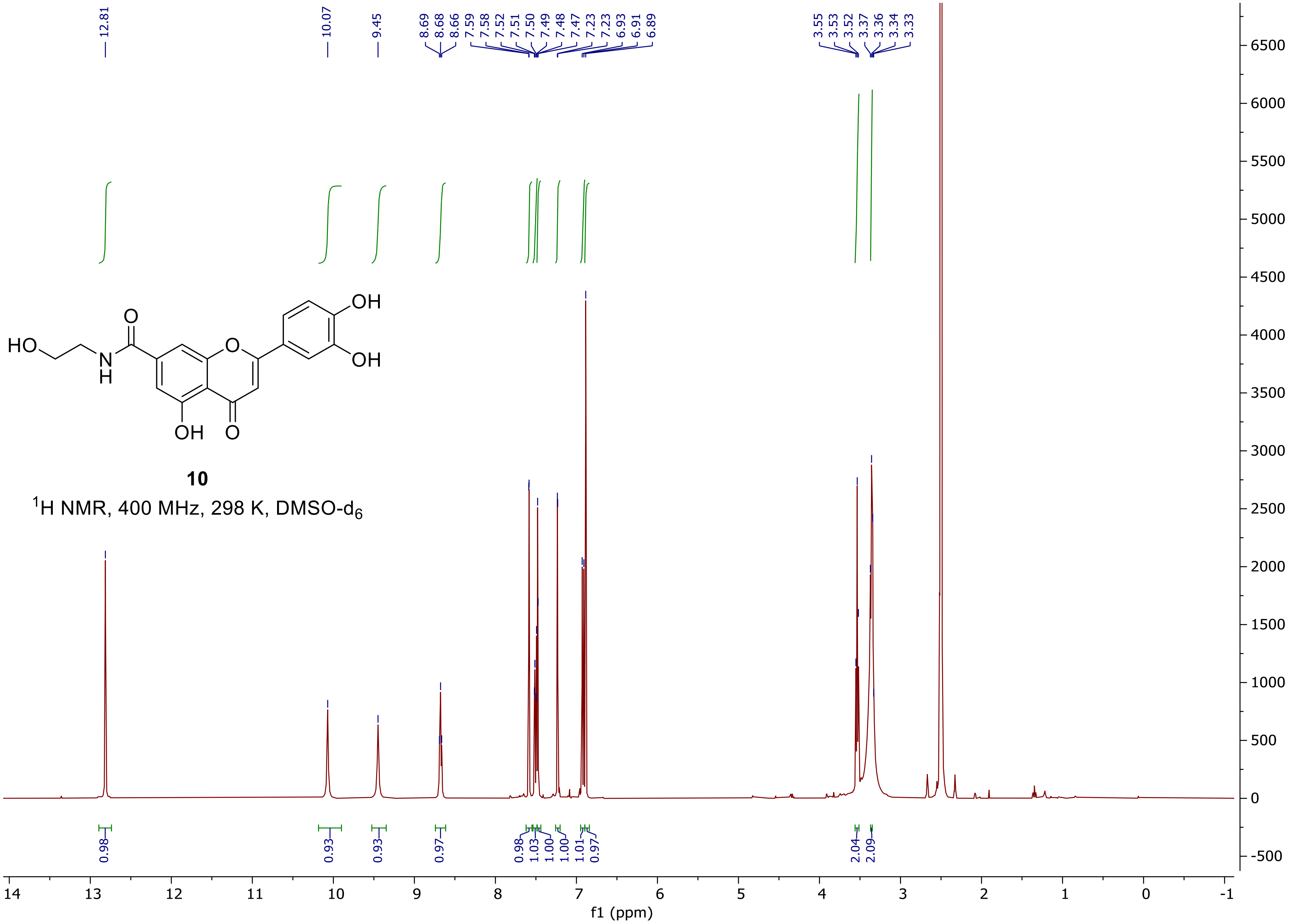
12.81
10.07
9.45
8.69
8.68
8.66
7.59
7.58
7.52
7.51
7.50
7.49
7.48
7.47
7.23
7.23
6.93
6.91
6.89
3.55
3.53
3.52
3.37
3.36
3.34
3.33
HO
O
N
H
O
OH
O
OH
OH
10
^{1}H NMR, 400 MHz, 298 K, DMSO-d$_6$
0.98
0.93
0.93
0.97
0.98
1.03
1.00
1.00
1.01
0.97
2.04
2.09
f1 (ppm)

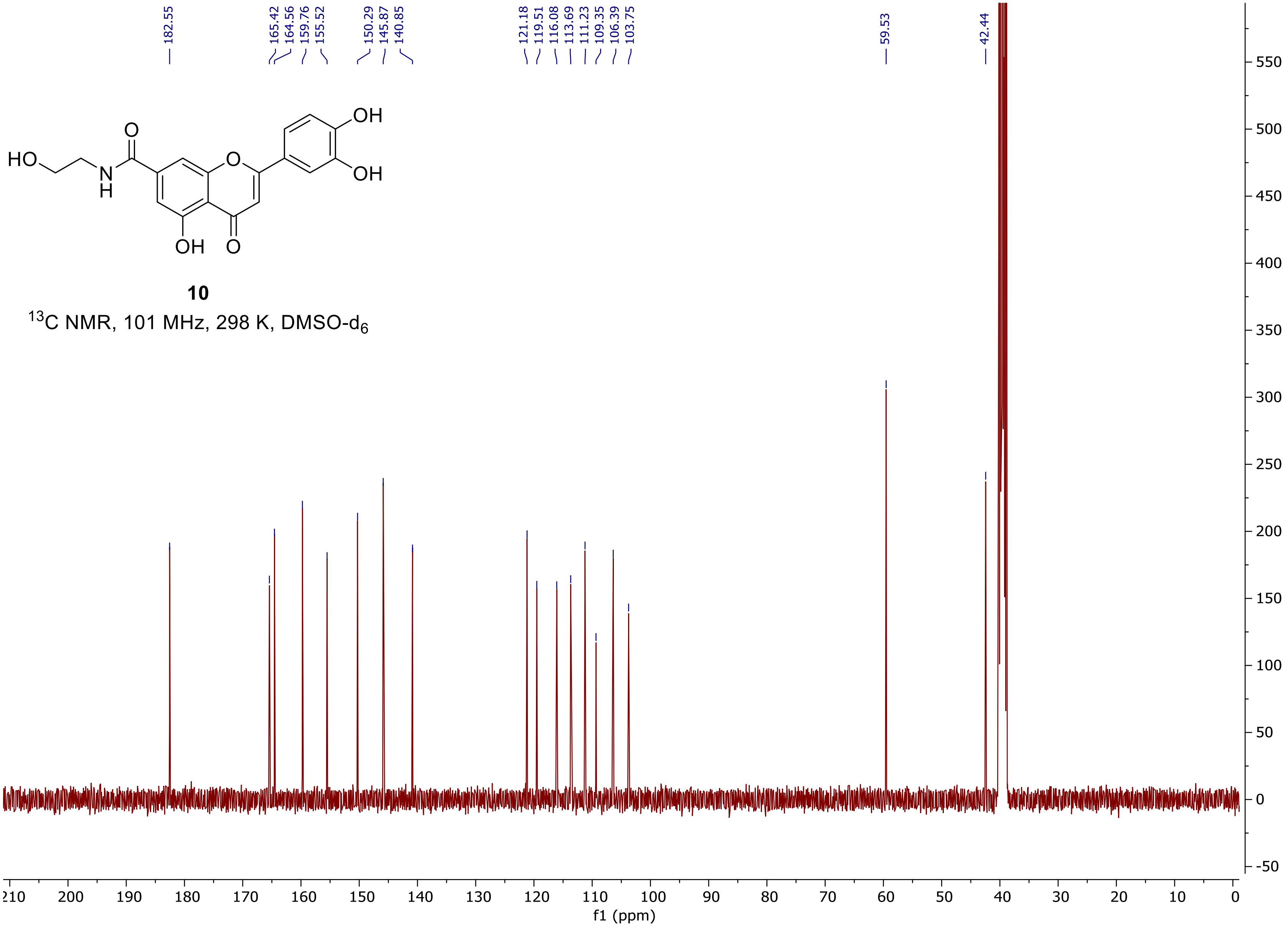
182.55
165.42
164.56
159.76
155.52
150.29
145.87
140.85
121.18
119.51
116.08
113.69
111.23
109.35
106.39
103.75
59.53
42.44
HO
N
H
O
O
OH
OH
OH
O
10
13C NMR, 101 MHz, 298 K, DMSO-d6
f1 (ppm)

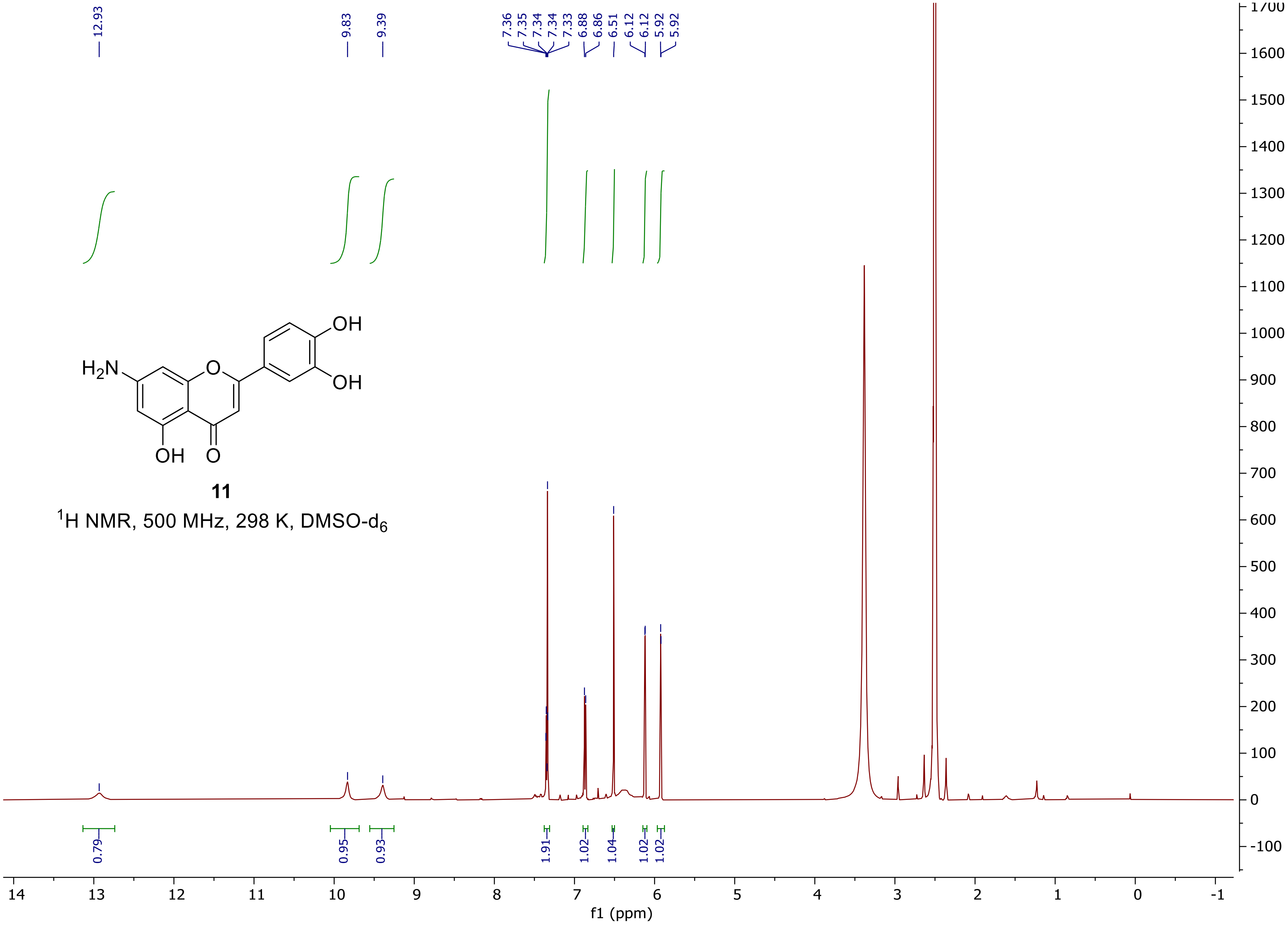

11

^{1}H NMR, 500 MHz, 298 K, DMSO-d$_6$

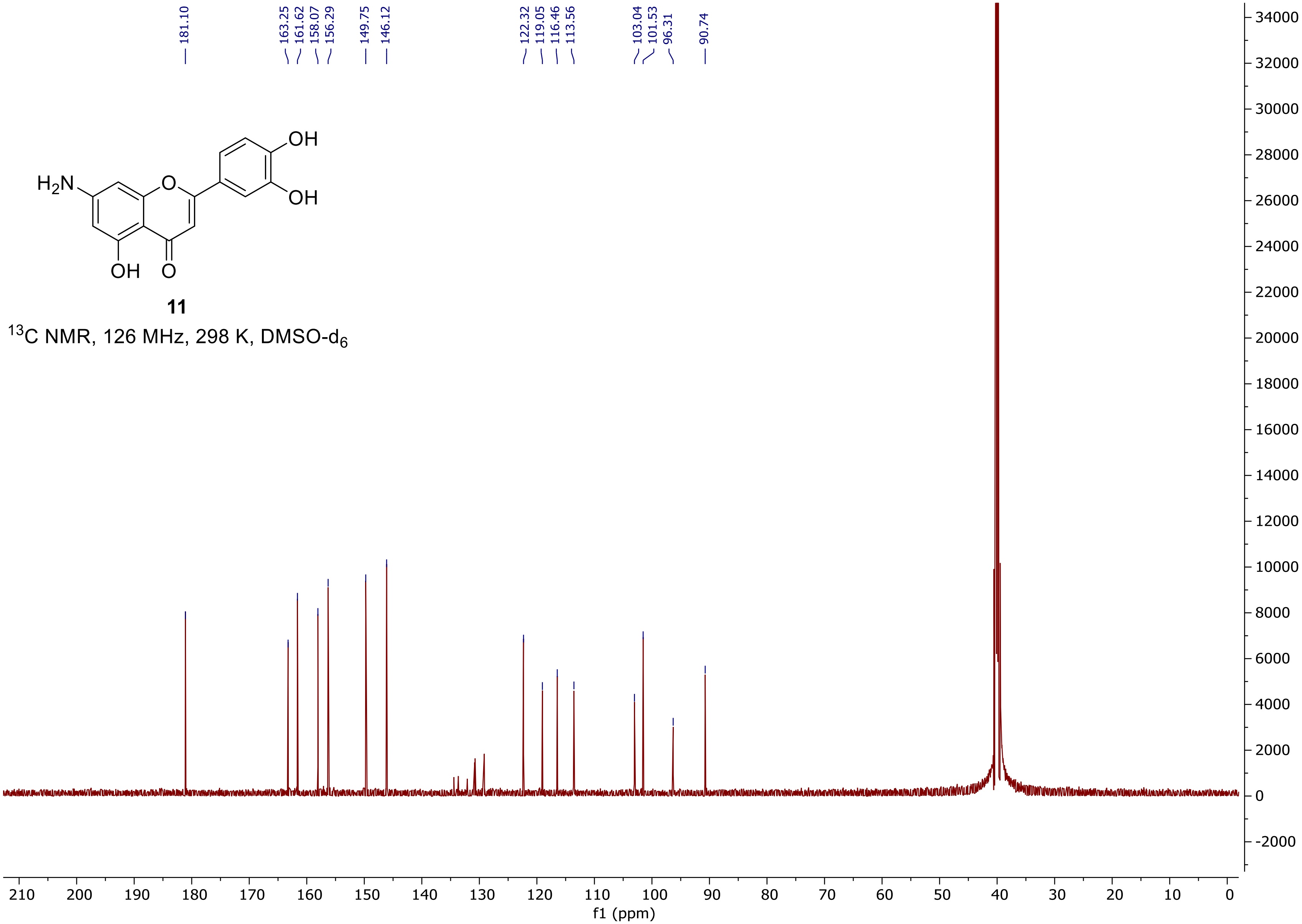

11

^{13}C NMR, 126 MHz, 298 K, DMSO-d_6

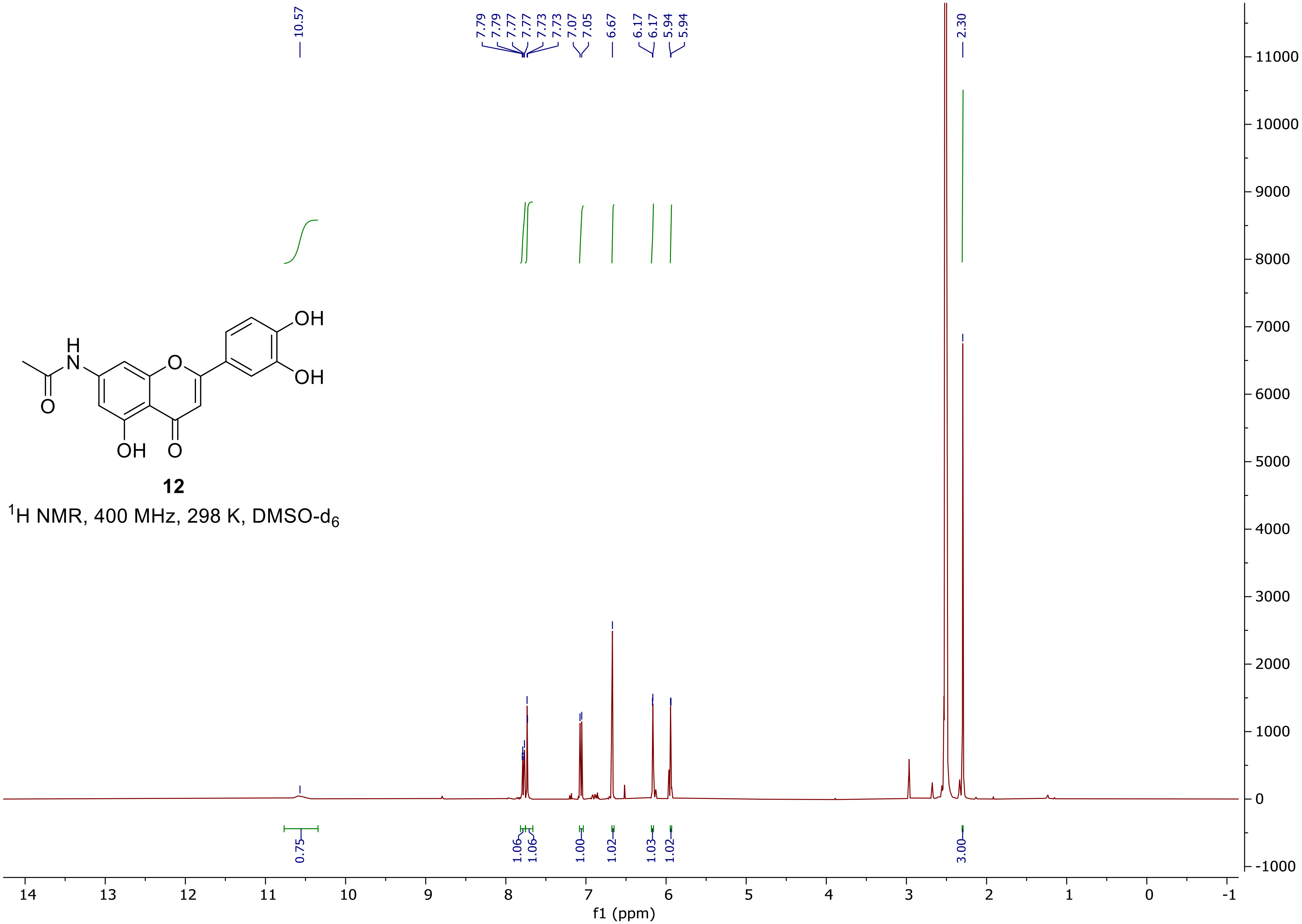

12

^{1}H NMR, 400 MHz, 298 K, DMSO-d$_6$

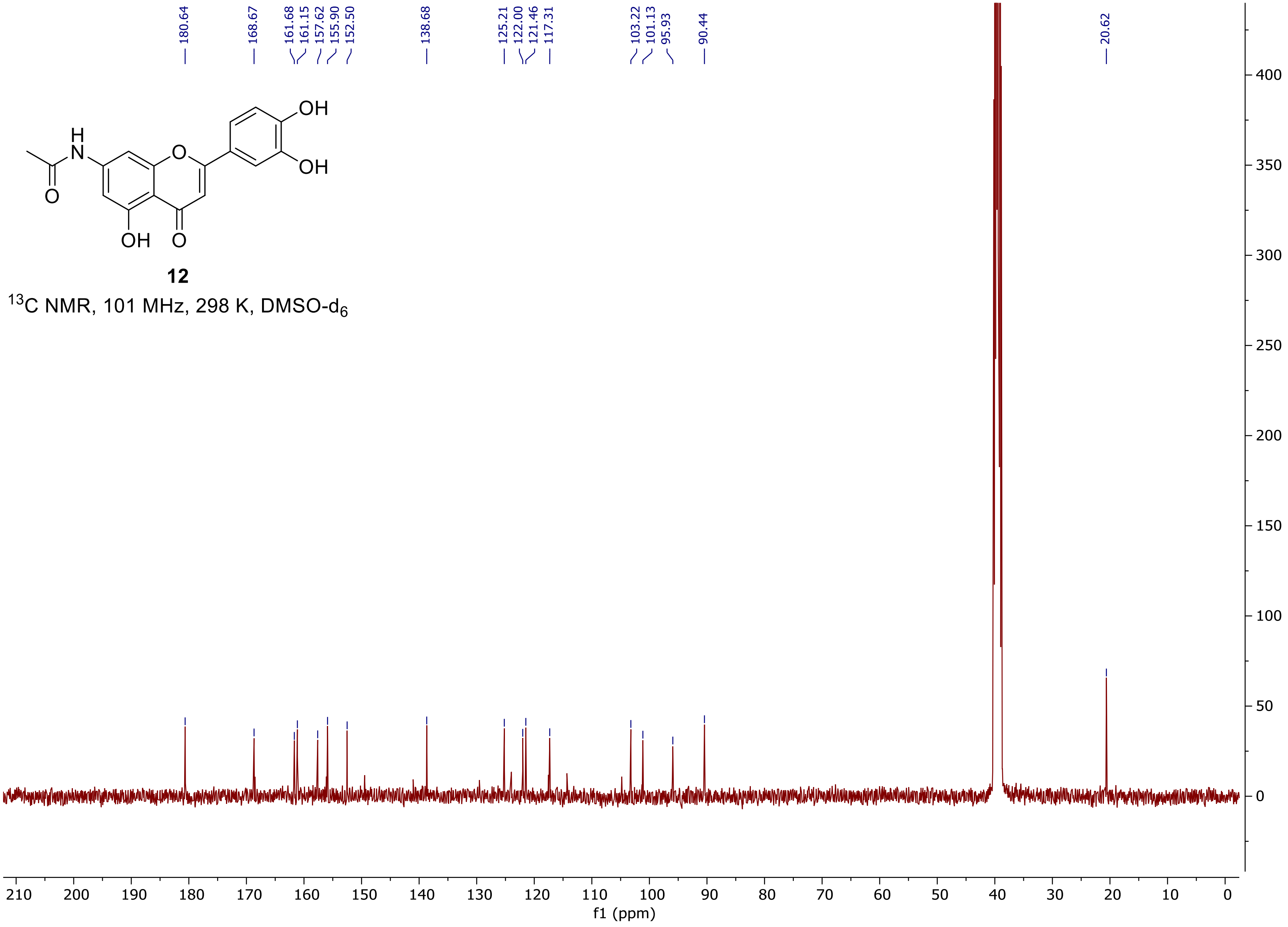
180.64
168.67
161.68
161.15
157.62
155.90
152.50
138.68
125.21
122.00
121.46
117.31
103.22
101.13
95.93
90.44
20.62
OH
H
N
O
O
OH
OH
O
12
^{13}C NMR, 101 MHz, 298 K, DMSO-d_6
f1 (ppm)

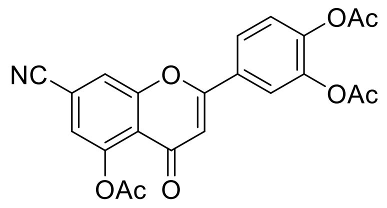

13

^{1}H NMR, 400 MHz, 298 K,CDCl$_3$

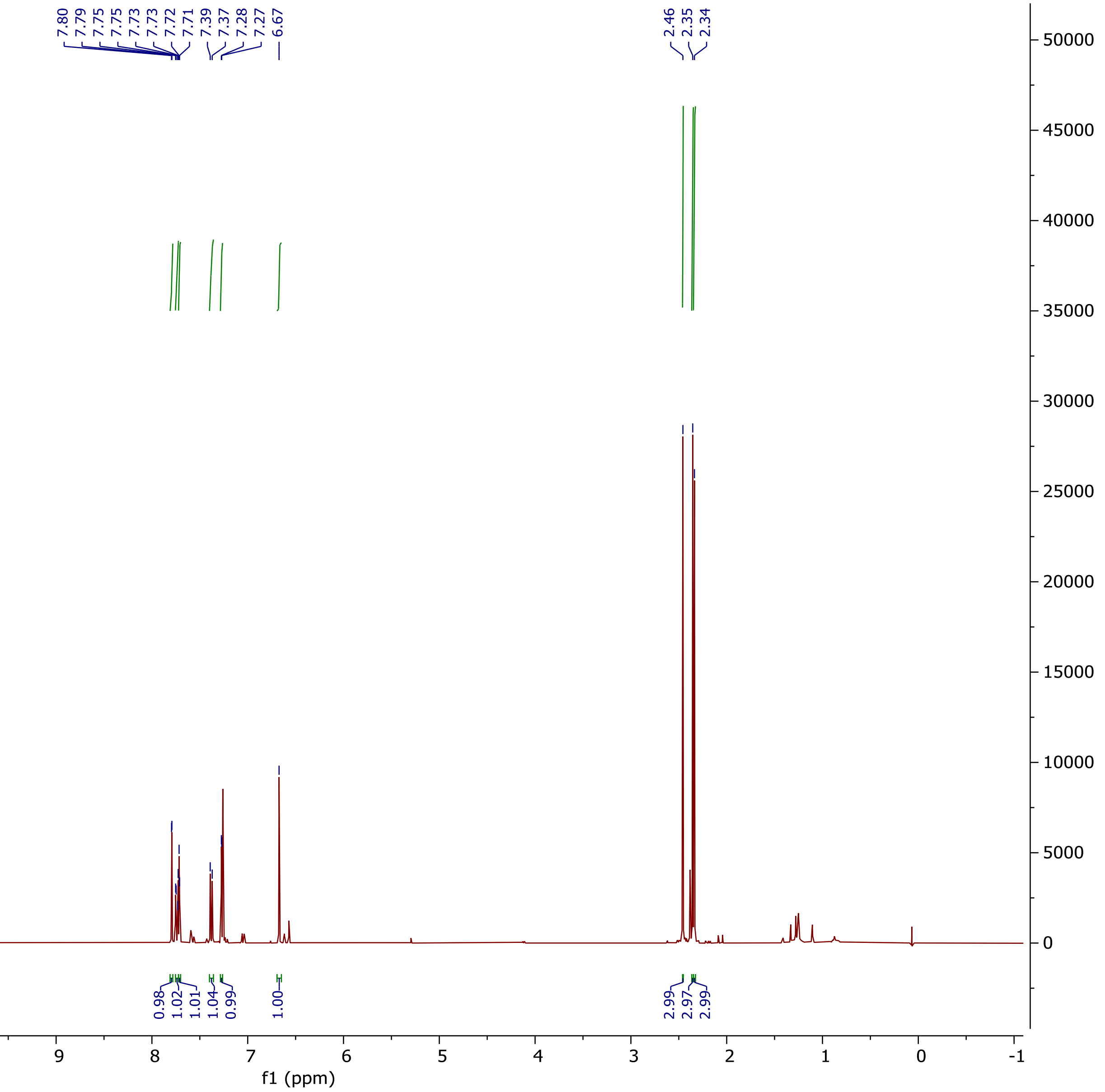

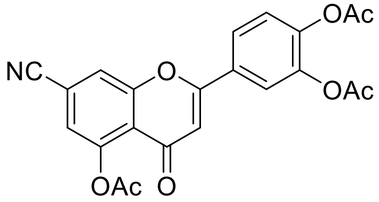

13

^{13}C NMR, 101 MHz, 298 K,$CDCl_3$

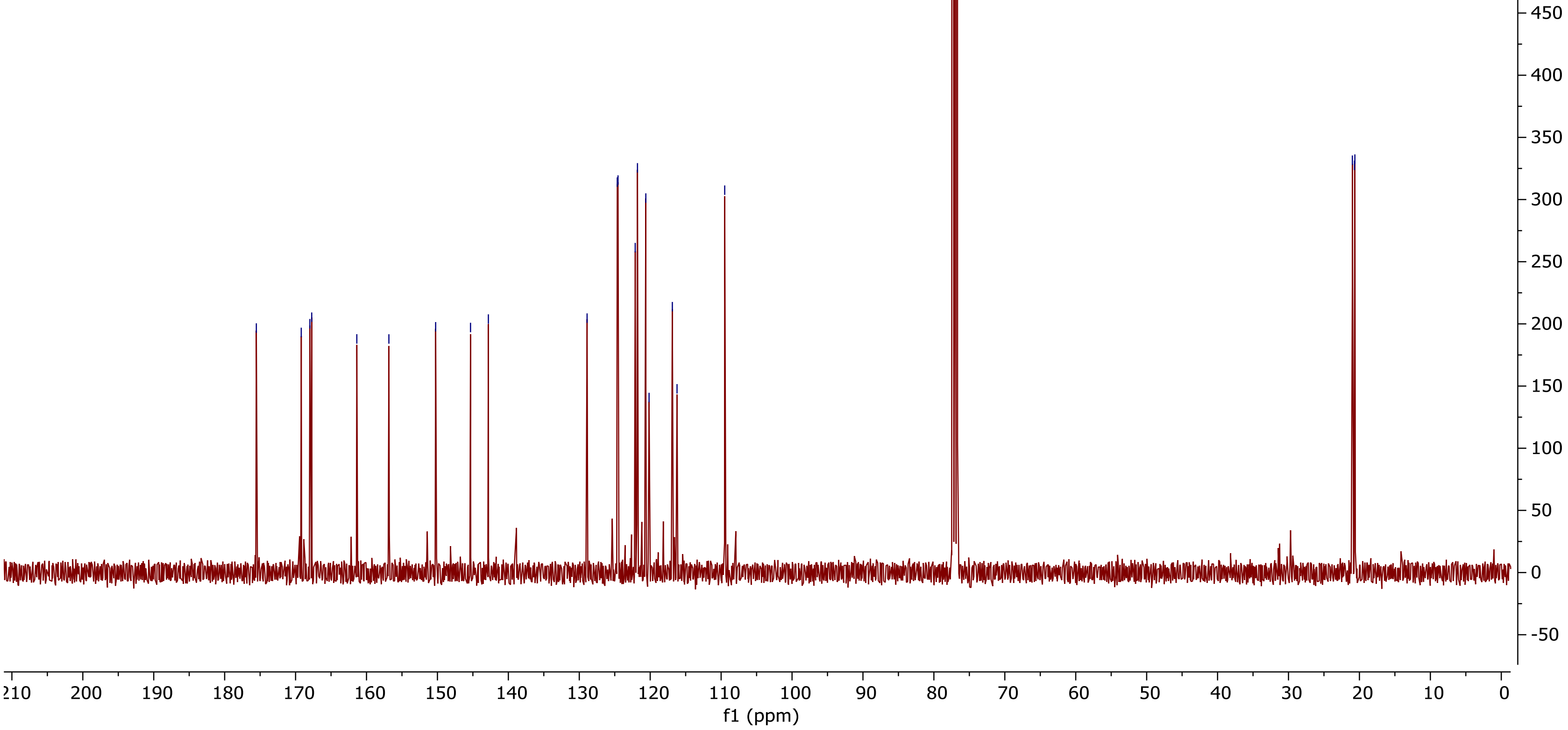

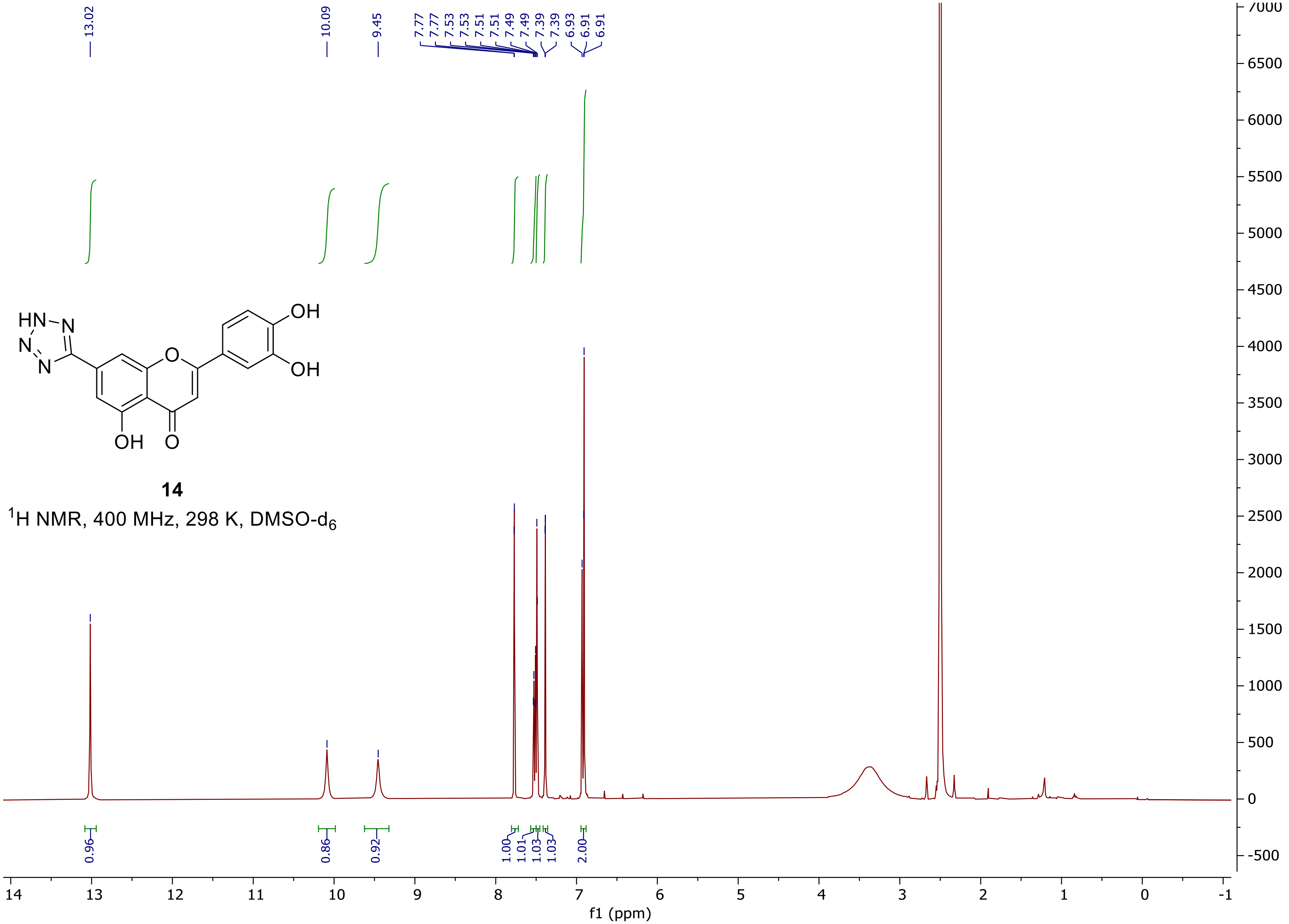
13.02
10.09
9.45
7.77
7.77
7.53
7.53
7.51
7.51
7.49
7.49
7.39
7.39
6.93
6.91
6.91
HN
N
N
N
O
OH
OH
OH
O
14
^{1}H NMR, 400 MHz, 298 K, DMSO-d$_6$
0.96
0.86
0.92
1.00
1.01
1.03
1.03
2.00
f1 (ppm)

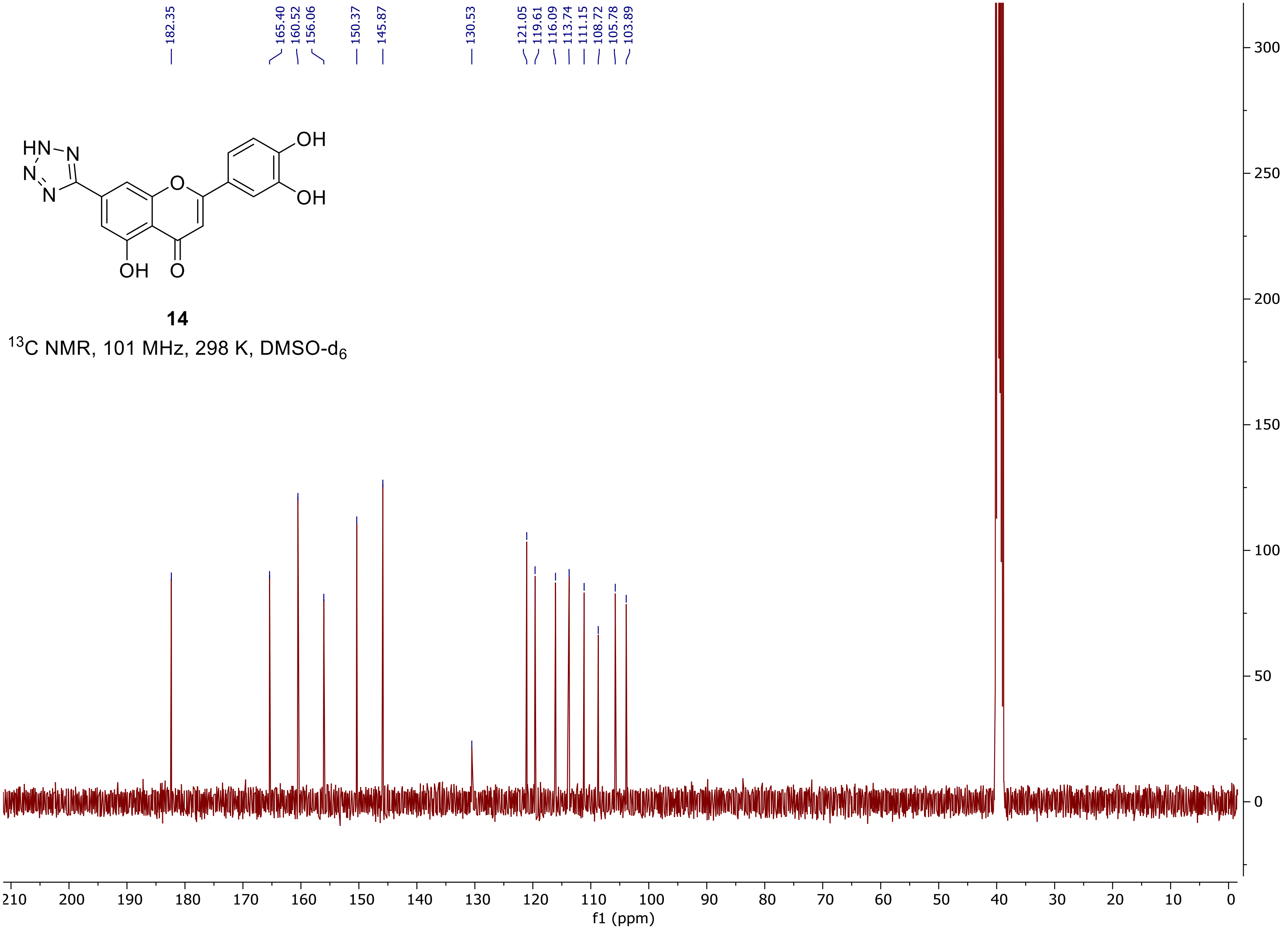
182.35
165.40
160.52
156.06
150.37
145.87
130.53
121.05
119.61
116.09
113.74
111.15
108.72
105.78
103.89
HN
N
N
N
O
OH
OH
OH
O
14
13C NMR, 101 MHz, 298 K, DMSO-d6
f1 (ppm)

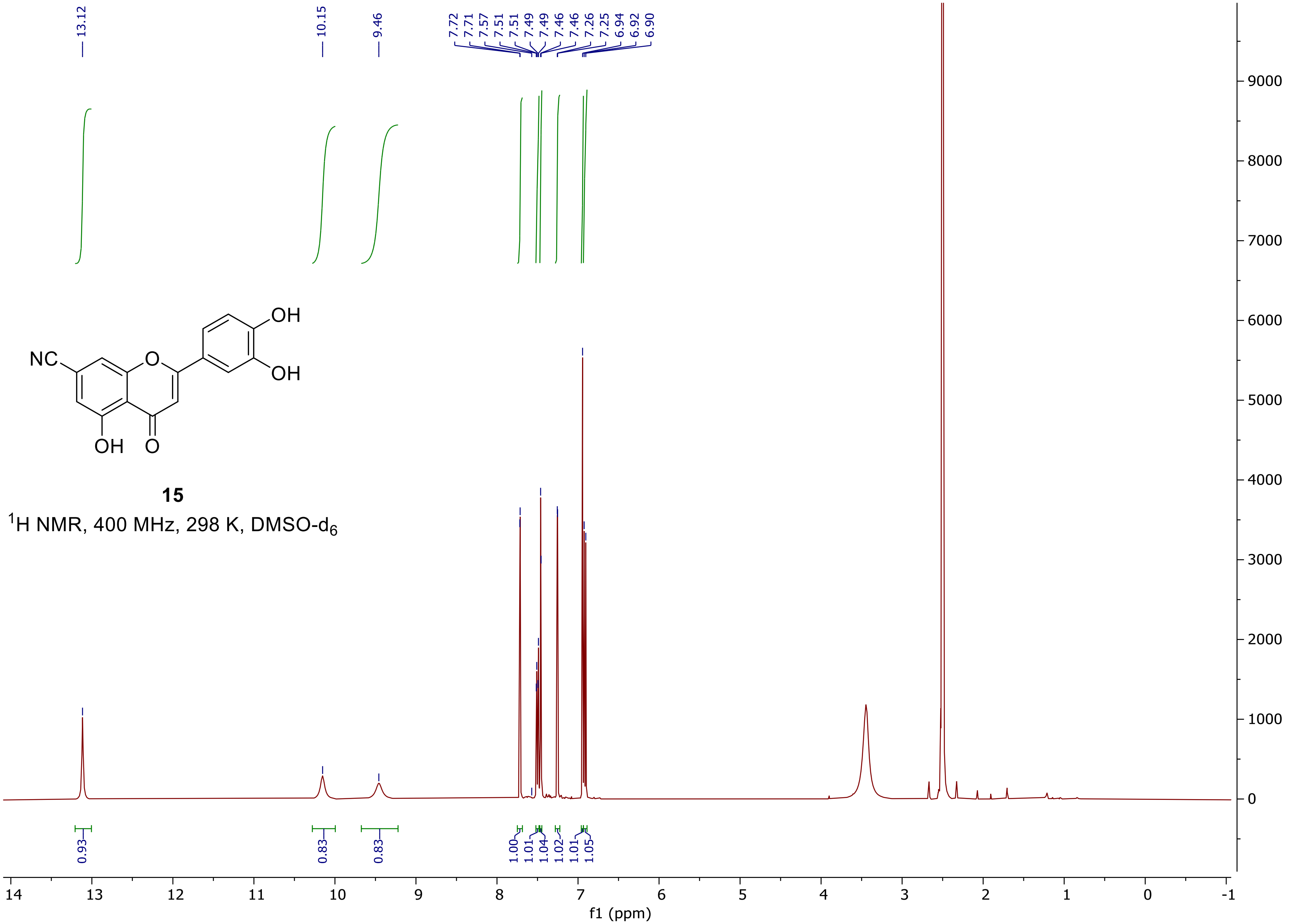

15

^{1}H NMR, 400 MHz, 298 K, DMSO-d_6

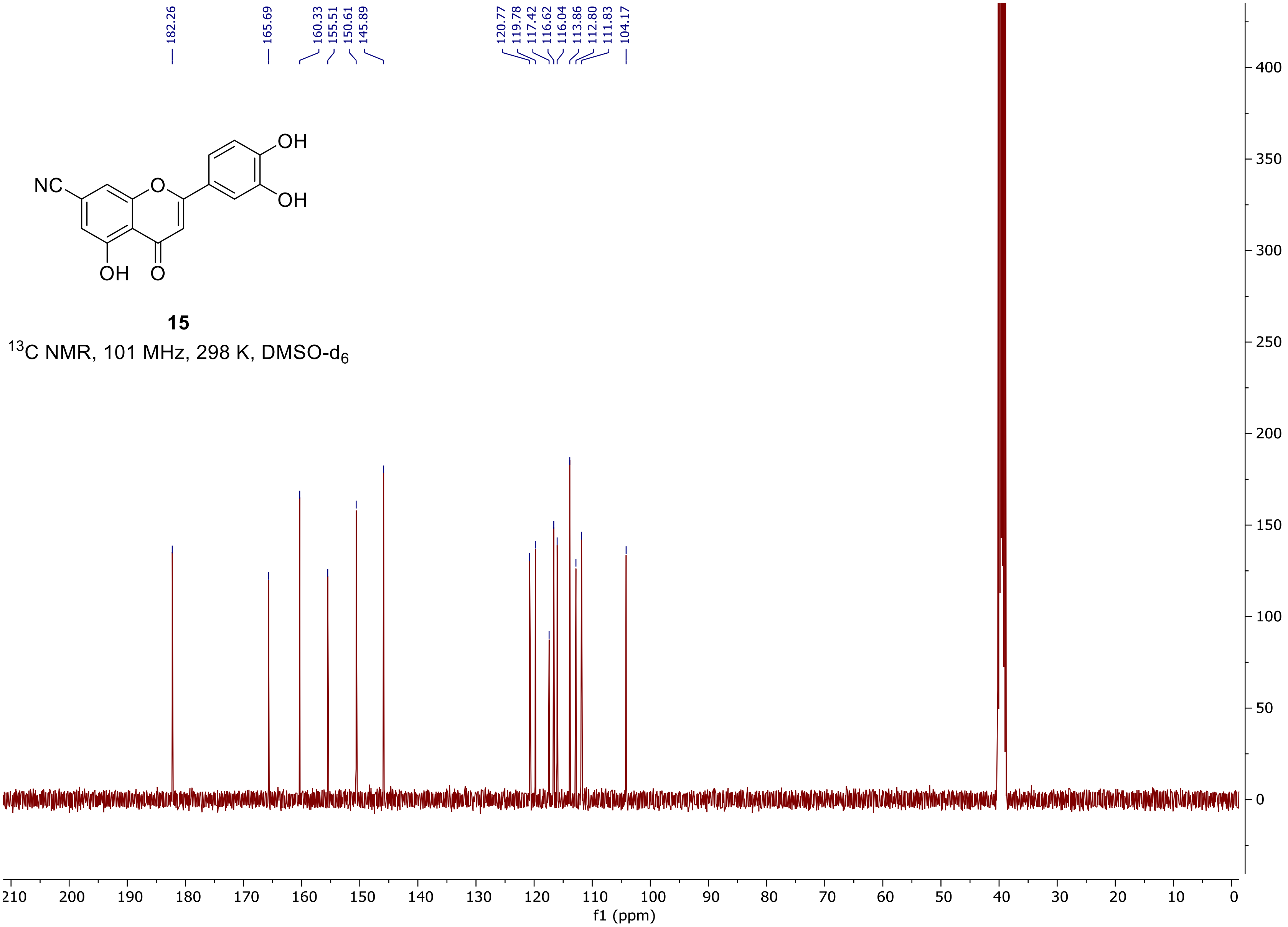

182.26
165.69
160.33
155.51
150.61
145.89
120.77
119.78
117.42
116.62
116.04
113.86
112.80
111.83
104.17
NC
O
OH
OH
OH
O
15
^{13}C NMR, 101 MHz, 298 K, DMSO-d$_6$
f1 (ppm)

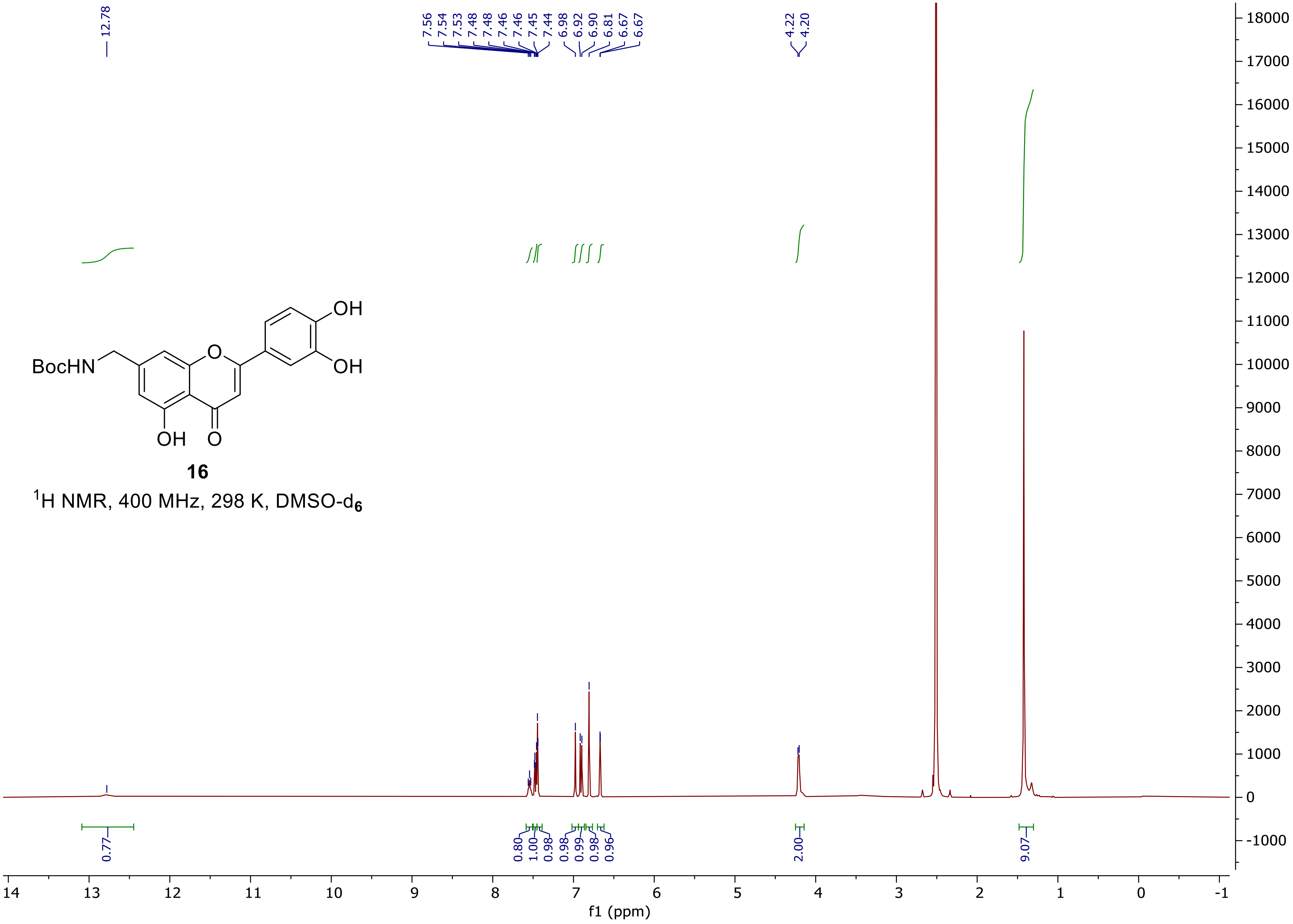

16

^{1}H NMR, 400 MHz, 298 K, DMSO-d_6

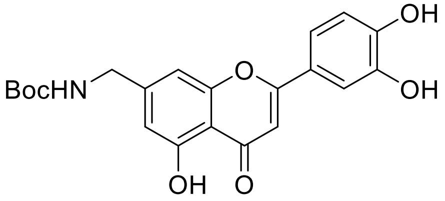

16

^{13}C NMR, 101 MHz, 273 K, DMSO-d_6

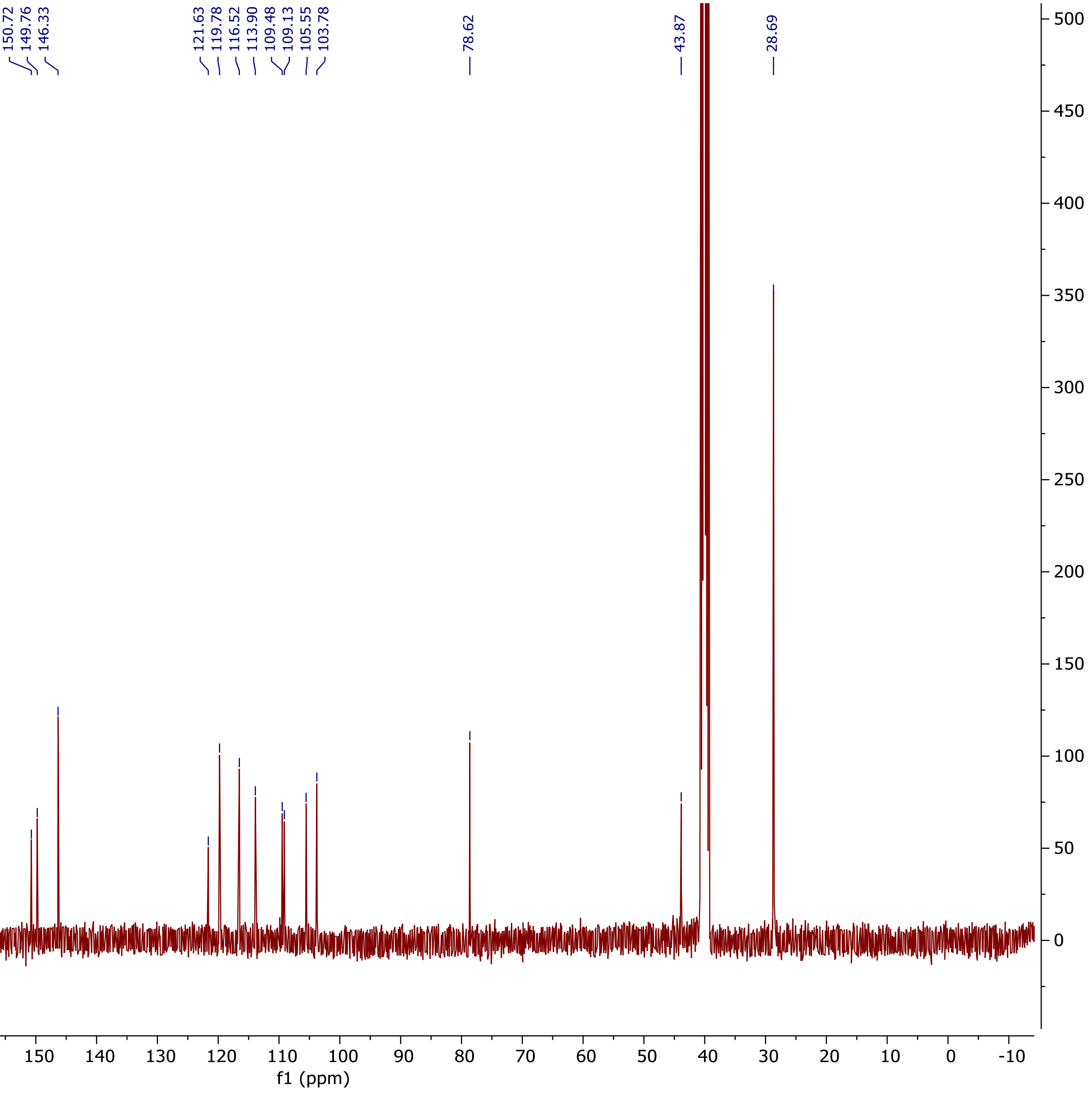

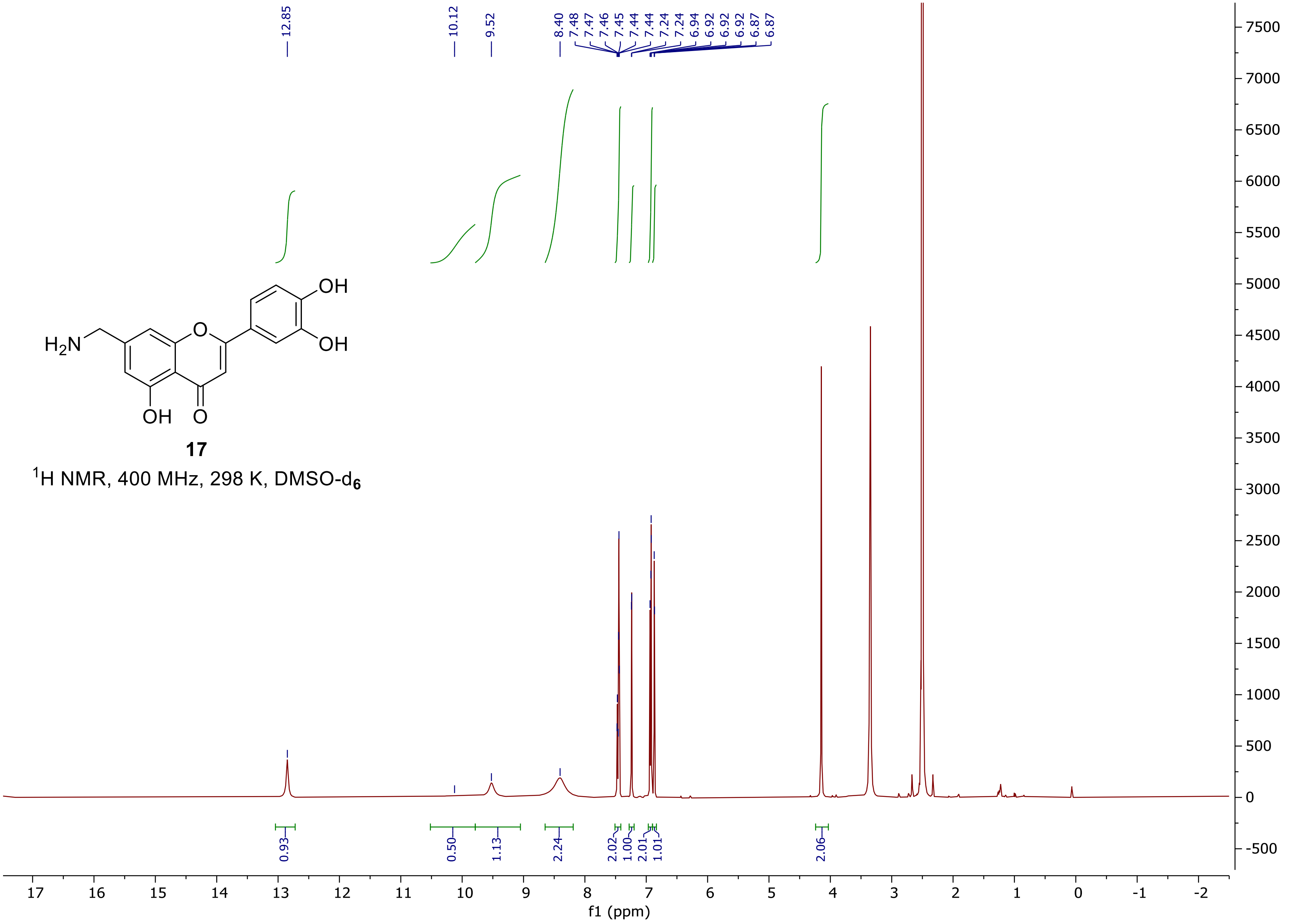
12.85
10.12
9.52
8.40
7.48
7.47
7.46
7.45
7.44
7.44
7.24
7.24
6.94
6.92
6.92
6.92
6.87
6.87
OH
OH
H2N
O
OH
O
17
1H NMR, 400 MHz, 298 K, DMSO-d6
0.93
0.50
1.13
2.24
2.02
1.00
2.01
1.01
2.06
f1 (ppm)

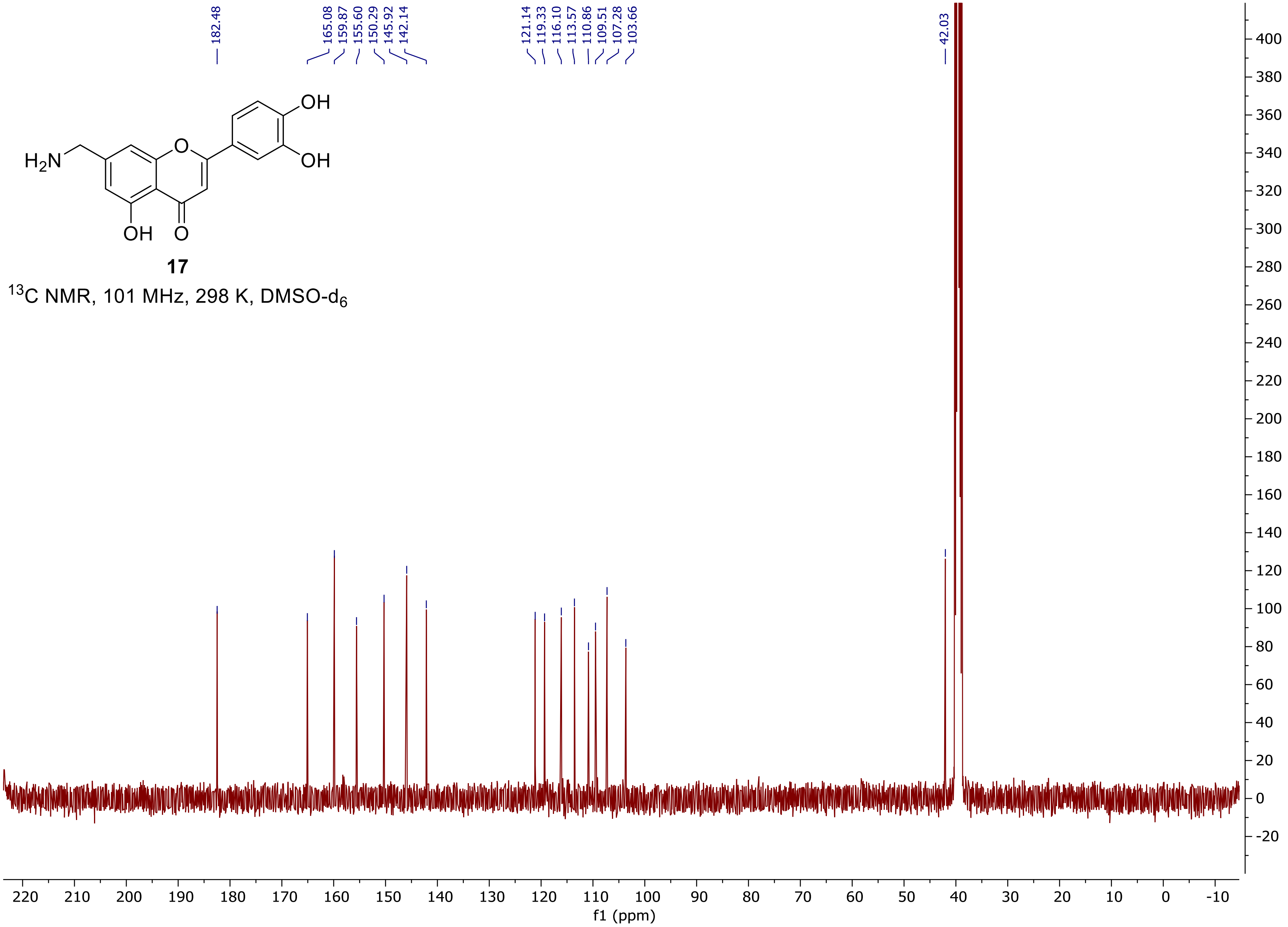

17

^{13}C NMR, 101 MHz, 298 K, DMSO-d_6

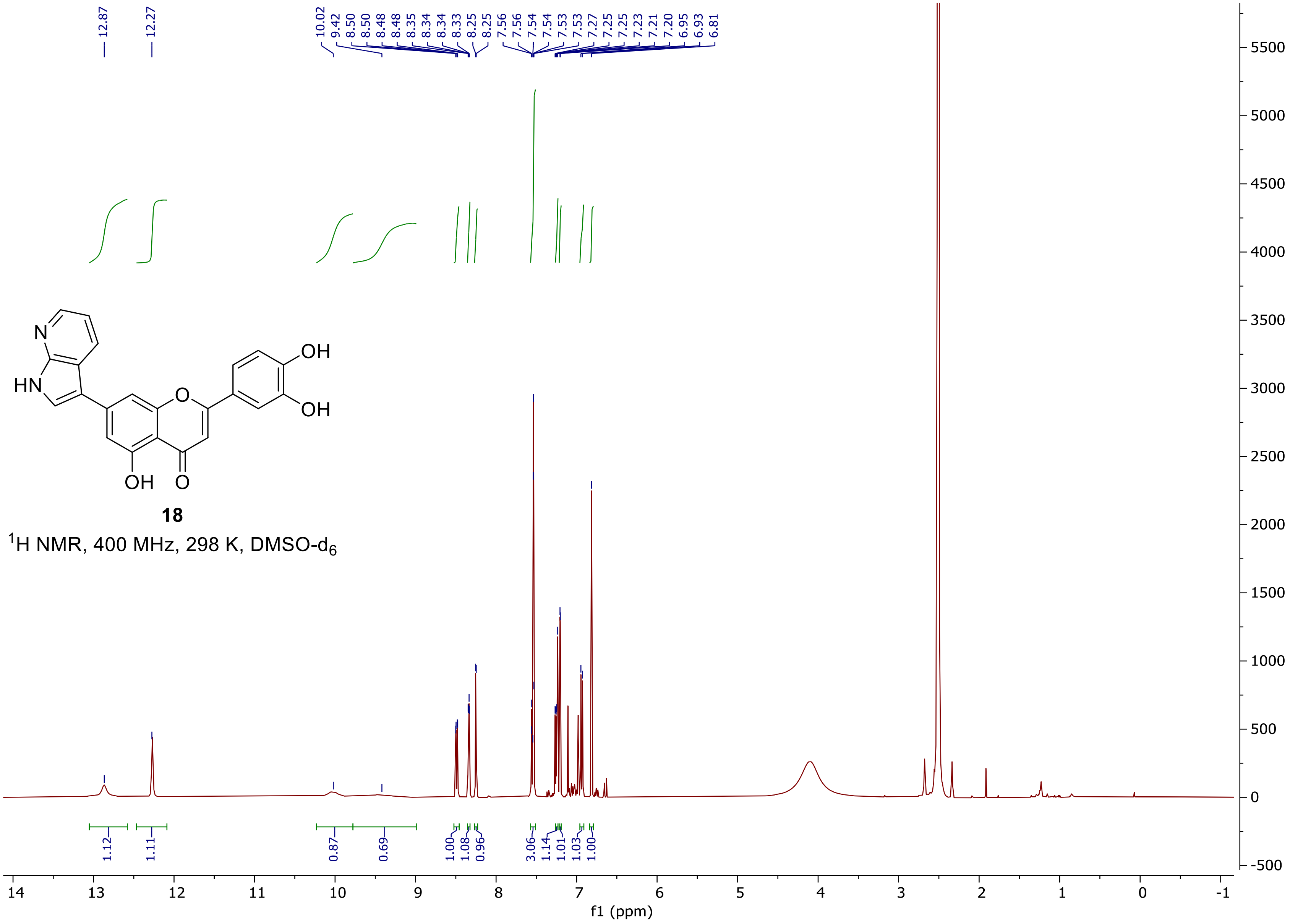
12.87
12.27
10.02
9.42
8.50
8.50
8.48
8.48
8.35
8.34
8.34
8.33
8.25
8.25
7.56
7.56
7.54
7.54
7.53
7.53
7.27
7.25
7.25
7.23
7.21
7.20
6.95
6.93
6.81
18
1H NMR, 400 MHz, 298 K, DMSO-d6
1.12
1.11
0.87
0.69
1.00
1.08
0.96
3.06
1.14
1.01
1.03
1.00
f1 (ppm)

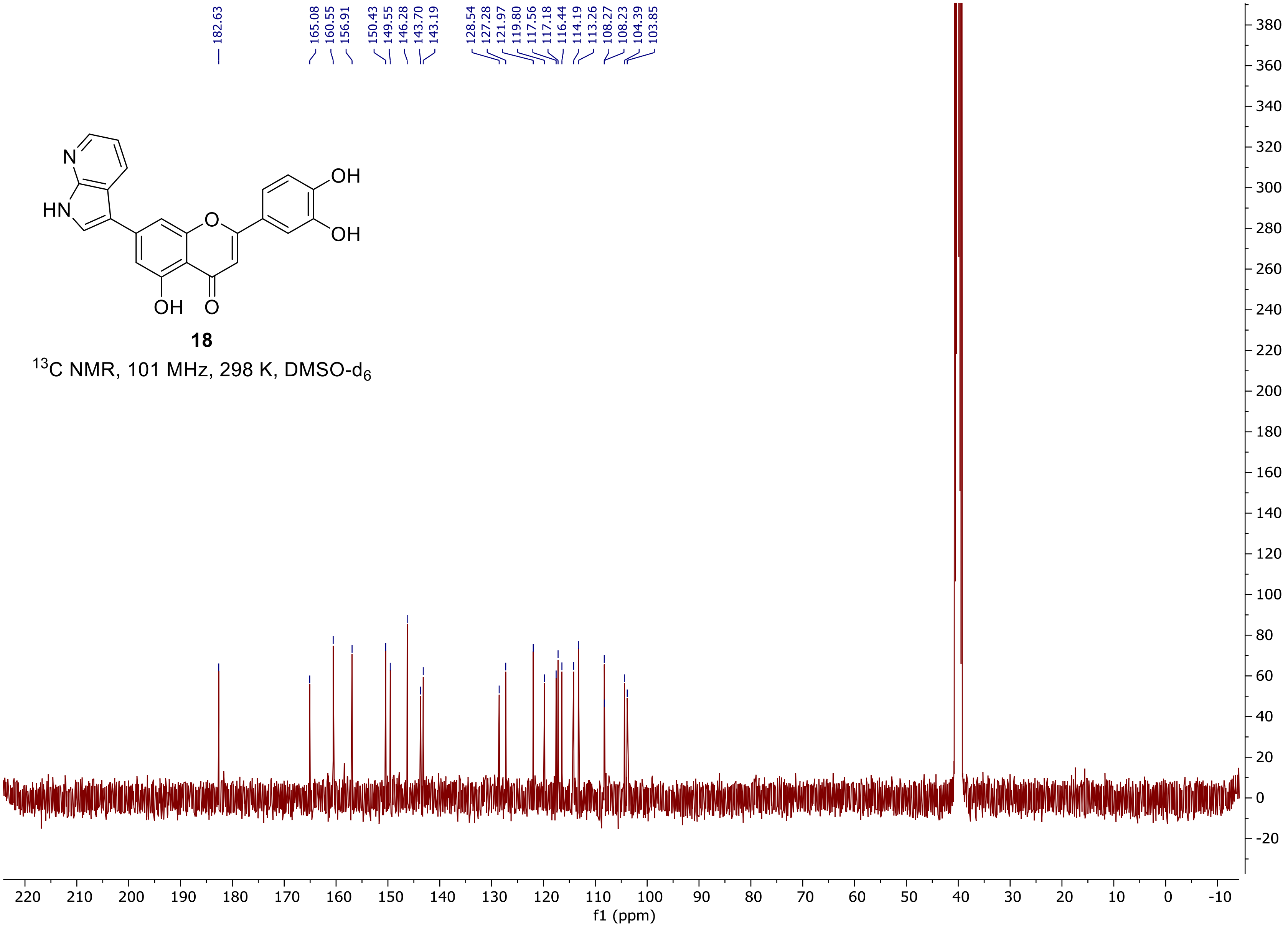

18

^{13}C NMR, 101 MHz, 298 K, DMSO-d_6

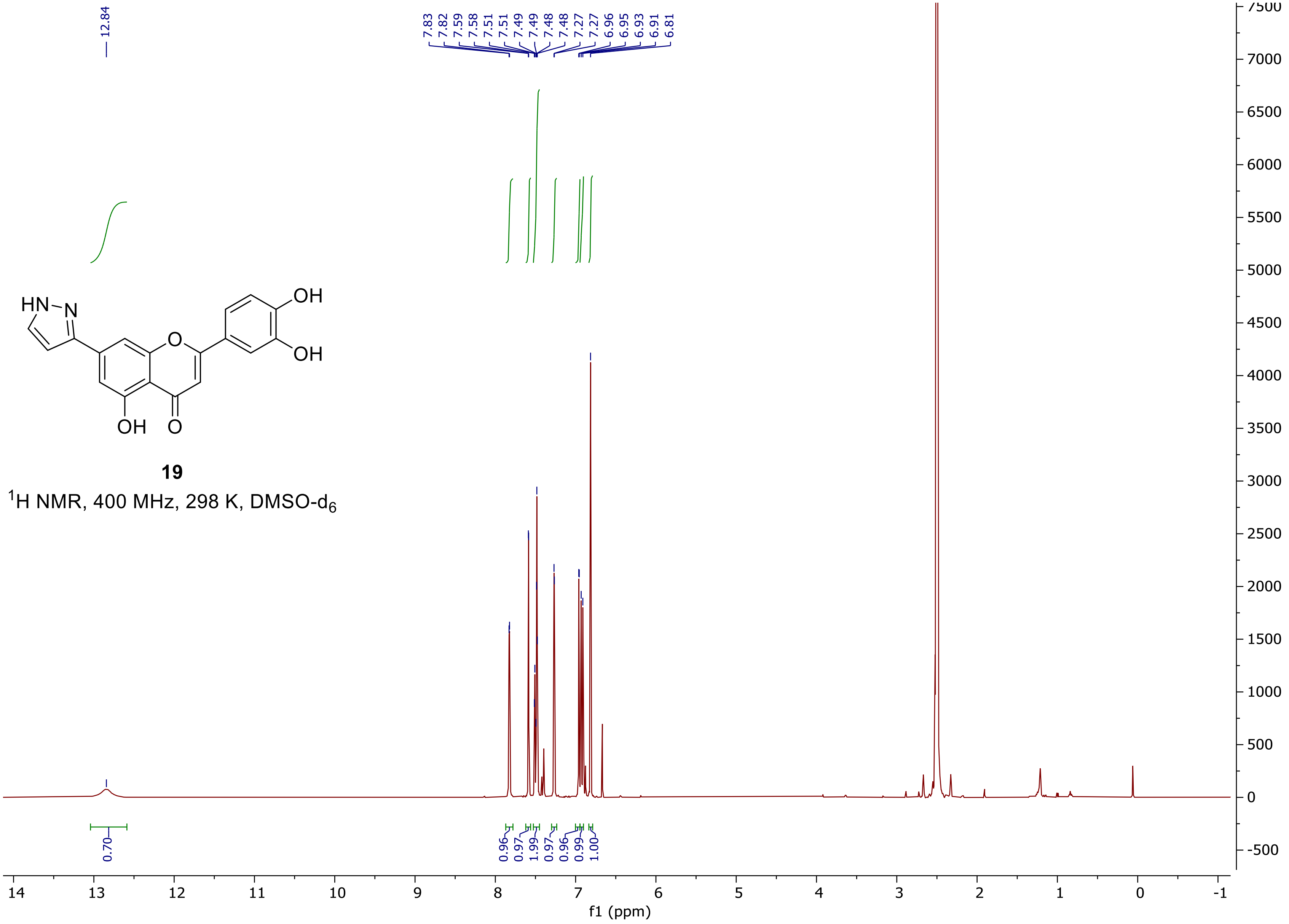
12.84
7.83
7.82
7.59
7.58
7.51
7.51
7.49
7.49
7.48
7.48
7.27
7.27
6.96
6.95
6.93
6.91
6.81
HN
N
O
OH
OH
OH
O
19
^{1}H NMR, 400 MHz, 298 K, DMSO-d$_6$
0.70
0.96
0.97
1.99
0.97
0.96
0.99
1.00
f1 (ppm)

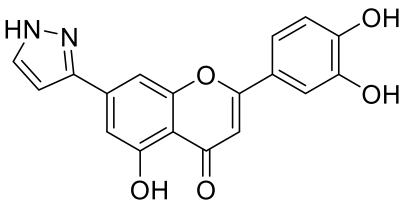

19

^{13}C NMR, 101 MHz, 298 K, DMSO-d_6

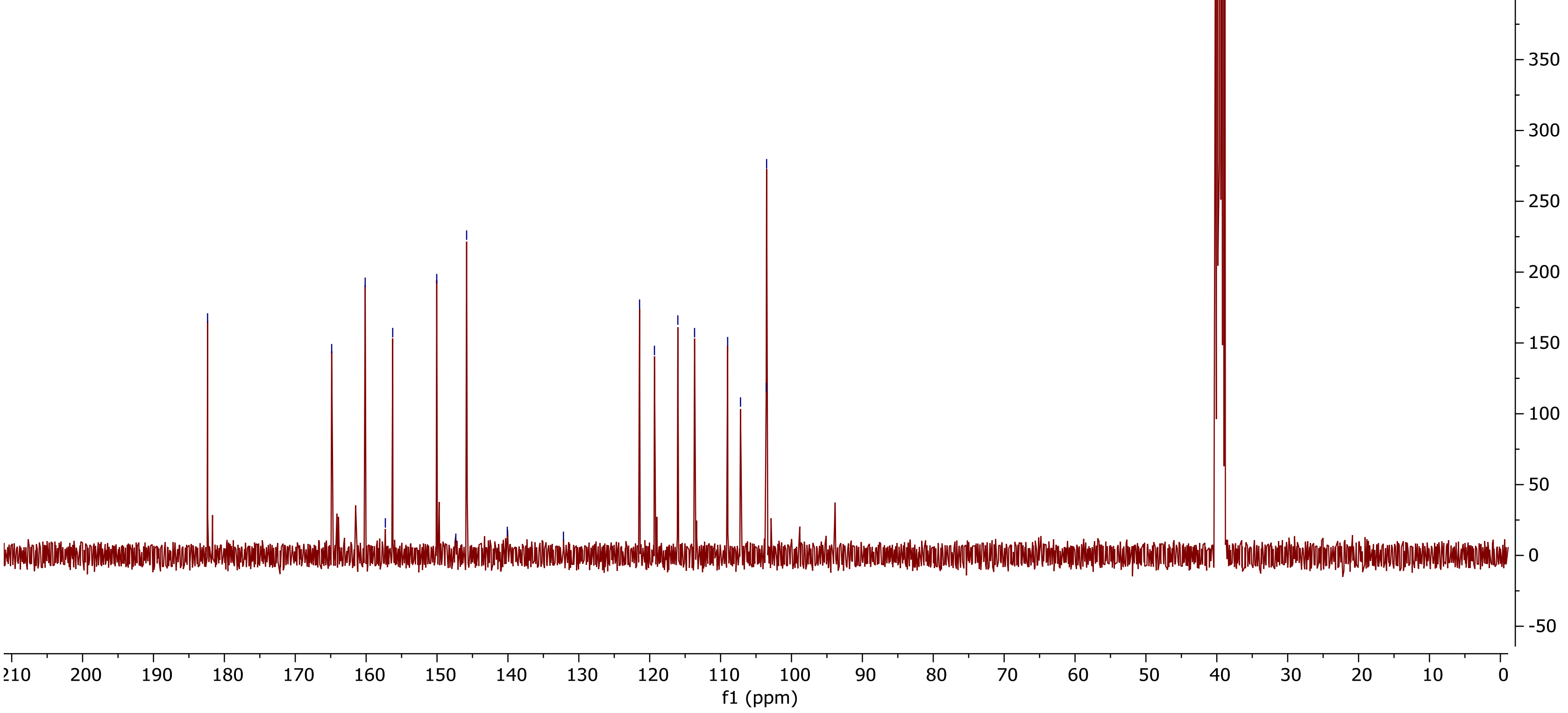

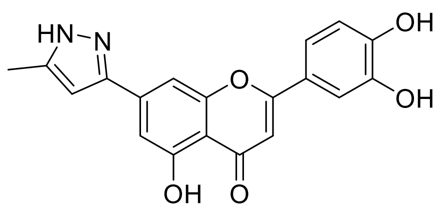

20

^{1}H NMR, 400 MHz, 298 K, DMSO-d$_6$

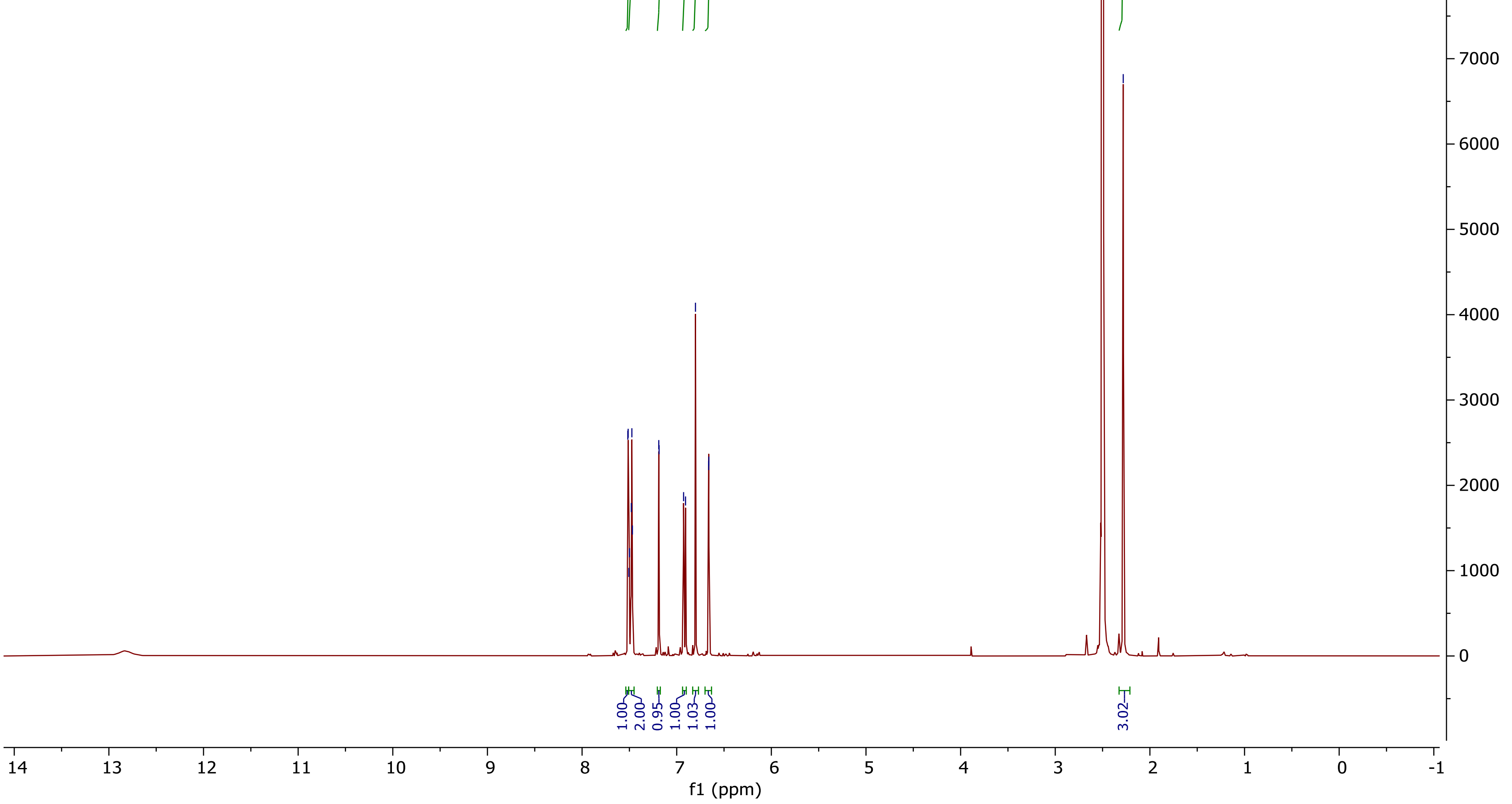

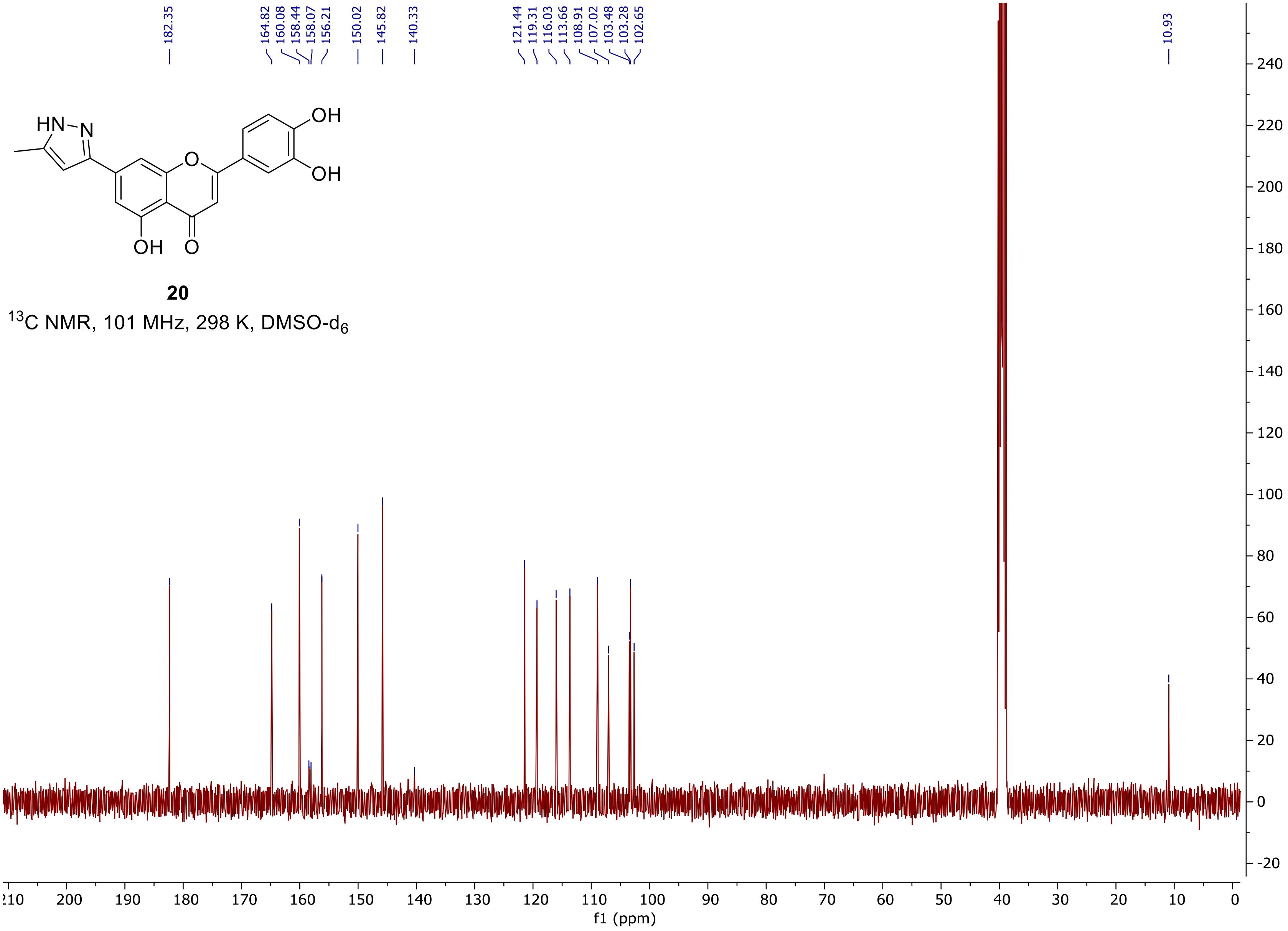

20

^{13}C NMR, 101 MHz, 298 K, DMSO-d$_6$

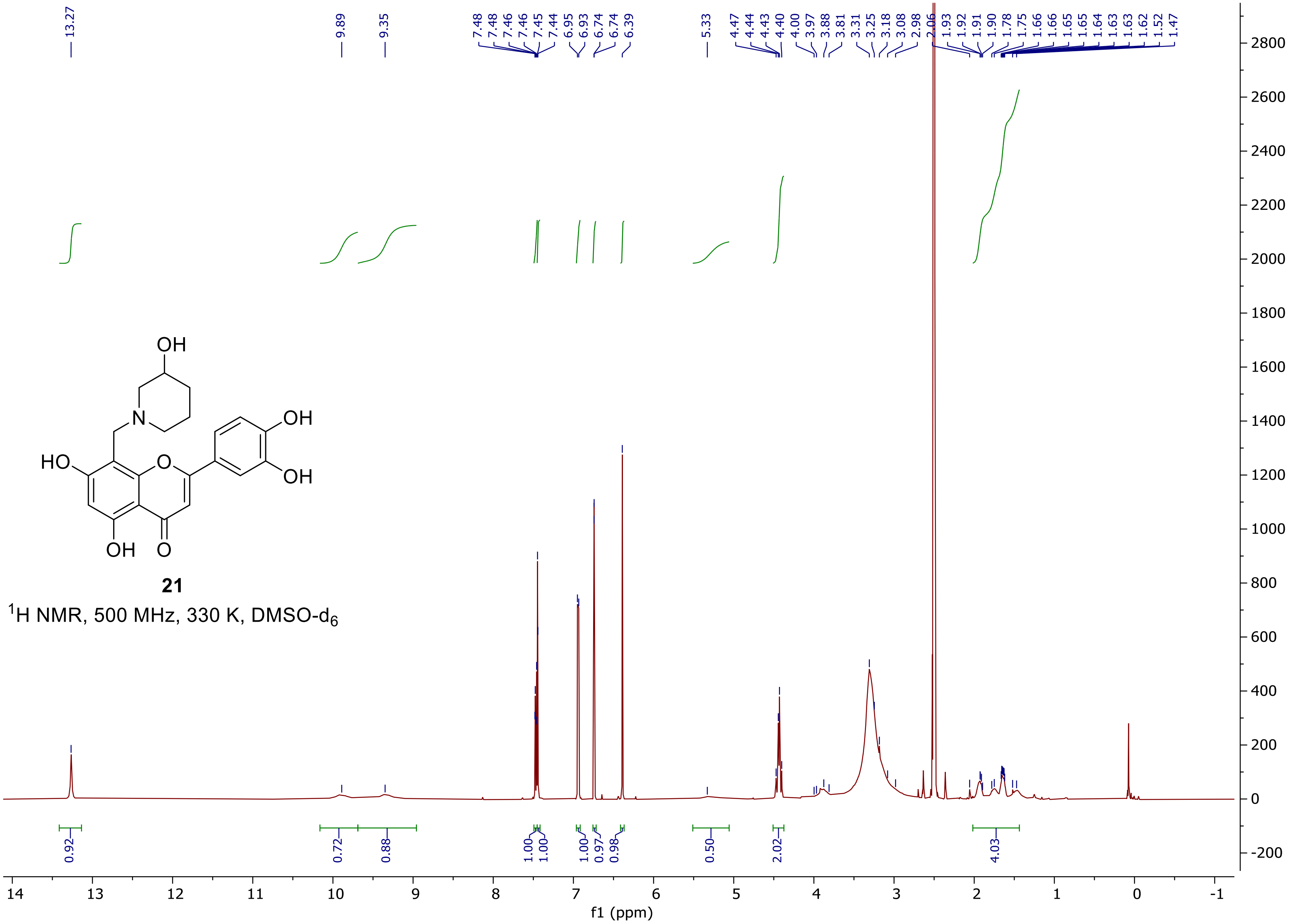

21

^{1}H NMR, 500 MHz, 330 K, DMSO-d_6

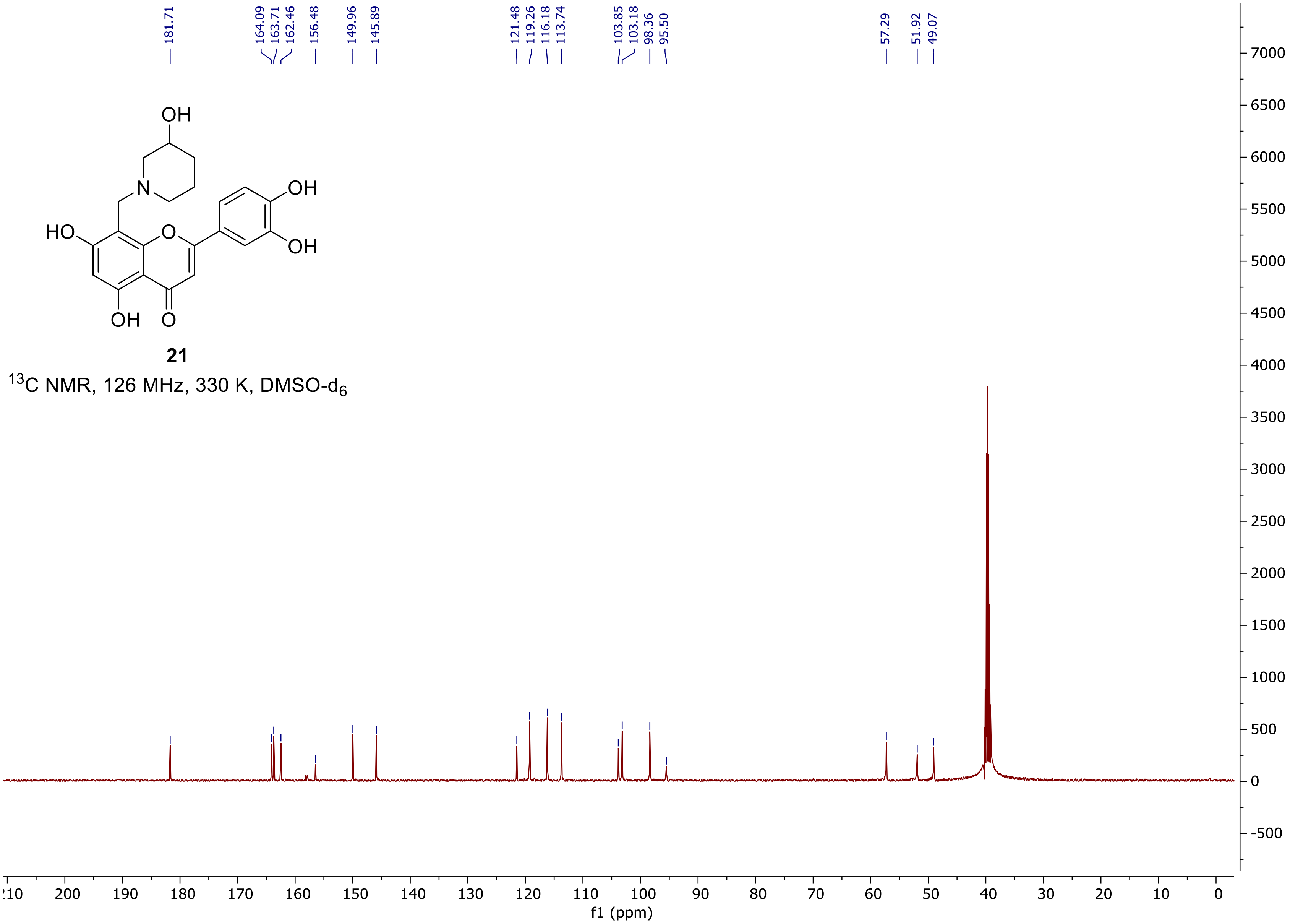

21

^{13}C NMR, 126 MHz, 330 K, DMSO-d_6

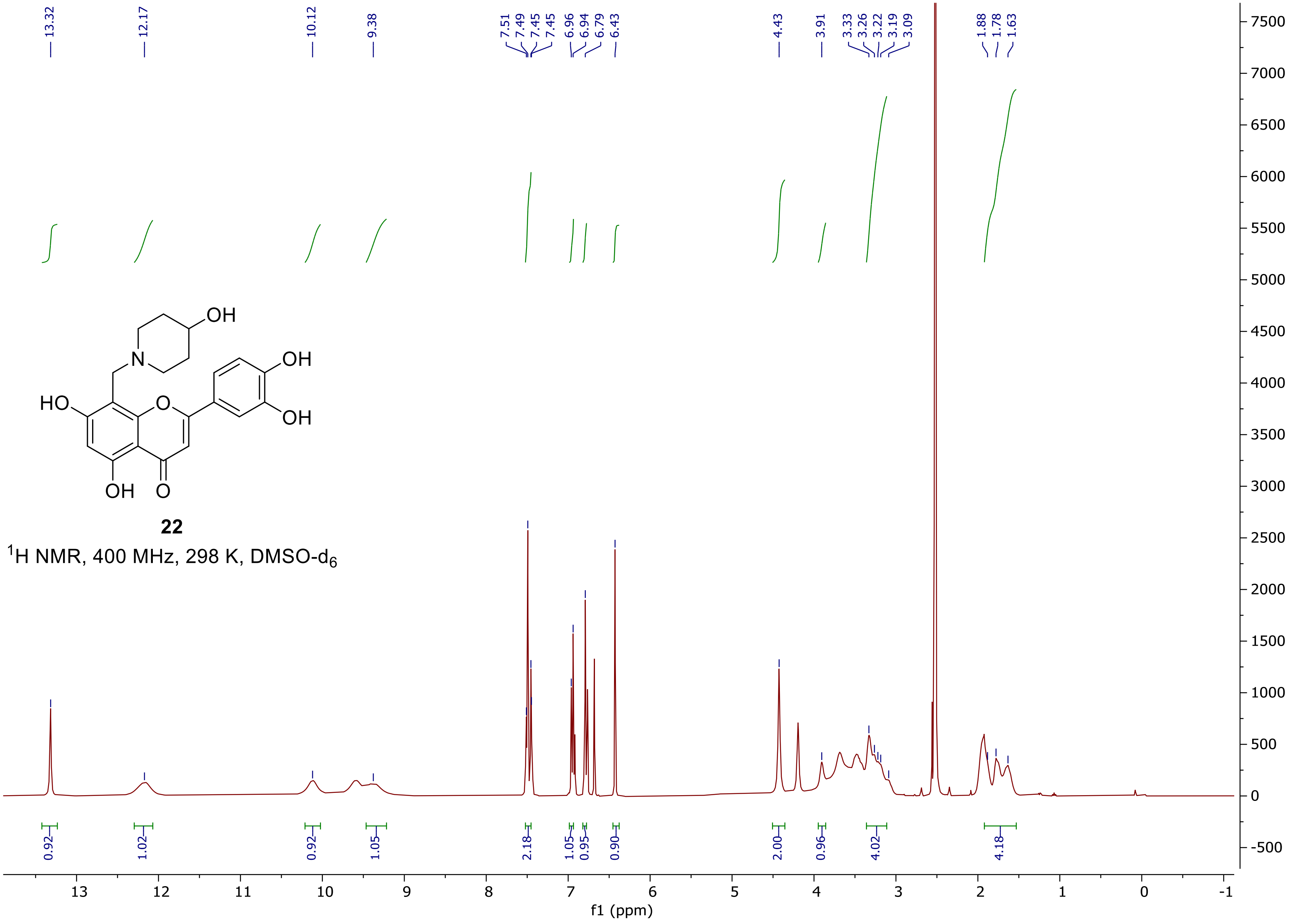

13.32
12.17
10.12
9.38
7.51
7.49
7.45
7.45
6.96
6.94
6.79
6.43
4.43
3.91
3.33
3.26
3.22
3.19
3.09
1.88
1.78
1.63
OH
N
OH
HO
O
OH
OH
O
22
¹H NMR, 400 MHz, 298 K, DMSO-d6
0.92
1.02
0.92
1.05
2.18
1.05
0.95
0.90
2.00
0.96
4.02
4.18
f1 (ppm)

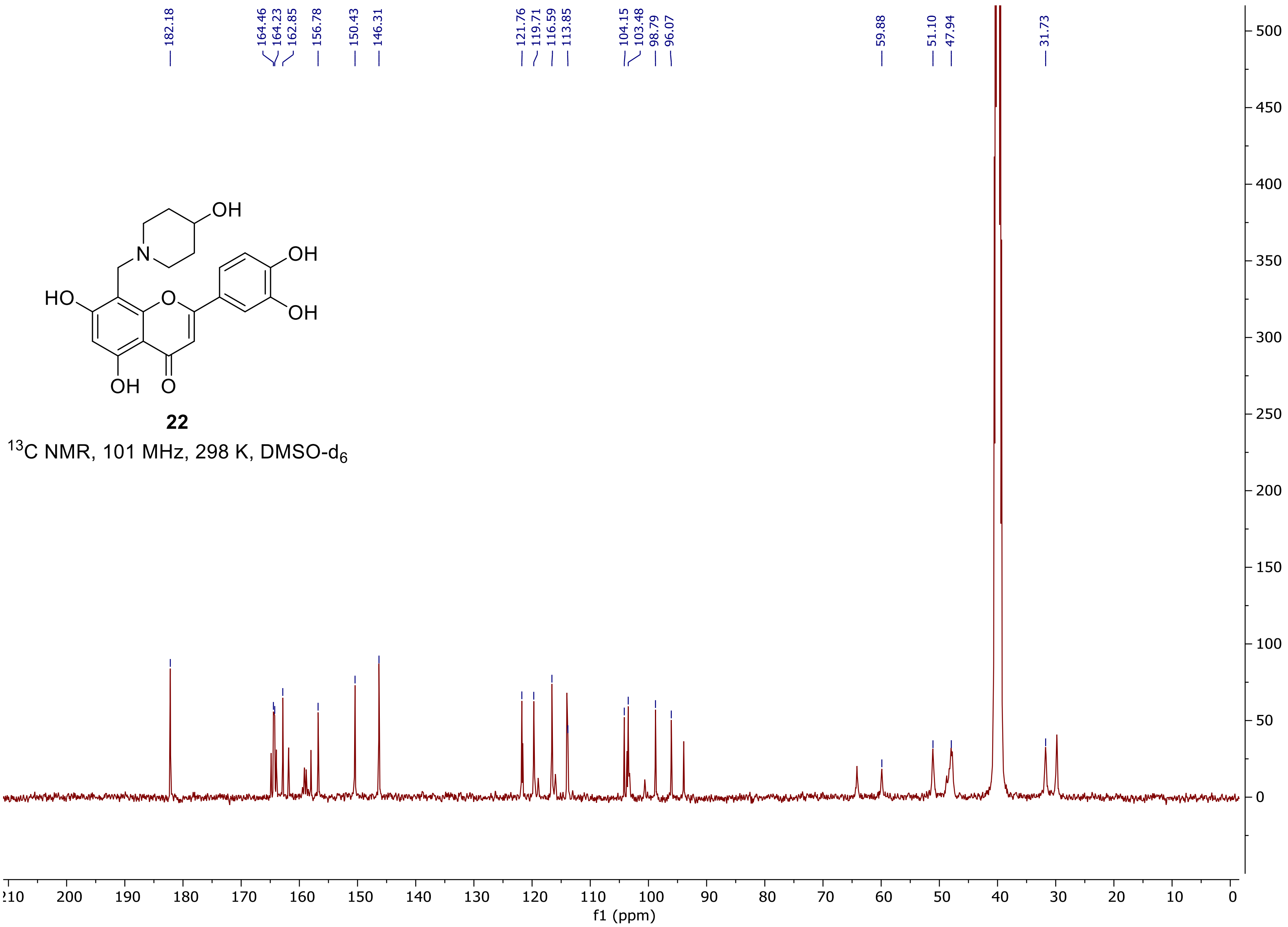

22

^{13}C NMR, 101 MHz, 298 K, DMSO-d$_6$

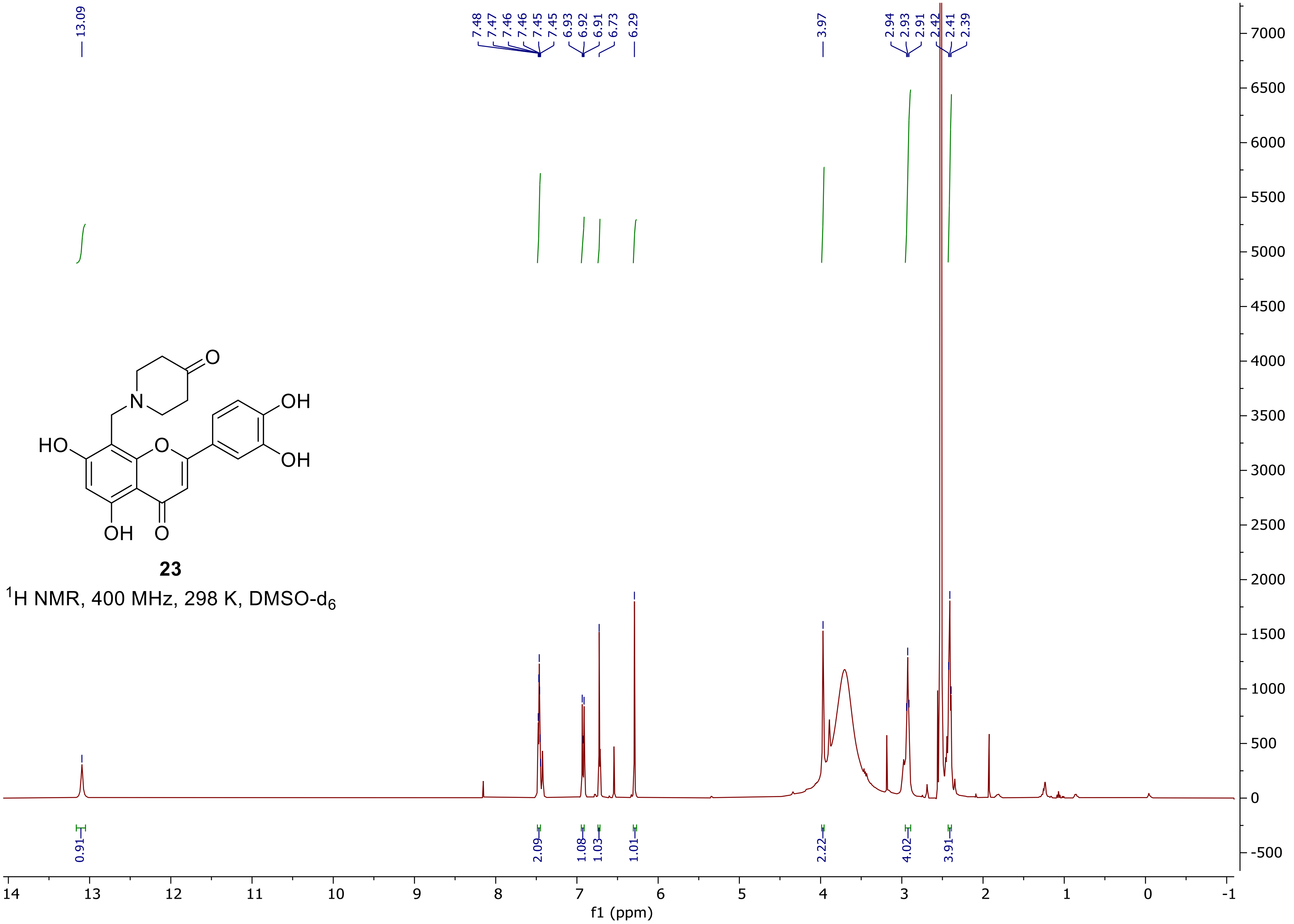

23

^{1}H NMR, 400 MHz, 298 K, DMSO-d_6

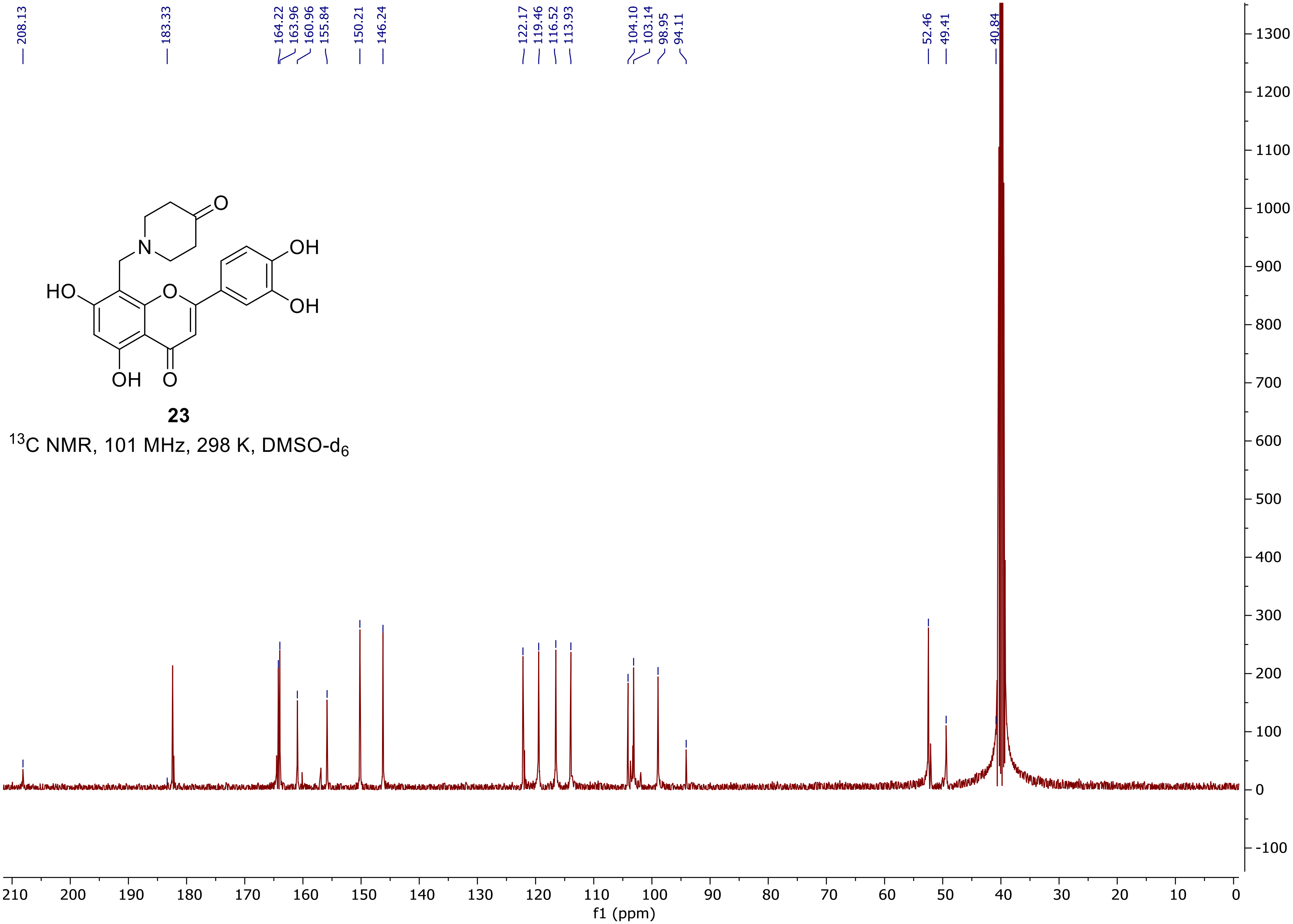

23

^{13}C NMR, 101 MHz, 298 K, DMSO-d_6

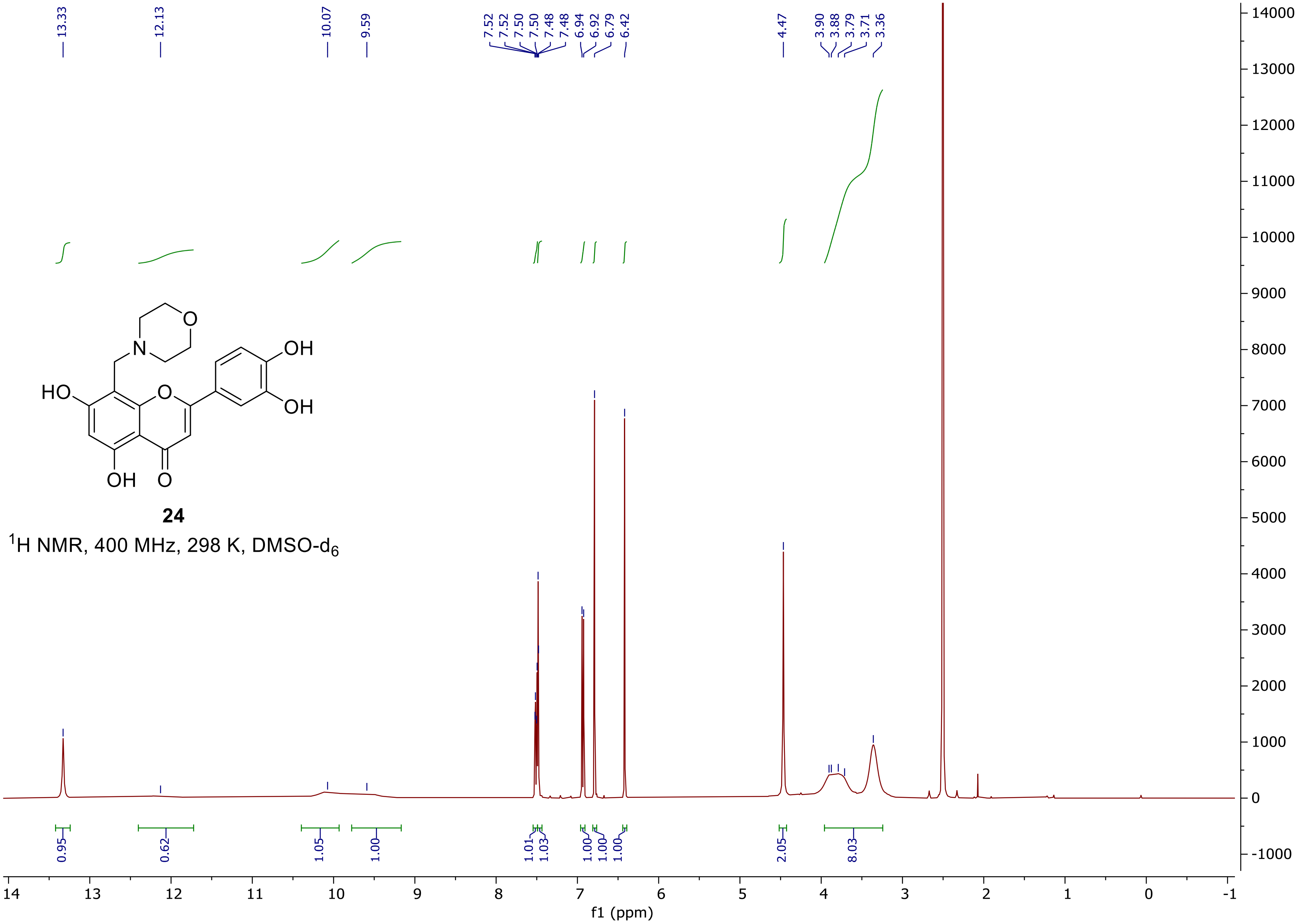

24

^{1}H NMR, 400 MHz, 298 K, DMSO-d_6

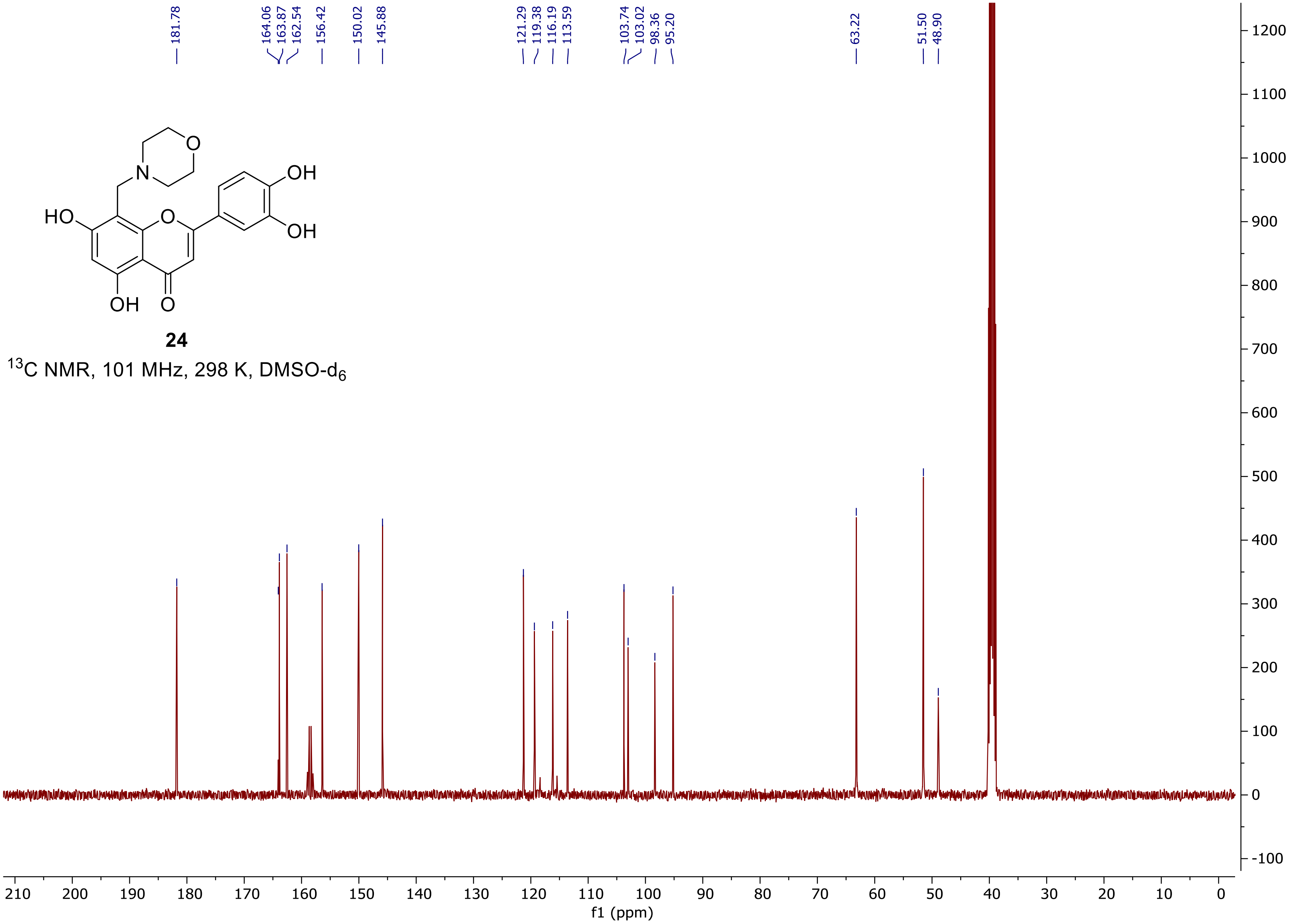
O
N
OH
HO
O
OH
OH
O
24
^{13}C NMR, 101 MHz, 298 K, DMSO-d_6
181.78
164.06
163.87
162.54
156.42
150.02
145.88
121.29
119.38
116.19
113.59
103.74
103.02
98.36
95.20
63.22
51.50
48.90
f1 (ppm)

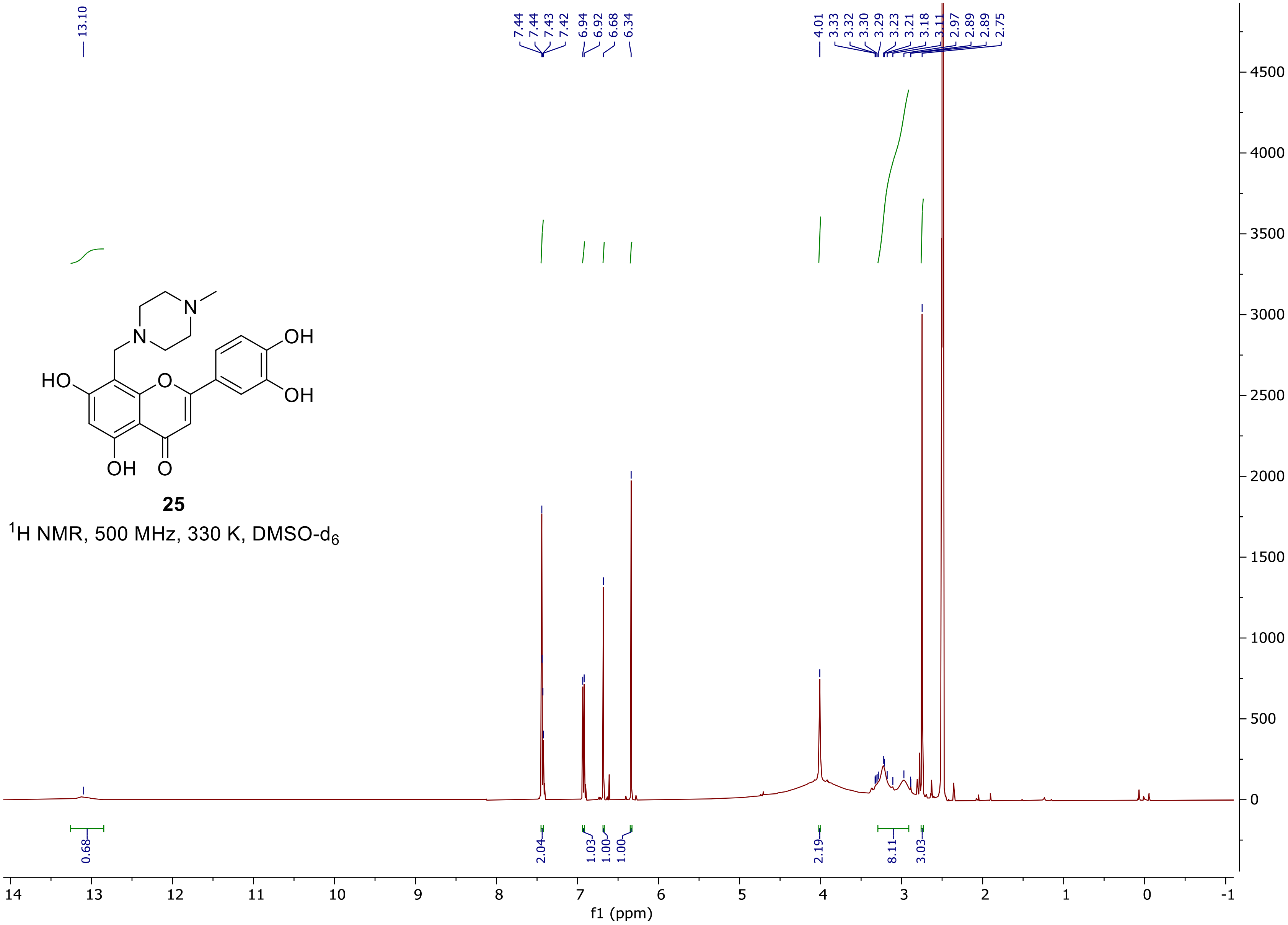

25

^{1}H NMR, 500 MHz, 330 K, DMSO-d_6

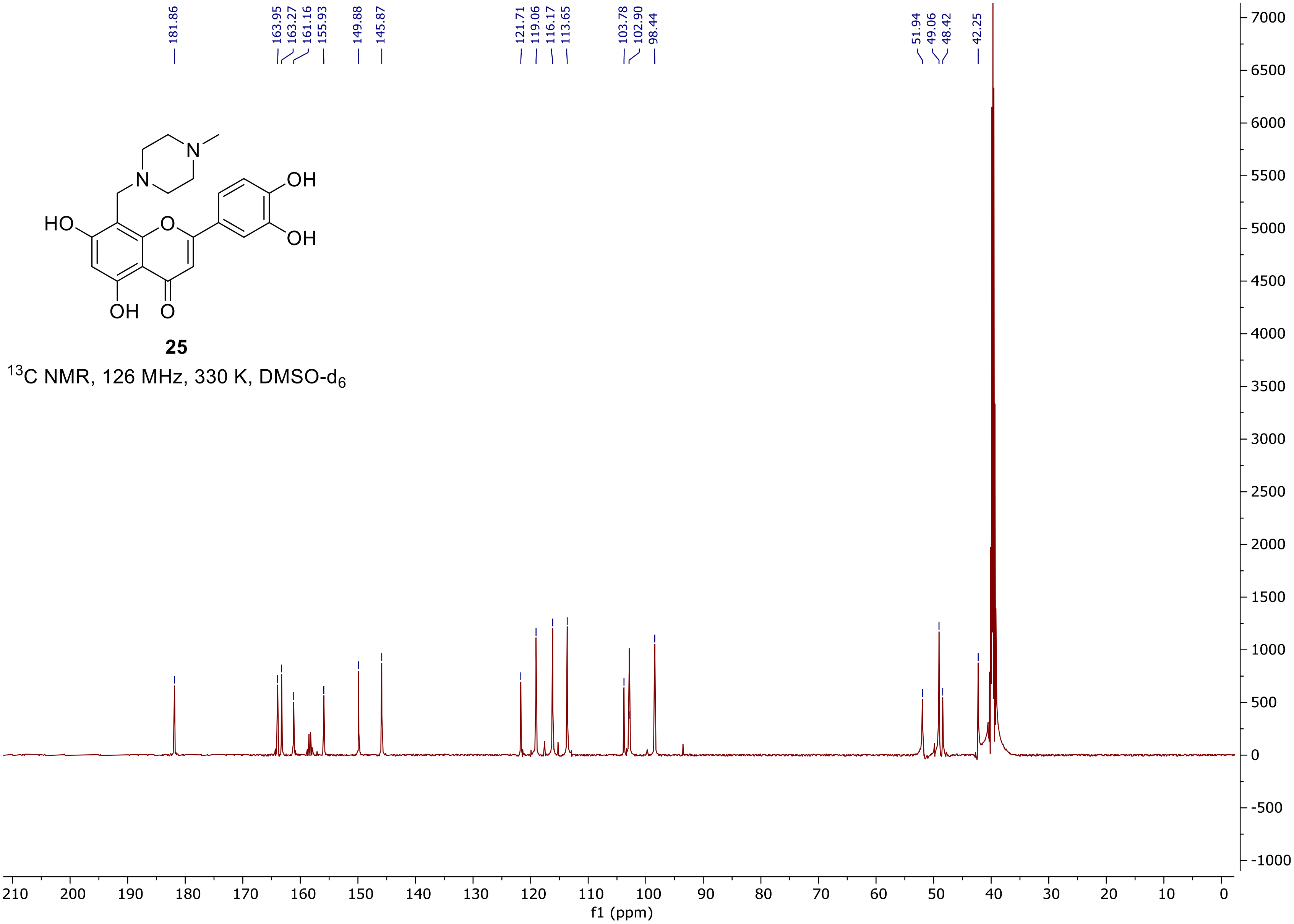

N
N
HO
O
OH
OH
OH
O
25
13C NMR, 126 MHz, 330 K, DMSO-d6
181.86
163.95
163.27
161.16
155.93
149.88
145.87
121.71
119.06
116.17
113.65
103.78
102.90
98.44
51.94
49.06
48.42
42.25
7000
6500
6000
5500
5000
4500
4000
3500
3000
2500
2000
1500
1000
500
0
-500
-1000
210
200
190
180
170
160
150
140
130
120
110
100
90
80
70
60
50
40
30
20
10
0
f1 (ppm)

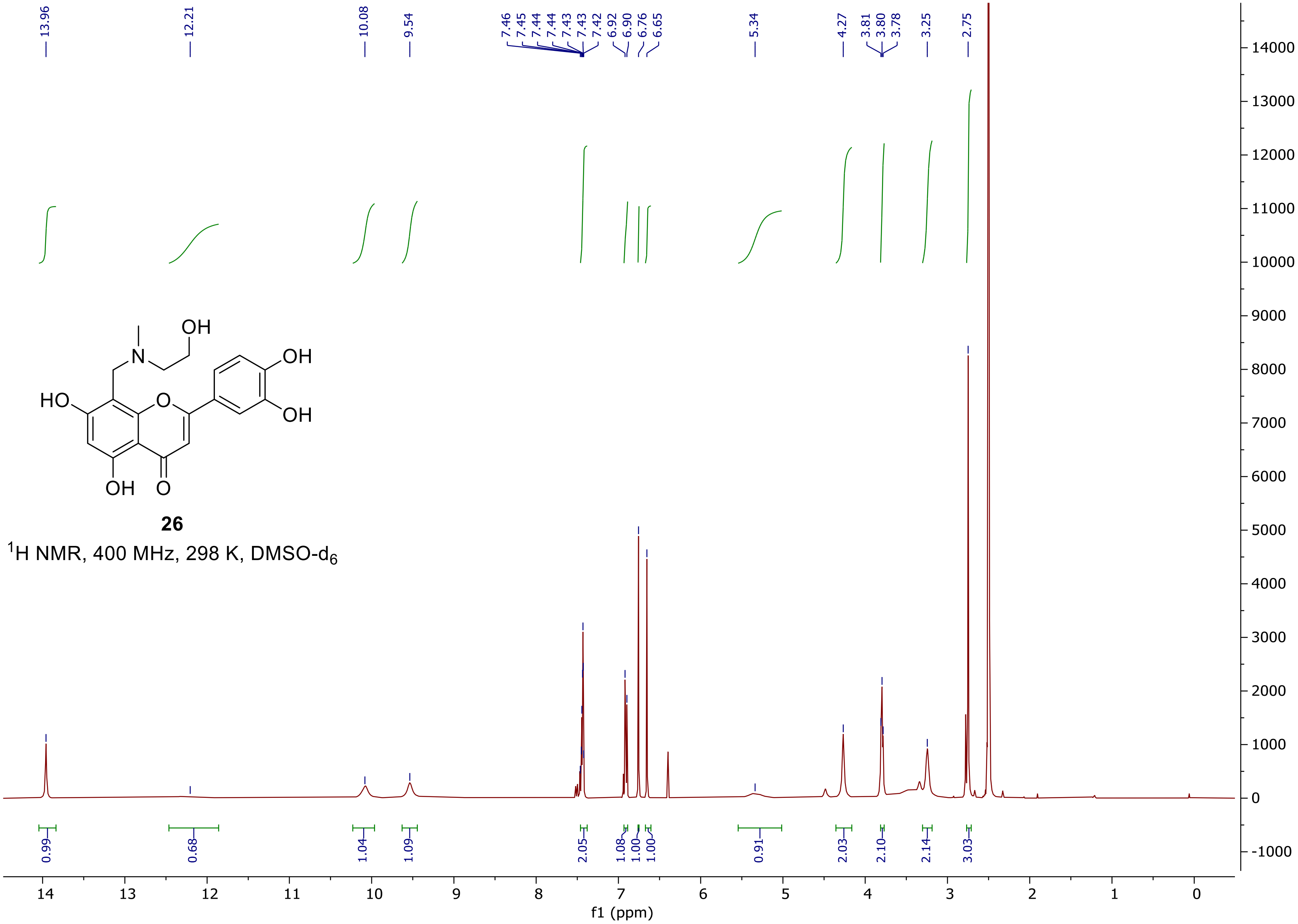
13.96
12.21
10.08
9.54
7.46
7.45
7.44
7.44
7.43
7.43
7.42
6.92
6.90
6.76
6.65
5.34
4.27
3.81
3.80
3.78
3.25
2.75
OH
N
OH
HO
O
OH
OH
OH
O
26
^{1}H NMR, 400 MHz, 298 K, DMSO-d$_6$
0.99
0.68
1.04
1.09
2.05
1.08
1.00
1.00
0.91
2.03
2.10
2.14
3.03
f1 (ppm)
14 13 12 11 10 9 8 7 6 5 4 3 2 1 0
14000 13000 12000 11000 10000 9000 8000 7000 6000 5000 4000 3000 2000 1000 0 -1000

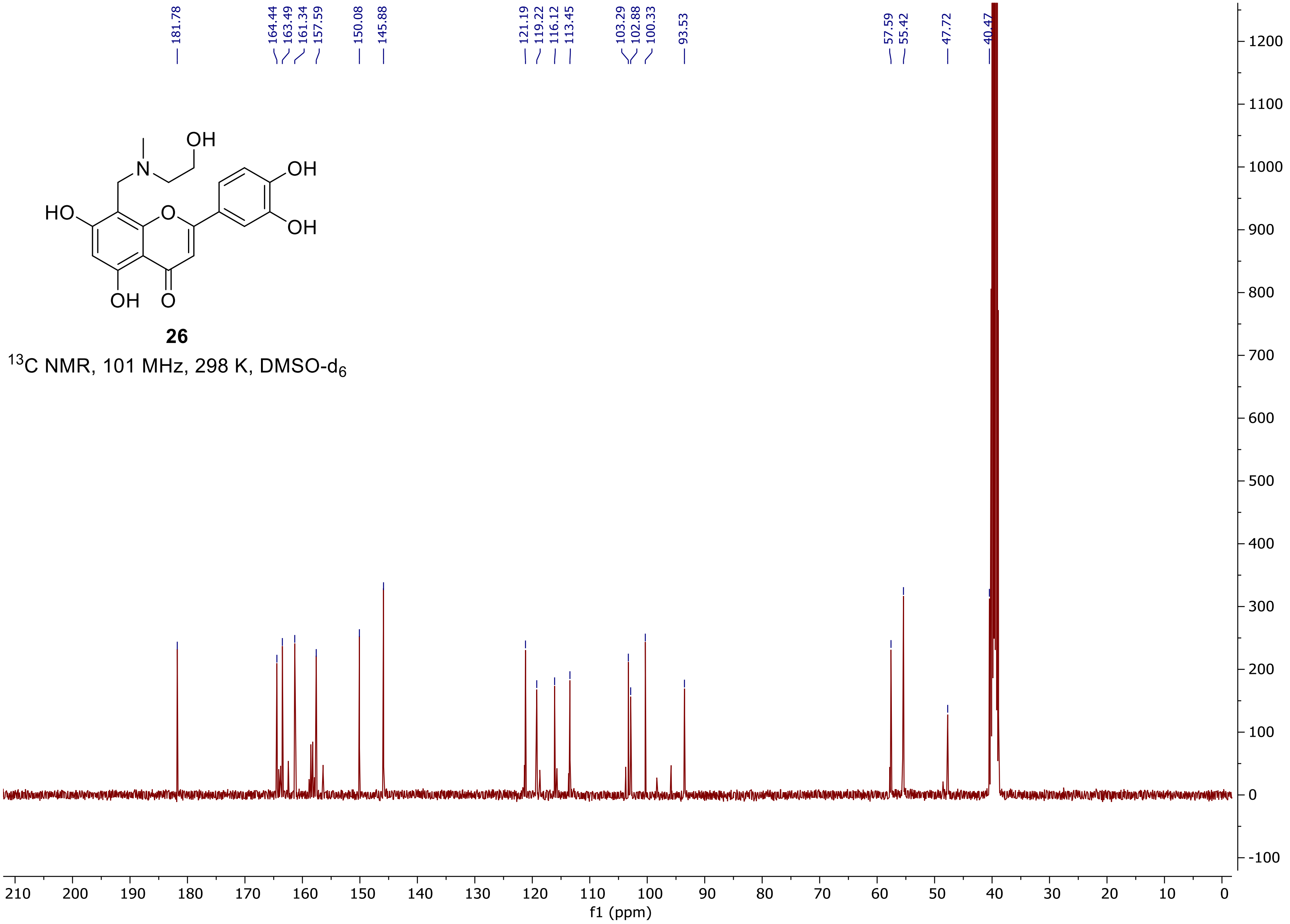

181.78
164.44
163.49
161.34
157.59
150.08
145.88
121.19
119.22
116.12
113.45
103.29
102.88
100.33
93.53
57.59
55.42
47.72
40.47
OH
N
OH
HO
O
OH
OH
O
26
^{13}C NMR, 101 MHz, 298 K, DMSO-d$_6$
f1 (ppm)

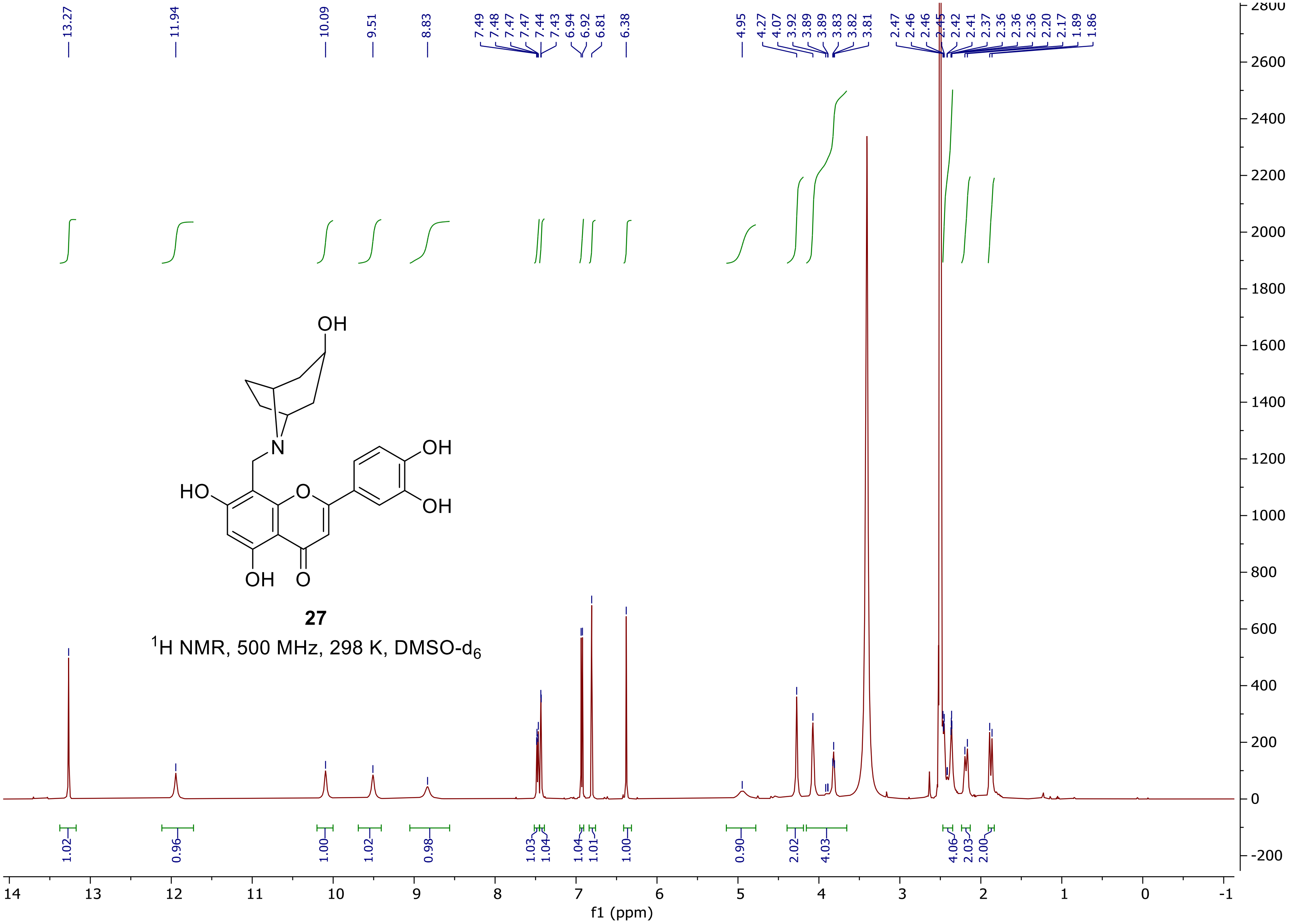
13.27
11.94
10.09
9.51
8.83
7.49
7.48
7.47
7.47
7.44
7.43
6.94
6.92
6.81
6.38
4.95
4.27
4.07
3.92
3.89
3.89
3.83
3.82
3.81
2.47
2.46
2.46
2.45
2.42
2.41
2.37
2.36
2.36
2.36
2.20
2.17
1.89
1.86
OH
N
HO
O
OH
OH
OH
O
27
1H NMR, 500 MHz, 298 K, DMSO-d6
1.02
0.96
1.00
1.02
0.98
1.03
1.04
1.04
1.01
1.00
0.90
2.02
4.03
4.06
2.03
2.00
f1 (ppm)

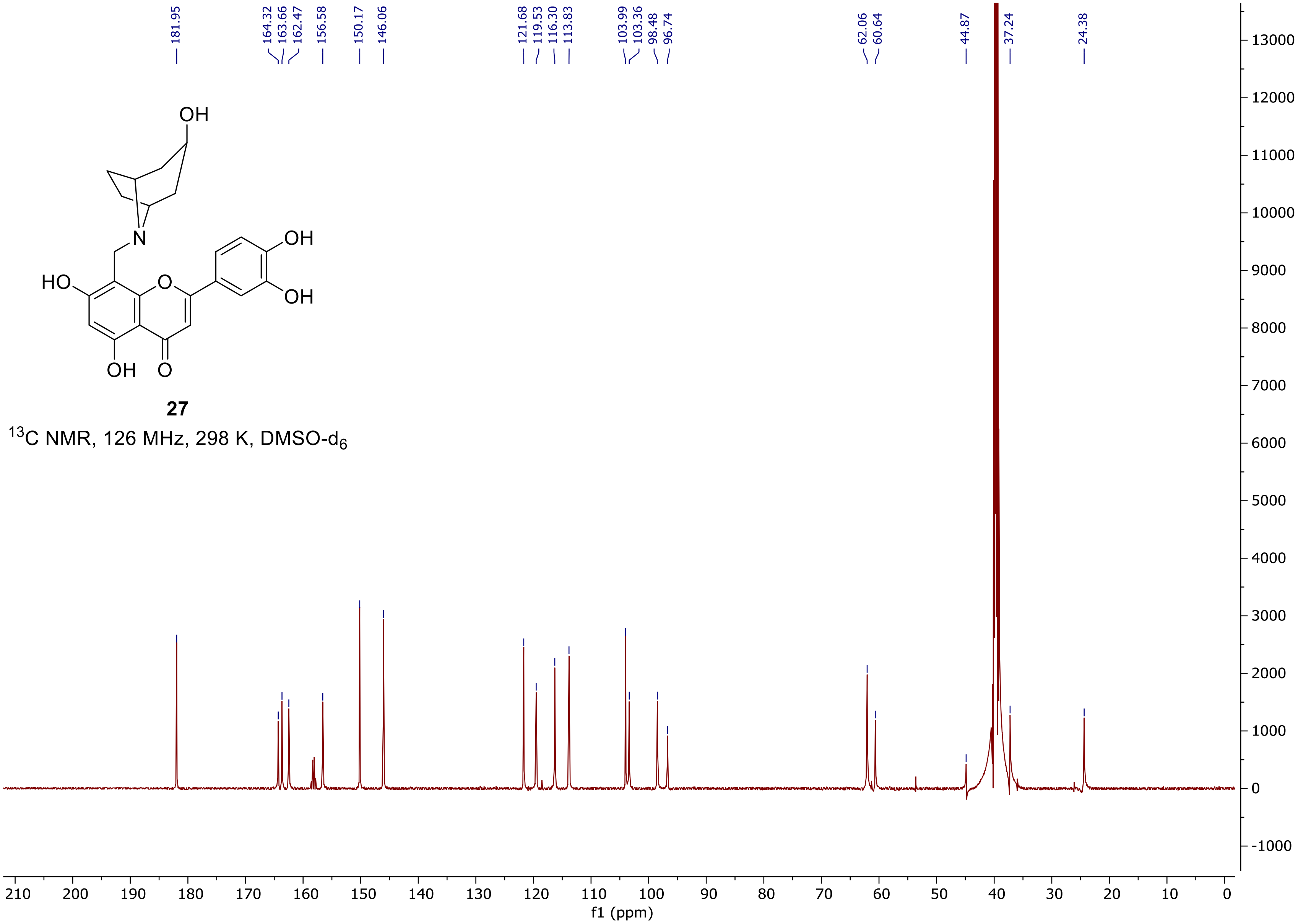
181.95
164.32
163.66
162.47
156.58
150.17
146.06
121.68
119.53
116.30
113.83
103.99
103.36
98.48
96.74
62.06
60.64
44.87
37.24
24.38
OH
N
OH
OH
HO
O
OH
O
27
^{13}C NMR, 126 MHz, 298 K, DMSO-d$_6$
13000
12000
11000
10000
9000
8000
7000
6000
5000
4000
3000
2000
1000
0
-1000
210 200 190 180 170 160 150 140 130 120 110 100 90 80 70 60 50 40 30 20 10 0
f1 (ppm)

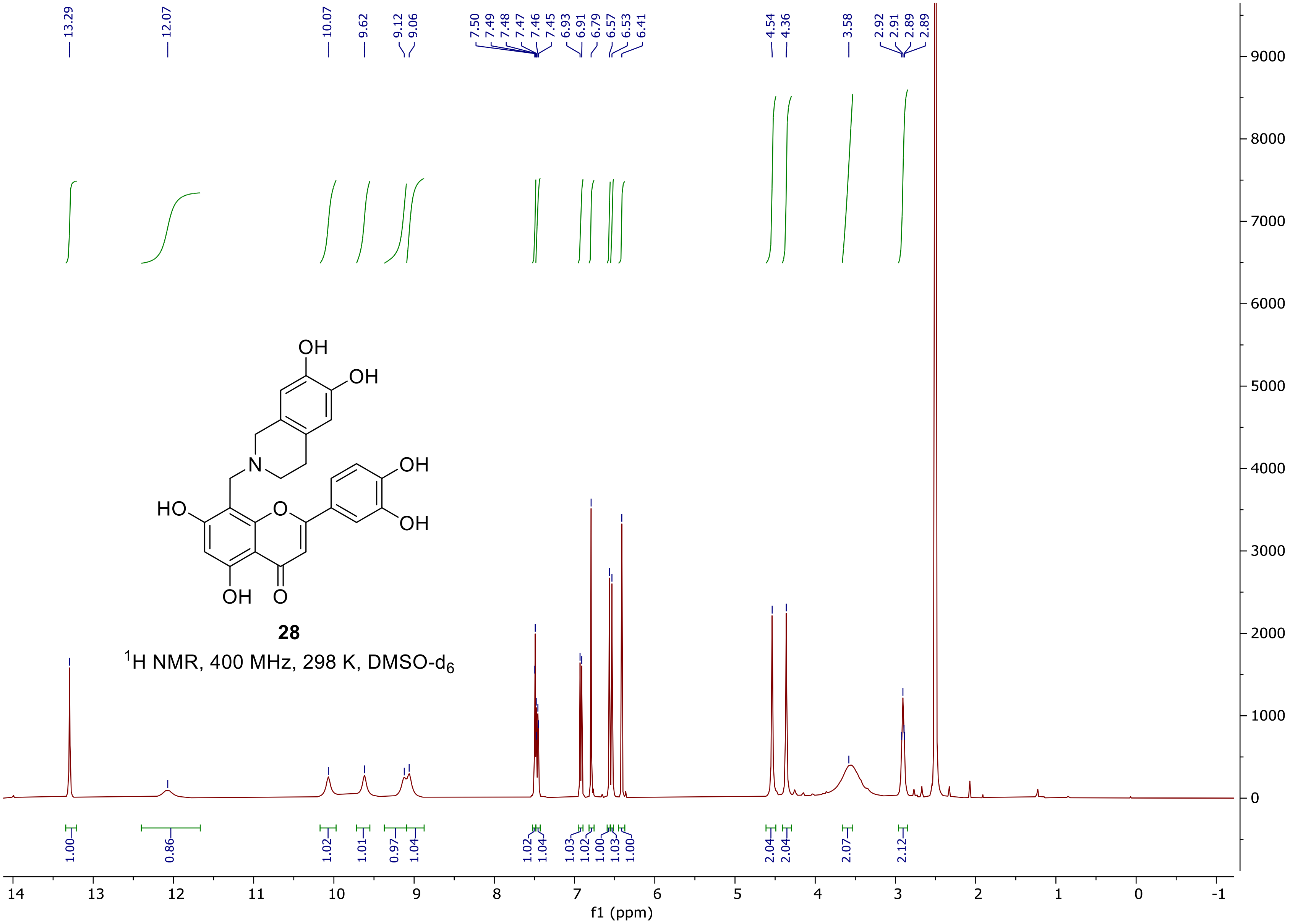

13.29
12.07
10.07
9.62
9.12
9.06
7.50
7.49
7.48
7.47
7.46
7.45
6.93
6.91
6.79
6.57
6.53
6.41
4.54
4.36
3.58
2.92
2.91
2.89
2.89
28
¹H NMR, 400 MHz, 298 K, DMSO-d6
1.00
0.86
1.02
1.01
0.97
1.04
1.02
1.04
1.03
1.02
1.00
1.03
1.00
2.04
2.04
2.07
2.12
f1 (ppm)

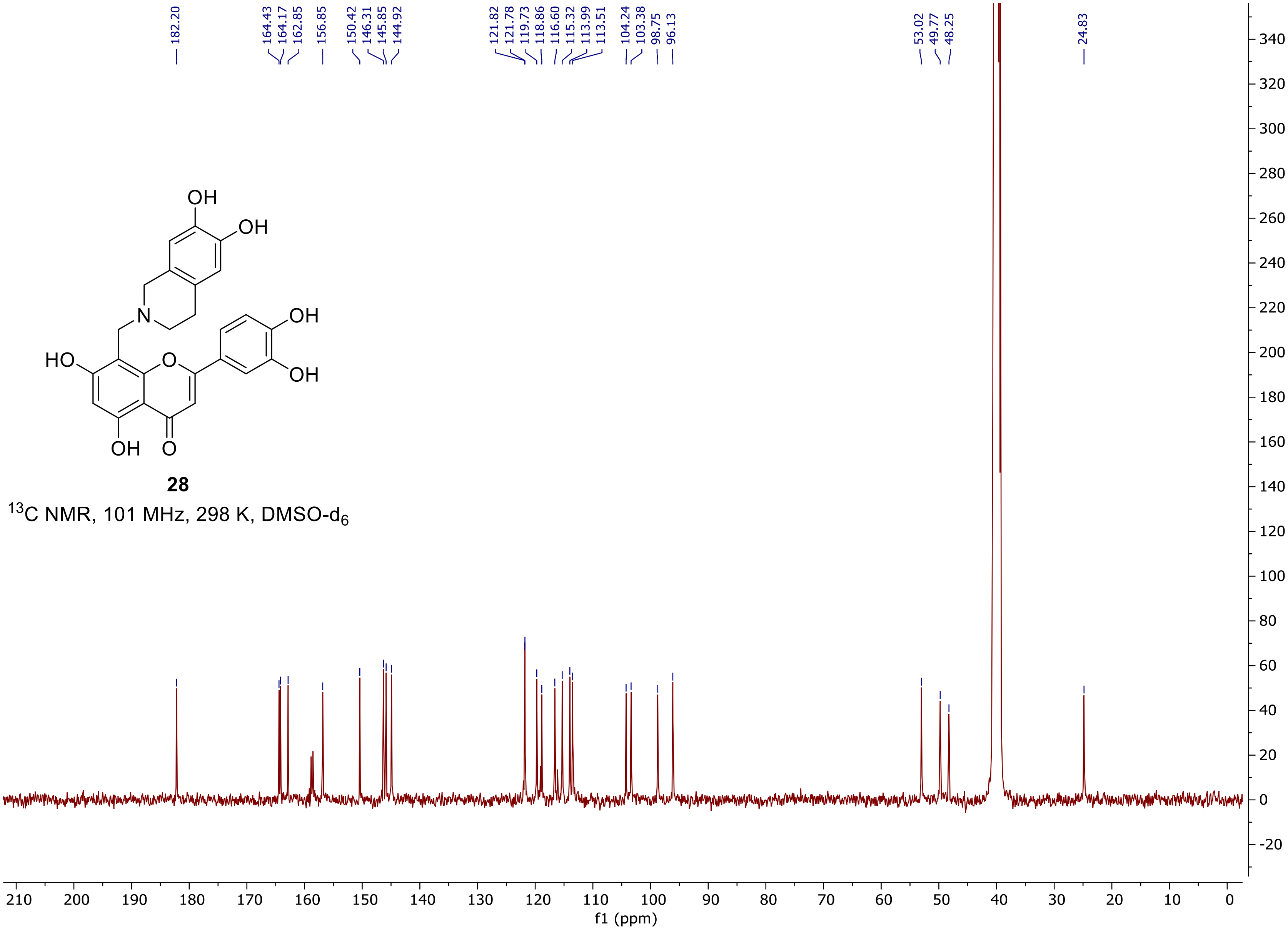
182.20
164.43
164.17
162.85
156.85
150.42
146.31
145.85
144.92
121.82
121.78
119.73
118.86
116.60
115.32
113.99
113.51
104.24
103.38
98.75
96.13
53.02
49.77
48.25
24.83
OH
OH
N
OH
HO
O
OH
OH
O
28
^{13}C NMR, 101 MHz, 298 K, DMSO-d_6
f1 (ppm)

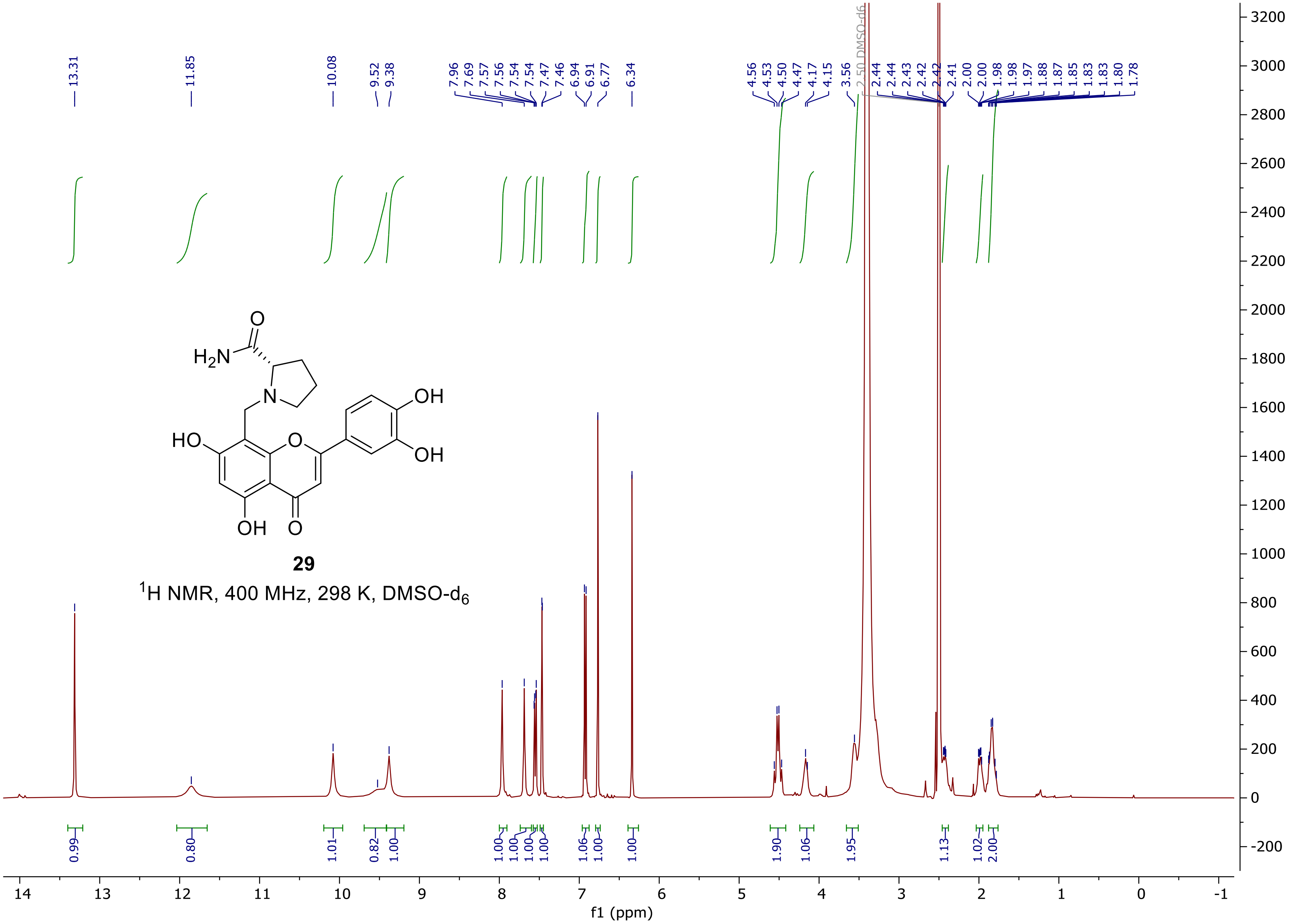

29

^{1}H NMR, 400 MHz, 298 K, DMSO-d_6

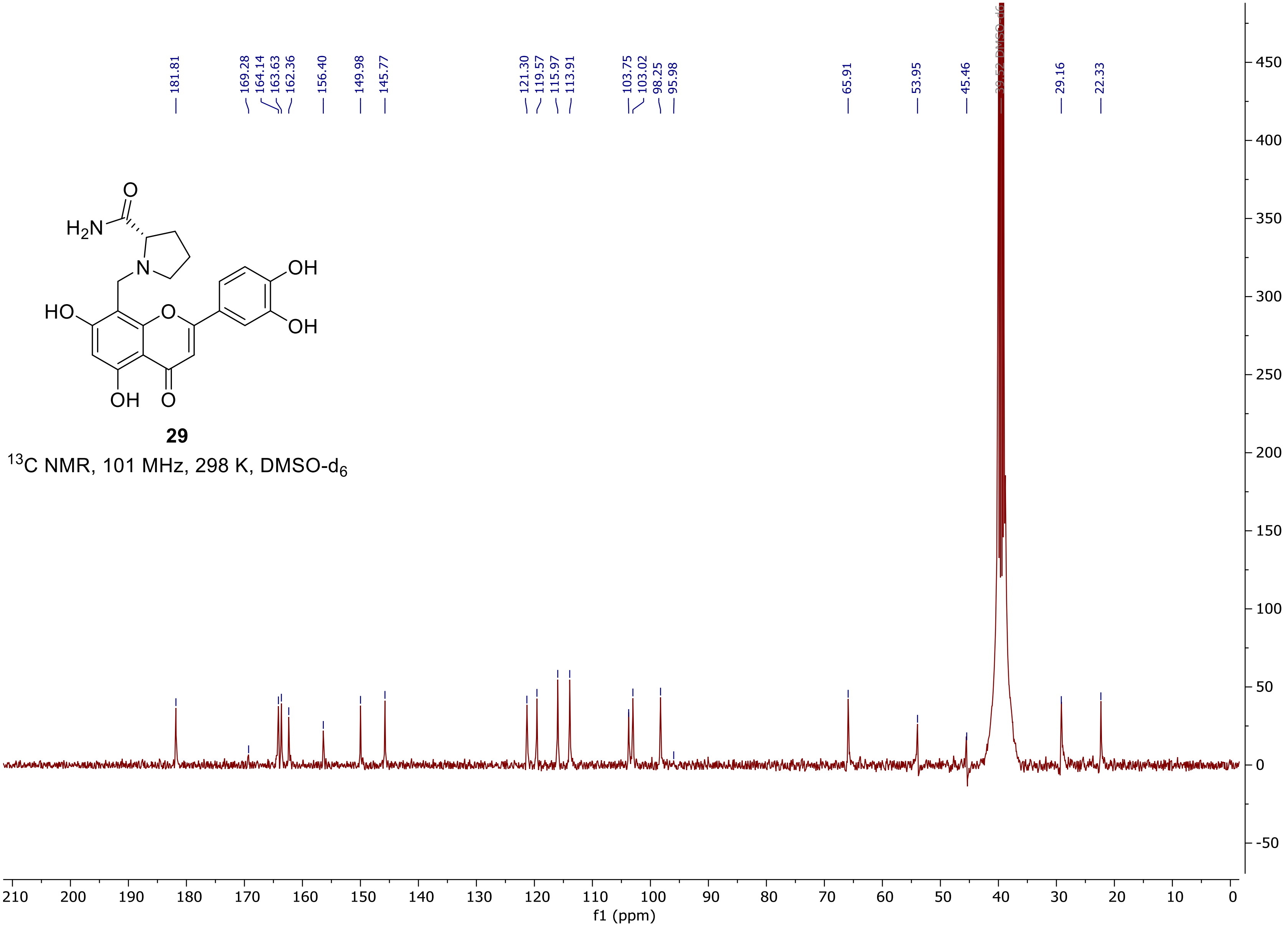

29
13C NMR, 101 MHz, 298 K, DMSO-d6
181.81
169.28
164.14
163.63
162.36
156.40
149.98
145.77
121.30
119.57
115.97
113.91
103.75
103.02
98.25
95.98
65.91
53.95
45.46
29.16
22.33
f1 (ppm)

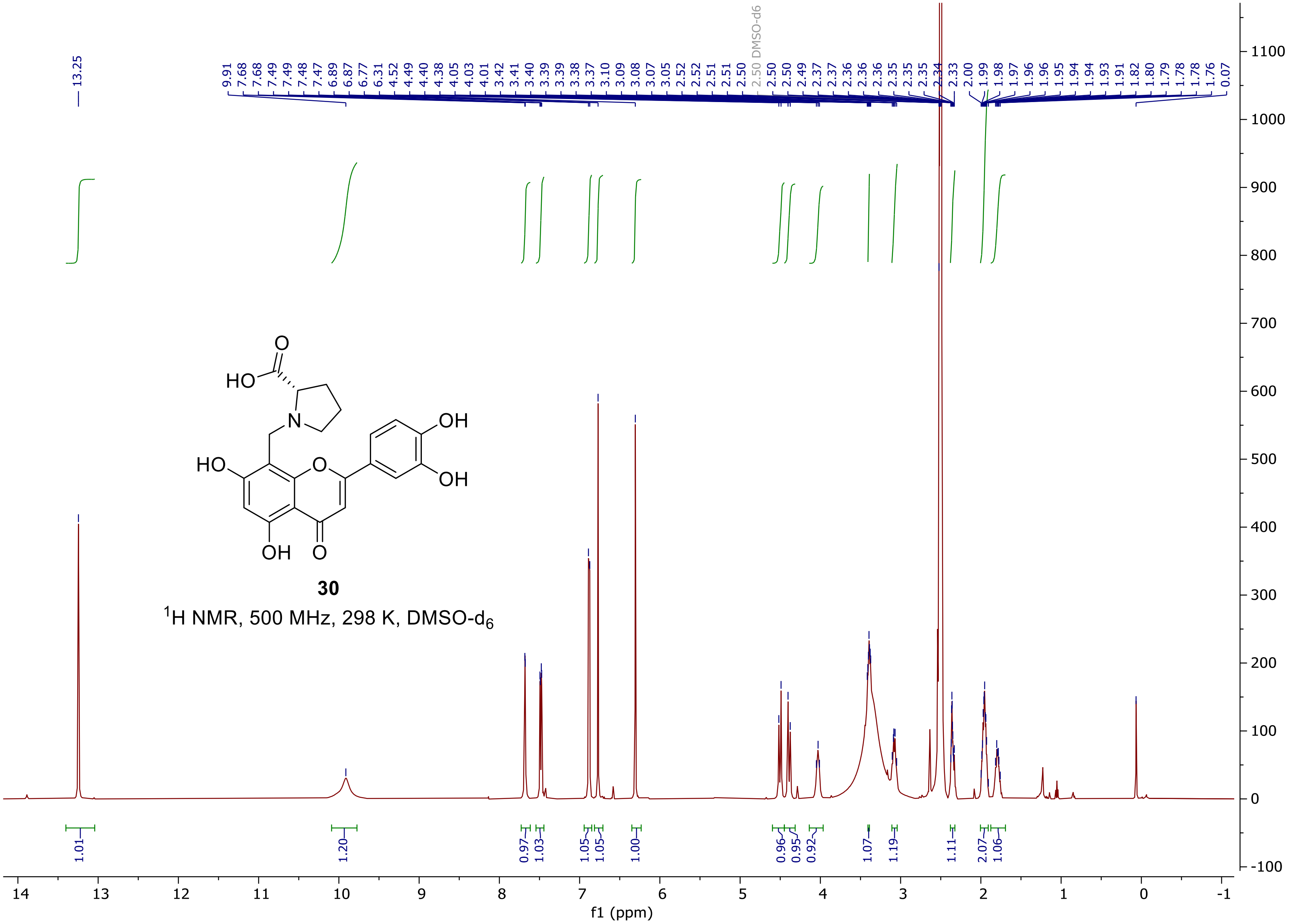
13.25
9.91 7.68 7.68 7.49 7.49 7.48 7.47 6.89 6.87 6.77 6.31 4.52 4.49 4.40 4.38 4.05 4.03 4.01 3.42 3.41 3.40 3.39 3.39 3.38 3.37 3.10 3.09 3.08 3.07 3.05 2.52 2.52 2.51 2.51 2.50 2.50 DMSO-d6 2.50 2.50 2.49 2.37 2.37 2.36 2.36 2.36 2.35 2.35 2.35 2.34 2.33 2.00 1.99 1.98 1.97 1.96 1.96 1.95 1.94 1.93 1.91 1.82 1.80 1.79 1.78 1.78 1.76 0.07
1.01 1.20 0.97 1.03 1.05 1.05 1.00 0.96 0.95 0.92 1.07 1.19 1.11 2.07 1.06
HO
O
N
OH
HO
O
OH
OH
O
30
^{1}H NMR, 500 MHz, 298 K, DMSO-d$_6$
f1 (ppm)

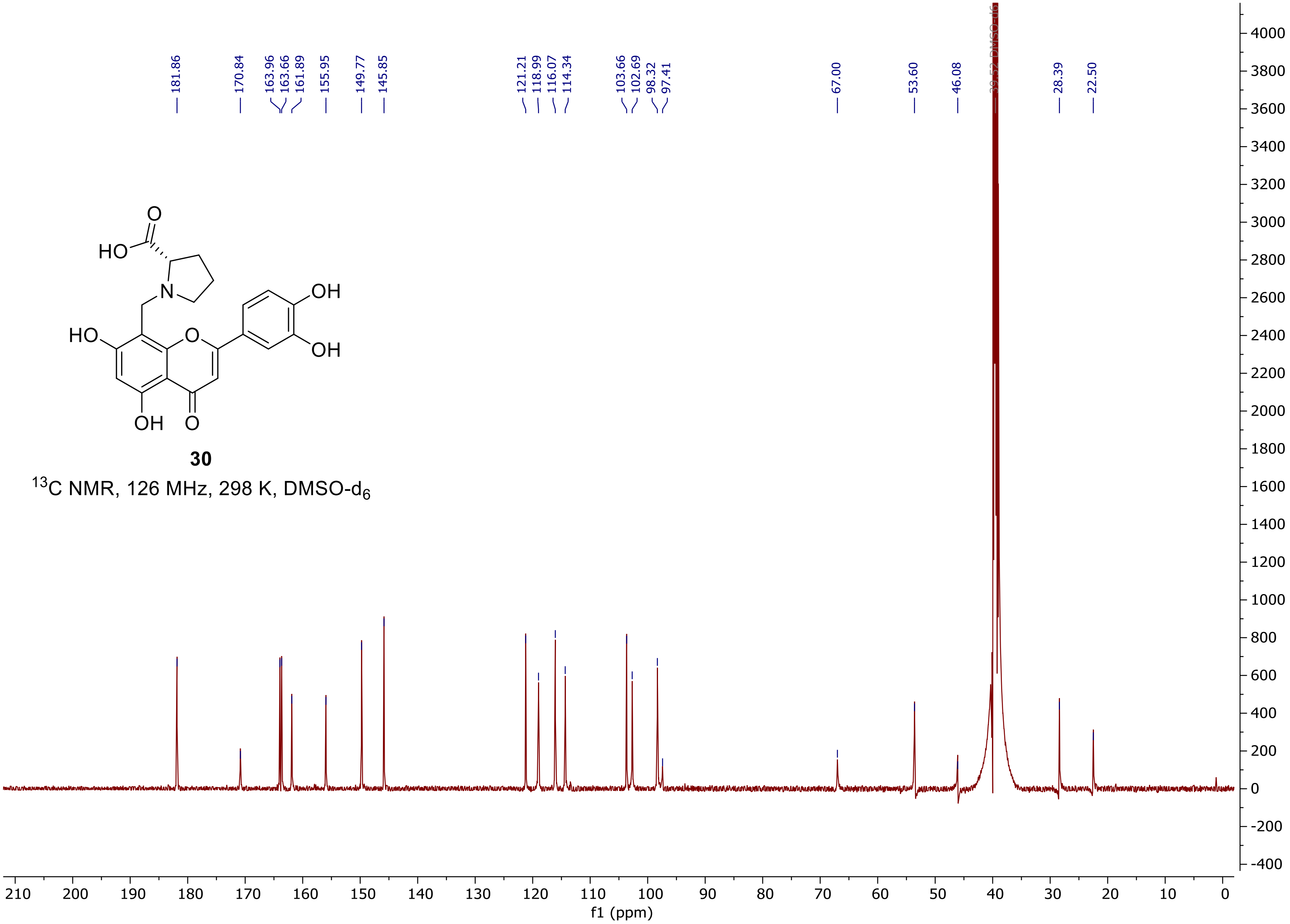

30

^{13}C NMR, 126 MHz, 298 K, DMSO-d_6

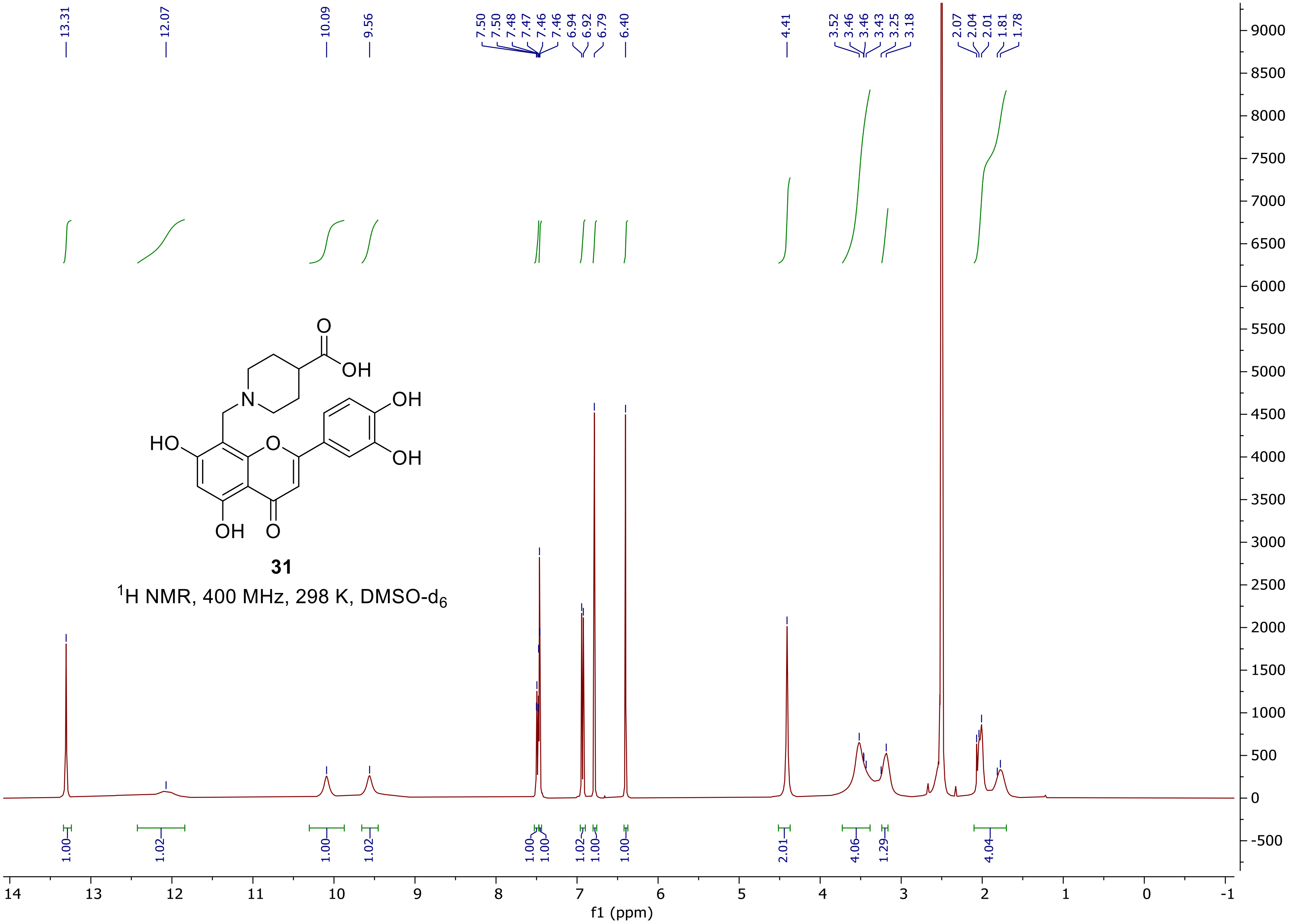

31

^{1}H NMR, 400 MHz, 298 K, DMSO-d$_6$

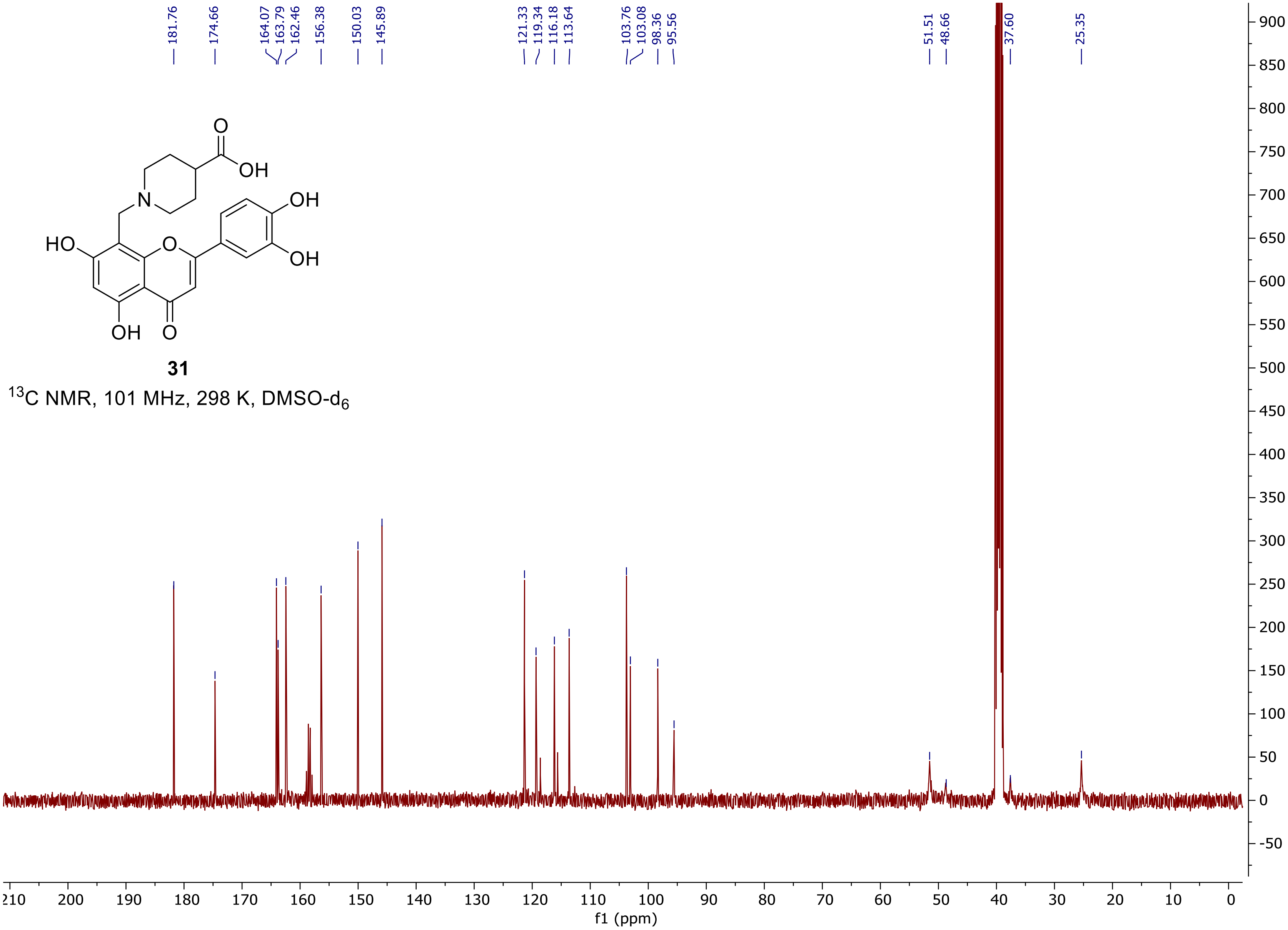
181.76
174.66
164.07
163.79
162.46
156.38
150.03
145.89
121.33
119.34
116.18
113.64
103.76
103.08
98.36
95.56
51.51
48.66
37.60
25.35
OH
N
OH
HO
O
OH
OH
O
31
^{13}C NMR, 101 MHz, 298 K, DMSO-d$_6$
f1 (ppm)

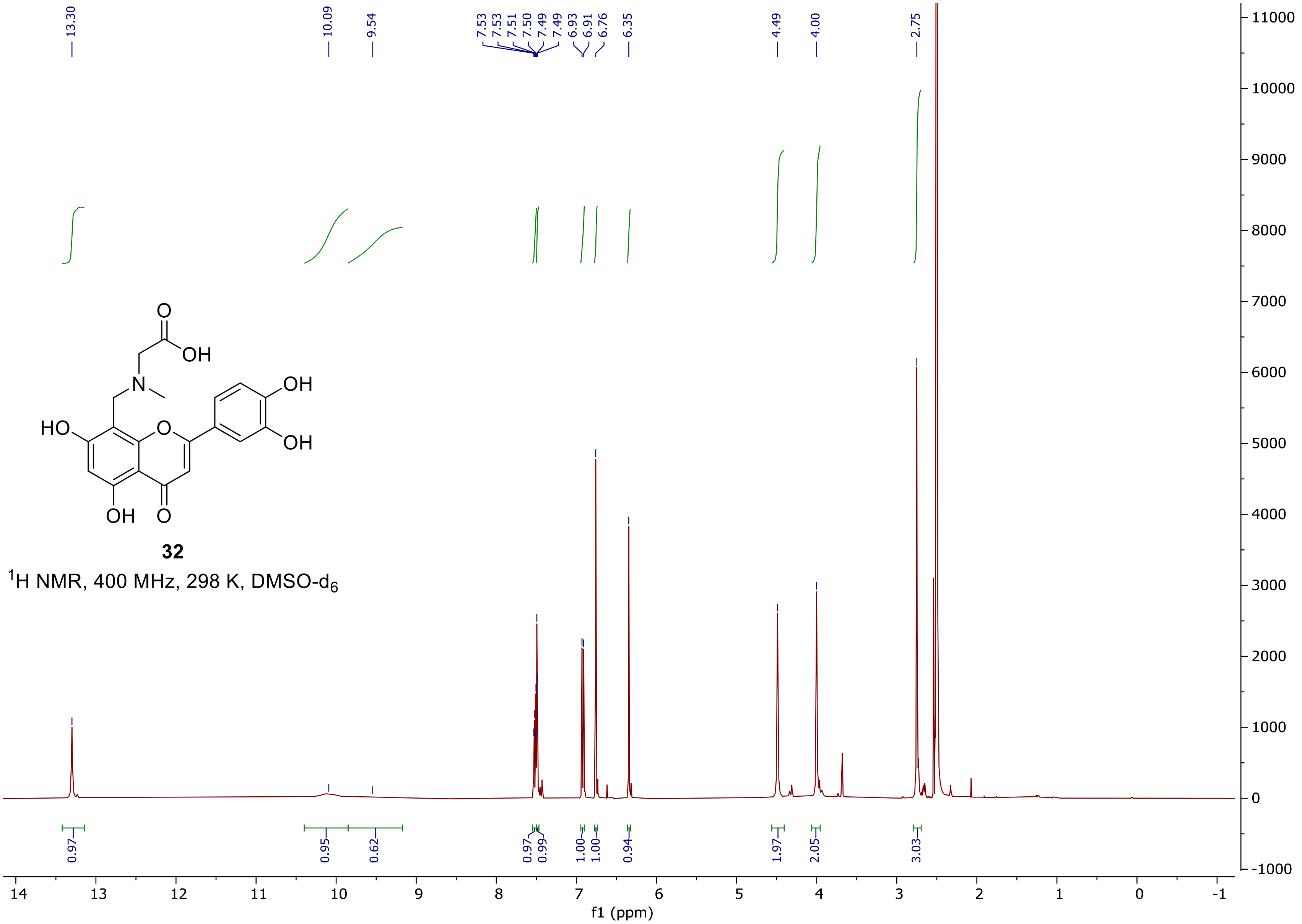

13.30
10.09
9.54
7.53
7.53
7.51
7.50
7.49
7.49
6.93
6.91
6.76
6.35
4.49
4.00
2.75
32
^{1}H NMR, 400 MHz, 298 K, DMSO-d$_6$
0.97
0.95
0.62
0.97
0.99
1.00
1.00
0.94
1.97
2.05
3.03
f1 (ppm)

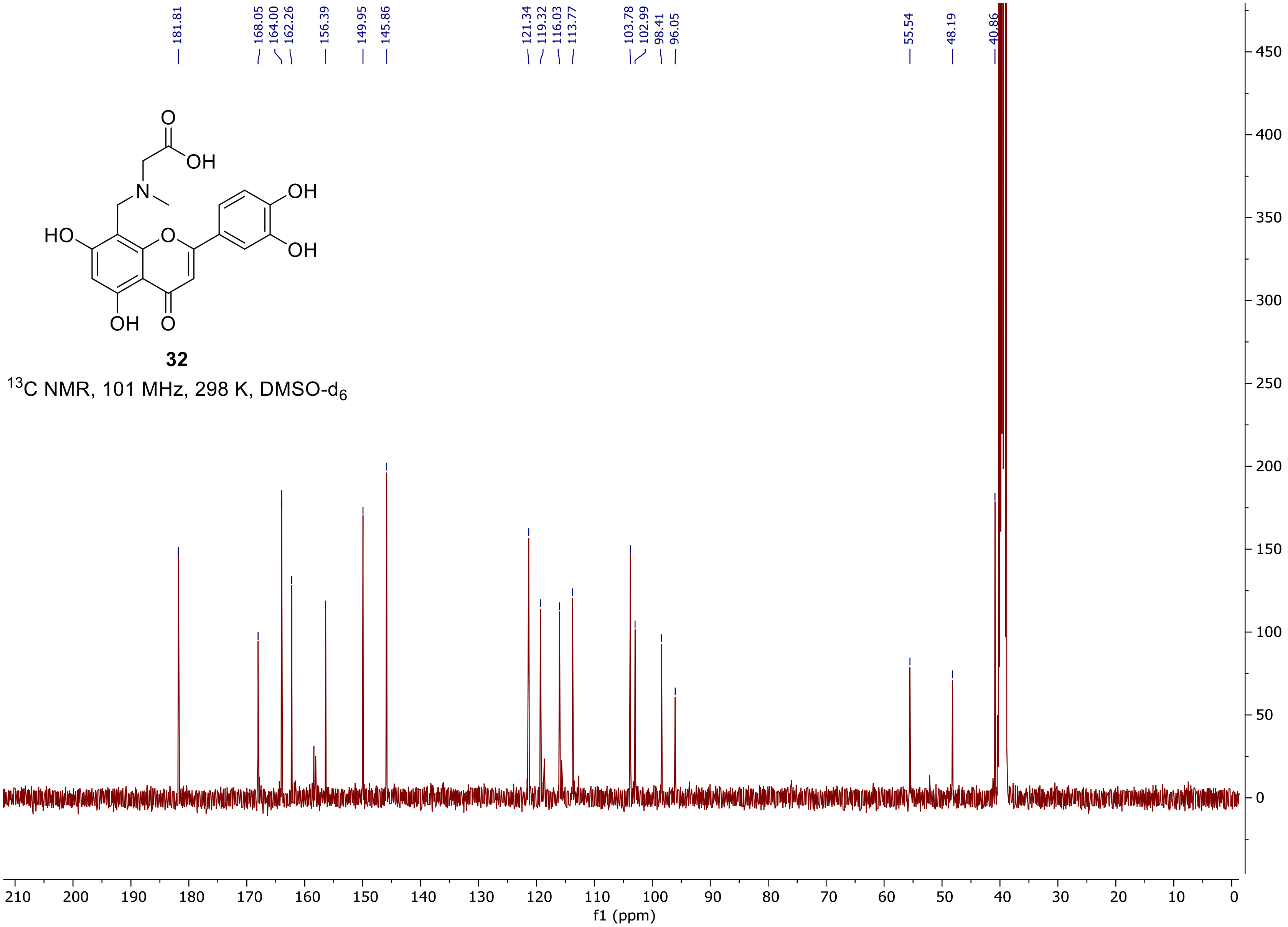

32

^{13}C NMR, 101 MHz, 298 K, DMSO-d$_6$

HPLC analysis of final compounds

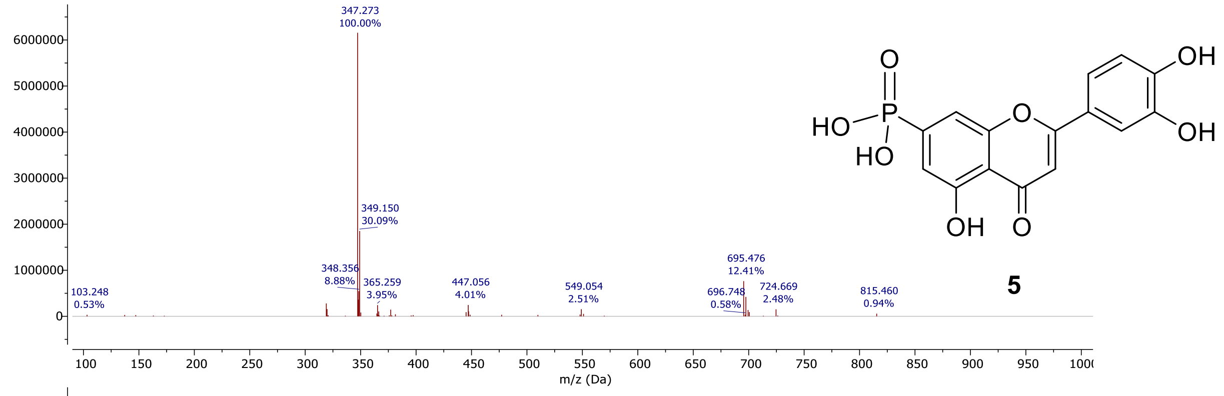

5

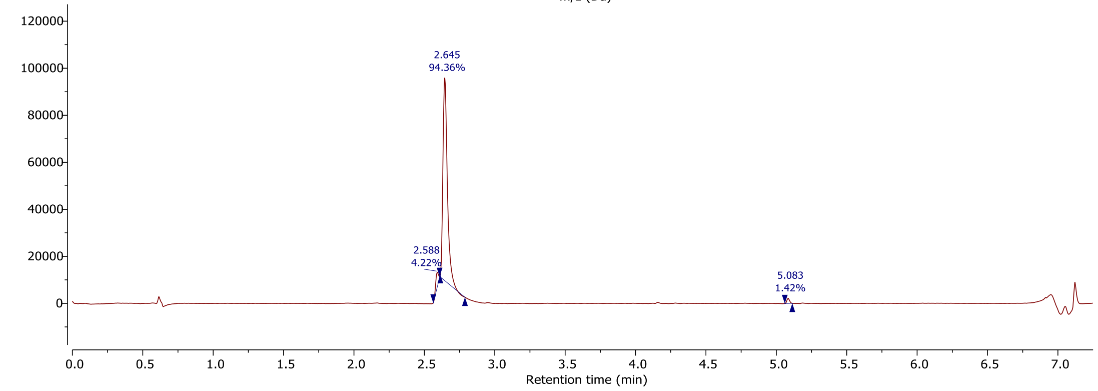

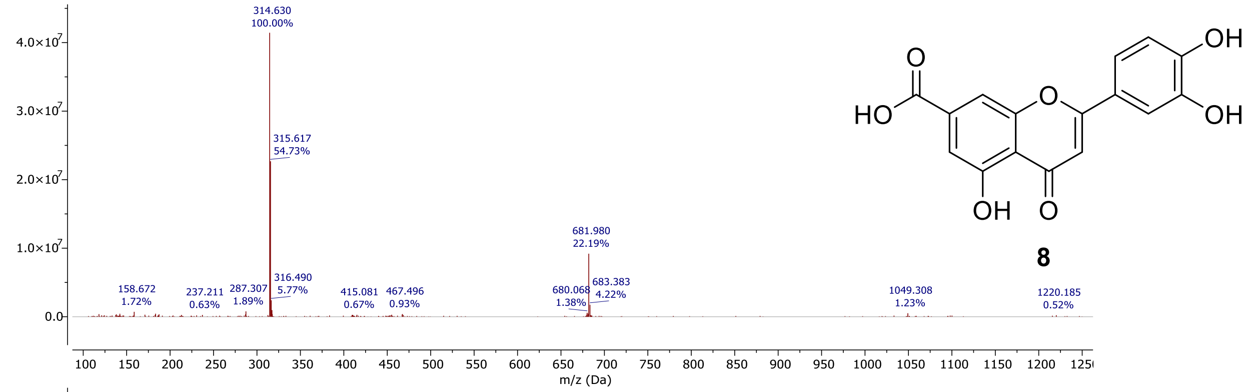

8

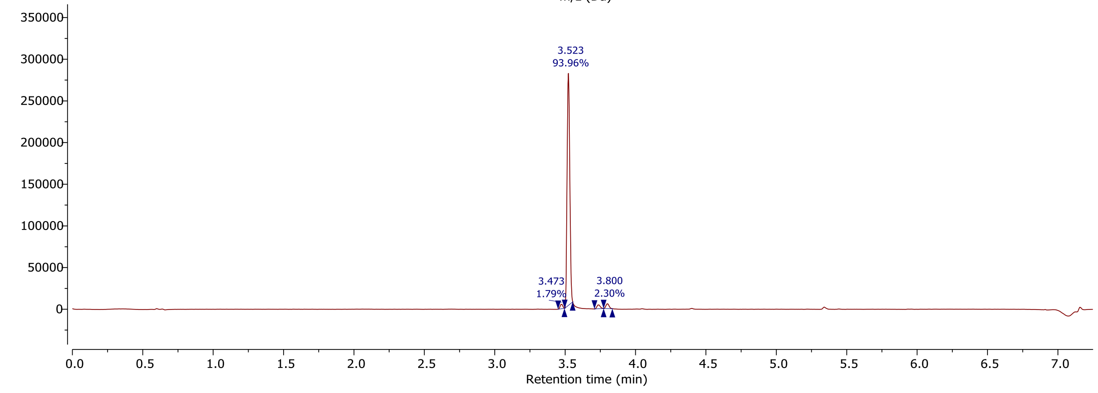

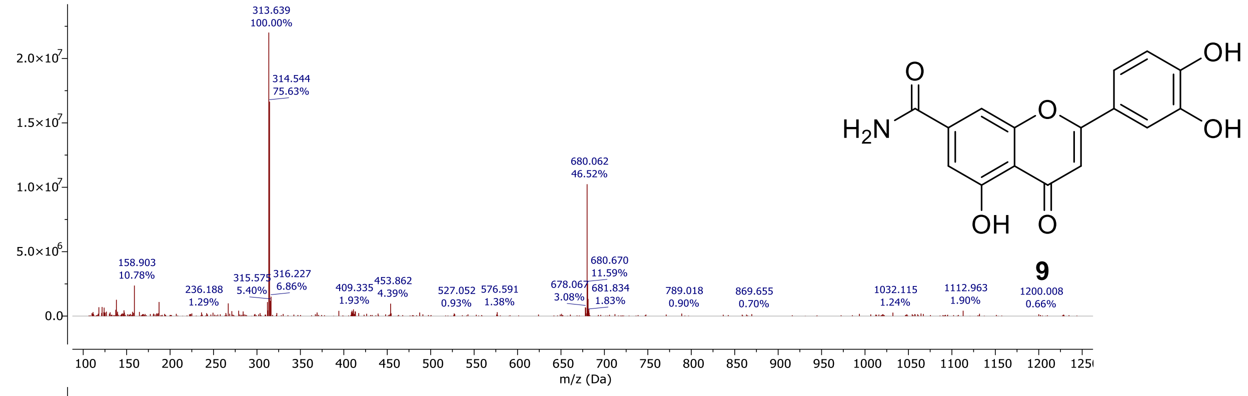
313.639
100.00%
314.544
75.63%
680.062
46.52%
158.903
10.78%
236.188
1.29%
315.575
5.40%
316.227
6.86%
409.335
1.93%
453.862
4.39%
527.052
0.93%
576.591
1.38%
678.067
3.08%
680.670
11.59%
681.834
1.83%
789.018
0.90%
869.655
0.70%
1032.115
1.24%
1112.963
1.90%
1200.008
0.66%
m/z (Da)
OH
OH
O
O
H_2N
OH
O

9

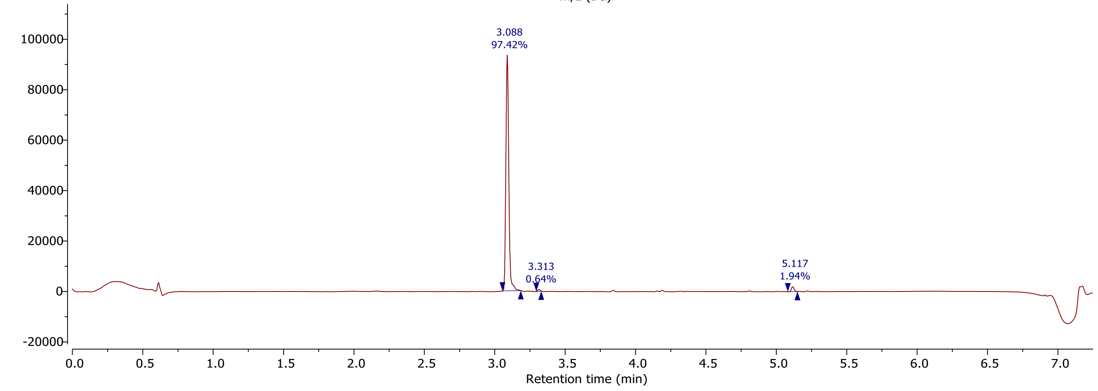
3.088
97.42%
3.313
0.64%
5.117
1.94%
Retention time (min)

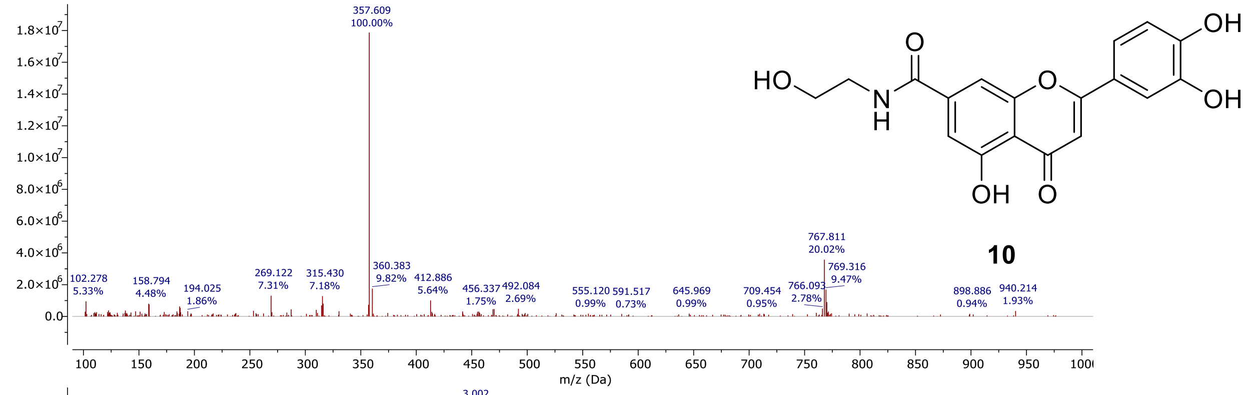
357.609
100.00%
102.278
5.33%
158.794
4.48%
194.025
1.86%
269.122
7.31%
315.430
7.18%
360.383
9.82%
412.886
5.64%
456.337
1.75%
492.084
2.69%
555.120
0.99%
591.517
0.73%
645.969
0.99%
709.454
0.95%
766.093
2.78%
767.811
20.02%
769.316
9.47%
898.886
0.94%
940.214
1.93%
m/z (Da)
OH
OH
O
O
HO
N
H
OH
O

10

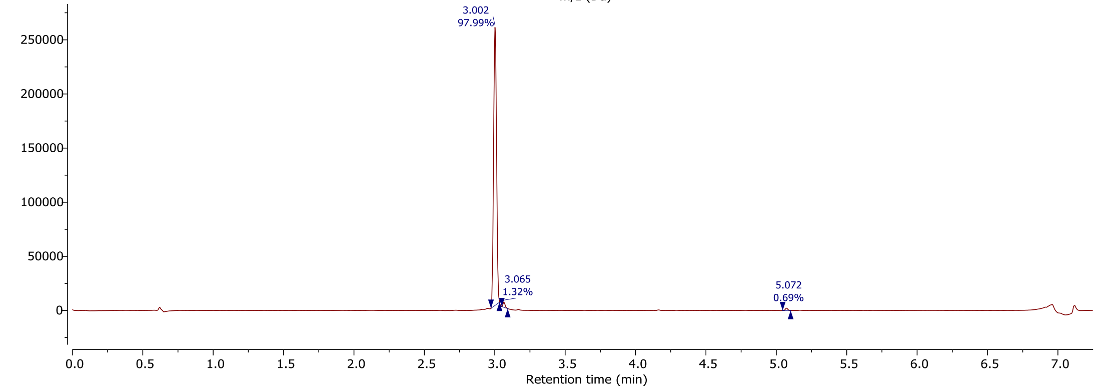
3.002
97.99%
3.065
1.32%
5.072
0.69%
Retention time (min)

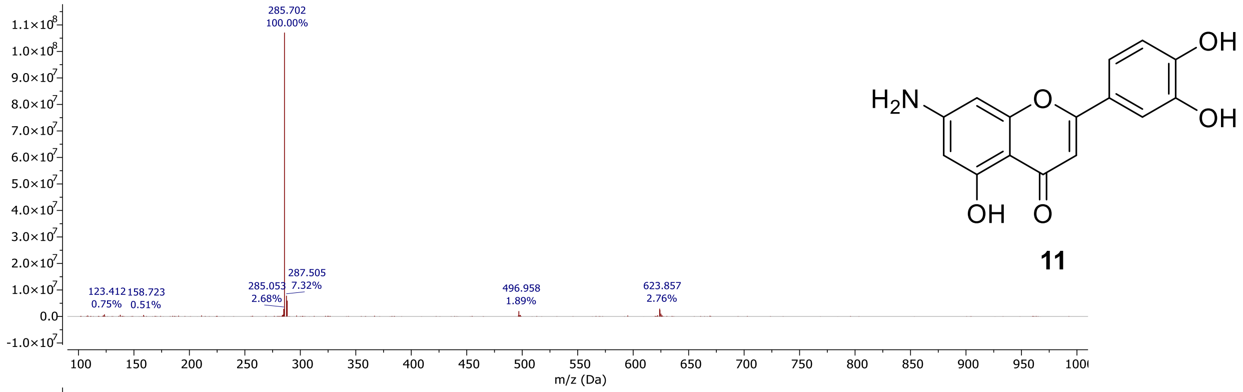
285.702
100.00%
123.412
0.75%
158.723
0.51%
285.053
2.68%
287.505
7.32%
496.958
1.89%
623.857
2.76%
m/z (Da)
OH
OH
H_2N
O
OH
O

11

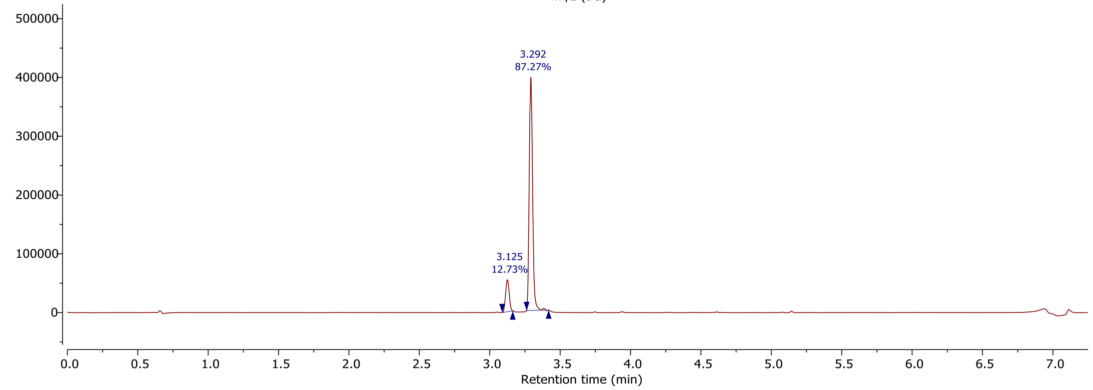
3.292
87.27%
3.125
12.73%
Retention time (min)

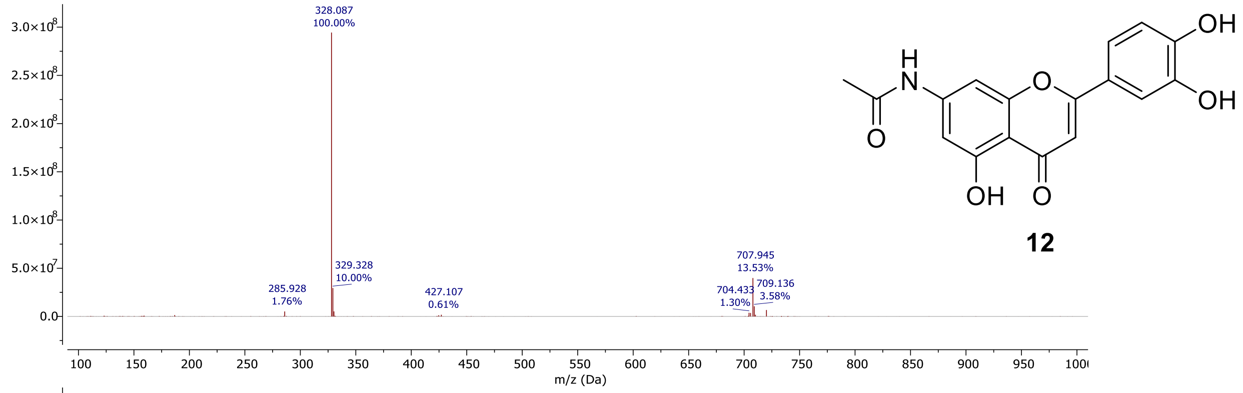
328.087
100.00%
329.328
10.00%
285.928
1.76%
427.107
0.61%
707.945
13.53%
704.433
1.30%
709.136
3.58%
m/z (Da)
OH
H
N
O
OH
O
OH
O

12

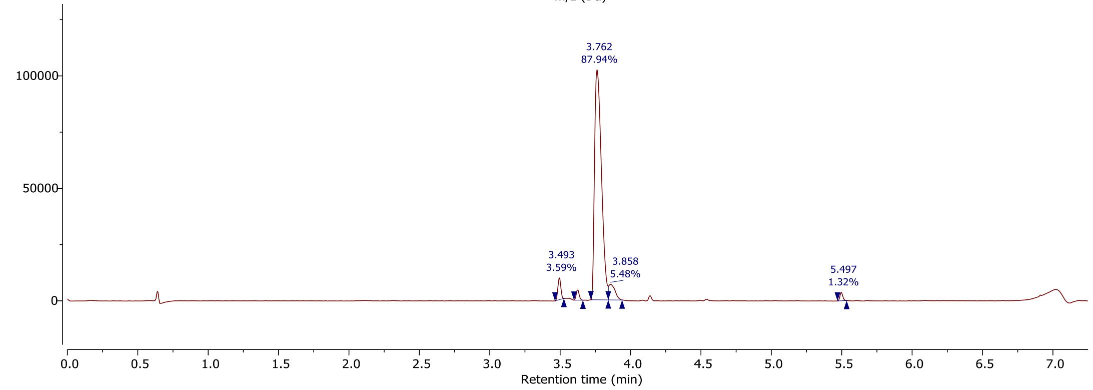
3.762
87.94%
3.493
3.59%
3.858
5.48%
5.497
1.32%
Retention time (min)

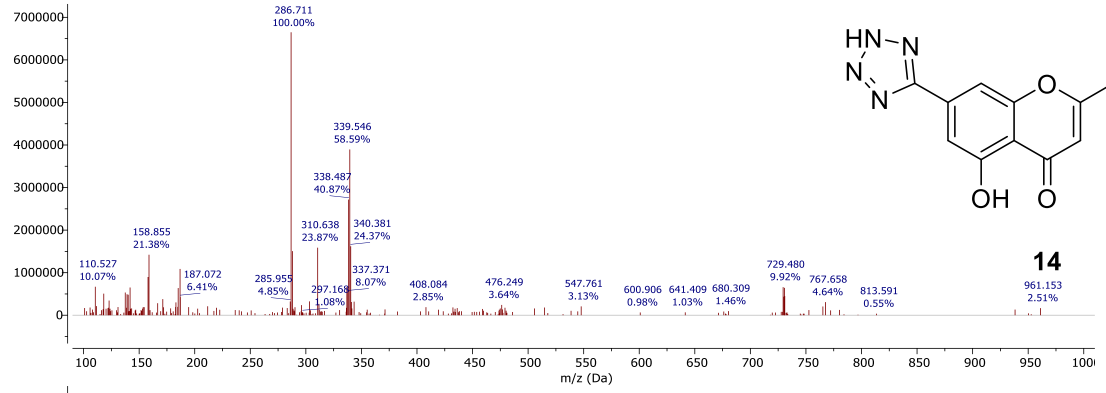
286.711
100.00%
339.546
58.59%
338.487
40.87%
310.638
23.87%
340.381
24.37%
158.855
21.38%
187.072
6.41%
110.527
10.07%
285.955
4.85%
297.168
1.08%
337.371
8.07%
408.084
2.85%
476.249
3.64%
547.761
3.13%
600.906
0.98%
641.409
1.03%
680.309
1.46%
729.480
9.92%
767.658
4.64%
813.591
0.55%
961.153
2.51%
m/z (Da)
HN
N
N
N
OH
OH
O
OH
O
14

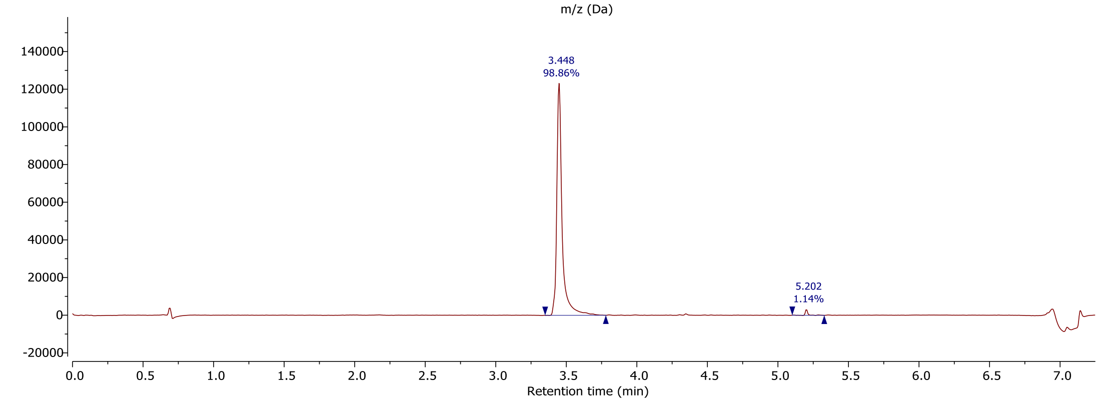
3.448
98.86%
5.202
1.14%
Retention time (min)

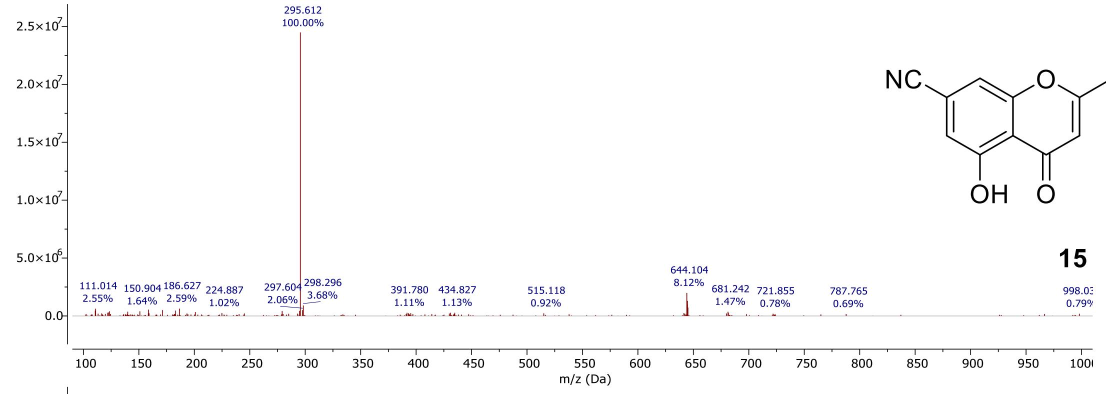
295.612
100.00%
111.014
2.55%
150.904
1.64%
186.627
2.59%
224.887
1.02%
297.604
2.06%
298.296
3.68%
391.780
1.11%
434.827
1.13%
515.118
0.92%
644.104
8.12%
681.242
1.47%
721.855
0.78%
787.765
0.69%
998.03
0.79%
m/z (Da)
NC
O
OH
OH
OH
O
15

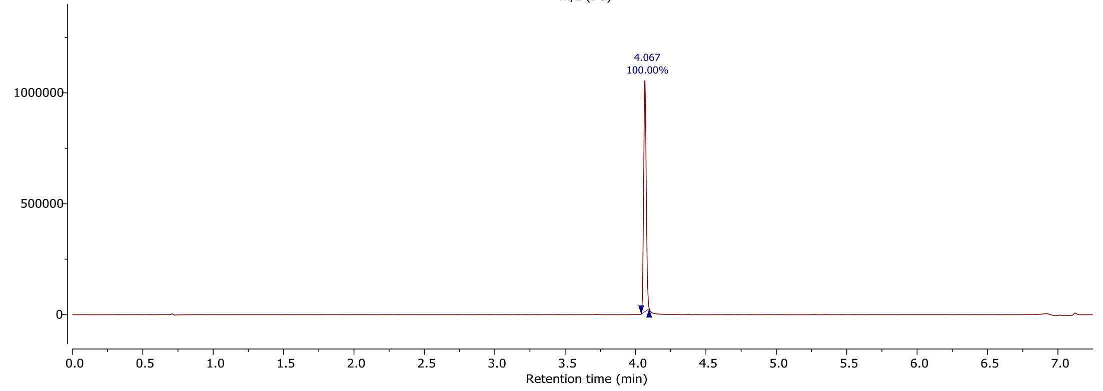
4.067
100.00%
Retention time (min)

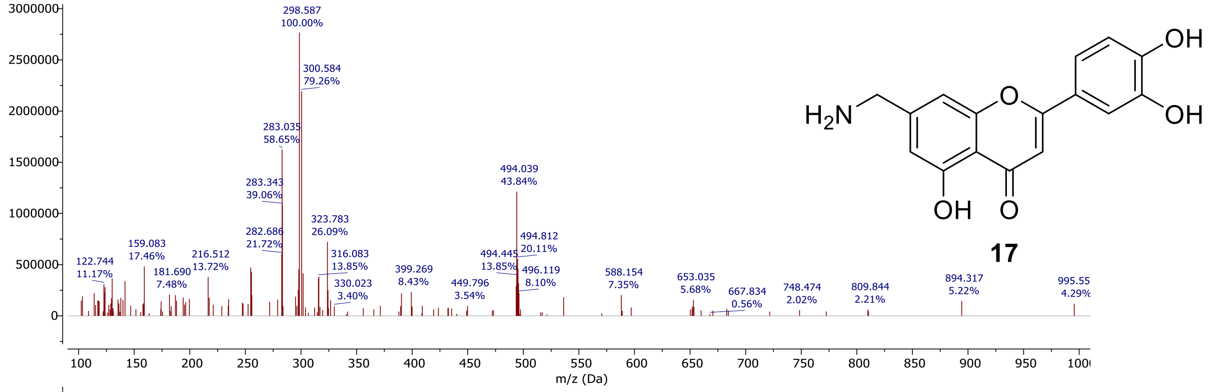
298.587
100.00%
300.584
79.26%
283.035
58.65%
283.343
39.06%
282.686
21.72%
323.783
26.09%
316.083
13.85%
330.023
3.40%
494.039
43.84%
494.812
20.11%
494.445
13.85%
496.119
8.10%
122.744
11.17%
159.083
17.46%
181.690
7.48%
216.512
13.72%
399.269
8.43%
449.796
3.54%
588.154
7.35%
653.035
5.68%
667.834
0.56%
748.474
2.02%
809.844
2.21%
894.317
5.22%
m/z (Da)

17

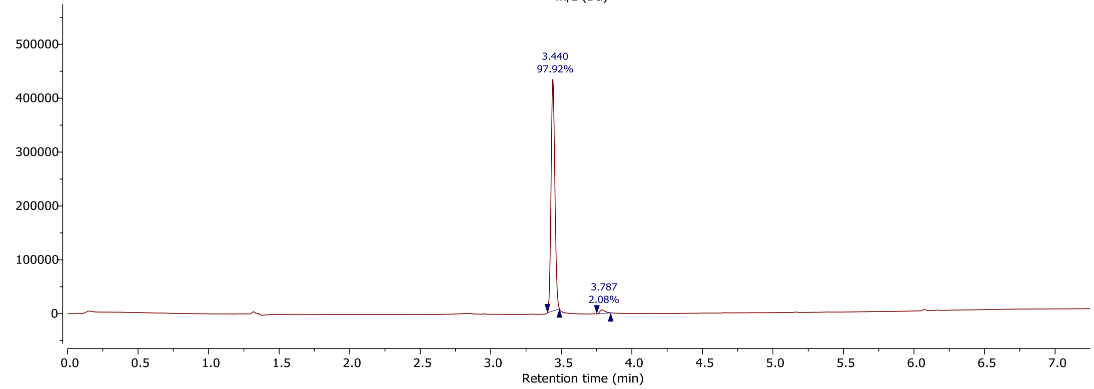
3.440
97.92%
3.787
2.08%
Retention time (min)

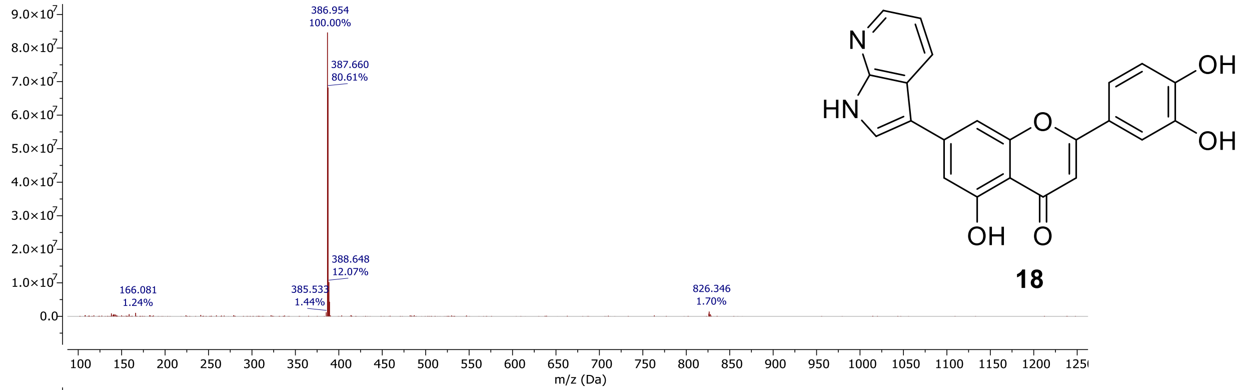
386.954
100.00%
387.660
80.61%
388.648
12.07%
166.081
1.24%
385.533
1.44%
826.346
1.70%
m/z (Da)

18

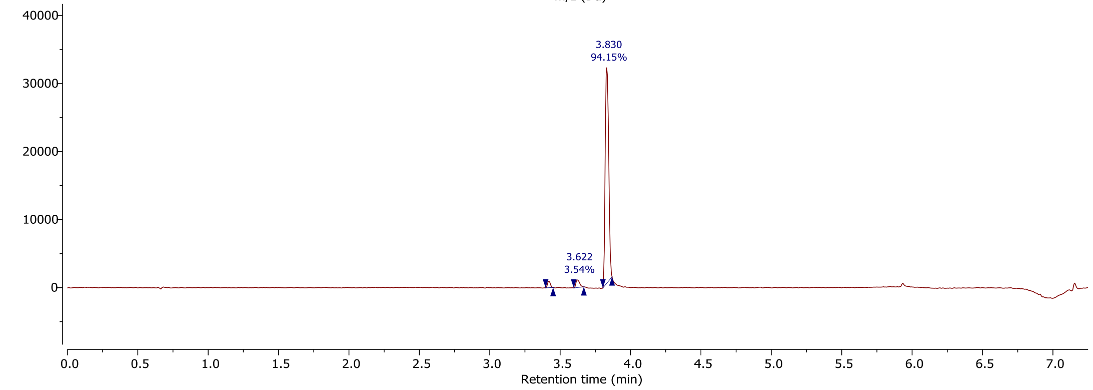
3.830
94.15%
3.622
3.54%
Retention time (min)

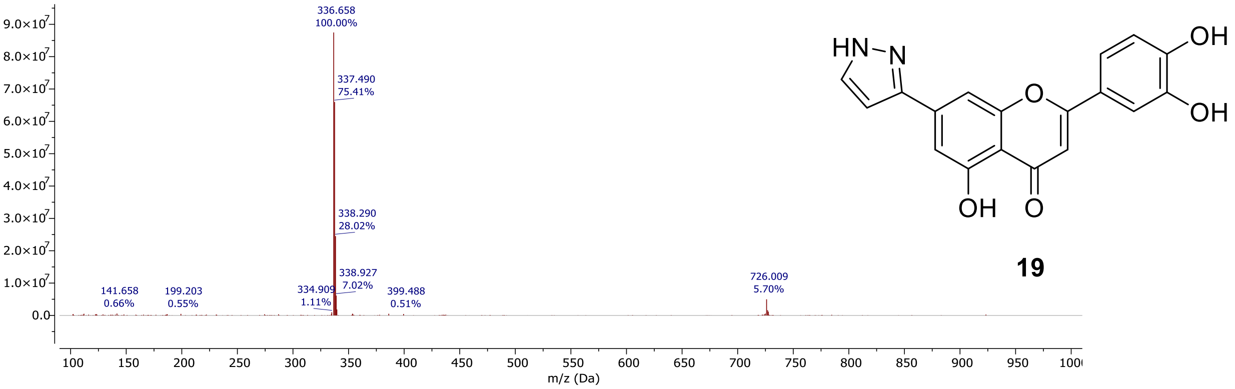
336.658
100.00%
337.490
75.41%
338.290
28.02%
338.927
7.02%
334.909
1.11%
141.658
0.66%
199.203
0.55%
399.488
0.51%
726.009
5.70%
m/z (Da)
HN–N
OH
OH
O
OH
O

19

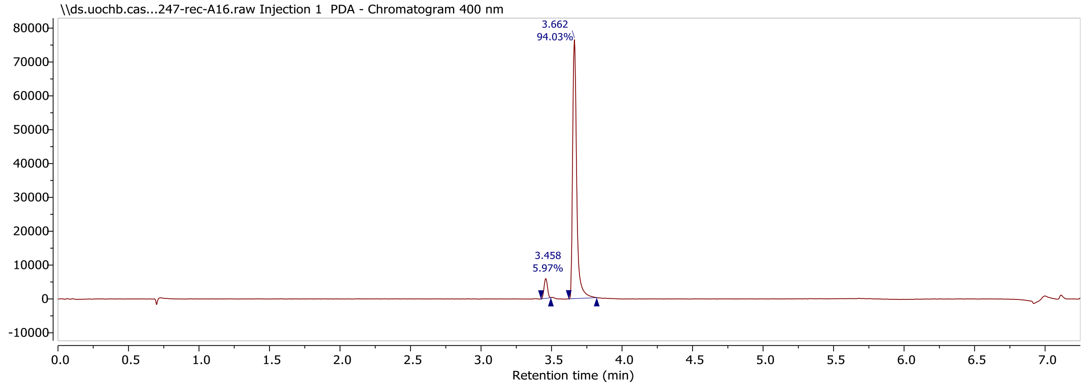
\\ds.uochb.cas...247-rec-A16.raw Injection 1 PDA - Chromatogram 400 nm
3.662
94.03%
3.458
5.97%
Retention time (min)

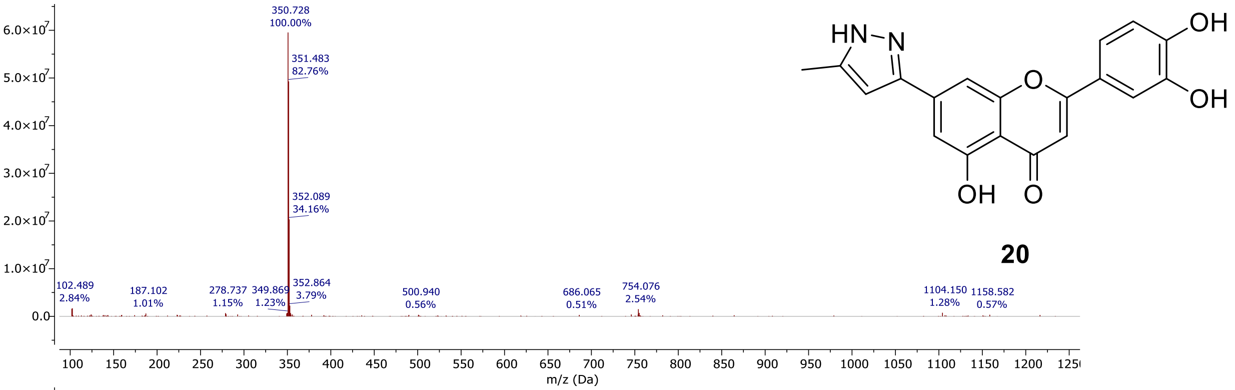
350.728
100.00%
351.483
82.76%
352.089
34.16%
352.864
3.79%
349.869
1.23%
102.489
2.84%
187.102
1.01%
278.737
1.15%
500.940
0.56%
686.065
0.51%
754.076
2.54%
1104.150
1.28%
1158.582
0.57%
m/z (Da)
HN–N
OH
OH
O
OH
O

20

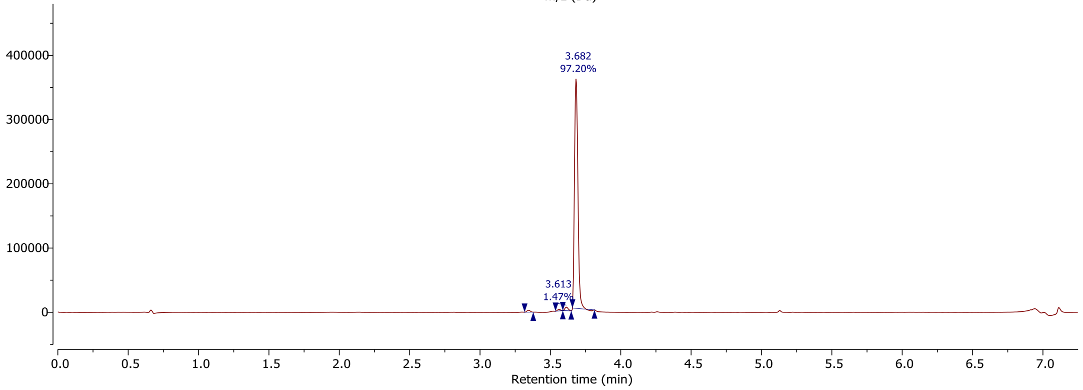
3.682
97.20%
3.613
1.47%
Retention time (min)

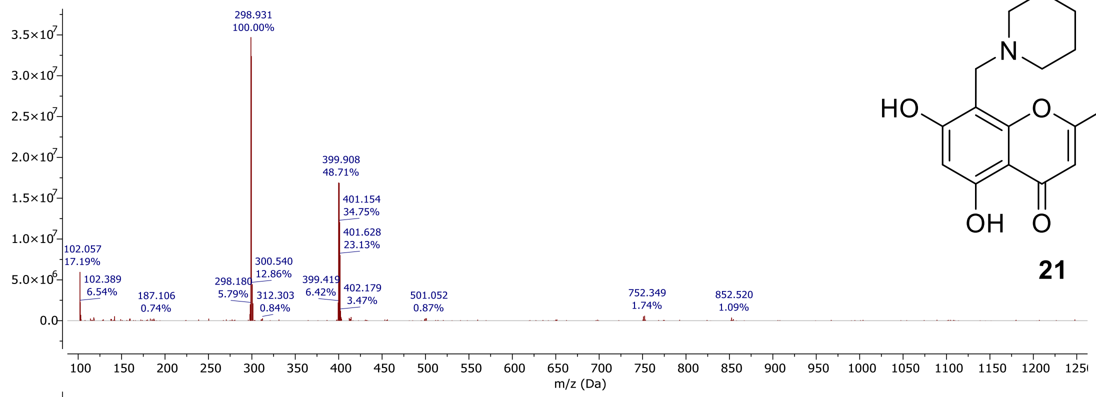
298.931
100.00%
399.908
48.71%
401.154
34.75%
401.628
23.13%
102.057
17.19%
102.389
6.54%
187.106
0.74%
298.180
5.79%
300.540
12.86%
312.303
0.84%
399.419
6.42%
402.179
3.47%
501.052
0.87%
752.349
1.74%
852.520
1.09%
m/z (Da)

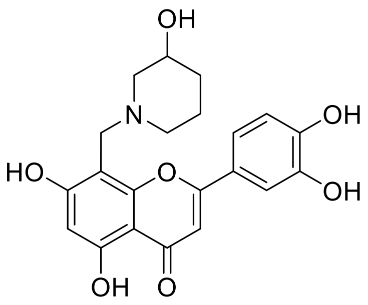
OH
N
OH
HO
O
OH
OH
O

21

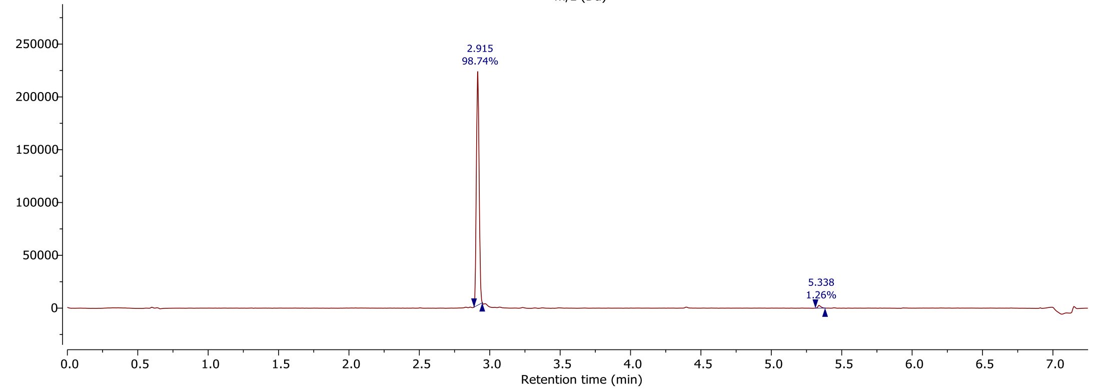
2.915
98.74%
5.338
1.26%
Retention time (min)

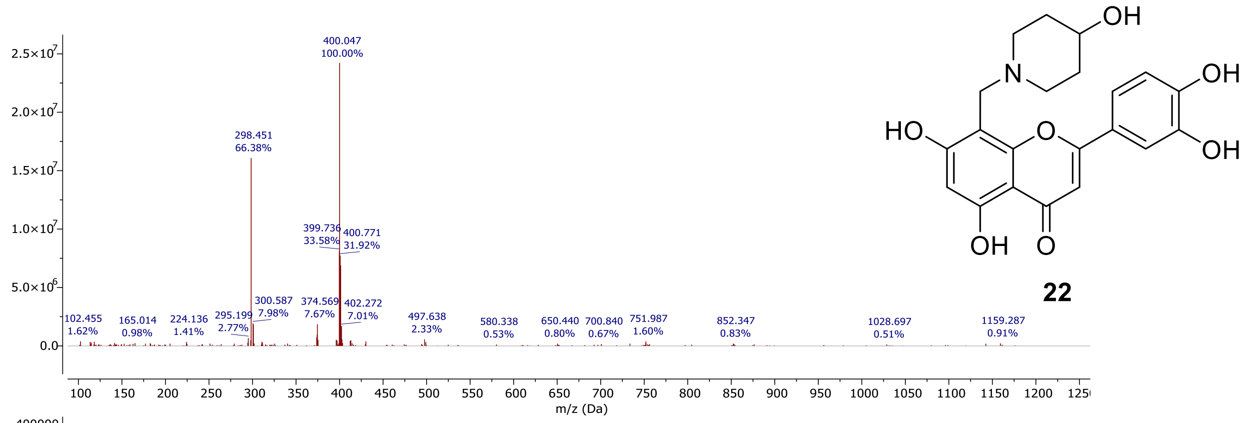
400.047
100.00%
298.451
66.38%
399.736
33.58%
400.771
31.92%
102.455
1.62%
165.014
0.98%
224.136
1.41%
295.199
2.77%
300.587
7.98%
374.569
7.67%
402.272
7.01%
497.638
2.33%
580.338
0.53%
650.440
0.80%
700.840
0.67%
751.987
1.60%
852.347
0.83%
1028.697
0.51%
1159.287
0.91%
m/z (Da)
OH
N
OH
HO
O
OH
OH
O

22

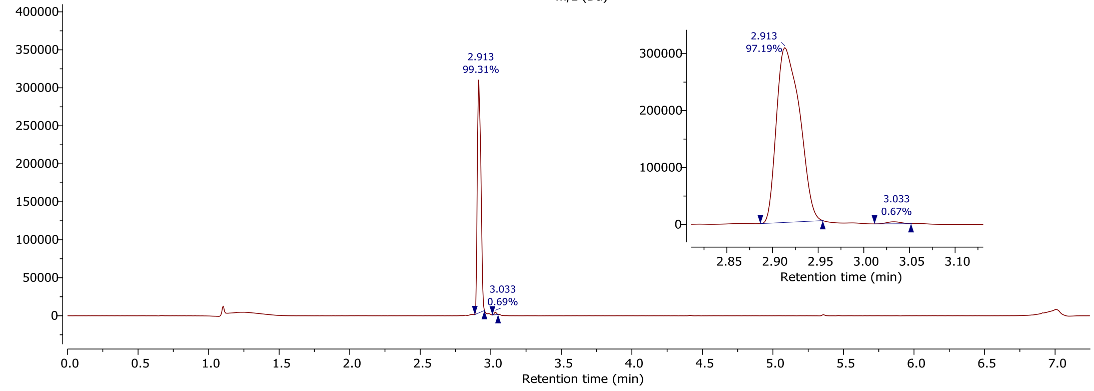
2.913
99.31%
3.033
0.69%
2.913
97.19%
3.033
0.67%
Retention time (min)
Retention time (min)

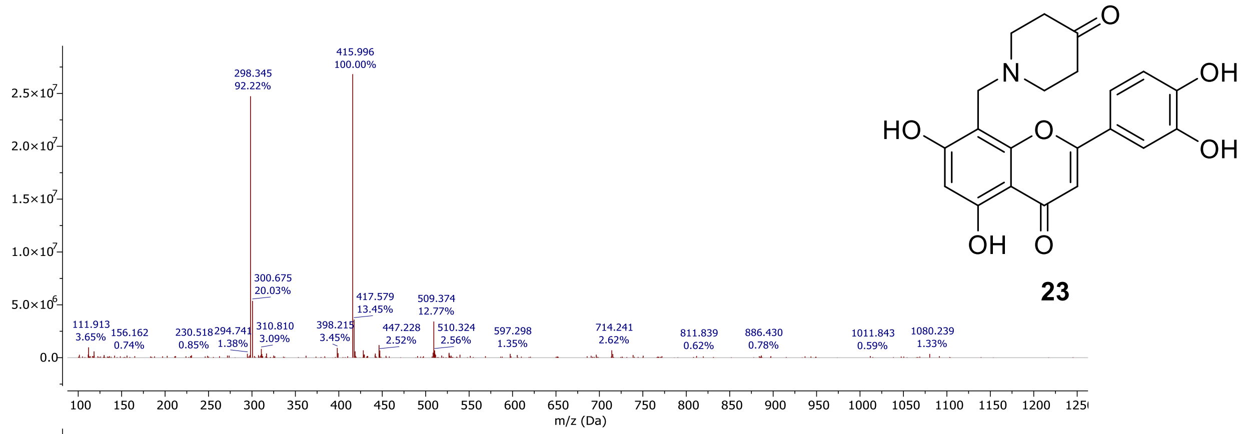
O
N
OH
HO
O
OH
OH
O
298.345
92.22%
415.996
100.00%
300.675
20.03%
417.579
13.45%
509.374
12.77%
111.913
3.65%
156.162
0.74%
230.518
0.85%
294.741
1.38%
310.810
3.09%
398.215
3.45%
447.228
2.52%
510.324
2.56%
597.298
1.35%
714.241
2.62%
811.839
0.62%
886.430
0.78%
1011.843
0.59%
1080.239
1.33%
m/z (Da)

23

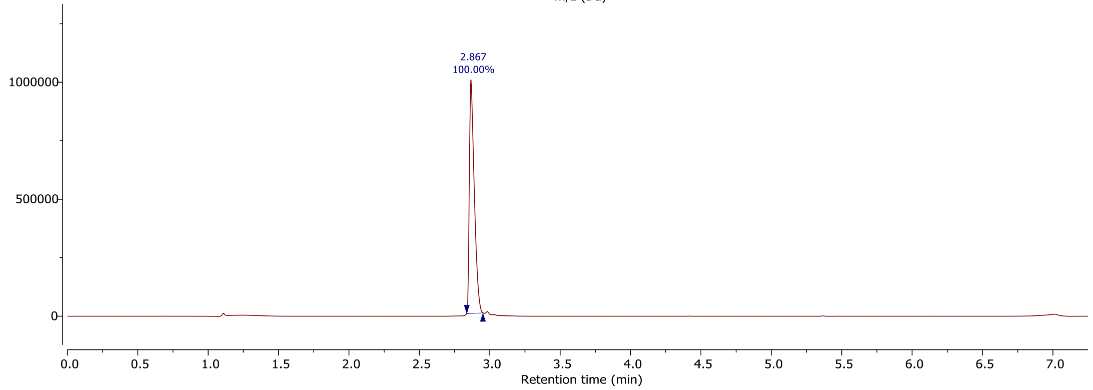
2.867
100.00%
Retention time (min)

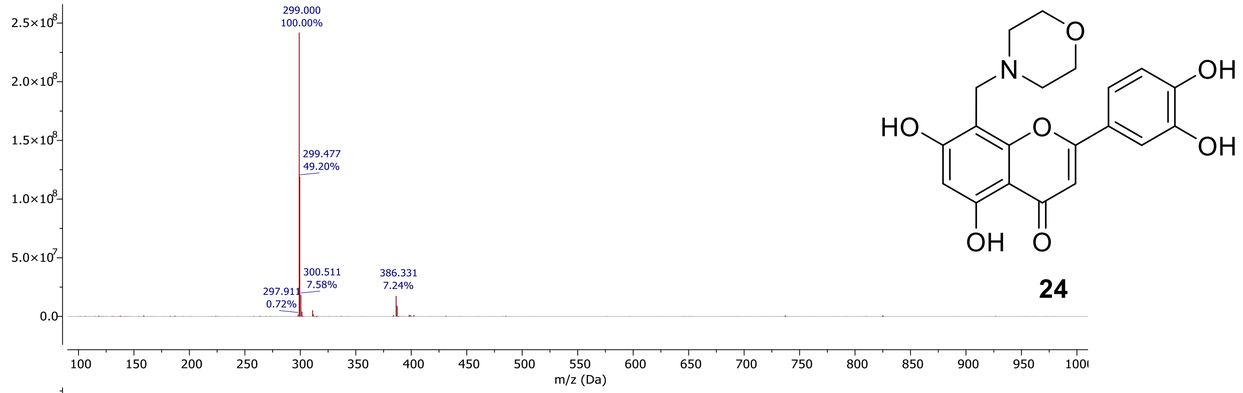
O
N
OH
HO
O
OH
OH
O
299.000
100.00%
299.477
49.20%
300.511
7.58%
297.911
0.72%
386.331
7.24%
m/z (Da)

24

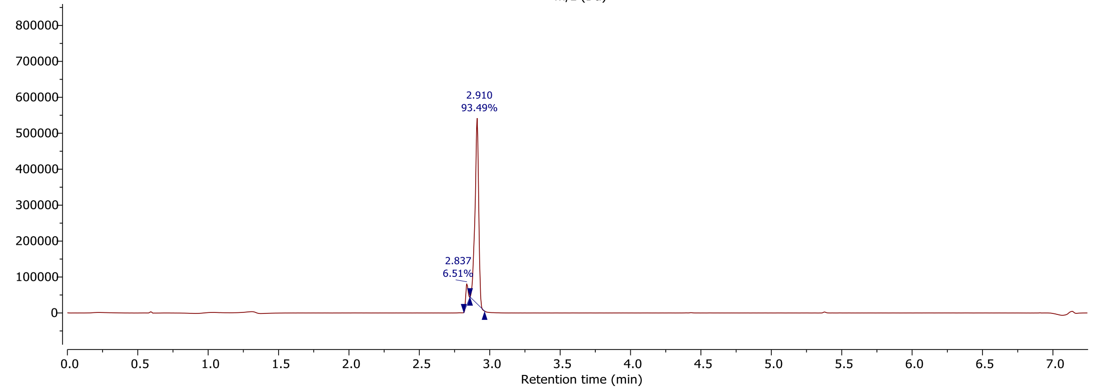
2.910
93.49%
2.837
6.51%
Retention time (min)

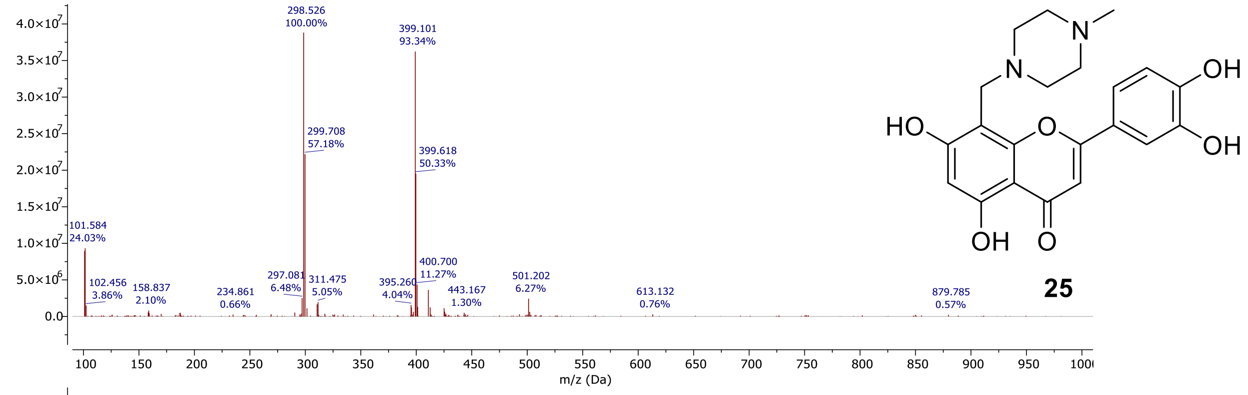
298.526
100.00%
399.101
93.34%
299.708
57.18%
399.618
50.33%
101.584
24.03%
102.456
3.86%
158.837
2.10%
234.861
0.66%
297.081
6.48%
311.475
5.05%
395.260
4.04%
400.700
11.27%
443.167
1.30%
501.202
6.27%
613.132
0.76%
879.785
0.57%
m/z (Da)
N
N
HO
O
OH
OH
OH
O

25

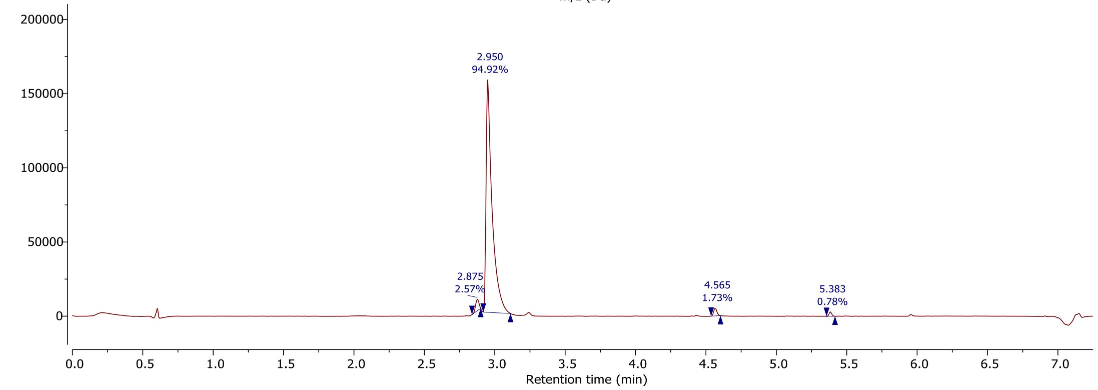
2.950
94.92%
2.875
2.57%
4.565
1.73%
5.383
0.78%
Retention time (min)

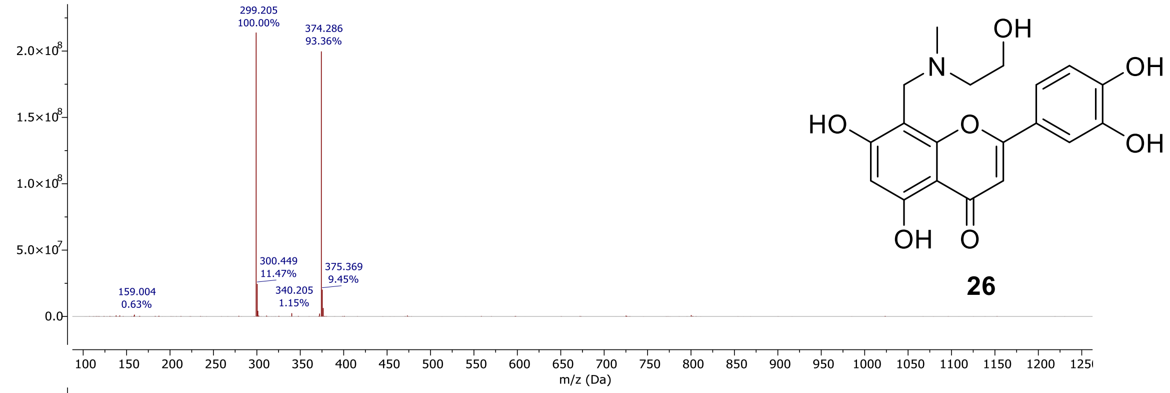
299.205
100.00%
374.286
93.36%
159.004
0.63%
300.449
11.47%
340.205
1.15%
375.369
9.45%
m/z (Da)
N
OH
HO
O
OH
OH
OH
O

26

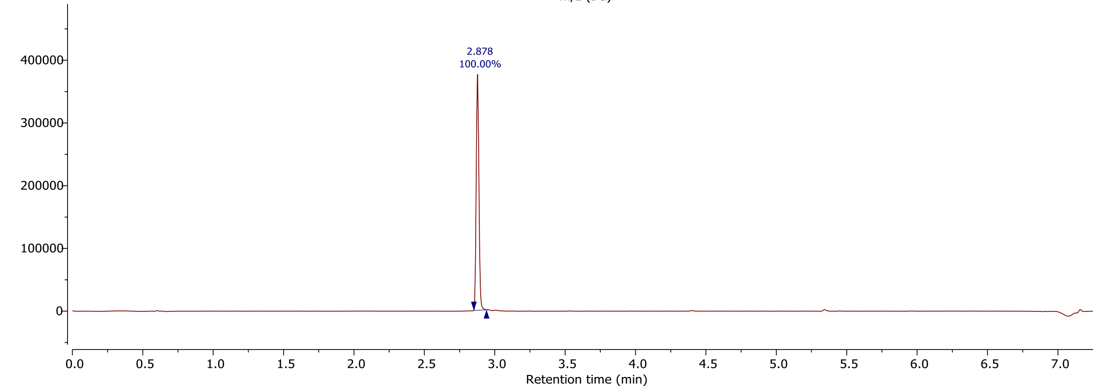
2.878
100.00%
Retention time (min)

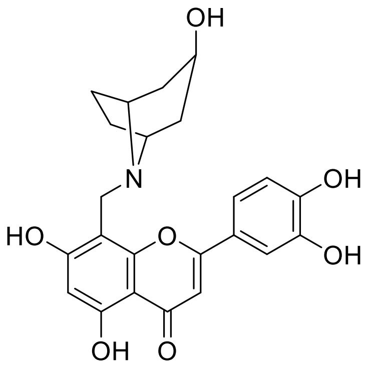
OH
N
HO
O
OH
OH
OH
O

27

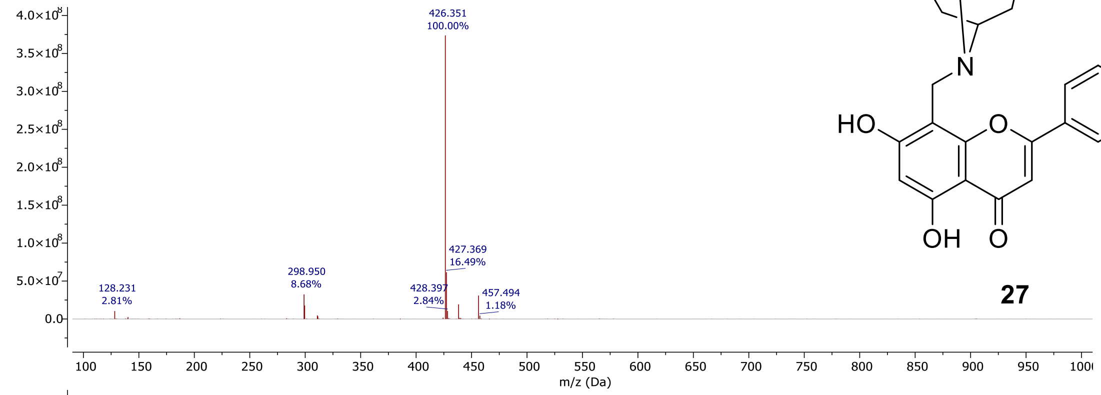
426.351
100.00%
427.369
16.49%
298.950
8.68%
128.231
2.81%
428.397
2.84%
457.494
1.18%
m/z (Da)

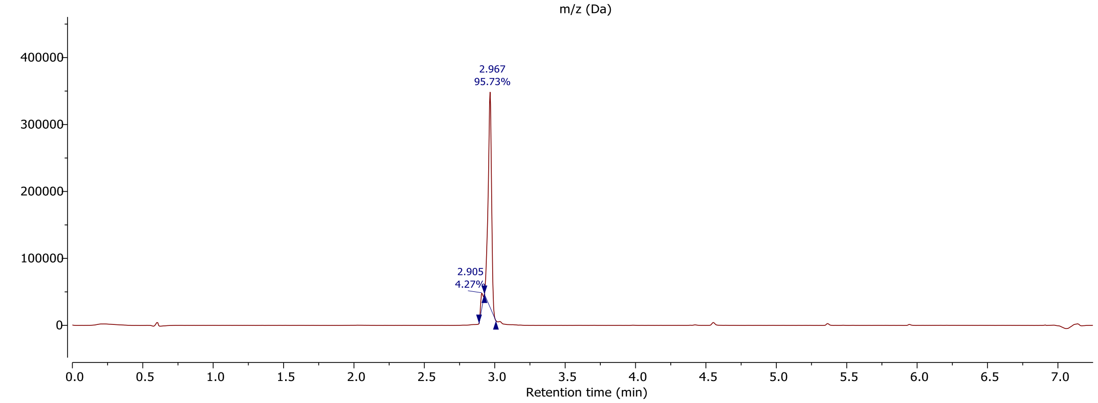
2.967
95.73%
2.905
4.27%
Retention time (min)

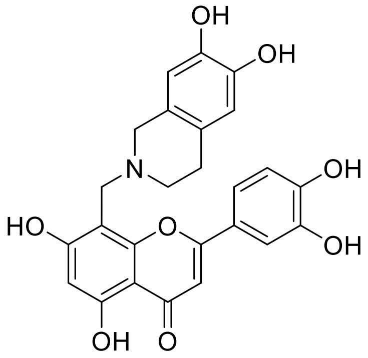
OH
OH
N
OH
HO
O
OH
OH
O

28

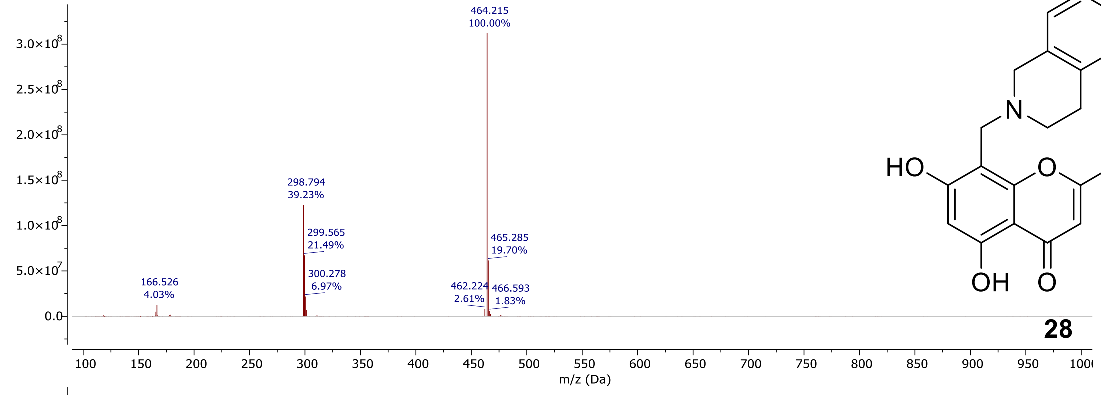
464.215
100.00%
298.794
39.23%
299.565
21.49%
300.278
6.97%
465.285
19.70%
166.526
4.03%
462.224
2.61%
466.593
1.83%
m/z (Da)

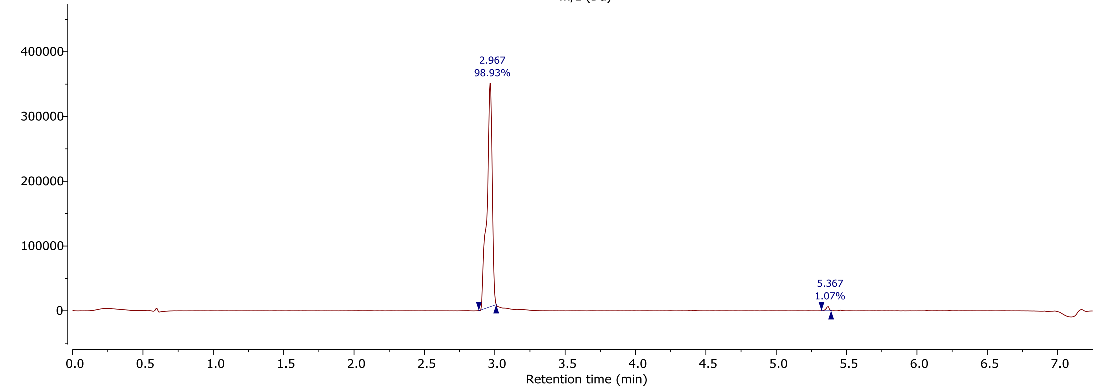
2.967
98.93%
5.367
1.07%
Retention time (min)

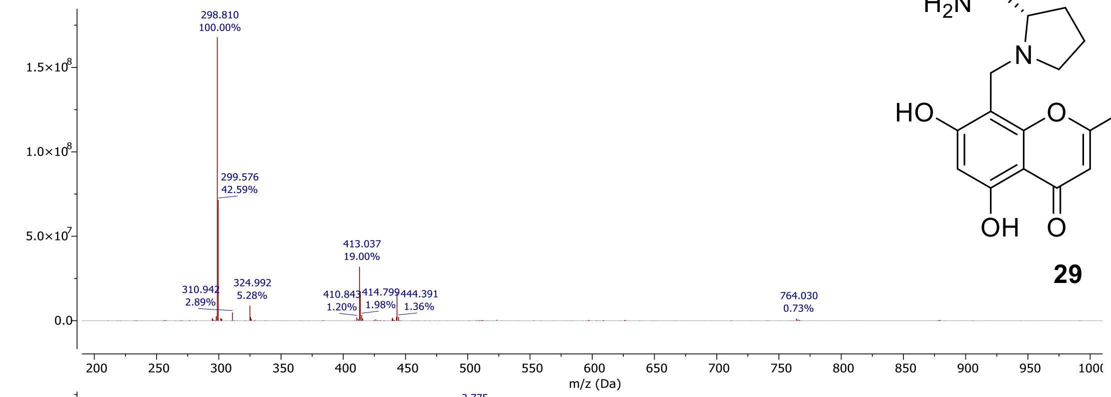
298.810
100.00%
299.576
42.59%
310.942
2.89%
324.992
5.28%
413.037
19.00%
410.843
1.20%
414.799
1.98%
444.391
1.36%
764.030
0.73%
1.5×10^8
1.0×10^8
5.0×10^7
0.0
m/z (Da)

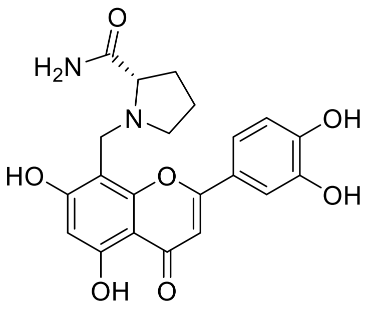
H2N
O
N
OH
HO
O
OH
OH
O

29

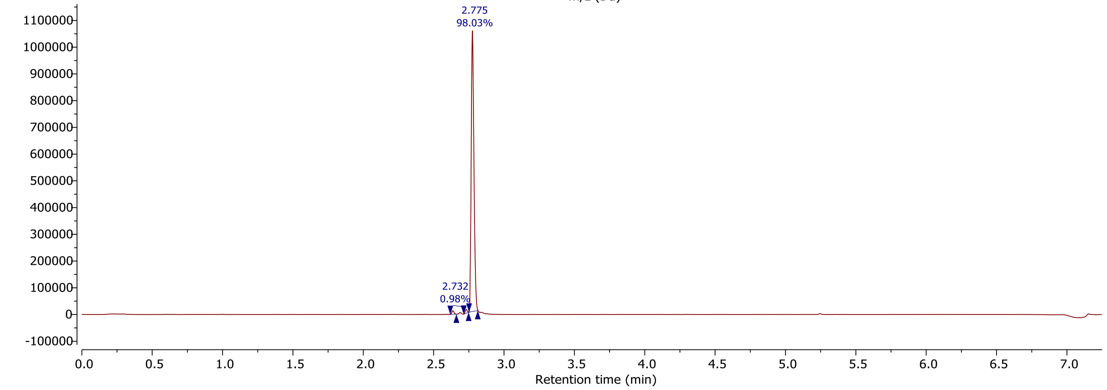
2.775
98.03%
2.732
0.98%
Retention time (min)

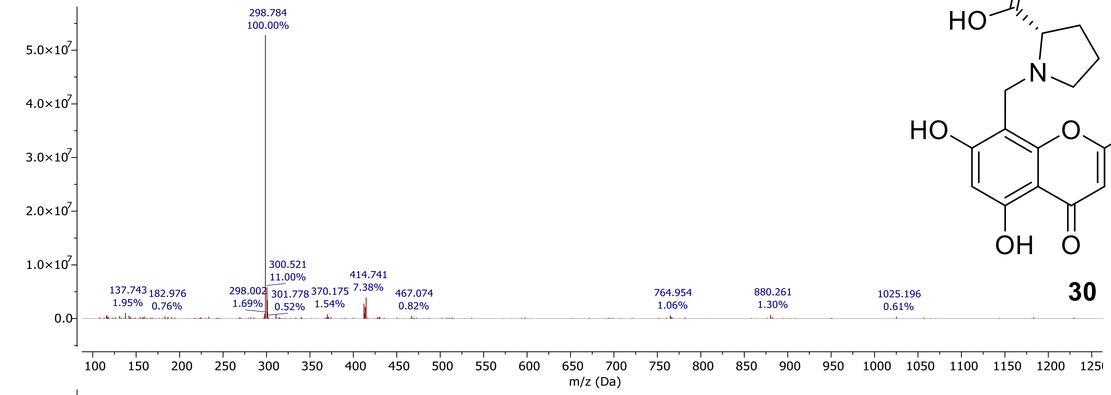
298.784
100.00%
137.743
1.95%
182.976
0.76%
298.002
1.69%
300.521
11.00%
301.778
0.52%
370.175
1.54%
414.741
7.38%
467.074
0.82%
764.954
1.06%
880.261
1.30%
1025.196
0.61%
5.0×10^7
4.0×10^7
3.0×10^7
2.0×10^7
1.0×10^7
0.0
m/z (Da)

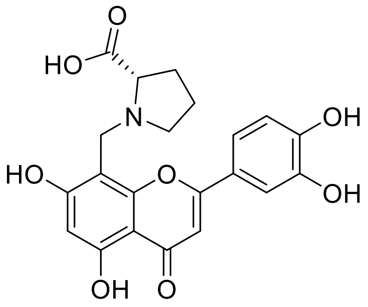
HO
O
N
OH
HO
O
OH
OH
O

30

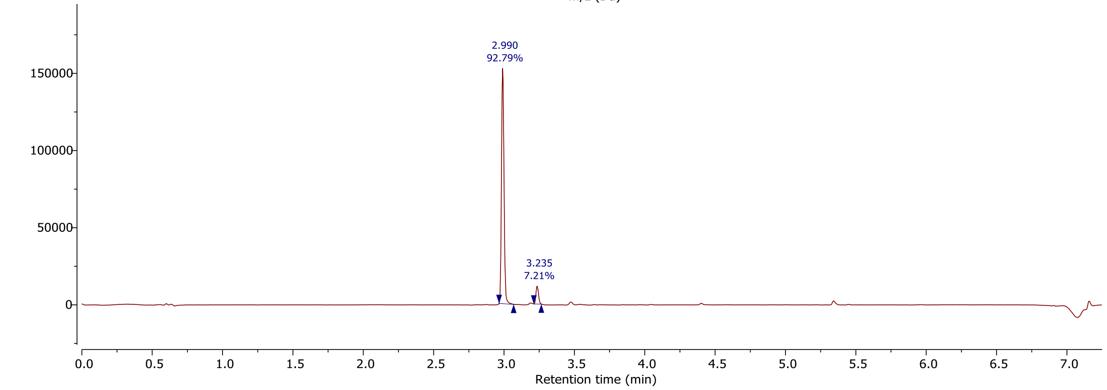
2.990
92.79%
3.235
7.21%
Retention time (min)

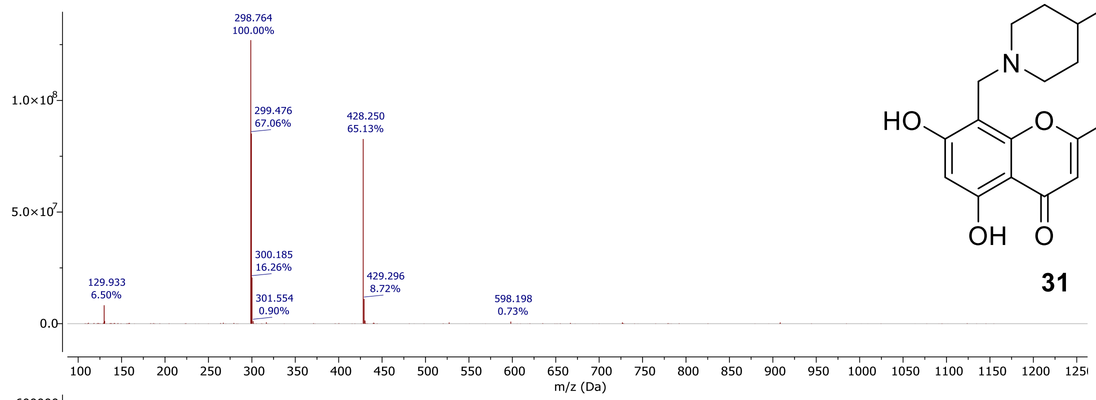
298.764
100.00%
299.476
67.06%
428.250
65.13%
300.185
16.26%
429.296
8.72%
129.933
6.50%
301.554
0.90%
598.198
0.73%
m/z (Da)

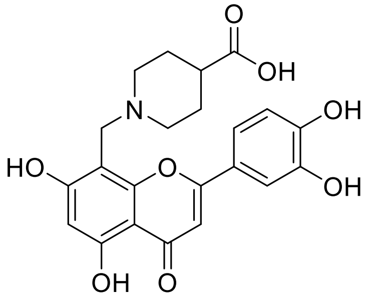
O
OH
N
OH
HO
O
OH
OH
O

31

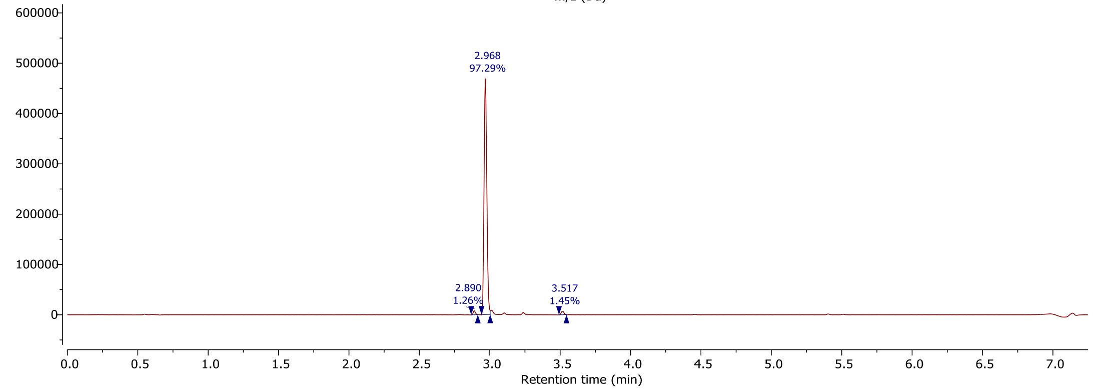
2.968
97.29%
2.890
1.26%
3.517
1.45%
Retention time (min)

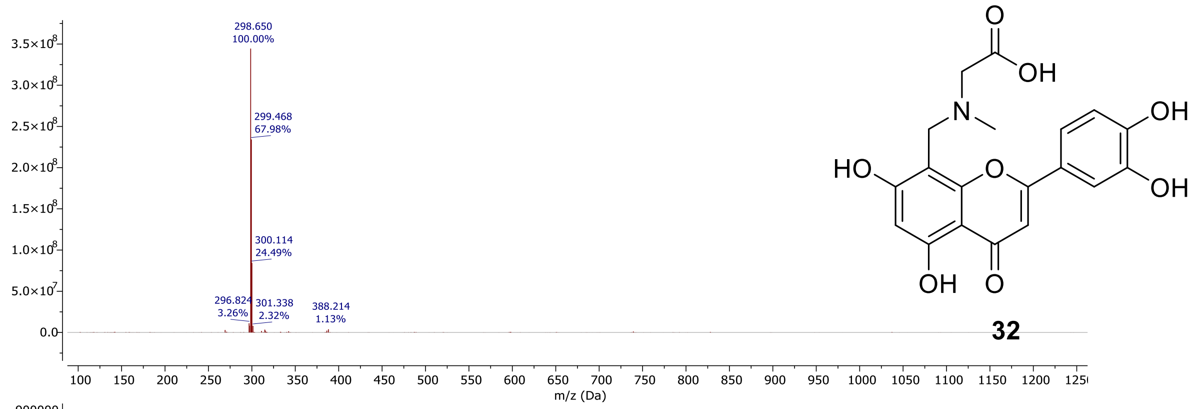
298.650
100.00%
299.468
67.98%
300.114
24.49%
296.824
3.26%
301.338
2.32%
388.214
1.13%
m/z (Da)
O
OH
N
OH
HO
O
OH
OH
O

32

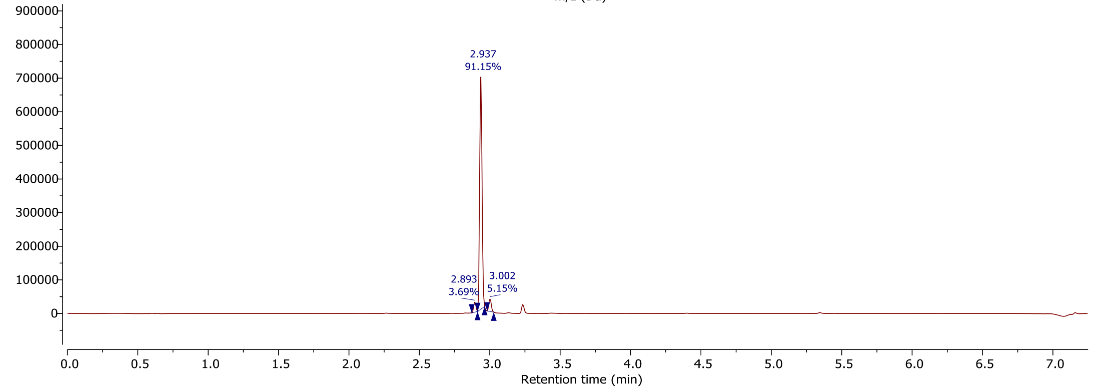
2.937
91.15%
2.893
3.69%
3.002
5.15%
Retention time (min)
